# Nemertean, Brachiopod, and Phoronid Neuropeptidomics Reveals Ancestral Spiralian Signaling Systems

**DOI:** 10.1093/molbev/msab211

**Published:** 2021-07-17

**Authors:** Daniel Thiel, Luis A Yañez-Guerra, Mirita Franz-Wachtel, Andreas Hejnol, Gáspár Jékely

**Affiliations:** 1 Living Systems Institute, University of Exeter, Exeter, United Kingdom; 2 Sars International Centre for Marine Molecular Biology, University of Bergen, Bergen, Norway; 3 Interfaculty Institute for Cell Biology, Eberhard Karls Universität Tübingen, Tübingen, Germany; 4 Department of Biological Sciences, University of Bergen, Bergen, Norway

**Keywords:** ***Key* *words:*** RFamide, agatoxin-like peptide, pleurin, APGWamide, neuropeptide, GPCRs, Trochozoa, GnRH, vasopressin, GPR139

## Abstract

Neuropeptides are diverse signaling molecules in animals commonly acting through G-protein coupled receptors (GPCRs). Neuropeptides and their receptors underwent extensive diversification in bilaterians and the relationships of many peptide–receptor systems have been clarified. However, we lack a detailed picture of neuropeptide evolution in lophotrochozoans as in-depth studies only exist for mollusks and annelids. Here, we analyze peptidergic systems in Nemertea, Brachiopoda, and Phoronida. We screened transcriptomes from 13 nemertean, 6 brachiopod, and 4 phoronid species for proneuropeptides and neuropeptide GPCRs. With mass spectrometry from the nemertean *Lineus longissimus*, we validated several predicted peptides and identified novel ones. Molecular phylogeny combined with peptide-sequence and gene-structure comparisons allowed us to comprehensively map spiralian neuropeptide evolution. We found most mollusk and annelid peptidergic systems also in nemerteans, brachiopods, and phoronids. We uncovered previously hidden relationships including the orthologies of spiralian CCWamides to arthropod agatoxin-like peptides and of mollusk APGWamides to RGWamides from annelids, with ortholog systems in nemerteans, brachiopods, and phoronids. We found that pleurin neuropeptides previously only found in mollusks are also present in nemerteans and brachiopods. We also identified cases of gene family duplications and losses. These include a protostome-specific expansion of RFamide/Wamide signaling, a spiralian expansion of GnRH-related peptides, and duplications of vasopressin/oxytocin before the divergence of brachiopods, phoronids, and nemerteans. This analysis expands our knowledge of peptidergic signaling in spiralians and other protostomes. Our annotated data set of nearly 1,300 proneuropeptide sequences and 600 GPCRs presents a useful resource for further studies of neuropeptide signaling.

## Introduction

Neuropeptides are a diverse group of neuronal signaling molecules found in most animals (Jékely 2013; Mirabeau and Joly 2013; Elphick et al. 2018; Thiel et al. 2018; Quiroga Artigas et al. 2020). Most mature neuropeptides consist of 2–40 amino acids and derive from longer proneuropeptide (pNP) precursor sequences. Precursor sequences can be a few hundred amino acids long containing one or multiple neuropeptides. The active peptides in a pNP can be interspersed with other, often nonconserved peptides (intersequences) with no known biological function (Jékely 2013; Mirabeau and Joly 2013; Christie 2017; Veenstra 2019; Takahashi 2020). Neuropeptide sequences in pNPs are generally flanked by basic residues for enzymatic cleavage (Veenstra 2000; Hook et al. 2008). The phylogenomic analysis of neuropeptides can be challenging due to the short length and often high degree of divergence of the active peptides. The intersequences are usually even less conserved between species. Nevertheless, it has been possible to reconstruct the deep evolutionary history of most bilaterian peptidergic systems by the combined analysis of pNPs and neuropeptide receptors (Jékely 2013; Mirabeau and Joly 2013; Tian et al. 2016; Thiel et al. 2018).

Most neuropeptides activate G-protein coupled receptors (GPCRs) that belong to either the class A (rhodopsin type) or class B (secretin type) of GPCRs (Caers et al. 2012; Frooninckx et al. 2012; Mirabeau and Joly 2013; Foster et al. 2019). GPCRs are several hundred amino acids long and their degree of conservation make phylogenetic analyses feasible when reconstructing the evolution of peptidergic systems (Jékely 2013; Mirabeau and Joly 2013). Another useful approach is to compare the exon–intron structure of pNPs (Mair et al. 2000; Yañez Guerra et al. 2020; Zhang et al. 2020). In several cases, the overall organization of orthologous pNP genes can be conserved (e.g., a region coding for the signal peptide followed by a single peptide and a C-terminal Cys-containing domain) (Semmens et al. 2015; Liutkeviciute et al. 2016). Furthermore, an increased sampling across taxa has often helped to clarify relationships, revealing hidden orthologs or lineage-specific losses, gains, and divergences (Jékely 2013; Mirabeau and Joly 2013; Semmens et al. 2016; Martín-Durán et al. 2017; Thiel et al. 2018; Yañez Guerra et al. 2020). Some lineages can retain signaling systems that had been lost in related species. For example, corazonin and luqin are widespread neuropeptides in protostomes but not detectable in vertebrates. These pNPs and their receptors are, however, present in ambulacrarians like the hemichordate *Saccoglossus kowalevskii* or the starfish *Asterias rubens*, indicating that they represent ancestral bilaterian signaling systems (Tian et al. 2016; Yañez -Guerra and Elphick 2020).

Among the lophotrochozoans, a major and diverse clade of bilaterians, the study of neuropeptides has mostly been limited to mollusks (Veenstra 2010; Stewart et al. 2014; Adamson et al. 2015; Bose et al. 2017; De Oliveira et al. 2019) and annelids (Veenstra 2011; Conzelmann, Williams, Krug, et al. 2013; Kerbl et al. 2017). Neuropeptide receptors are also known from mollusks and annelids (Conzelmann, Williams, Tunaru, et al. 2013; Jékely 2013; Mirabeau and Joly 2013; Bauknecht and Jékely 2015; Schmidt et al. 2018). These studies have shown that lophotrochozoans possess a relatively conserved neuropeptide complement with evidence for many ancestral bilaterian peptidergic systems (Conzelmann, Williams, Krug, et al. 2013; De Oliveira et al. 2019). In addition, mollusks and annelids possess neuropeptide duplications and a few seemingly unique neuropeptides specific to only one of these clades.

The neuropeptide complement of other lophotrochozoans—or spiralians in general—has not been sampled thoroughly (Saberi et al. 2016; De Oliveira et al. 2019) or was limited to single peptides (Thiel et al. 2017, 2019). A recent study on the evolution of neuropeptides in mollusks surveyed other lophotrochozoans as well, but only with limited taxonomic sampling (De Oliveira et al. 2019). The results provided first insights into the neuropeptide complement of nemerteans, brachiopods, and phoronids, but failed to recover several ancestral neuropeptides known from mollusks and annelids, raising the question whether this is due to extensive losses during lophotrochozoan evolution.

Here, we present a comprehensive bioinformatic survey of the neuropeptide and neuropeptide GPCR complement of nemerteans, brachiopods, and phoronids. We investigated several species in each group and were able to identify many previously undetected families. To verify cleavage patterns and the predicted active peptides, we complemented the in silico analysis with a mass spectrometric screen in a nemertean. This enabled us to reconstruct a deeply sampled complement of peptidergic systems in these organisms and to clarify major evolutionary patterns in lophotrochozoan neuropeptide evolution. We present evidence for protostome-specific expansions of signaling systems, the orthology of some arthropod and spiralian neuropeptides, and the lophotrochozoan ancestry of neuropeptides that have been described as clade specific, and describe the evolution of neuropeptide paralogs in different spiralian groups.

## Results

### Bioinformatic Identification of the Proneuropeptide Complement in Nemerteans, Brachiopods, and Phoronids

**Fig. 1.**
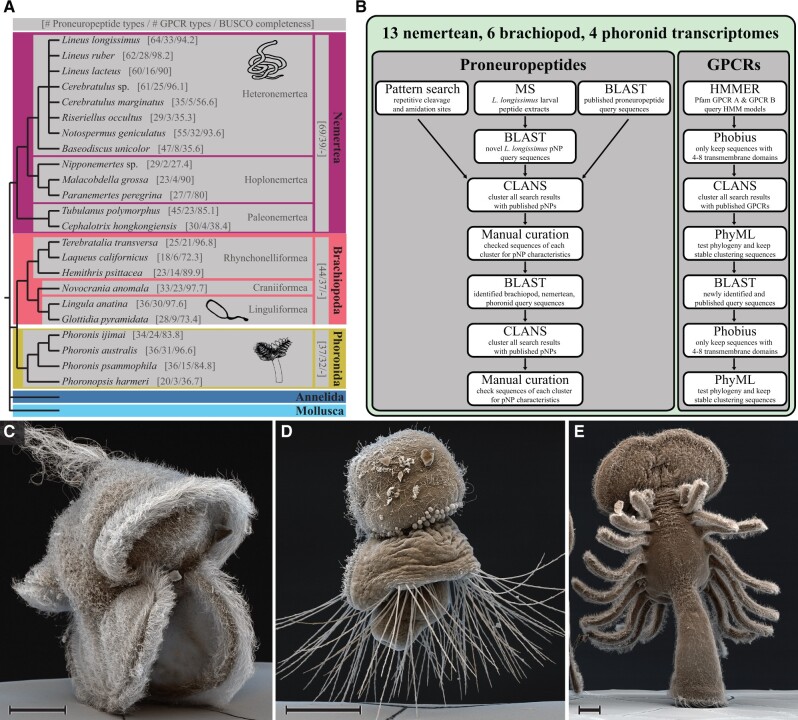
Investigated taxa and pipeline for the identification of peptidergic signaling systems. (*A*) Investigated taxa. Numbers in square brackets indicate the number of identified neuropeptide precursor types (out of a total of 72 types), number of identified neuropeptide GPCR types (out of a total of 41 known types), and BUSCO completeness of transcriptomes (in %). The depicted relationships of nemertean species are based on Andrade et al. (2014) and Kvist et al. (2015), the relationships of phoronids are based on Santagata and Cohen (2009), the relationship of brachiopod species is based on Kocot et al. (2017) and Marlétaz et al. (2019), with the taxonomic classifications according to http://www.marinespecies.org/ (status: July 2020; last accessed July 20, 2021). (*B*) Pipeline for the identification of proneuropeptides and neuropeptide GPCRs. (*C*) Scanning electron micrograph of a *Lineus longissimus* (Nemertea) larva. (*D*) SEM image of a *Terebratalia transversa* (Brachiopoda) larva. (*E*) SEM image of a *Phoronis muelleri* (Phoronida) larva. Scale bars: 50 µm.

For a comprehensive characterization of neuropeptide signaling in trochozoans, we searched for neuropeptide precursors and neuropeptide GPCRs in transcriptomes of 13 nemertean, 6 brachiopod, and 4 phoronid species and analyzed them in an evolutionary context (fig. 1).

**Fig. 2.**
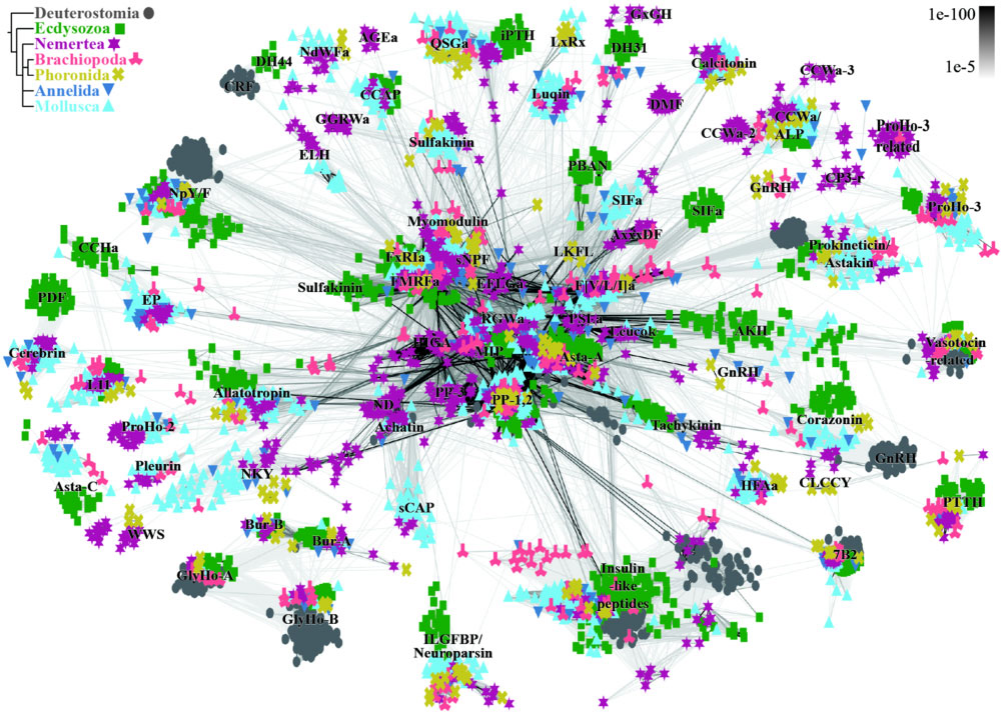
Cluster analysis of neuropeptide precursors. Connections are based on blast similarities <1e-10 as shown on the upper right. Animal groups are color and symbol coded as shown on the upper left. 7B2, Neuroendocrine protein 7B2; a, amide; ALP, agatoxin-like peptide; AKH, adipokinetic hormone; Asta, allatostatin; Bur, Bursicon; CCAP, crustacean cardioacceleratory peptide; CP3-r, CCWamide-Prohormone 3-related; CRF, corticotropin releasing factor; DH, diuretic hormone; ELH, egg-laying hormone; EP, excitatory peptide; ETH, ecdysis triggering hormone; GlyHo-A, glycoprotein hormone alpha; GlyHo-B, glycoprotein hormone beta; GnRH, gonadotropin-releasing hormone; ILGFBP, insulin-like growth factor binding protein; L11, elevenin; Leucok, leucokinin; MIP, myoinhibitory peptide/allatostin B; NpY/F, neuropeptide Y/F; PBAN, pheromone biosynthesis activating neuropeptide; PDF, pigment dispersing factor; PP, pedal peptide; ProHo, Prohormone; PTTH, Prothoracicotropic hormone; RFa’s, RFamides, Wa’s, Wamides.

To identify neuropeptide precursors, we used a combined approach of relaxed BLAST searches and pattern searches. In addition, we analyzed peptide extracts of the larvae of the nemertean *Lineus longissimus* by MS/MS (see below). The resulting list of full-length precursors was manually curated and used as a new query database in a second, more stringent BLAST search to recover further orthologs across all species that may have escaped detection. This approach improved our coverage by recovering some “hidden orthologs” (Martín-Durán et al. 2017). All full-length precursor candidates were analyzed by similarity-based clustering (fig. 2) followed by manual curation (see supplementary material 1, Supplementary Material online). Although similarity-based clustering is not a phylogenetic method, it is a powerful approach to classify large numbers of distantly related sequences where a phylogenetic analysis is problematic.

With this comprehensive strategy, we identified a total of approximately 1,300 potential neuropeptide precursor candidates including different paralogs and isoforms (supplementary materials 1–3, Supplementary Material online). We grouped these candidates into 72 orthology groups of proneuropeptide precursors, some of which constitute paralogs only found in one or more of the examined phyla (fig. 2; supplementary materials 1–3, Supplementary Material online). From these 72 groups, we found 69 in nemerteans, 44 in brachiopods, and 37 in phoronids. In all three animal groups, we detected corazonin, GnRH, two paralogs of vasotocin, sNPF, NKY, NPY/NPF, FMRFamide, myomodulin, L11, sulfakinin, allatotropin, allatostatin A (with a second paralog in nemerteans), calcitonin, Cerebrin/PDF, QSGamide, PTTH, various insulin-like peptides, glycoprotein hormone alpha and beta, 7B2/secretogranin V, CCWamide (two additional paralogs in nemerteans), F[V/L/I]amide, FxRIamide (were “x” stands for a variable amino acid), HFAamide, insulin-like growth factor binding protein related, pedal peptide 1 and 2, prohormone 3, and prokineticin. In nemerteans and brachiopods, we also identified a second prohormone 3 paralog. All of these peptides have previously been reported from annelids or mollusks and represent broadly distributed families across the lophotrochozoans.

Other broadly distributed lophotrochozoan families that we found in the nemerteans and brachiopods but not the phoronid transcriptomes include a second GnRH paralog, luqin, myoinhibitory peptide (MIP)/allatostatin B, RGWamide, excitatory peptide, allatostatin C (with a second paralog in nemerteans), DH31, pleurin, proenkephalin, prohormone 2/GNxQN, and a third group of pedal peptides from nemerteans and mollusks, which form a cluster near the pedal peptide 1 and 2 sequences.

In nemerteans and phoronids, but not in brachiopods, we also detected NdWFamide, bursicon alpha and bursicon beta, and in brachiopods and phoronids but not in nemerteans we found CLCCY. In phoronids, but not brachiopods and nemerteans, we detected orthologs of annelid and mollusk LxRx peptides. Propeptides that are known from annelids or mollusks, and that we detected only in nemerteans are achatin, CCAP, a second NKY paralog, leucokinin, tachykinin, SIFamide, EFLGamide/TRH, DH44/ELH, HIGA, and sCAP. Some peptide families represent lineage-specific innovations with no clear similarity to other neuropeptides. In nemerteans and brachiopods, we found a peptide with the common motif AxxxDF. LKFL is a phoronid-specific peptide, ND peptide and PSLamide are nemertean specific. CP3-r is a nemertean-specific Wamide group with some similarity to CCWa and prohormone 3 sequences.

The full complement of identified and annotated pNP sequences, with predicted cleavage and amidation sites of all investigated species, is given in the supplementary proneuropeptide list (supplementary material 1, Supplementary Material online).

### Mass Spectrometry Confirms Predicted Cleavage Sites and Reveals Novel pNPs

To complement our bioinformatic proneuropeptide search, we analyzed peptide extracts from larvae of the nemertean *L. longissimus* (fig. 1*C*) by mass spectrometry. Larvae of this species were comparatively easy to obtain in sufficient numbers, could be reared to different stages, and the reference transcriptome is of high quality (94.2% BUSCO completeness, supplementary material 4, Supplementary Material online). Following an MS/MS run of the peptide extracts, we screened for hits against the translated transcriptome of *L. longissimus*, using a random digestion of the target database. This allowed us to randomly test for the accuracy of predicted cleavage sites and modifications, as well as to find additional, so far unknown neuropeptides.

**Fig. 3.**
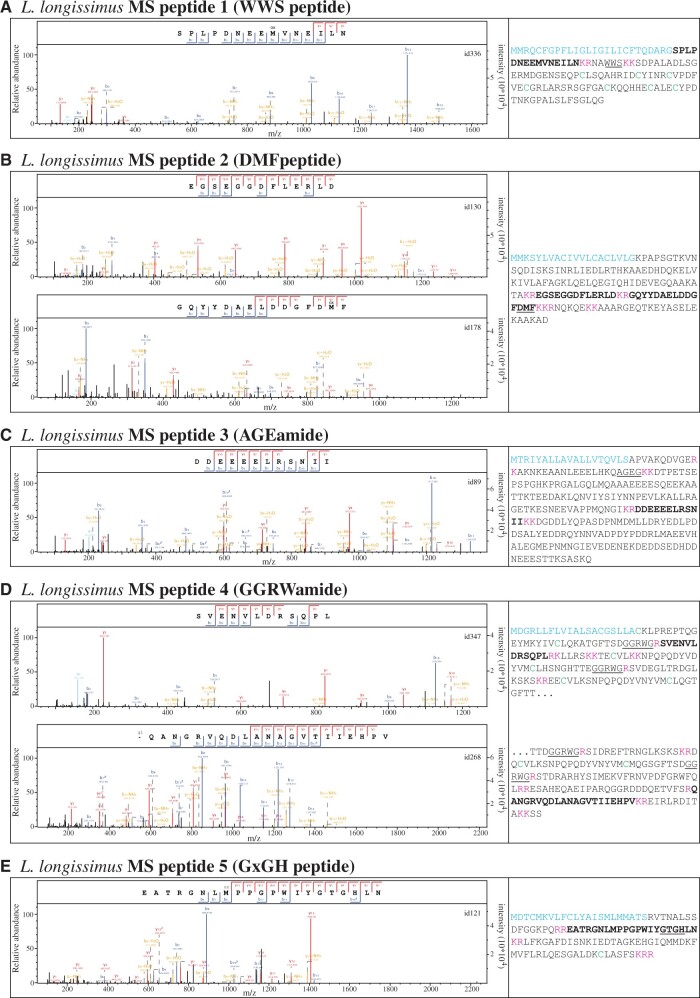
Neuropeptides discovered by mass spectrometry in *Lineus longissimus*. Spectra of newly identified peptides and their precursor sequences. *(A)* WWS peptide. *(B)* DMF peptide. *(C)* AGEamide. *(D)* GGRWamide. *(E)* GxGH peptide. Spectra of peptides are shown on the left side of the panels with the corresponding precursor sequences shown to the right. Precursor sequences are marked as follows: signal peptide in blue, detected peptide in bold, name-giving sequence underlined, cleavage sites in magenta, cysteine residues in green. The precursor of MS peptide 4 (GGRWamide) is split into two partial sequences.

We could confirm 46 peptides that were predicted between dibasic cleavage sites on 18 precursors which were identified by BLAST or motif search (Allatostatin A1, Allatostatin A2, Allatostatin C2, Allatotropin, AxxxDF, CCAP, CCWamide, Corazonin, DH44/ELH, F[V/L/I]amide, FMRFamide, Pedal peptide 1, Pedal peptide 2, Pedal peptide 3, Prohormone 2, PSLamide, RGWamide, SIFamide/FF peptide) (supplementary materials 5 and 6, Supplementary Material online). Some peptides also suggested mono-basic cleavage sites, usually N-terminal to an arginine residue with a second basic amino acid residue 3–6 positions N-terminal of the cleavage site (fig. 3*D*, supplementary material 5, Supplementary Material online). Such monobasic cleavage sites are known as alternative cleavage sites to the classic dibasic cleavage sites (Veenstra 2000; Southey et al. 2008), although they are less common than dibasic sites. We also detected peptides that were shorter than predicted, missing single or multiple amino acids at their C- or N-termini (supplementary materials 5 and 6, Supplementary Material online). For example, from the Pedal peptide 1 and 3 precursors we detected multiple peptides by mass spectrometry, including full-length predicted peptides, as well as shorter, potentially degraded versions. Many predicted modifications like N-terminal pyroglutamate formation or C-terminal amidation were confirmed (if the full N-terminus or C-terminus was present) (supplementary materials 5 and 6, Supplementary Material online).

Finally, we identified seven peptides derived from five different precursors that were not detected by BLAST or motif search (fig. 3). We added these new precursors to our BLAST screens and found homologs in other species (WWS peptide, DMF peptide, AGEamide, GGRWamide, GxGH; fig. 2, supplementary materials 1 and 7, Supplementary Material online). Only the WWS peptide (MS-peptide 1) precursor was also detected outside nemerteans, in phoronid species (fig. 2, supplementary materials 1 and 7, Supplementary Material online), whereas the other four precursor types were only identified in nemertean species (fig. 2, supplementary materials 1 and 7, Supplementary Material online). The presence of these precursors with similar sequences between predicted cleavage sites in multiple species supports the neuropeptidergic nature of these sequences.

### Receptor Analysis Reveals a Bilaterian W/Y/Famide-Activated GPCR System That Expanded into Multiple RF/Wamide-Activated Systems in Protostomes

**Fig. 4.**
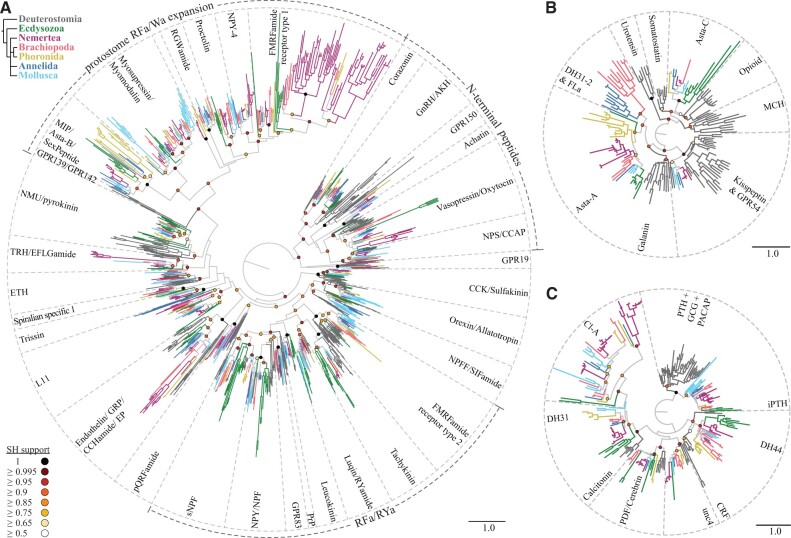
Maximum-likelihood analysis of rhodopsin and secretin-type neuropeptide GPCRs. (*A*) Rhodopsin *beta* GPCRs. (*B*) Rhodopsin *gamma* GPCRs. (*C*) Secretin GCPRs. Terminal branches are color-coded according to taxon as shown on the upper left. SH-aLRT support values of major nodes are color-coded in circles as indicated on the lower left. Scale bars on the lower right of each tree indicate the inferred amino acid substitutions per site. Dashed lines demarcate orthologous receptor types. A double crossing through a branch (nemertean FMRFamide type 1 receptors) indicates that the branch length was halfened. AKH, adipokinetic hormone; Asta, allatostatin; CCAP, crustacean cardioacceleratory peptide; CCK, cholecystokinin; CRF, corticotropin releasing factor; DH, diuretic hormone; ETH, ecdysis triggering hormone; EP, excitatory peptide; GCG, glucagon, GHS, growth hormone secretagogue; GnRH, gonadotropin releasing hormone; GRP, gastrin releasing peptide; iPTH, insect parathyroid hormone; L-DCc, lophotrochozoan DH31/Calcitonin cluster; NMU, neuromedin-U; NPS, neuropeptide-S; NPY/NPF, neuropeptide Y, neuropeptide F; MCH, melanin concentrating hormone; MIP, myoinhibitory peptide; PACAP, pituitary adenylate cyclase-activating polypeptide; PBAN, pheromone biosynthesis activating neuropeptide; PDF, pigment dispersing factor; pQRFPa, pyroglutamylated RFamide peptide; PTH, parathyroid hormone; PRP, prolactin releasing peptide; SH, Shimodaira–Hasegawa approximate-likelihood ratio test; TRH, thyrotropin releasing hormone; VIP, vasoactive intestinal peptide.

For a more comprehensive overview of the peptidergic signaling systems in nemerteans, brachiopods, and phoronids, we complemented our proneuropeptide survey with an analysis of neuropeptide GPCRs. To identify the full set of neuropeptide GPCRs, we used an initial Hidden Markov Model (HMM) search (*e*-value 1e-10) and analyzed our candidates by clustering, followed by a preliminary phylogenetic analysis. The preliminary results were then used to perform a BLAST search to find potential hidden orthologs, followed by a final phylogenetic analysis (fig. 4, supplementary materials 8–17, Supplementary Material online). With this analysis we identified and classified over 600 neuropeptide GPCR candidates from our transcriptomes.

In our analysis of rhodopsin beta-type GPCRs, we recovered three monophyletic “supergroups” of receptors that also group together in previous analyses (Mirabeau and Joly 2013; Bauknecht and Jékely 2015; Elphick et al. 2018; Thiel et al. 2018). These include deuterostome and protostome GPCRs most of which are activated by peptides that are encoded at the N-terminus of the propeptide, such as GnRH, corazonin, NG peptide, and vasotocin-related peptides, which are referred to as N-terminal peptides in Elphick et al. (2018) (N-terminal peptide, fig. 4*A*). The second “supergroup” has in common that many of their activating peptides end in RFamide or RYamide, such as short neuropeptide F, prolactin releasing peptide, neuropeptide F/Y, luqin, and the FMRFamide type 2 receptor, which are referred to as RFa-type receptors in Elphick et al. (2018) (RFa/RYa fig. 4*A*) and are represented with deuterostome and protostome orthologs (fig. 4*A*). Exceptions in this group that usually do not end in RFamide or RYamide are tachykinin and leucokinin. The third “supergroup” consists of protostome GPCRs related to RFamide and Wamide-activated receptors (fig. 4*A*). This protostome-specific GPCR expansion is in accordance with a previous study of xenambulacrarian GPCRs (Thiel et al. 2018). The individual protostome receptor types (including MIP/allatostatin B, proctolin, RGWamide, NPY-4, FMRFamide type 1, myosuppressin/myomodulin) lack direct orthologs in deuterostomes. For these families, also no directly orthologous propeptides have been found in deuterostomes to date (Jékely 2013; Mirabeau and Joly 2013). The recently reported MIP-related neuropeptides in the ambulacrarians *Saccoglossus kowalevskii* and *Apostichopus japonicus* (Zieger et al. 2021) are misidentified nonneuropeptide sequences (supplementary material 18, Supplementary Material online). The deuterostome receptor sequences most closely related to this supergroup are the GPR142 and GPR139 receptors from humans and *Branchiostoma floridae* (fig. 4*A*, supplementary material 8, Supplementary Material online). This further details a previously described relationship of protostome MIP (also called allatostatin-B) and proctolin receptors to the deuterostome GPR139/142 (Mirabeau and Joly 2013; Elphick et al. 2018). With the additional inclusion of RGWamide, NPY-4, FMRFamide type 1, and myosuppressin GPCRs, we found that these GPCRs formed together with MIP and proctolin GPCRs a protostome-only clade that is sister to the deuterostome GPR139 and GPR142 sequences (fig. 4*A*, supplementary material 8, Supplementary Material online). This two-to-many orthology relationship indicates an expansion of this group in protostomes after the divergence of bilaterians into the protostome and deuterostome lineages.

The human GPR139 has been shown to be activated by adrenocorticotropic hormone (ACTH), α- and β-melanocyte stimulating hormone, W peptides, aromatic l- and d-amino acids, and various pharmacological agonists with aromatic rings (Isberg et al. 2014; Nøhr et al. 2017; Vedel et al. 2020). GPR142 is still an orphan receptor. Protostome receptors of this expanded group are activated by neuropeptides with amidated aromatic C-termini, such as MIP [Wamide] (Kim et al. 2010; Conzelmann, Williams, Tunaru, et al. 2013; Peymen et al. 2019), myosuppressin [RFamide] (Egerod et al. 2003), RGWamide [Wamide] (Bauknecht and Jékely 2015), *P. dumerilii* neuropeptide Y-4 [RFamide] (Bauknecht and Jékely 2015), and arthropod FMRFamide [RFamide] (Cazzamali and Grimmelikhuijzen 2002; Meeusen et al. 2002).

Exceptions are the spiralian myomodulins and proctolin-related signaling systems. Myomodulins constitute the spiralian orthologs of the insect RFamide myosuppressin, but usually end in RLamide/RMamide and PRXamide (De Oliveira et al. 2019). The observation that most peptides from this supergroup possess an amidated aromatic amino acid on their C-terminus suggests that ancestral myomodulin-like peptides were also Famides or Wamides, similar to their arthropod orthologs the myosuppressins. Proctolins are nonamidated peptides usually ending with a C-terminal Thr residue and are described from insects and crustaceans (Johnson et al. 2003; Orchard et al. 2011). The peptide that activates the orthologous spiralian proctolin receptor is so far unknown. We found members of every investigated trochozoan clade in each of the individual protostome-specific GPCR types within this supergroup. The spiralian receptors that are related to the type 1 FMRFamide receptors seem to have duplicated into at least two paralogous groups after the ecdysozoan–spiralian split, with a further subsequent expansion in the nemertean lineage. The only identified ligand of one of these receptors belongs to the sister clade of the type 1 FMRFamide GPCR-related group and is the *P. dumerilii* NPY-4 […SRPRFamide] (Bauknecht and Jékely 2015). This *P. dumerilii* precursor is similar to NPY/NPF peptides from other species (Conzelmann, Williams, Krug, et al. 2013) that activate a different group of receptors related to RYamide/RFamide signaling (fig. 4*A*). It remains to be seen if the *P. dumerilii* NPY-4 peptide also activates NPY/NPF receptors and what are the lophotrochozoan ligands of the type 1 FMRFamide-related receptors.

As most protostome receptors in this expanded group are activated by peptides with C-terminal amidated aromatic amino acids and also the human GPR139 is activated by peptides, amino acids, and other antagonists with aromatic rings, it is likely that the ancestral bilaterian GPCR was activated by peptides with C-terminal aromatic amino acids. RFamides and Wamide peptides are also present outside Bilateria, in Cnidaria and Placozoa (Walker et al. 2009; Nikitin 2015; Hayakawa et al. 2019; Koch and Grimmelikhuijzen 2019, 2020; Williams 2020). Such nonbilaterian RFamide/Wamide peptides may be orthologous to the whole protostome expansion, rather than to specific bilaterian peptides. As this GPCR expansion happened within protostomes, it is likely that the corresponding proneuropeptides evolved and diversified in parallel with the receptors. This suggests that there are no direct orthologs of MIP, proctolin, RGWamide, and so forth in deuterostomes or cnidarians.

### The GPCR Analysis Reveals a Conserved Set of Lophotrochozoan Systems in Nemerteans, Brachiopods, and Phoronids

**Fig. 5.**
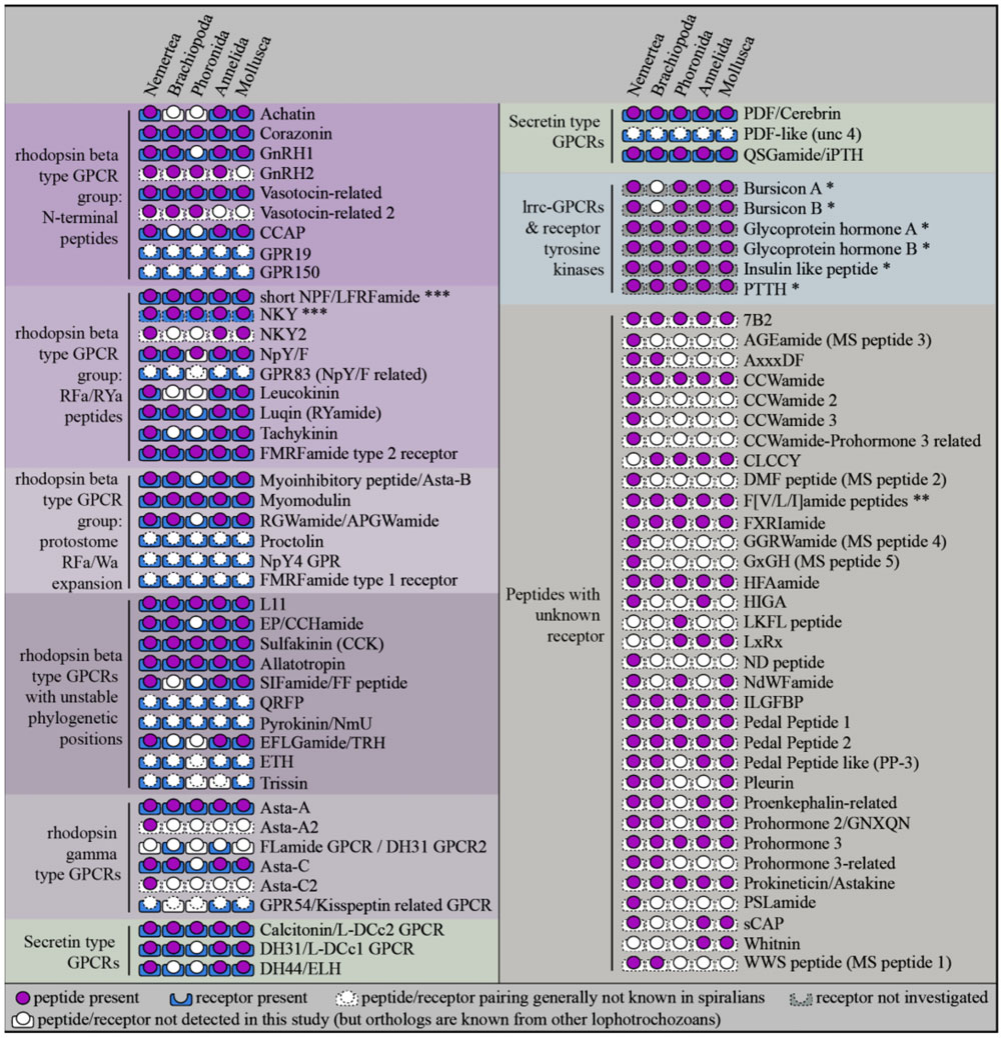
Presence of proneuropeptides and neuropeptide GPCRs. Signaling systems are divided into monophyletic GPCR groups in agreement with previous studies (Elphick et al. 2018; Thiel et al. 2018), with the exception of the rhodopsin beta type GPCRs with unstable phylogenetic positions. The presented resolution goes back to the last common ancestor of the five phyla shown, although many of the groups have deeper conservation in bilateria as also evident from the trees in figure 4. A filled circle indicates the presence of a propeptide in at least one taxon of the corresponding clade, a filled square around the lower half of the circle indicates the presence of a receptor. A white circle or square with full line indicates that the precursor or receptor was not found in any of the species of this animal group, but potential orthologs are known from other lophotrochozoans. If the circle or square has a dotted line, the corresponding precursor or receptor is generally not known in lophotrochozoans. The QSGamide/iPTH peptide–receptor pairing is assumed based on the QSGamide and iPTH orthology (Xie et al. 2020) but has not been proven in Lophotrochozoa. *The presence of leucine-rich-repeat containing GPCRs or non-GPCR neuropeptide receptors was not investigated in this study. **F[V/L/I]amides may include phylogenetically different spiralian peptides with similar C-terminal motifs. ***The depicted NKY receptors and short neuropeptide F receptors refer to the same receptor. lccr, leucine-rich repeat containing. Peptides and receptors are paired according to Jékely (2013), Bauknecht and Jékely (2015), Elphick et al. (2018), Schwartz et al. (2019), and Xie et al. (2020).

In our GPCR analysis, we confirmed 40 orthology groups of rhodopsin beta, rhodopsin gamma, and secretin-type neuropeptide GPCR families (Jékely 2013; Bauknecht and Jékely 2015; Elphick et al. 2018; Schwartz et al. 2019). Thirty orthology groups contained sequences from at least one species of nemerteans, brachiopods, and phoronids: corazonin, GnRH, vasotocin-related, CCAP, GPR150 (N-terminal peptide group of rhodopsin beta-type GPCRs, figs. 4*A* and 5), GPR19, sNPF, luqin, tachykinin, FMRFamide type 2 (RY/RFamide group of rhodopsin beta-type GPCRs, figs. 4*A* and 5) MIP/allatostatin B, myomodulin, RGWamide, proctolin, NPY-4, FMRFamide type 1 (protostome RFa/Wa expansion of rhodopsin beta-type GPCRs, figs. 4*A* and 5), L11, EP/CCHamide, sulfakinin, allatotropin, QRFP, pyrokinin (rhodopsin beta GPCRs with unstable phylogenetic positions, figs. 4*A* and 5), allatostatin A, allatostatin C (rhodopsin gamma-type GPCRs, figs. 4*B* and 5), DH44/ELH, PDF/cerebrin, the PDF-like (unc4), two DH31/calcitonin-related receptor groups (L-DCc's), and the iPTH/PTH+GCG+PACAP GPCRs (secretin-type GPCRs, figs. 4*C* and 5). The two clusters of DH31/calcitonin-related GPCRs (L-DCc1 and L-DCc2) correspond to two previously described lophotrochozoan secretin-type GPCR clusters (Schwartz et al. 2019; Cardoso et al. 2020) as further discussed below.

GPCRs related to SIFamide signaling are generally present in lophotrochozoans but were absent in brachiopods (figs. 4*A* and 5) and NPY/F, GPR83, EFLGamide/TRH, ETH, and Trissin-related receptors were missing from phoronids (fig. 4A). Our GPCR query sequences, however, only contained trissin receptor-related sequences from mollusks and it remains to be tested whether this type of receptor is also present in annelids. Achatin, leucokinin, and GPR54/Kisspeptin-related GPCRs are present in annelids, mollusks, and nemerteans but were not recovered from phoronids or brachiopods (fig. 4*A* and *B*). The rhodopsin gamma-type *P. dumerilii* FLamide receptor and DH31 receptor-type 2 were recovered as closely related, similar to a previous analysis (Bauknecht and Jékely 2015), with a group of brachiopod sequences identified as potential orthologs to the whole annelid cluster (fig. 4*B*). The only system with a known peptide–receptor pairing in which we identified a proneuropeptide but no corresponding receptor is the NPY/NPF in phoronids (figs. 4*A* and 5).

From 28 peptidergic signaling systems with known peptide–receptor pairing in lophotrochozoans (or clear orthologs in ecdysozoans) (Bauknecht and Jékely 2015; Elphick et al. 2018; Schwartz et al. 2019; Xie et al. 2020) we found at least either a precursor or a receptor in at least one nemertean, brachiopod or phoronid species (fig. 5, supplementary material 3, Supplementary Material online), with only few exceptions. In phoronids, we could detect neither proneuropeptide nor GPCR sequences related to achatin, leucokinin and TRH/EFLGamide signaling systems and brachiopods seem to lack proneuropeptides and receptors for achatin, leucokinin, and SIFamide. Our conclusion that achatin has been lost from brachiopods is in contrast to a recent publication that reports a brachiopod achatin (De Oliveira et al. 2019). This annotated brachiopod achatin sequence (lingulaAnatina.g6587.t1) (De Oliveira et al. 2019), however, is a glycine-rich nonneuropeptide sequence that spuriously clustered with the glycine-rich achatin pNPs (supplementary material 19, Supplementary Material online).

The discovery of so many peptidergic systems can be attributed to a large part to our thorough and iterative search strategy, the use of transcriptomes from multiple species, and the combined analysis of proneuropeptides and neuropeptide GPCRs.

### Trochozoan CCWamides Are Orthologous to Arthropod Agatoxin-like Peptides

CCWamide has so far been described in mollusks, annelids, phoronids, and entoprocts. Here, we identified CCWamide precursors also in nemerteans and brachiopods. We also found evidence that CCWamide is the spiralian ortholog of arthropod agatoxin-like peptides (ALPs) (fig. 2). The U8-agatoxin peptide was first identified as a venom component in spiders (Skinner et al. 1989) but ALPs have later also been found in hexapods and crustaceans (Sturm et al. 2016; Christie et al. 2020).

**Fig. 6.**
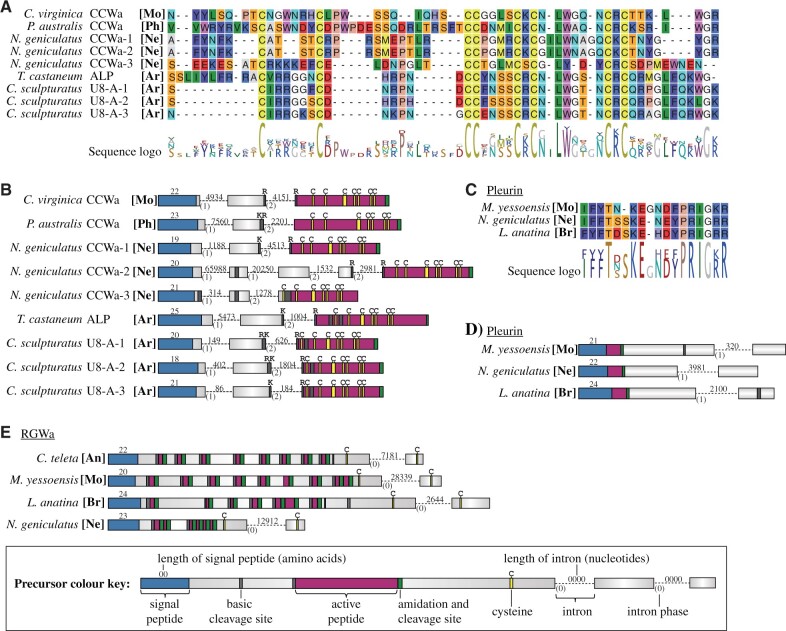
Peptide alignments and genomic precursor structure. (*A*) Alignment of CCWamide and agatoxin-like peptides. (*B*) Genomic exon–intron structure of CCWamide and agatoxin-like peptide precursors. (*C*) Alignment of pleurin peptides. (*D*) Genomic exon–intron structure of pleurin precursors. (*E*) Genomic exon–intron structure of APGWamide and RGWamide precursors. [An] annelid, [Ar] arthropod, [Br] brachiopod, [Mo] mollusk, [Ne] nemertean, [Ph] phoronid.

A comparison of CCWamide and ALP precursors reveals a conserved precursor structure of the comparably short precursors. The signal peptide is followed by a nonconserved peptide, which is separated by a cleavage site from the C-terminal CCWamide/ALP peptide. The predicted CCWamide/ALP peptides possess eight conserved Cys residues with an aromatic amino acid often occurring between the sixth and seventh Cys and an amidated aromatic amino acid at the C-terminus (fig. 6*A*).

To further assess the potential homology of CCWamide and ALP peptides, we carried out a gene structure analysis, which is a powerful tool in homology assessments for neuropeptide precursors (Mair et al. 2000; Yañez Guerra et al. 2020; Zhang et al. 2020). The gene structure comparison of CCWamides from the mollusk *Crassostrea virginica*, the phoronid *P. australis* and the nemertean *Notospermus geniculatus* with the ALP of the flour beetle *Tribolium castaneum* and the three agatoxin paralogs of the arachnid *Centruroides sculpturatus* reveals a conserved intron–exon structure of the precursors (fig. 6*B*, supplementary material 20, Supplementary Material online). The precursors are usually encoded in three different exons, except for one of the three *N. geniculatus* (CCWa2) paralogs, which is encoded in five exons. All precursors encode the signal peptide in the first exon and the mature CCWamide/ALP peptide in the last exon. We also found that in the case of all the arthropod genes, one of the cleavage sites that gives rise to the mature peptide ALP is interrupted by an intron. The second to last exon contains the sequence coding for one or two basic amino acids (K/R) in the C-terminal region of the predicted protein, and the last exon contains the region coding for one basic amino acid in the N-terminal region of the predicted peptide. We found the same pattern in the *CCWamide* gene from *C. virginica*, and the *CCWamide1* and *CCWamide2* genes from *N. geniculatus*. In the case of the gene from *P. australis*, only the two basic amino acids in the C-terminal region of the second to last exon are present. Additionally, both the CCWamide and ALP genes show a conserved exon–intron phase positioned between the second and the third nucleotide of the basic amino acid codon of the C-terminal basic amino acid. This phase is conserved in all the sequences analyzed, even the CCWamide 3 from *N. geniculatus*, which does not contain such basic amino acids in the second to last exon.

Collectively, these findings unify the family of CCWamides from lophotrochozoans and ALP peptides from ecdysozoans and demonstrate their single protostomian ancestry. To date, no receptor from either family has been identified. The fact that ALPs are described as part of spider venoms but orthologs are present in nonvenomous arthropods and in lophotrochozoans suggests that the neuropeptide has been recruited secondarily as a toxin. The evolution of toxin components from neuropeptides is a recurrent theme in toxin evolution and has also been reported in cone snails (Cruz et al. 1987; Robinson et al. 2017) and the sea anemone *Nematostella vectensis* (Sachkova et al. 2020).

### Pleurin Is Also Present outside Mollusca

Pleurin has so far been considered to be a mollusk-specific neuropeptide as no orthologs have been found in other groups (De Oliveira et al. 2019). Many mollusk pleurin precursors encode multiple pleurin peptides in the C-terminal half of the propeptide (Veenstra 2010; Bose et al. 2017; Wang et al. 2017; De Oliveira et al. 2019). Some bivalve species such as *Nucula tumidula* and *Mizuhopecten yessoensis*, as well as more distantly related neomeniomorph species such as *Neomenia megatrapezata* and *Wirenia argentea*, however, possess only a single peptide copy directly after the signal peptide (Zhang et al. 2018). Here, we identified pleurin precursors in two brachiopod and six nemertean transcriptomes. An alignment of the active peptide shows strong similarities between the peptides of the mollusks *Lottia gigantea* and *Mizuhopecten yessoensis*, the nemertean *N. geniculatus* and the brachiopod *L. anatina* (fig. 6*C*). The orthology is further supported by the presence of a single genomic intron in a similar position and with the same intron phase in all four genes (fig. 6*D*, supplementary material 20, Supplementary Material online). The presence of pleurin in nemerteans and brachiopods identifies pleurin as a trochozoan neuropeptide, which may have been lost in annelids and potentially also in phoronids. All nemertean and brachiopod pleurin precursors only have a single predicted active peptide directly after the signal peptide. The presence of only a single peptide copy in brachiopods, nemerteans, and different mollusks suggests that the multicopy nature of various mollusk pleurins evolved from an ancestral single-copy pleurin precursor.

### APGWamide Is the Mollusk Ortholog of RGWamide

Precursors encoding the RGWamide peptides have been described from different annelid species (Veenstra 2011; Conzelmann, Williams, Krug, et al. 2013; Martín-Durán et al. 2021), and the RGWamide receptor was identified in the annelid *P. dumerilii* (Bauknecht and Jékely 2015)*.* A previous study proposed that the annelid RGWamide may be the ortholog of the mollusk APGWamide (Veenstra 2011), but no further explanation was given and APGWamide was classified as a mollusk-specific peptide (De Oliveira et al. 2019). Here, we identified RGWamide precursors in nemerteans and brachiopods, and orthologs of the *P. dumerilii* RGWamide receptor in species of all five trochozoan clades (figs. 2, 3, and 5*A*). The receptor analysis reveals that the RGWamide signaling system is generally present in all trochozoans, including mollusks, and that it is part of the protostome specific RFamide/Wamide signaling-system expansion. An investigation of the precursors and mature peptides of RGWamides and APGWamides reveals several similarities which support the orthology of these neuropeptides. Most APGWamide and RGWamide precursors contain multiple peptide copies in their more N-terminal region and two cysteine residues at their C-terminus (fig. 6*E*, supplementary materials 7 and 20, Supplementary Material online). The two cysteine residues are separated by 20–30 amino acids without any RGWamide/APGWamide copy in between. The gene structure analysis of the APGWamide precursor of *Mizuhopecten yessoensis* (Mollusca), and the RGWamide precursors of *Capitella teleta* (Annelida), *L. anatina* (Brachiopoda) and *N. geniculatus* (Nemertea) reveals a single phase-0 intron between the exons coding for the two C-terminal Cys residues (fig. 6*E*). Furthermore, precursors of heteronemertean RGWamides also contain the tetrapeptide APGWamide as their most N-terminal predicted active peptide (supplementary materials 1 and 7, Supplementary Material online). In addition, several of the brachiopod and nemertean precursors encode active peptides that follow each other in direct succession, leading potentially to a cleavage into GWamide dipeptides instead of RGWamide. Such GWamide dipeptides have also been predicted in the APGWamide precursor of the mollusk *Lottia gigantea* (Veenstra 2010) and have been identified by mass spectroscopy in *Sepia officinalis* as a product of alternative processing (Henry and Zatylny 2002). Overall, our findings demonstrate that mollusk APGWamides and RGWamides from other spiralians are orthologous. We therefore suggest that the molluskan RGWamide GPCR orthologs that we used in our phylogenetic analysis (*Lottia gigantea*: XP_009046545, XP_009052844; *Crassostrea gigas*: EKC25208; supplementary materials 8 and 9, Supplementary Material online) are likely to be activated by molluskan APGWamide peptides.

### Vasotocin-Related Duplications in Spiralians and Diversification in Nemerteans

**Fig. 7.**
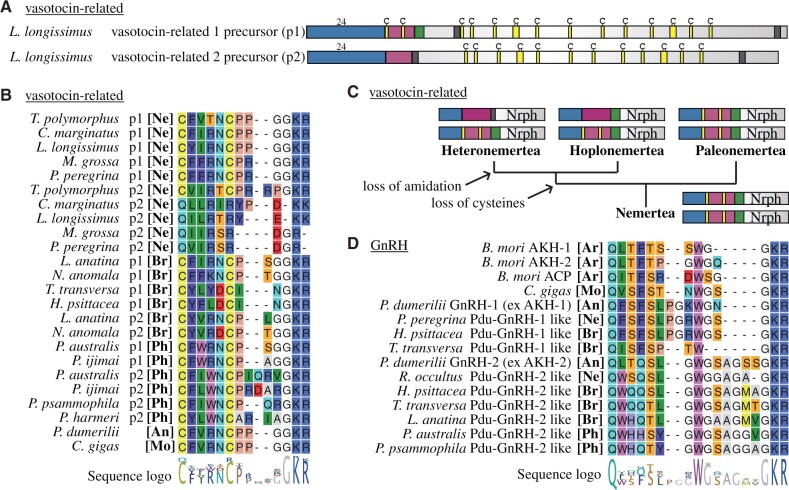
Vasotocin-related and GnRH-related peptides. (*A*) Precursor structure of *L. longissimus* vasotocin-related paralogs. Length of signal peptide in amino acids is shown above the precursors, rest is to scale. (*B*) Alignment of vasotocin-related peptides. (*C*) Evolution of vasotocin-related proneuropeptides in nemerteans. Precursors are not to scale. (*D*) Alignment of GnRH and related peptides. *B. mori*, *Bombyx mori*; *C. gigas*, *Crassostrea gigas*; Nrph, neurophysin; p1, paralog 1; p2, paralog 2; Pdu, *Platynereis dumerilii*; [An], annelid; [Ar], arthropod; [Br], brachiopod; [Mo], mollusk; [Ne], nemertean; [Ph], phoronid.

Vasotocin-related orthologs can be found throughout bilaterians. Within the vertebrate lineage, the whole signaling system (pNPs and GPCRs) expanded and led to the evolution of the paralogous vasopressin and oxytocin systems. The nomenclature of this peptide is confusing with different names across animals including cephalotocin in cephalopods (Bardou et al. 2010), asterotocin in the starfish *Asterias rubens* (Semmens et al. 2016), inotocin in insects (Stafflinger et al. 2008) and vasotocin, inotocin or mesotocin for different teleost homologs (Van den Dungen et al. 1982; Larson et al. 2006). Here, we use vasotocin-related derived from combining the names of the mammalian paralogs vasopressin/oxytocin. The peptide precursor of vasotocin-related neuropeptides is strongly conserved throughout bilaterians: The N-terminal signal peptide is directly followed by a single copy of the bioactive peptide, which is then followed by a large neurophysin domain that is characterized by conserved Cys residues (fig. 7*A*). The precursor is therefore often referred to as a combination of the active peptide (vasotocin, oxytocin, …) and neurophysin (e.g., vasotocin–neurophysin).

We detected two vasotocin-related paralogs in the linguliform brachiopods *L. anatina* and *G. pyramidata*, and the craniiform brachiopod *Novocrania anomala*, but only a single ortholog in the rynchonelliform brachiopods. We also identified two vasotocin-related paralogs in the phoronid species *P. ijimai* and *P. australis*, as well as in the paleonemertean *Tubulanus polymorphus*. In hetero- and hoplonemerteans, however, we detected a novel peptide, which is potentially related but strongly divergent. Similar to other vasotocin-related peptides, the precursor of this novel peptide encodes a single copy of the active peptide, which is then followed by a C-terminal neurophysin domain (fig. 7*A*). The predicted novel peptides, however, lack the strongly conserved cysteine residues found in canonical vasotocin-related peptides and possess an N-terminal glutamine instead (fig. 7*B*). The active peptides of hoplonemerteans are potentially amidated at their C-terminus, similar to the C-terminal amidation of classical vasotocin-related peptides. The active peptides of the heteronemertean species, however, seem to have a nonamidated C-terminus. The presence of a neurophysin-like domain in a nonvasotocin-related precursor has also been described for echinoderm NG peptides. The neurophysin domain in NG peptides, however, dates back to an ancestral bilaterian duplication of a neurophysin containing precursor that subsequently led to the evolution of vasotocin-related neuropeptides from one paralog and neuropeptide S/NG peptide/CCAP from the other paralog, as seen in extant animals (Mirabeau and Joly 2013; Semmens et al. 2015; Semmens and Elphick 2017).

To assess the evolutionary scenarios that led to the vasotocin-related paralogs in nemerteans, brachiopods, and phoronids, we calculated two maximum likelihood trees (supplementary materials 21–25, Supplementary Material online). One tree is based on the alignment of the whole precursors, excluding the signal peptide (supplementary materials 21, 22, and 24, Supplementary Material online) and the other one is based on the neurophysin domain only (supplementary materials 21, 23, and 25, Supplementary Material online). Both phylogenies suggest that the strongly diverged hoplo- and heteronemertean peptides are related to the vasotocin-related paralog 2 of the paleonemertean *T. polymorphus*, which constitutes a “classic” vasotocin-related paralog (fig. 7*B*, supplementary material 21, Supplementary Material online). This suggests that the novel hoplo- and heteronemertean peptides are strongly divergent vasotocin-related paralogs with a stepwise loss of the two characteristic cysteine residues in the ancestral lineage leading to Hoplonemertea and Heteronemertea and a further loss of the amidation site in the heteronemertean lineage (fig. 7*C*). The evolution of the brachiopod and phoronid vasotocin-related paralogs is less clear. Both trees suggest a common origin of the phoronid paralog 2 and brachiopod paralog 2 precursors (supplementary material 21, Supplementary Material online), which is the precursor that was not detected in rhynchonelliform brachiopods. The neurophysin tree further suggests a common origin of the phoronid paralog 1 and brachiopod paralog 1, suggesting that the phoronid and brachiopod paralogs are derived from a single duplication event. This is in contrast to the tree that is based on the alignment of the full precursor, which is unclear about the origin of the linguliform brachiopod paralog 1 precursors in relation to other trochozoan species. There is no support for a single duplication event in the ancestral trochozoan lineage, as annelid and mollusk species generally seem to possess only a single ortholog (Veenstra 2010, 2011; Conzelmann, Williams, Krug, et al. 2013; Stewart et al. 2014; Martín-Durán et al. 2021). We did not identify vasotocin-related GPCR duplications that generally correspond to the presence of two vasotocin-related paralogs in the investigated clades. The only duplication of vasotocin-related GPCRs that we detected was in nemerteans—specifically in several heteronemertean species and in the hoplonemertean *Nipponemertes* sp (supplementary material 9a, Supplementary Material online).

### Ancestral Duplication of GnRH-Related Neuropeptides in Spiralians

GnRH-like neuropeptides and their receptors underwent several duplications in different bilaterian lineages. The protostome–deuterostome last common ancestor already possessed two paralogous GnRH-like signaling systems called GnRH and corazonin (Tian et al. 2016; Zandawala et al. 2018). Along the arthropod lineage, the GnRH system duplicated to give rise to the paralogous AKH and ACP systems (Hansen et al. 2010; Hauser and Grimmelikhuijzen 2014; Tian et al. 2016; Zandawala et al. 2018), whereas the corazonin system has been maintained. In addition to these ancestral duplications, more recent duplications of GnRH or corazonin precursors occurred in some annelids (Veenstra 2011; Conzelmann, Williams, Krug, et al. 2013; Martín-Durán et al. 2021). We could find both corazonin and GnRH precursors and their putative GPCRs in nemerteans, brachiopods, and phoronids (figs. 4*A* and 7*D*, supplementary material 1, Supplementary Material online). In addition, we found evidence for a duplication of GnRH neuropeptides in the ancestral lophotrochozoan lineage.

There has been some confusion over the annotation of the GnRH and corazonin precursors in many trochozoan species in the past. This was recently clarified based on peptide sequence similarity (Zandawala et al. 2018) and receptor deorphanization in the annelid *Platynereis dumerilii* (Williams et al. 2017; Andreatta et al. 2020), although there is still some confusion left as one of the *P. dumerilii* GnRH-like peptides only activates a corazonin-like receptor (Andreatta et al. 2020). In short, there have been two GnRH like paralogs described in *P. dumerilii*, originally named AKH-1 and AKH-2 (Conzelmann, Williams, Krug, et al. 2013). AKH-1 was renamed to GnRH-1 based on sequence similarity of the peptide (Zandawala et al. 2018). A deorphanization assay using a heterologous expression system showed, however, that this GnRH1 peptide does not activate any of the two *P. dumerilii* GnRH GPCRs, but at high concentrations the two *P. dumerilii* corazonin receptors and was therefore referred to as GnRHL3. GnRH2 specifically activated the two *P. dumerilii* GnRH receptors (Andreatta et al. 2020). The two peptides that have originally been described as GnRH-1 and GnRH-2 (Conzelmann, Williams, Krug, et al. 2013) have been renamed to Corazonin-1 and Corazonin-2 based on sequence similarity as well as receptor activation (Williams et al. 2017; Andreatta et al. 2020). The predicted *P. dumerilii* GnRH2 peptide has a longer C-terminus than other GnRH-like peptides and to our knowledge no similar paralog has been described in any mollusk species so far. In the nemertean *R. occultus*, the brachiopods *T. transversa*, *H. psittacea* and *L. anatina*, as well as in the three *Phoronis* species we found peptides that are similar to the *P. dumerilii* GnRH-2 with its extended C-terminus (fig. 7*D*). In the nemertean *P. peregrina* and the brachiopods *H. psittacea* and *T. transversa*, we found peptides that are nearly identical to the *P. dumerilii* GnRH-1 and the GnRH that is known from mollusks (fig. 7*D*). The presence of both paralogs in the annelid, nemertean and brachiopod lineage and the lack of GnRH type 1 in phoronids and GnRH type 2 in mollusks indicates a duplication of the GnRH neuropeptide precursor in the ancestral trochozoan lineage. This duplication was then potentially followed by a secondary loss of GnRH type 2 in the mollusk lineage and GnRH type 1 in the phoronid lineage, although we cannot rule out that the lack of GnRH type 1 in phoronids is due to a lack of detection.

We could not detect any ancestral duplications of GnRH GPCRs. The GnRH GPCR branch is divided into two main branches in our analysis, but only sequences of the annelids *P. dumerilii* and *Capitella teleta* are represented in both branches, whereas brachiopod, phoronid, nemertean, and mollusk sequences are either present in one or the other branch. There may be a second GnRH receptor in annelids, as only the GnRH-2 but not the shorter GnRH-1 peptide activates the canonical GnRH receptor in *P. dumerilii* (Andreatta et al. 2020). In contrast, *Crassostrea gigas* GnRH, which is more similar to the *P. dumerilii* GnRH-1, activated the typical GnRH receptor (Li et al. 2016).

## Discussion

In this study, we analyze and describe the neuropeptide and neuropeptide GPCR complement of nemerteans, brachiopods and phoronids. Our study demonstrates how an increased taxon sampling and a comprehensive search strategy can help identify peptidergic systems and unravel their evolution. The inclusion of multiple taxa helped to identify the complement of larger taxonomic groups since even species with high-quality transcriptomes (supplementary material 4, Supplementary Material online) do not have the full set of neuropeptides or neuropeptide GPCRs in the data set (supplementary material 3, Supplementary Material online). The use of multiple search approaches, followed by the use of the initial results as new search queries helped to increase the discovery of pNPs and neuropeptide GPCRs within species. The combination of sequence alignments and gene structure analyses helped to determine the homologies of CCWamides and ALPs, as well as APGWamides and RGWamides. Using our combined approach, it was also possible to identify pleurin as an ancestral spiralian neuropeptide. Our data set of neuropeptide precursors (supplementary materials 1, 2, and 25, Supplementary Material online) and neuropeptide GPCRs (supplementary 8-11) can be used as a resource when identifying signaling systems in unexamined taxa or when trying to identify new peptide–receptor pairs.

### Several Widely Distributed Ligands and GPCRs Are Still Missing Their Partner

There are several outstanding questions about neuropeptide–receptor systems in protostomes (see also fig. 5). Of the neuropeptides analyzed here, the receptor for the lophotrochozoan CCWamides and their ecdysozoan ALP orthologs is currently unknown. This receptor is expected to be present in both ecdysozoans and lophotrochozoans and may have undergone an expansion in nemerteans, in parallel with the expansion of CCWamide pNPs (fig. 2, supplementary material 1, Supplementary Material online). Another pNP with a wide distribution in protostomes but unknown receptor is prohormone 3. We detected a second prohormone 3-like paralog in nemerteans and the brachiopod *Laqueus californicus* (fig. 2, supplementary material 1, Supplementary Material online) and a nemertean CCWamide paralog group that showed stronger connections to prohormone 3 (fig. 2, supplementary material 1, Supplementary Material online). Prokineticin/astakine-related pNPs also have no known receptor, which is exceptional, as the deuterostome prokineticin GPCRs seem to be ancestral to bilaterians but have been lost in protostomes (Mirabeau and Joly 2013; Thiel et al. 2018).

There is a large number of other neuropeptides with unknown receptors, most of which are restricted to lophotrochozoan groups without known orthologs in ecdysozoans or deuterostomes. It may be that some of these peptides activate GPCRs with unknown lophotrochozoan ligands, or receptors outside the rhodopsin or secretin GPCR family. Potential and poorly explored receptor families include peptide gated ion channels (Golubovic et al. 2007; Schmidt et al. 2018) or leucine-rich repeat-containing GPCRs like those for glycoprotein hormone or relaxin-related signaling (Jékely 2013; Roch and Sherwood 2014).

In addition to neuropeptides with unknown receptors, there are also lophotrochozoan orphan GPCRs for some of which only the deuterostome or ecdysozoan ortholog has been deorphanized. These include orthologous GPCRs of trissin receptors, kisspeptin receptors, ETH receptors, QRFamide receptors, and proctolin receptors. The molluskan peptide PKYMDT has been proposed to be a potential ortholog of the insect neuropeptide proctolin (Veenstra 2010) but an orthologous peptide has so far not been identified in any other lophotrochozoan. The endogenous ligands of lophotrochozoan FMRFamide type-1 receptors and ecdysozoan type-2 receptors are also unknown. The FMRFamide type-1 receptor was deorphanized from *Drosophila melanogaster* and is activated by different FMRFamide-like peptides with the highest sensitivity to endogenous *Drosophila* FMRFamide peptides (Cazzamali and Grimmelikhuijzen 2002; Meeusen et al. 2002). In lophotrochozoans, however, FMRFamide activates a completely different receptor (Bauknecht and Jékely 2015; Thiel et al. 2017), the FMRFamide type-2 receptor (see also fig. 4*A*). The corresponding lophotrochozoan ligand of the FMRFamide type-1 receptor is unknown. As these receptors are part of the protostome W/Y/Famide-activated GPCR expansion, it is likely that its native ligand has an amidated aromatic amino acid at its C-terminus.

Recently, the first protostome PTH GPCRs have been deorphanized in insects (Zatylny-Gaudin et al. 2016; Xie et al. 2020). The receptors belong to the group of GPCRs that are orthologous to the vertebrate PTH+GCG+PACAP receptors (Mirabeau and Joly 2013; Xie et al. 2020) (fig. 4*C*) and are referred to as cluster-B in Cardoso et al. (2020). Various orthologous lophotrochozoan sequences of the insect PTH proneuropeptide (iPTH) were published alongside the iPTH receptor deorphanization and identified as PXXXamides (Xie et al. 2020). At closer inspections, several of these lophotrochozoan iPTH/PXXXamide propeptide orthologs have been published before as QSGamides in annelids, mollusks, and brachiopods (Conzelmann, Williams, Krug, et al. 2013; De Oliveira et al. 2019). This orthology of iPTH and QSGamide proneuropeptides has also been confirmed in our proneuropeptide cluster analysis (fig. 2). It is therefore likely that the lophotrochozoan QSGamides activate the lophotrochozoan PTH GPCRs orthologs, but a final confirmation in the form of a receptor-activation assay has yet to be done.

### Open Questions from Deorphanization Experiments

There are a few studies with partially opposing results that might seem to challenge the hypothesis of a long-term peptide–receptor co-evolution (Jékely 2013; Mirabeau and Joly 2013). One of these concerns the *P. dumerilii* FLamide receptor and the *P. dumerilii* DH31 receptor type 2. This receptor group is puzzling for two reasons. The *P. dumerilii* DH31 receptor 1 is a typical ortholog of the bilaterian DH31/Calcitonin secretin-type GPCRs (fig. 4*C*). The *P. dumerilii* DH31 receptor 2 is a rhodopsin gamma type GPCR (fig. 4*B*) and no other orthologous receptor activated by DH31-related peptides is known to date. The closest related and deorphanized GPCR to the *P. dumerilii* DH31 receptor 2 is the *P. dumerilii* FLamide receptor in our tree (fig. 4*B*), which is in accordance with a previous analysis (Bauknecht and Jékely 2015). The *P. dumerilii* DH31 receptor 2 and FLamide receptor sequences, however, are more closely related to each other than to any other nonannelid sequence and their only orthologs were found in brachiopods. FLamide-like peptides with similar motifs ending in FLamide, FVamide, or FIamide are ubiquitously present in lophotrochozoans (fig. 2) (Conzelmann, Williams, Krug, et al. 2013; Bauknecht and Jékely 2015) and it seems rather unusual that no receptors were identified outside annelids and brachiopods, even though the potential ligands have been found in multiple phoronids, nemertean (supplementary material 1, Supplementary Material online), and mollusk species (De Oliveira et al. 2019). This raises the question whether there is a second type of receptor for F[V/L/I]amide peptides.

Another uncertainty relates to the receptors of NKY and sNPF peptides in lophotrochozoan. A large-scale screen identified a receptor for the two *P. dumerilii* NKY peptides (Bauknecht and Jékely 2015). In our phylogeny, this GPCR is orthologous to deorphanized mollusk, arthropod, and nematode sNPF receptors (Mertens et al. 2002; Garczynski et al. 2007; Bigot et al. 2014; Christ et al. 2018; Matsumoto et al. 2019). This grouping is consistent with a recent phylogenetic analysis of NPY/NPF and sNPF/PrRP receptors (YañezGuerra et al. 2020). Both NKY and sNPF peptides have an RY/Famide motif, albeit NKY peptides are very long (up to 43 residues compared with five–six in sNPF). However, the *P. dumerilii* NKY receptor can also be activated in the high nanomolar range by FMRFamide (Bauknecht and Jékely 2015). *Platynereis dumerilii* sNPF peptides (originally annotated as RYamide; Conzelmann, Williams, Krug, et al. 2013) have not yet been tested on the *P. dumerilii* NKY receptor and may represent the endogenous ligands. If this turns out to be the case, this would suggest that NKY peptides have a different endogenous receptor.

The secretin-type DH31/calcitonin-related receptors (L-DC receptors) have been deorphanized in the two mollusks *Crassostrea gigas* (Schwartz et al. 2019) and *Mytilus galloprovincialis* (Cardoso et al. 2020), and in the annelid *P. dumerilii* (Bauknecht and Jékely 2015). The *P. dumerilii* receptor was activated by the endogenous *P. dumerilii* DH31 peptide in the nanomolar range and groups in our analysis in the L-DCc1 group (fig. 4*C*, supplementary material 8, Supplementary Material online). DH31 is a protostome-specific calcitonin paralog (Conzelmann, Williams, Krug, et al. 2013) that has been lost in most mollusks except for polyplacophorans (De Oliveira et al. 2019), whereas calcitonin has been lost in ecdysozoans (Conzelmann, Williams, Krug, et al. 2013). In the mollusk *C. gigas*, calcitonin receptors were activated by *C. gigas* calcitonins in the nanomolar range (Schwartz et al. 2019). These receptors belong to a calcitonin receptor cluster CTR (Schwartz et al. 2019), which corresponds to cluster A in another study (Cardoso et al. 2020), and L-DCc2 in our analysis (fig. 4*C*). One of these *C. gigas* receptors (CTR1) was also identified by an earlier phylogenetic analysis as a potential calcitonin receptor (Dubos et al. 2003). In the mussel *M. galloprovincialis*, receptors belonging to our L-DCc1 cluster (referred to as CALCR in Cardoso et al. [2020] and DH31R in Schwartz et al. [2019]), however, were activated by mussel calcitonins in the micromolar range and, puzzlingly, by vertebrate calcitonins in the nanomolar range (Cardoso et al. 2020). Calcitonin and DH31 precursors coexist in annelids, nemerteans, brachiopods, and polyplacophorans. In the annelid *P. dumerilii*, only the receptor for DH31 was identified. It remains to be determined whether there is a consistent peptide–receptor pairing between GPCRs from these two paralogous DH31/calcitonin groups and the two paralogous peptides calcitonin and DH31. We identified both receptor types and both neuropeptides in the brachiopods *L. anatina* and *Novocrania anomala*, and the nemerteans *Cerebratulus* spec. and *T. polymorphus*. These or other species with both peptide and receptor paralogs can be useful when testing how the two receptor and ligand families evolved.

### Neuropeptide Signaling in Other Spiralians

Spiralian phylogeny and nomenclature have been in a flux with the clades collectively referred to as Spiralia (Hejnol et al. 2009; Edgecombe et al. 2011; Dunn et al. 2014) or Lophotrochozoa (Telford et al. 2015; Kocot et al. 2017; Laumer et al. 2017; Bleidorn 2019; Marlétaz et al. 2019). Some trees suggest a close relationship of mollusks, annelids, nemerteans, brachiopods, and phoronids (referred to as Trochozoa). In other analyses, however, Trochozoa are paraphyletic with gastrotrichs, entoprocts, ectoprocts, or platyhelminths more closely related to the individual trochozoan clades. Regardless of the exact phylogeny, with the here presented evidence from nemerteans, brachiopods and phoronids in combination with previous studies on annelids and mollusks, we have now a well-sampled complement of neuropeptide signaling systems in these major lineages. Some spiralian branches, however, still lack deeper sampling, including Syndermata, Gnathostomulida, and Micrognathozoa (Gnathifera) (Dunn et al. 2014; Laumer et al. 2017) as well as Chaetognatha (Bleidorn 2019; Marlétaz et al. 2019). One study that included three species of bdelloid rotifers from the genus *Rotaria* only identified very few pNPs (De Oliveira et al. 2019). An in-depth survey in gnathiferans with an increased taxon sampling is the next frontier in the comparative genomics of spiralian peptidergic systems and could help to clarify some of the remaining uncertainties.

## Materials and Methods

### Transcriptomic Resources

For increased taxon sampling, we collected transcriptomes of different nemertean, brachiopod, and phoronid species. Transcriptomes of the phoronid *Phoronis australis* (downloaded March 23, 2020), the brachiopod *Lingula anatina* (downloaded March 23, 2020), and the nemertean *N. geniculatus* (downloaded spring 2019) were retrieved from https://marinegenomics.oist.jp (see also Luo et al. [2018]). Transcriptomes of the phoronids *Phoronis psammophila* and *Phoronis ijimai* (originally annotated as *P. vancouverensis*; see Hirose et al. [2014] for conspecific status), the brachiopods *Glottia pyramidata, Hemithris psittacea, Laqueus californicus* and *Novocrania anomala*, the nemerteans *Cephalothrix hongkongiensis, Cerebratulus marginatus, Lineus lacteus, Malacobdella grossa, Paranemertes peregrina* and *T. polymorphus* were downloaded from 10.5061/dryad.30k4v, a public data set made available by Kocot et al. (2017). Transcriptomes of the phoronid *Phoronopsis harmeri*, the brachiopods *Novocrania anomala* and *Terebratalia transversa*, and the nemerteans *Lineus longissimus* and *Lineus ruber* were assembled according to Cannon et al. (2016). Sequencing data of the nemerteans *Baseodiscus unicolor* (SRR1505175), *Cerebratulus* spec. (SRR1797867), *Nipponemertes* spec. (SRR1508368), and *Riseriellus occultus* (SRR1505179) were retrieved from NCBI, trimmed with Trimmomatic 0.35 (Bolger et al. 2014), error-corrected with SPAdes 3.6.2 (Nurk et al. 2013) and assembled with Trinity 2.2.0 (Grabherr et al. 2011). All newly assembled transcriptomes were uploaded to https://doi.org/10.5281/zenodo.4556028. The two *Novocrania anomala* transcriptomes were merged and sequence redundancy was reduced in all final transcriptomes with CDhit-EST (Li and Godzik 2006; Fu et al. 2012) (using a threshold of 95% similarity). Transcriptomes were translated into protein sequences with TransDecoder (TransDecoder; http://transdecoder.github.io/) and a defined minimum length of 60 amino acids. A completeness assessment of each transcriptome was performed with BUSCO v4.0.6 (Seppey et al. 2019) using the protein mode and the lineage data set metazoa_odb10 (Creation date of the database: November 20, 2019, number of BUSCOs: 954).

### Identification and Analysis of Neuropeptide GPCRs

To identify potential neuropeptide receptors, we analyzed the translated nemertean, brachiopod, and phoronid transcriptomes for neuropeptide GPCRs. We did not include leucine-rich repeat containing GPCRs such as relaxin or glycoprotein hormone-related GPCRs, or non-GPCR neuropeptide receptors such as the insulin or PTTH receptor tyrosine kinases.

Multiple sequence alignments of Rhodopsin GPCR A (PF00001) and Secretin GPCR B (PF00002) were downloaded from the PFAM database (https://pfam.xfam.org; last accessed July20, 2021). The sequence alignments were used for an HMM search using hmmer-3.1b2 (Eddy 2011) with an *e*-value cutoff of 1e-10. The resulting sequences were analyzed using Phobius (Käll et al. 2007) to predict the number of transmembrane domains and only sequences with a minimum of four and maximum of eight transmembrane (TM) domains were kept. To avoid missing GPCRs that are mispredicted to have eight TM domains instead of the characteristic seven TM domains of GPCRs, we selected eight TMs as the maximum threshold. For our further analysis we used previously analyzed neuropeptide GPCRs as reference sequences (Mirabeau and Joly 2013; Bauknecht and Jékely 2015; Thiel et al. 2018; Yañez Guerra et al. 2020), complemented with sequences that were retrieved from NCBI for receptor types that were initially underrepresented. To determine whether the candidate sequences are indeed potential neuropeptide receptors, we used CLANS (Frickey and Lupas 2004) in an initial cluster analysis. The sequence candidates were then separated into rhodopsin beta, rhodopsin gamma, and secretin neuropeptide GPCRs for further phylogenetic analyses. Sequence candidates were aligned with MAFFT version 7, using the iterative refinement method E-INS-i and the standard scoring matrix BLOSUM62 (Katoh and Standley 2013). Alignments were trimmed with TrimAl in automated mode (Capella-Gutiérrez et al. 2009). Maximum-likelihood trees were calculated with the online application of PhyML 3.0 (Guindon et al. 2010) (http://www.atgc-montpellier.fr/phyml/; last accessed July 20, 2021) using the model LG+G4 which was automatically selected by the Smart Model Selection tool (Lefort et al. 2017) and aLRT-SH-like branch support with 1,000 replicates. Based on these trees, we determined robust groups of neuropeptide GPCR candidates. We then used this set of neuropeptide GPCR sequences as reference sequences for a second search in all translated transcriptomes using this time a BLASTp search (1e-50). To include potentially new receptor types or paralogs that might have been missing in our reference database (hidden orthologs), we also included transcriptomes of *Crassostrea gigas* and *Daphnia pulex* in this second search (downloaded from http://metazoa.ensembl.org, 05.05.2020; last accessed July 20, 2021). All new candidates from the BLAST search were added to the existing list, and a second phylogenetic analysis was carried out using the same methodology as before.

### Identification and Analysis of Neuropeptide Precursors

To identify neuropeptide precursor sequences, we combined a relaxed tBLASTn search (1e-1) with a pattern search and then used the combined results as new reference sequences in a second, more stringent tBLASTn search (1e-5). As query sequences we compiled a reference database of neuropeptide precursor sequences from previously analyzed data sets (Conzelmann, Williams, Krug, et al. 2013; Jékely 2013; Adamson et al. 2015; Bose et al. 2017; De Oliveira et al. 2019; Xie et al. 2020; Martín-Durán et al. 2021), partially complemented with sequences retrieved from NCBI (supplementary materials 26 and 27, Supplementary Material online). We used this database for a first, relaxed tBLASTn search in our transcriptomes. For the pattern search, we scanned the translated transcriptomes for potential neuropeptide precursors that code for multiple peptide copies using regular expressions. To reduce a high number of false-positive hits in the pattern search, we first sorted for sequences that possess an N-terminal signal peptide with the command line version of SignalP 4.1 (Petersen et al. 2011). We then used Unix bash regex commands to scan these secretomes for sequences with at least three repeats of amidation sites that are separated by 2–25 amino acids or at least five repeats of dibasic cleavage sites that are separated by 5–25 amino acids (grep -E -B 1 -e “((.{2,25}G[KR][KR])|(.{2,25}[KR](.{1}|.{3}|.{5})G[KR*])){3,}”-e “(.{5,25}[KR][KR]){5,}” “input_transcriptome.fasta” | grep -E -v “[-][-]” > output_file.fasta). The hits of the multicopy peptides from the BLASTn and regex search were combined for each species, sequence redundancy was reduced using CDhit (threshold 0.95), and all candidates longer than 650 amino acids were deleted. The resulting proneuropeptide sequence collection was clustered together with the reference sequences using CLANS (threshold 1e-5). Nonconnected sequences were deleted after manual inspection and samples from every cluster were taken, blasted on NCBI, and checked for the existence of a signal peptide. The general precursor structure was then manually curated based on the presence of basic cleavage sites, amidation sites, and conserved cysteines. All detected nemertean, brachiopod, and phoronid neuropeptide precursors were then used as new reference sequences in a second, more stringent tBLASTn search (threshold 1e-5) to detect potential hidden orthologs. New sequences were added to the existing collection and the precursors were checked again. The final groups of sequences were manually annotated based on precursor structure, peptide motifs and similarity to known neuropeptides. Analysis of the exon–intron structure of genes encoding different neuropeptide precursors was performed using the SPLIGN online alignment tool (Kapustin et al. 2008).

### Peptide Extraction and Mass Spectrometry (LC-MS/MS)

Peptide extraction and mass spectrometry were done as previously described (Thiel et al. 2018) with slight modifications. We collected about 100 lab-spawned larvae of the nemertean *L. longissimus* in different stages. Specimens were starved for 1 day and rinsed several times with sterile seawater. Larvae were collected into an Eppendorf cup and centrifuged for 30 s in a table-top centrifuge. The seawater was replaced with ultrapure water, larvae were immediately centrifuged again, and the water was replaced with an extraction buffer (90% methanol, 9% acetic acid, 1% distilled water). The mixture was ground with a pestle and vortexed vigorously. The suspension was centrifuged at about 13,000 × g for 20 min at 4 °C. The supernatant was collected, evaporated in a vacuum concentrator, and dissolved in 300 µl ultrapure water. Neuropeptide mixtures were reduced and alkylated as described in Borchert et al. (2010) and desalted with C18 StageTips (Rappsilber et al. 2007).

LC-MS analysis was carried out on an EasyLC nano-UHPLC coupled to a Q Exactive HF mass spectrometer (both Thermo Fisher Scientific). Separation of the peptide mixture was done as previously described (Kliza et al. 2017) with slight modifications. Peptides were eluted with an 87-min segmented gradient of 10–33–50–90% HPLC solvent B (80% acetonitrile in 0.1% formic acid). The mass spectrometer was operated in the positive ion mode. Full scan was acquired in the mass range from *m*/*z* 300 to 1,650 at a resolution of 120,000 followed by higher energy collisional dissociation (HCD) fragmentation of the seven most intense precursor ions. High-resolution HCD MS/MS spectra were acquired with a resolution of 60,000. The target values for the MS scan and MS/MS fragmentation were 3 × 10^6^ and 10^5^ charges with a maximum fill time of 25 and 110 ms, respectively. Precursor ions were excluded from sequencing for 30 s after MS/MS. MS/MS on singly charged precursor ions was enabled. The acquired MS raw files were processed using the MaxQuant software suite v.1.5.2.8 (Cox and Mann 2008).

Extracted peak lists were submitted to a database search using the Andromeda search engine (Cox et al. 2011) to query target–decoy databases consisting of the translated *Lineus longissimus* transcriptome sequences and commonly observed contaminants (285 entries). No enzyme specificity was defined. The minimal peptide length was set to four amino acids. The initial precursor mass tolerance was set to 4.5 ppm, for fragment ions a mass tolerance of 20 ppm was used. Carbamidomethylation of cysteines was defined as fixed modification in the database search. A set of expected variable modifications was defined in the database search: pyroglutamate formation of N-terminal glutamine, oxidation of methionine, acetylation of the peptide N-terminus, amidation of the peptide C-terminus, and sulfation of tyrosine. False discovery rates were set to 1% at peptide, modification site, and protein group level, estimated by the target/decoy approach (Elias and Gygi 2007). The data are available via ProteomeXchange with identifier PXD023147.

## Supplementary Material

Supplementary data are available at *Molecular Biology and Evolution* online.

## Supplementary Material

msab211_Supplementary_Data
