# Supplementary material for: Nemertean, Brachiopod, and Phoronid Neuropeptidomics Reveals Ancestral Spiralian Signaling Systems
Source: Mol Biol Evol. 2021 Jul 17;38(11):4847–66. doi: 10.1093/molbev/msab211 (PMC8557429; doi:10.1093/molbev/msab211)
Supplement: msab211_Supplementary_Data [file msab211_supplementary_data.zip › Supplementary_Material_01_Proneuropeptide_list.docx]

­## 7B2

>7B2_Phoronida_Phoronis_australis_TRINITY_DN298815_c0_g1_i2.p1

MFLVFTLASVCLCATHGLADKYDDLFLQEILRQIEQSNTGSQDYGDDYGSLPYKQNYGDFIDDVPQPVSNYQDISGADQMTDPKLPDVPVPAQPRDNEFEAQNTNMGYQYMSGGAGEVDADGHVKDPEFQEVKTDAGLPSYCNPPNPCPKGKTSEKDNCQDNVPDTGAFNQRYVQWQMRRGDCACDTEHMFPSSCPKGAEKISSRP­EESQTKLDDMDSLISQMLGMETQNENPFVSHGEKRGTLVAKKGSSHRDKRSINPYMDGEILKTVEKKAPHVGQQQHFDENDTKPYTFEYMEKMFGSTA

>7B2_Phoronida_Phoronis_ijimai_(P._vancouverensis)_comp95755_c0_seq6.p1

EVKTDAGLPSYCNPPNPCPKGKTSEKDNCQDNVPDSGAFNQRYVQWQMRRGDCGCDTEHMFPKSCPKGAEKIST

>7B2_Phoronida_Phoronopsis_harmeri_c131685_g2_i2.p2

LSFLWGKGLVGYCQDNVPDSGDFNQRYVQWQMKRGDCGCDTEHMFPASCPKGAEKISSHPEEPAPSISEMDSLISDILGTDTRSENPFVAQGEKRATLVAKKGSYHRTKRSVNPYLDGDNIQTVAKKAPPMMAHEDHNYNVNMMSPAAFAEMEKMFGSH

>7B2_Phoronida_Phoronopsis_harmeri_c131685_g2_i4.p1

RLCPLSSLRVPFIIYVYQCAGATDNCQDNVPDSGDFNQRYVQWQMKRGDCGCDTEHMFPASCPKGAEKISSHPEEPAPSISEMDSLISDILGTDTRSENPFVAQGEKRATLVAKKGSYHRTKRSVNPYLDGDNIQTVAKKAPPMMAHEDHNYNVNMMSPAAFAEMEKMFGSH

>7B2_Phoronida_Phoronis_psammophila_comp67737_c0_seq1.p1

EAQNNKWGYQYMSGGAGEVDSEGNVKDPDKQEVKTDAGLPSYCNPPNPCPKGKSSKTDNCQDNVPDTSEFNQRYVQWQMRRGDCGCDTEHMFPDSCPKGAEKISTRPEQAENKLDDIDSLISEMLGIESESDNPYVSHGEKRATLVAKKGSAPRSKRSTGTNPYLQGEKLGIVAKKAPPMEHMRTSFQDPMFNSFP

>7B2_Brachiopoda_Novocrania_anomala_Nano.rna.tri2.25330.1.p1

MNLLVFTTTILALFQLSYTYVYDNLDSNLYLRDMLMDLANRDSQIPPDDSDVIPDNRLGQNWAEDDEQSDMPALPDDVYIPYQYSTESEDEFKPENGEEADLEGTPITVAETTALNPQKPARDQEKPHQSALWGYQYVSGGAGEGKQHLTPSGGQENKQEIKTDALLPAYCNPPNPCPKGYTEKDNCQPNARDTVAFNRKYNAWEQQTGACKCDKEHMFHCPNSDNTVTSKGQGEASKNEAILSNMLDTAMKSEDGDALDNIIRSMVEKEHDEGTNPFEQGEKRGSLVAKKFHSINKRFSAEDMDGISDEENKVEANPYLTGDRLRIVAKKSPPLM

>7B2_Brachiopoda_Hemithris_psittacea_comp22607_c0_seq1.p1

MNFEMWRLQSIFFCLAISIFAECSCQTFGDGQHMDADILKDVLANYVSGQKDGSYYGIPNYSDIDGVGLYQGNHDSQAGVEDPTYSSSVSNNVDEDLTEESTTPQSDSKSTGNEQVGESDGTSSEPLEEKPQQKSTLQTKDHEFLSHNPIWGSQYISGGAGEGKQLLQPNGEIENKPEIKTDNLPAYCSPPNPCPKGFTALDGCQEHIPDTVDFNRRYNYMQLVRGSCSCDSEHMFNCPAGFDEISAQKPADAGRDAGRDSSDPLDRVIANLLSEKNGADNPYAKGNKRNTLVAKKVPRHKRATYHNTEAFQATFFGKNLKNRRLPIVAKKGAL

>7B2_Brachiopoda_Terebratalia_transversa_Ttra.rna.tri.7059.1.p1

MTVLLFLFVTSFFTCQVIADSRQYDLNEQVPSNDVILRQVLTHLSRNDNYYDNYLNPMVHQSTTSDDKFDSQGGIEQSTDVVDSGANVDENSTEINNEDKMADSSEIKEKSFLAPPKLDGKTVQTKDHEYLGQSSLWGNQYISGGAGEGKQHLGPNGDKDNHQEIKTDPDLPGYCSPPNPCPKGYTAKDGCQEHIPDTVAFNRRYNYMQLARGSCSCDSEHMFNCPAGYDKIASQQKDDQSSDPIDVLVERLLSGSDGEKENPYARGVKRSSLVAKKFRTKRAYSKNQMKRNPYFSGERHIIVAKKGRSPYNKSL

>7B2_Brachiopoda_Laqueus_californicus_comp45123_c0_seq5.p1

MTTVLLLFLTCFFTCHVIGDSSSQYDIDGQGPSNDAILRQVLTHFTRNNNYYNNLLLNDAIAYQSTPSVDKFDSQEKSADNEDDDSGEKIGDVNSTEINKEDKMAESAEIQGHQSYPPTKLDGKSVQTKDHEYLGQSSLWGNQYISGGAGEGKQHLVGPNGDKDNHQEIKTDPDLPGYCSPPNPCPKGYTAQQDGCQEHIPDTVSFNRRYNYMQLARGSCSCDSEHMFNCPAGYDKIASQQKSDQTSDPIDVLVERLLSGGDDEKENPYARGVKRNSMVAKKFRTN

>7B2_Brachiopoda_Lingula_anatina_comp150898_c0_seq1.p1

MRLSTYMLFAVMATILTFSHASYYEDNDALFREMLAEMEENEREKQYGVDGSYDPYAAFEDGDKYDDSYGPFAEANLNPTVLYNPGIEKPDSNDELAGDDPRSADIRDQEKENQNMLWGYQYVSGGAGEGKQHLVGNIDNSMKQEVKTDAALPAYCNPPNPCPKGKTAAKDNCQENIPDTAAFNQRYNNWQQKSGACKCDNEHMFNCPDGATVSSKKAYGDDYGSDELDNLSNPFSQGSKRDSLVPKKHLHKREAEKVMKDPIKLPNPNMILTDNSNLRIVAKKSPPLY

>7B2_Brachiopoda_Lingula_anatina_comp150898_c0_seq3.p1

MRLSTYMLFAVMATILTFSHASYYEDNDALFREMLAEMEENEREKQYGVDGSYDPYAAFEDGDKYDDSYGPFAEANLNPTVLYNPGIEKPDSNDELAGDDPRSADIRDQEKENQNMLWGYQYVSGGAGEGKQHLVGNIDNSMKQEVKTDAALPAYCNPPNPCPKGKTAAKDNCQENIPDTAAFNQRYNNWQQKSGACKCDNEHMFNCPDGATVSSKKAYGDDYGSDELDNLVKSLLGENTKTRDEESNPFSQGSKRDSLVPKKHLHKREAEKVMKDPIKLPNPNMILTDNSNLRIVAKKSPPLY

>7B2_Brachiopoda_Glottidia_pyramidata_comp43425_c0_seq5.p1

MGYSTTILLVVMVTAVTFTHASYYEDNDALFREMLAQMEDANEQAKQYGDEISYDPYAPYVEDEDKYERNYEPYAGDVYTAPVLTDPQVVKSESDHQAGDDPRSTDIRDQEKEHQNMLWGYQYVSGGAGEGKQHLVGNLDNSMKQEVKTDAALPAYCNPPNPCPKGKTAAKDNCQENIPDTGAFNQRFNEWQQKSGACKCDNDLDALVKSLLGENTKTREEEKNPFSQGSKRDSLVPKKHLYKREAETDMIYQNIKFPEADILGRENSGLRVVAKKSPPQF

>7B2_Brachiopoda_Glottidia_pyramidata_comp43425_c0_seq3.p1

MGYSTTILLVVMVTAVTFTHASYYEDNDALFREMLAQMEDANEQAKQYGDEISYDPYAPYVEDEDKYERNYEPYAGDVYTAPVLTDPQVVKSESDHQAGDDPRSTDIRDQEKEHQNMLWGYQYVSGGAGEGKQHLVGNLDNSMKQEVKTDAALPAYCNPPNPCPKGKTAAKDNCQENIPDTGAFNQRFNEWQQKSGACKCDNEHMFNCPDGATVSSKKTSNDAYDNNDLDALVKSLLEPVQSGLEARLVGSQETSI

>7B2_Brachiopoda_Glottidia_pyramidata_comp43425_c0_seq1.p1

MGYSTTILLVVMVTAVTFTHASYYEDNDALFREMLAQMEDANEQAKQYGDEISYDPYAPYVEDEDKYERNYEPYAGDVYTAPVLTDPQVVKSESDHQAGDDPRSTDIRDQEKEHQNMLWGYQYVSGGAGEGKQHLVGNLDNSMKQEVKTDAALPAYCNPPNPCPKGKTAAKDNCQENIPDTGAFNQRFNEWQQKSGACKCDNEHMFNCPDGATVSSKKTSNDAYDNNDLDALVKSLLGENTKTREEEKNPFSQGSKRDSLVPKKHLYKREAETDMIYQNIKFPEADILGRENSGLRVVAKKSPPQF

>7B2_Nemertea_Malacobdella_grossa_comp21872_c0_seq1_m.19224

MSFGVIHVASAFFICLIGMNALPHSEKVFLERLFDMELHEKPFHEGFDYVNEDQQEHPMFPDNVLPHFSEYDVIGERPEDIFPGRNLQFHHDSAQYGSMAQEIRSNDLKDPETGQQSSLWGTHYVSGGAGEGNQVLKPNGQERNRQEVKTDNVLPAYCEPPNPCPKGYSETNNCMDDVNDTPPYNRIYIEEQQQSGYCACDTEHMKHCPNDKPRIDANMYDTPDKTDLELAHPLNENNMDGGVAKKAPRILIQSLLTKMKAPYGINPYFNGFKLRTVAKKSPQAIDSDRLDY

>7B2_Nemertea_Paranemertes_peregrina_comp26069_c0_seq1_m.33834

MMSLTLSTAAVLLVCLVGLNAYRYPDTELLARLYDLEMRRQPLFEDDPRYQSLQLEEEHPMVLDDILPYFPEEEMDDTDYYHDNDNNNIQIRDDTEPYSQTDVHSNDPRVPETAQQSALWGTHYVSGGAGEGIQLLKPNGEERNKQEVKSDNALPAYCEPPNPCPKGYTESANCVEGVGDTPNFNRKYILAQQESGYCACDSEHMKHCPHDKPKIDTKPAATEADIMTALNKQDLDLDEKRRMYSRVAKKAPRMYKRSTRLLG

>7B2_Nemertea_Paranemertes_peregrina_comp32113_c0_seq1_m.90944

MTFVRMEAALVLLCLVGLNAFRYPDEEILERLYDLEMRQQPLFRDDPRYQSLQQQEEEHPMVLDSILPYFPEEGEDDEDESSQGSDYYRENNNIQPRDDADQYLRTAAELRSNDIRDPEAAQQSALWGTHYVSGGAGEGKQVLKPNGEEKNKLEVKSDNALPAYCEPPNPCPKGYTESDNCVRAVKDTPAFNRKYILSQQESGYCTCDSEHMKQCPHKAKMGSKQDSTEADILNALNKQDIQIDAKRRLFNRVAKKSPRMFKRSIRLDEDRNPYIGGPKLRTAAKKSPPAMEWDKWTHYNKMADDERV

>7B2_Nemertea_Paranemertes_peregrina_comp26069_c0_seq2_m.33838

MMSLTLSTAAVLLVCLVGLNAYRYPDTELLARLYDLEMRRQPLFEDDPRYQSLQLEEEHPMVLDDILPYFPEEEMDDTDYYHDNDNNNIQIRDDTEPYSQTDVHSNDPRVPETAQQSALWGTHYVSGGAGEGIQLLKPNGEERNKQEVKSDNALPAYCEPPNPCPKGYTESANCVEGVGDTPNFNRKYILAQQESGYCACDSEHMKHCPHDKPKIDTKPAATEADIMTALNKQDLDLDEKRRMYSRVAKKAPRMYKRSTRAEERKSQFISGPRLHTAAKKSPLSMKRNKWSHYERALQNDRRAWKRQNMS

>7B2_Nemertea_Tubulanus_polymorphus_comp28823_c0_seq1_m.20303

MMTTHRTSLASVLLLASFLTAGLGDITYDYNYDSSPQTDNQYQRLQKQGYSDYNFGDDEGLYPSNFYDNTDQDDGDMRDYVDEKGYDDTEGYNKHSSQYEGDDEFQEKDTQLRSNDPRDPEKMQQSSLWGTHYVSGGAGEGKQHLKPNGEEDNKQEVKSDAGLPAYCEPPNPCPKGFTEDQNCRKTVKDTADFNRKYILSQQDSRYCACDSEHMKHCPRNQPKIDTKQDASANLKEILAEENLGKNPFVKGNKRNKLVAKKRPAKRSKRNVHVKGNPYLSGEKLNIAAKKSPPDFPFKKWDHFNKMMESSIY

>7B2_Nemertea_Lineus_longissimus_c47447_g1_i2.p1

MSYIYFSACLVTLALIGGARADPAYRDILDGIYQYRNLRNDGYFDTIPAFEELEKDNEPTDAIPDDMSYYPQYGWEGDQPEEDDNDLASIGLADPYGYEEDEEAQAQEDDQQGPFQLRSTNDLRDPELLENSALWGTHYVSGGAGEGKQHLKPNGEQKNKQEVKSDAALPAYCEPPNPCPKGYSEDKNCQKDVKDTAEFNRQLIKEKQSKGECACDSEHMKKCPHNRPNINTKSDSGDLAALLKNNDFGGSKRNKLVAKKAPHMIRKRYIADEKRARYGYYWKGQKLPTVAKKSPQNIEYDKWWHLKDAKDSEMQMK*

>7B2_Nemertea_Lineus_longissimus_c47447_g1_i1.p1

MSYIYFSACLVTLALIGGARADPAYRDILDGIYQYRNLRNDGYFDTIPAFEELEKDNEPTDAIPDDMSYYPQYGWEGDQPEEDDNDLASIGLADPYGYEEDEEAQAQEDDQQGPFQLRSTNDLRDPELLENSALWGTHYVSGGAGEGKQHLKPNGEQKNKQEVKSDAALPAYCEPPNPCPKGYSEDKNCQKDVKDTAEFNRQLIKEKQSKGECACDSEHMKKCPHNRPNINTKSDSGDLAALLKNNDFGDISSLLPQGSKRNKLVAKKAPHMIRKRYIADEKRARYGYYWKGQKLPTVAKKSPQNIEYDKWWHLKDAKDSEMQMK*

>7B2_Nemertea_Cerebratulus_spec_TRINITY_DN37114_c0_g1_i2_m.107252

MSYAYVCVCVVTLALLSGARADPNYRDLLDDIYQYRTLRSDGYFDNIPAFEDLQKDNEASDAIPDDMSYYPNGWDGGDEVQDNGLASLGLSDPYGYEDAEAEAEAEAEAEAEAAAEQGYDGPQDPLQLRSTNDLRDPELLENSALWGTHYVSGGAGEGKQHLKPNGEQKNKQEVKSDASLPAYCEPPNPCPKGYTKDKNCQMDVKDSAEYNRKLIMKKQSRGECACDSEHMKSCPKNRPNINTKSDGSDLEALLKDKDLEGSKRNTLVAKKSPHMIRKRYIADRQKRFGNYMKGQKLPTVAKKSPKDVEFDRWWHLKNMKDSEMQMK

>7B2_Nemertea_Cerebratulus_spec_TRINITY_DN37114_c0_g1_i1_m.107245

MSYAYVCVCVVTLALLSGARADPNYRDLLDDIYQYRTLRSDGYFDNIPAFEDLQKDNEASDAIPDDMSYYPNGWDGGDEVQDNGLASLGLSDPYGYEDAEAEAEAEAEAEAEAAAEQGYDGPQDPLQLRSTNDLRDPELLENSALWGTHYVSGGAGEGKQHLKPNGEQKNKQEVKSDASLPAYCEPPNPCPKGYTKDKNCQMDVKDSAEYNRKLIMKKQSRGECACDSEHMKSCPKNRPNINTKSDGSDLEALLKDKDLEDISSLLPQGSKRNTLVAKKSPHMIRKRYIADRQKRFGNYMKGQKLPTVAKKSPKDVEFDRWWHLKNMKDSEMQMK

>7B2_Nemertea_Notospermus_geniculatus_g12222.t1

MATLMNALSKKDKVIVMRYTSLSVCVVTLALLSGTRAADPAYRDILDGIYQYRNLRSDGYFNTIPAFEDVQDNEPTDAIPDDMSYYPQYWEDAEQADDGDVQDNDLASIGLGDPYGYDDAQDDEQDAYEPQDPLQLRSTSDLRDPELLENSALWGTHYVSGGAGEGKQHLKPNGEQKNKQEVKSDAALPAYCEPPNPCPKGYSEDKNCQKSVKDTAEYNRKLIMKKQSRGECACDGEHMKKCPKNRPNINTKSNSGEDLADLLKNKDFGGAKRDTLVAKKSPHMIRKRYVADDKRTRYGNYFKGERLPTVAKKSPVIDTEYDKWWHLKNIKENEMQMK

>7B2_Nemertea_Lineus_lacteus_comp23616_c0_seq2.p1

MSYISFSACLVSLALLGGARADPAYRDILDGIYQYRNLRNDGYFDTIPAFEELEKSNEPTDAIPDDMSYYPQYGWEGDEPQDQDNDLASLGLTDPYGYEEDEEAEAAAEDQQGPFQLRSTSDLRDPELLENSALWGTHYVSGGAGEGKQHLKPNGEQKNKQEVKSDAALPAYCEPPNPCPKGYSEDKNCRKDVKDTAEYNRQLIREKQAKGECACDSEHMKNCPHNRPNINTKSDGGDLAALLKNSDFGGSKRDTLVAKKAPHMIRKRYIADEKRARYGYYWKGQKLPTVAKKSPENMEYDKWWHLKDAKDSEMQMK*

>7B2_Nemertea_Lineus_lacteus_comp23616_c0_seq1.p1

MSYISFSACLVSLALLGGARADPAYRDILDGIYQYRNLRNDGYFDTIPAFEELEKSNEPTDAIPDDMSYYPQYGWEGDEPQDQDNDLASLGLTDPYGYEEDEEAEAAAEDQQGPFQLRSTSDLRDPELLENSALWGTHYVSGGAGEGKQHLKPNGEQKNKQEVKSDAALPAYCEPPNPCPKGYSEDKNCRKDVKDTAEYNRQLIREKQAKGECACDSEHMKNCPHNRPNINTKSDGGDLAALLKNSDFGDISSLLPQGSKRDTLVAKKAPHMIRKRYIADEKRARYGYYWKGQKLPTVAKKSPENMEYDKWWHLKDAKDSEMQMK*

>7B2_Nemertea_Lineus_ruber_Lvir.rna.tri.12543.1.p1

MIYFSVCVVSLALLGGVRADPAYRDILDGIYQYRNLRNDGYFDTIPAFEELQKNEANEPTDAIPDDMSYYPQYGWEGDRPEELDNDLASIGLGDPYGYEQDEDEAEAEEENQQGPFQLRSTSDLRDPELLENSALWGTHYVSGGAGEGKQHLKPNGEQKNKQEVKSDAALPAYCEPPNPCPKGYSEDKNCQKGVKDTAEYNRQLIKDKQAKGECACDSEHMKKCPHNRPNINTKSDSGDLAALLKNNDFGDISSLLPQGAKRNTLVAKKSPHMIKKRYIADEKRARYGFYMKGSKLPTVAKKSPENMEYDKWWHLKNTKDSEMQMK*

>7B2_Nemertea_Cerebratulus_marginatus_comp724625_c0_seq1.p1

WRNSLPYVFEDLQKDNEPSDAIPDDMSYYPYGWDEADQEEPQDNDLASVGLGVPYGYDDAEAEAEAEAEAEAEAQQAYDGPQDPLQLRSTNDLRDPELLENSALWGTHYVSGGAGEGKQHLKPNGEQKNKQEVKSDASLPAYCEPPNPCPKGYTKDKNCQTDVKDSAEYNRKLIMKKQSRGECACDSEHMKSCPKNRPNINTKSDGSDLEALLKNKDLGGSK

>7B2_Nemertea_Riseriellus_occultus_TRINITY_DN48035_c1_g1_i1.p1

QYRNLRSDGYFDTIPAFEELENNNEPTDAIPDDMSYYPQYGWEGDQPEEQGNDLASLGLADPYGYEQDDEEEEAQADQQGPFQLRSTSDLRDPELLENSALWGTHYVSGGAGEGKQHLKPNGEQKNKQEVKSDAALPAYCEPPNPCPKGYSEDKNCQKDVKDTAEYNRKLIKGKQAKGECACDSEHMKNCPHNRPNINTKSDSGDLAALLKNNDFGGSKRNTLVAKKAPHMIKKRYIADEKRARYGHYLKGQKLPIVAKKSPQNMEYDKWWHLKDDKDNEMQMK*

>7B2_Nemertea_Riseriellus_occultus_TRINITY_DN48035_c1_g1_i3.p1

QYRNLRSDGYFDTIPAFEELENNNEPTDAIPDDMSYYPQYGWEGDQPEEQGNDLASLGLADPYGYEQDDEEEEAQADQQGPFQLRSTSDLRDPELLENSALWGTHYVSGGAGEGKQHLKPNGEQKNKQEVKSDAALPAYCEPPNPCPKGYSEDKNCQKDVKDTAEYNRKLIKGKQAKGECACDSEHMKNCPHNRPNINTKSDSGDLAALLKNNDFGDISSLLPQGSKRNTLVAKKAPHMIKKRYIADEKRARYGHYLKGQKLPIVAKKSPQNMEYDKWWHLKDDKDNEMQMK*

## Glycoprotein hormone alpha

>GlyHoA_Phoronida_Phoronis_australis_TRINITY_DN212935_c0_g4_i1.p1

MNISKESLQLMKFLCVLVHLINIVRSADVWDAPGCHLVGHRQKIAVHGCVSFWATTNACRGYCVSYSVPTGQLLLARNPSQLYTSRSSCCNIVETHEVSYRARCIGGMRTFKFRSAAKCECLYCNTGDVVIYDLKK

>GlyHoA_Phoronida_Phoronis_ijimai_(P._vancouverensis)_comp74495_c0_seq1.p1

MKMNISKESLQLMKFLCLLVQLINIVRSADIWEAPGCHLVGHKQEIRVHGCVAFFARTNACRGYCVSYSIPTTKYQLEKTPRQVFTSRSACCNIVETHEVSYNVRCIGGYRTFTFRSAAKCDCLYCNKGDVVIYDLKK

>GlyHoA_Phoronida_Phoronis_psammophila_comp434876_c0_seq1.p1

MNFPKDLLQAMKIICYIVLLTLGGHCSEIWERPGCHLVGHTQKIMVHGCVAFWARTNACRGYCESYSVPSNKFQLENNPQQIYTSRS

>GlyHoA_Brachiopoda_Lingula_anatina_comp148683_c3_seq3.p1

MQPFRISDYTHVFKTVFLLLTLTLSAYSQDYKRPGCHIVSQIRKVKPKDCVGFEVRINACVGYCTSYAFPSPQRTLEANPNHIITSKSSCCNIVESHDIKVHVRCVGEQRTFVFKSAVRCSCSFCKKG

>GlyHoA_Brachiopoda_Novocrania_anomala_Nano.rna.tri2.12493.1.p1

MRLPSLAQCSHVAKVMLVLFTSFLTVLGTQSYRKPGCFITAHTREIKIEGCVSFWVKTNACIGFCTSYAYPSPQDTLLKNPHHVITSRATCCSITQTHDVPVTVDSCIGGPRKFIFKSALRCSCSVCQKG

>GlyHoA_Brachiopoda_Glottidia_pyramidata_comp32031_c0_seq3.p1

HYGRAHVFKTVFLLFTLIMSTYSQDYRKVGCYKMAHKKMVKPDGCVGFEVQTNACVGYCTSYAFPSPQRTLAANPNHIITSKSSCCNIMETHDVEVQDVWCIGGKRKFVFKSAESCSCSFCKKEKST

>GlyHoA_Brachiopoda_Glottidia_pyramidata_comp32031_c0_seq1.p1

MQPFRISDYTHVFKTVFLLFTLIMSTYSQDYRKVGCYKMAHKKMVKPDGCVGFEVQTNACVGYCTSYAFPSPQRTLAANPNHIITSKSSCCNIMETHDVEVQDVWCIGGKRKFVFKSAESCSCSFCKKEKST

>GlyHoA_Brachiopoda_Laqueus_californicus_comp38200_c0_seq1.p1

MNITMKMSTLILAVIIYGCLSAMVSSAKSLEVKKKLGCHRLGARVPIRIPGCVSFSINLNICGGYCTSYAIPSSYDTLVKNPKHLYTSKAQCCSIVDTEDVHVNVRCRFGMRSFVFKSAKVGGCACANCKKG

>GlyHoA_Brachiopoda_Terebratalia_transversa_Ttra.rna.tri.17008.1.p2

MNITMKISIFLMTIITFGCLATMASSANFLVNQVPKQEGCHALGAKNIPVTIPGCVSFVVNLNICAGYCKSYAFPSTSNTLRHNPLHVYTSKAQCCSMIESVDVHVKYVRCVTGKRNFVFKSAKRCGCANCKKG

>GlyHoA_Brachiopoda_Hemithris_psittacea_comp16488_c0_seq1.p1

MFHRKGNTSMFVKLLLVAAVLICHVATSTKIAEKLHYSKGPHRIGSTRLVKIPDCVGFYVRLNICGCYCTSYAIPSPMKTLVKNSNHIITSKAECCNMVDTEDIKVRVKCMGGYKTFIFKSAKKNGCVCSKCKTG

>GlyHoA_Nemertea_Lineus_ruber_Lvir.rna.tri.57000.1_m.100771

MFESTAHSTTRTGLIVTTVLVLLISPFLVMGKSPCFDLPGERQPSWCKPGCHKVGFRQPVAIPGCIKFWSETNACRGFCMSESYPSLADILHIAPKQTITTAGRCCVMKPKTIKYNVYCRGGNRTVMVKSAISCYCGSCQKY

>GlyHoA_Nemertea_Lineus_lacteus_comp19521_c0_seq1.p1

MFDCTVHSTTRTDLIVTTVLLLLISPFFVMGKNPCWDLPGYRQPSWCKPGCHKVGFRQPVEVPGCIKFWSETNACRGFCMSESYPSLDDILHIAPKQKITTAGRCCVMKPKMIKYNVYCRGGNRTVMVKSADSCYCGSCQKY*

>GlyHoA_Nemertea_Lineus_longissimus_c30082_g1_i1.p1

MLECTVHSTTRTDLIVTTVLFLLISPFFVMGKNPCWDLPEQRQPSWCKPGCHKVGFRQPVEVPGCIKFWSETNACRGFCMSESYPSLDDILHIAPKQKITTAGRCCVMKPKMIKYNVYCRGGNRTVMVKSADSCYCGSCQKY*

>GlyHoA_Nemertea_Notospermus_geniculatus_g22423.t1

MPSTSDRCLLEMMTPGSTRVDLVLMTALFVLLVCPFVAVAQGTRNPCRVLPIQRQPAWCRPGCHKVGWREVVRRPGCVPFVSETNACRGSCMSQSFPSVANILKIASRQTVTTEGRCCVMKPKIIRYNVLCRNGNQTIIMKSADECYCGSCQKY

>GlyHoA_Nemertea_Baseodiscus_unicolor_TRINITY_DN66218_c0_g1_i1.p1

MFQFLDRVVFLPIRYVPLSFTISIAALLLFVPPLQAKYPCFELPQSQQPRWCRPGCHKVGFLQQVEIPGCVKFTIETNACRGFCTSESYPSTHNILYMAPKQVITTAGKCCVMKPRTVRYEV

## Glycoprotein hormone beta

>GlyHoB_Phoronida_Phoronis_australis_TRINITY_DN229787_c0_g1_i1.p1

MENSVKIYYGACLILLASCAVLISARGIDTTLSCLVRDYSYNESTTHCWDFVATKACWGRCDTSEYAYYKLPYKIWNHQVCNYDSHVIKTVHLTKCHPGFRPEPKDIIFATSCSCKKCDEKNTHCTTN

>GlyHoB_Phoronida_Phoronis_ijimai_(P._vancouverensis)_comp60021_c0_seq1.p1

MENTIKICSGVCLVLLATCTVLISGSEDIATTLSCHVRDFSYNDSTTHCWDFVRTKACWGRCDTSEYAYYKLPYKIWNHQVCTYDSHVTKTIHLTKCHPGFRPEPKDIIFATSCSCKKCDEKDTHCITN

>GlyHoB_Phoronida_Phoronis_psammophila_comp64826_c0_seq1.p1

MGSEINVIPACVTTLLAICCVTSVYGNINAANTLQCHVRDYTYNETTDHCWDIVRTKACWGRCDTSEYADYRLPYKVWNHRVCNYGSHVKKTIHLTKCQPGFAPEPKDIIQATSCDCQKCNSANTHCTTN

>GlyHoB_Brachiopoda_Terebratalia_transversa_Ttra.rna.tri.15989.1.p1

MEYFSLSSHFLLLVMSLIISLVTCIDEDNVFECLRREYTKKINKPVRVGDEVLECSGNVTTLVCWGRCLSAEVGRAEYPHKEIYHPVCTYAGRTHFRFKLQKCHPNHPDPYVNVFNATDCACKACKSTDTSCESMALS

>GlyHoB_Brachiopoda_Hemithris_psittacea_comp4586_c0_seq1.p1

MYYHVTCTVYVLCLLYVISGQEIVLNAQSQENAIDADAVINACYRREYTFRVDKPHTTPSGNILECYDDVTVWSCWGRCLSSEEGHYEIPYKESEHRVCTYSGLVAFKHKLKNCHPEHPEPTVELFHATACSCTVCRSENTSCENLL

>GlyHoB_Brachiopoda_Glottidia_pyramidata_comp46211_c0_seq1.p1

MELPTALISLFPILWTIASARIDPKTMVECMPREYKHYVEKPPYIAPDGTHIPCKGYVTVMACYGRCKTSEIGDFQFPYKISYHTVCTYSDNIVKRLERLNCPGYPDPTVEVFDVERCECKICRYDSTSCENL

>GlyHoB_Brachiopoda_Lingula_anatina_comp135006_c0_seq2.p1

MELPAALISILPILWTLASGSVDPKTMLACNQREFRYNVEKPPYVAPDGTTITCGGTVKVWACYGRCETSEVGDYEFPFKLSFHNVCTYGGDVVRRRVTLDCPGYPDPTVELFDVERCECQVCRYDKTSCENI

>GlyHoB_Brachiopoda_Lingula_anatina_comp141390_c2_seq2.p1

MISFHCLLVAVLVLCVPMKTVIGCNPGNVLSCLETEYTYTINKPYQDPDTGEELHCYDEVTVYACCGRCQSYEYGEIGTPFKISEHYVCTYKTKTPRTVTLTHCHPDHPDPTAEVFDATCECRKCSSDDTSCENS

>GlyHoB_Brachiopoda_Novocrania_anomala_Nano.rna.tri2.11603.1.p1

MDWRLYPTVLLISLYILENHAIDPETTIQCLKRSYKQNIVKRSYMTESGEFLECRGWAVVDSCWGRCESYEIPDYKLPYKSSQHSVCTYSGFRKRLVNLQDCDPGYPDSTYTVFDAISCICKPCKSDDKTSCENN

>GlyHoB_Nemertea_Lineus_ruber_Lvir.rna.tri.42912.1_m.80470

MMKTHFLVSCLMFIIGVCLGDYVSNVTPCTVKKWTIRVEKPPFQTPYGRMLQCKGDVVVRGCRGLCETFERGTYEPPYLYRHHVQCTYNRITFRDVELSECDEGYPQRTYRVFEAAECGCKPCTSGHNCVGLING

>GlyHoB_Nemertea_Lineus_lacteus_comp31490_c0_seq1_m.251803

MMKTHFLVLWLMVLISGCLGVYESNATPCTVKNWKIRVEKPLFETPSGRFLQCKGDVVVRGCRGLCESFERGTYQPPYLYRHHVQCTYHRITYRDVELSECDEGYPQRTYRVFEAAECGCRRCTSGNSNCVGLING

>GlyHoB_Nemertea_Lineus_longissimus_c37754_g1_i1_m.44198

MMKTHFLVLWLMVLISDGLGGYESNVTPCTVKNWKIRVEKPPFQTPSGRLLQCKGDVVVRGCRGLCESFERGTYQPPYLYRHHVQCTYQRIAFRDVELSECDEGYPQRTYRVFEAAECGCRRCSSGNSNCVGLING

>GlyHoB_Nemertea_Notospermus_geniculatus_scaffold1019.g22422.t1.p1

MMKTNFFVIYLMVLIGICLGNPDQSPCEVRNWEMRIEKPPIQTSSGRVLRCRGDVIVRGCKGLCQTYERGSYRPPFFARHHVQCSYAGKVYREIELNDCDEGYPDRRYRVFDAEDCVCRRCYSSTTNCVGLLSG*

>GlyHoB_Nemertea_Cerebratulus_spec_TRINITY_DN962_c0_g1_i1_m.166300

GKLLRCRGDVVVSGCRGLCETFVRGTYEPPYLTRHHVQCTYQRIVWRNVELNQCDEGYPRPTHRVFEATQCGCRQCHSSNSNCVGLIH

## Bursicon alpha

>Bursicon alpha_Phoronida_Phoronis_australis_TRINITY_DN263387_c0_g1_i1.p1

MTCHSFMPQTTTSRGHRGRCLRDTVHLLLFLALLTQLCQSSRTRVCRTRRTLMLLTMRDCAEKRLLSRGCEGHCTSYLQVEFTQEGYRLHKSCHCCVPKTYREVQVPVNCLGGQRVVSFSVPTECICRPCFADVPTSEIVPSELSRNDDGLRFQGDEPATSEDVEEDVIFL

>Bursicon alpha_Phoronida_Phoronis_psammophila_comp58011_c0_seq1.p1

MPNLRTVTKVLVFITMVTHIAHCTRTEQCVRQRIIMVLRERDCRPKRLLMHACVGSCQSYSQVTFTRNGFRIQRSCKCCVPINHREVRTRIMCRGPEGGRVYQIKNFSVPSECMCRPCYPDVPASEIVPQELANYNNELNLQGDEPDSNPWRDEVLFI

>Bursicon alpha_Phoronida_Phoronis_ijimai_(P._vancouverensis)_comp96428_c0_seq7.p1

QLSYTFKADLPGEGLAYIQLSNECTEALKSCSCAAGSKAEENKCLRLEENCKRCHCNNTNSMISESKEGEHCDFVDSCKTCECVWSNYTYIWDNCKRLPNCQTTTVATTTRLTTILSTTTSRPTTTSSTTRTTSKPTTETTVASSTTQPTTTRSTTQSTTRRPTTSPTSRPTTTAPERCTDPKPALFNPAVKVSVDASSYMNVSYTPNAANMDNPFGWLAEPADHYPYLLVEFRVAITVYGILLKGRNMANLTMEYRNNASEPWTEHDFELTVVNGEVMRVIFSNQFAAKYVRFSPTKEVAFLPVGFNLDVLGCFDLKCEETGRITVKGPCVKTCEDIARGNTECPSINEDDHEGCQCPFGQVVYNGKCIEPKECPCYYEGKWYKAKEVFDDPNNCREGICLGDKLKFDSYENVTECEEQKNATCRPEEQTRIVEVENCKSSEEITYNVCGGSCSSKAHLTFGLAMFNMDCTCCKPTQFSMKTVQLTCADGSTIQRDYPHIEKCGCQACDYYPSLASTTVQAFPVTL*

>Bursicon alpha_Nemertea_Lineus_ruber_Lvir.rna.tri.90334.1.p1

MKICLVILVLNCMLLEITSGQELLTATRSQCIRRPIGMKLQYPNCQKKGVLSFVCQGRCPSYAQIFSSRIDAVEHSCKCCQE

>Bursicon alpha_Nemertea_Cerebratulus_marginatus_comp2154246_c0_seq1.p1

MKIYLVILVLNCIFLQKAYGQELVSGMRTRCIRRPIVMKLIYPNCAPKGVLSFVCQGRCASYAQIFSSRIDAIEHSCKCCQEVTEAERRVPLRCLDPNNHGVMRRVVAKFI

>Bursicon alpha_Nemertea_Tubulanus_polymorphus_comp299301_c0_seq1.p1

MRILCAIILCTLFTYVHTSPASDVSIPVPAACQRQRILLRIAKENCETKRILSFACRGTCPSYSQLTPDGLGMERSCKCCKEIRDVTRRVKLICRNPTTSHLHYIILEVKVPGR

>Bursicon alpha_Nemertea_Lineus_longissimus_c37465_g1_i1_m.42890

MKIVLGILVLNVILLAFTSGQELLTTRSRCIRRPIGMKLQYPNCQKKGVLSYVCQGRCPSYAQIFSSRIDAVEHSCKCCQEVMEVERRVPLLCLNPNNPGVRRRIVAKFMVPKNCMCRPCSAGIGASFLDPIDYTAKRAPVGWFGRIKR

>Bursicon alpha_Nemertea_Lineus_longissimus_c36337_g1_i1_m.37640

MESSPARCYGKIQILCLSLLWLVILTTPSNGQISKRDVALCEKTAMNLILHPPPMIADRCPKTIVPTIGCRGHCNSYSKVDMAYPFEITRSCSCCTPTSFAIDLVIMRCKDGQNIRRPFRYARDCICRPCDNHDKYNAYAEVGLEKRAGHVWSNHEQVIAEVKRSGGGESNLFGDISGDGSLAS

>Bursicon alpha_Nemertea_Notospermus_geniculatus_scaffold127.g5913.t1.p1

MASSASPNTGKMPVLQIFGFLLITLLTPSLGEPLIRNIPTCEKTAMNLILCCTPTSFAIHVVIMSCKDGQKFRRPFRYARDCICRPCDNHNRYNVHGNSEFEKRGEKNWSEPMSLEGRRANRADEDWYSLEDIALEGPLDKTIGTGRDVEE*

>Bursicon alpha_Nemertea_Notospermus_geniculatus_scaffold1172.g24107.t1.p1

MKICVVIVVLYSIIMHMVAGQELVSAARSRCVRRPIGMKLKYPNCAPRGVLSFVCQGRCHSYAQILSSRTDAIEHSCRCCQEVREVERRVALRCLNPNQPGVIRRIFVKFMVPQTCMCRPCSAGVGASVLDSIDYTAKRAPVGWFGRIKR*

>Bursicon alpha_Nemertea_Cerebratulus_spec_TRINITY_DN49340_c0_g1_i1.p2

KVDMDDPFEMRRSCSCCTPTSFAIHVVVMRCKDGQKFRRPFRYARDCICRPCDNHDKYNGHMDGLEKRGDTGWEEKRGDSGLRVEEALRSLEK

>Bursicon alpha_Nemertea_Nipponemertes_spec_TRINITY_DN27032_c0_g2_i1_m.69198

IGEASSNTAVGQKSRRSTVLAPGTSAQIDRHCKCCQQASHEDRRVPLLCRDSNRRIAMAMTTIRFPTSCMCRPCSSEEASVEPLEMTDYLVKKRRGFDEESVEDVRFGEPHDNKRERFASWEEQDSVEDSYPDL

## Bursicon beta

>Bursicon beta_Phoronida_Phoronis_psammophila_comp70449_c0_seq8.p1

MDGFTLNQVRLVTLAFSVSILPHLIQSRLTPRDDCRTVTVSLNLEQDRTVAQDDGVELQITCRGHVDVKKCEGTCVSRAVPSAMMFPRFKQDCKCCRESHFQNKVVTLGECHHGNQVKRDLAITVNFREPTDCTCVPCTLII

>Bursicon beta_Phoronida_Phoronis_australis_TRINITY_DN287144_c1_g1_i1.p1

MTDLFKIPIILLLVSHVVHSRYSTSDDCRSEIETLNMEQERTVIQENGISIHITCSGSVDVTKCEGTCVSRAKPSAKRYRRFEKNCRCCAESRLEDKVATLGNCHHGNQQRRDLAITVHYKQPADCRCAHCSM

>Bursicon beta_Phoronida_Phoronis_ijimai_(P._vancouverensis)_comp488768_c0_seq1.p2

SGSVDVTKCEGTCVSQATPTTEGYARFKKSCKCCTESRLEDKVVTLGDCYHGSQQRRDLAITVHYKQPDGCACTQCSM

>Bursicon beta_Nemertea_Paranemertes_peregrina_comp631478_c0_seq1.p1

MMMIAYVLVCLVPLAAYIGTTTASECHTIETDLVLSKEKSVEYGGRTRNLLCFGTVKVNKCEGMCDSQAQPSVVQR

>Bursicon beta_Nemertea_Paranemertes_peregrina_comp11152_c0_seq1_m.6955

GMCDSQVQPSVVQRTGFKKVCQCCSEGGFKNRLVRLSTCMDGSRQVRDQQAVFVLKEPSDCQCRDCAI

>Bursicon beta_Nemertea_Cerebratulus_spec_TRINITY_DN38774_c2_g1_i1_m.75983

MNARKLIQLILSVATLLLATMIITSQAAMDNDDFCETLDTELYVTKEKTYDYNGRTKRLICGGNVRVTKCEGLCKSHVQPSVVHYPGFKKSCQCCREGTLRNRLVTLNDCFDGSIRIIGQSVHFVIKEPADCACYDCAI

>Bursicon beta_Nemertea_Tubulanus_polymorphus_comp21224_c0_seq1.p1

MTCSTAVTVTSCIYMLLIITPGLMAIFDDRSLCETLETETVIPRETTYEYNGRIVRLLCMGKVKVKKCEGLCVSQVWPSVQKYPGFKQECRCCREGRLQSRVVTLTDCYNGSKQQVHGQSISVLIREPEDCSCFDCSMA*

>Bursicon beta_Nemertea_Lineus_ruber_Lvir.rna.tri.71322.1.p1

MKMAKLTQLILSVVTLLLATLIIASQAEVDDDDLCETLETELYVTKEKTYDYNGRTRRLLCGGTILVTKCEGLCKSHVQPSVVHYPGFKKSCQCCREGTLRSKLVTLHDCFDGSKRISGESANFLIKEPAECACYDCAI*

>Bursicon beta_Nemertea_Notospermus_geniculatus_scaffold1172.g24108.t1.p1

MTTKKLSQFLLFMVLLLLATLLISCQATLNDGDLCETLETDLHVTKEKSYDYNGRTRRLLCTGTLTVTKCEGLCTSHVQPSVVHYPGFKKSCKCCREGTLRNRLVTLHDCFDGSKRVSGQVANFIITEPAHCACHDCAI*

>Bursicon beta_Nemertea_Lineus_longissimus_c38083_g1_i1.p1

MKMAKLTQLILSMVTLLLATLIVTSQAAAGDDDLCETLETELYVTKEKTYDYNGRTRRLLCGGTIMVTKCEGLCKSHVQPSVVHYPGFKKSCQCCREGSLRSKLVTLHECFDGSKRISGQTANFLIKEPAECACYDCAI*

## Insulin-like peptide (ILP)

>Insulin-like peptide Phoronida_Phoronopsis_harmeri_c122112_g1_i1.p1

MINTRVIIMGPNVLWSVFLWIACLQFTSVYGGYERSCTLETQMEIPRGRLCSGHLTDVLRAVCGGRYYFLNKRTEERTEEQSNVEKRDINPFTRKRSHGEVFLNKRDATRFLAKTGSHSRGIVCECCYHQCRFRELRQYCMVNKRSIPEHVEAKTQSMINDIMNRYEQVTTQTPNTNRDEDSTDDIWSNSVE

>Insulin-like peptide Phoronida_Phoronis_australis_TRINITY_DN319395_c5_g3_i1.p1

MYRLVFVCVIVTNTLLTGVHAAEMYLCGATLANSLAFVCRGRGYYWTQSKRSGAYVTKRGAPENVRIRRNVHSPFLSDTEATDFLTSRDGGKTRIRRGIVDECCLSPCNFRHLESYCAAKTTIRSPATTTPRPRRTLTTAPPTTLSPPPTAPFNVYSGNDVNNESGGQLYNNHVIVKGMNEHGGSLADERSKVNATLVNSTAPVEPSTAPGNKLPPLKPVKVGQPTSISRVKFPSKMKLSKYIQKKLNKINRMRGKQKTRFQLSSKLGRYTTALPRLAKAIQTPFLHSLLKRYNQNLDWY*

>Insulin-like peptide Phoronida_Phoronis_australis_TRINITY_DN277627_c0_g2_i1.p1

MCSRVTWVITVCLASYITTARGGYERTCNLETQYEIPRGKLCSRRLPNLLRTICKGRYYGLSKRIDTTNGIEKRDSENLEVIPSLEEIFLSKRDASVFLLQRNTRNRWSGHHGATRGIVCECCYNQCNYRELHQYCKLKKRSTPNDNLSSEKVLKHILLRLESRITSQGKNVKKR

>Insulin-like peptide Phoronida_Phoronis_australis_TRINITY_DN288229_c0_g2_i5.p1

MLLTTILLSALSACVVAADRFCGLEDYNGNSRGICGPKIPQMLSVVCSEFGGYHSVNKKSYYDSAKVDSRDNHWPKDDDMFLTEKEATDYLHNPKGKRGSGNQNIVCECCVHQCSYGEMRGYCLKPKDVRRIKRSFPNAPIFKSHSPSPVFRDFLSAERNKRHVKLRGSVALGND

>Insulin-like peptide Phoronida_Phoronis_ijimai_(P._vancouverensis)_comp100160_c1_seq1.p1

MYRLVFVCVIITNTLLTGVHAADMYLCGATLANSLAFVCRGRGYYWTQSKRSGAYVAKRGAPENVRVRRNVHSPFLSDSEATDFLTSRDGGQTRIRRGIVDECCLSPCNFRHLESYCAAKTTIRSPTTTTPRPRRTRPTTALPTTTSPPPTTQPPTTQPPTAQPNVYSENDVSNESGGEFYNNDVMFNGLSADEENDENATHVNSTTPEVVTSTAPGNELPAFIPTKPARPSSISHVKFPSKMKLSKYIQKKLNRIKSMRGKQKRRFKFSSKLGRYTTALPRLAKAIQTPFLHSLLKQYNQNLGWH

>Insulin-like peptide Phoronida_Phoronis_ijimai_(P._vancouverensis)_comp86274_c0_seq1.p1

MLLTTILLSVLSACVVAADRFCGLEDYNGNSRGICGPRIPQMLSVVCSEFGGYHSINKKSYYDSVTVDSRDKLWPKDDDTFLSKKEATDYLYNPKGKRGRGNQNIVCECCVHQCSYGEMRGYCLKPKDVRRIKRSFPKAPIFKSRAPSPVFRDHLSTERNKRRVKLRDSLALGND

>Insulin-like peptide Phoronida_Phoronis_psammophila_comp68197_c0_seq5.p1

MLVATLILSALSAWEVKADRFCTADDYAGNNRGICGPRIPKMLSVLCSPHGYHSYSKRSSYYDTAPREEDELWKNQGVFLSEKKALDYLDTMSKRGTTQNIVCECCVHNCGAQEMQGYCLRPADVSRIKRSHPNAAIFKSNGPSSHLHNYLSAPRDFKRLKPAFFNR

>Insulin-like peptide Phoronida_Phoronis_psammophila_comp59791_c0_seq3.p1

MYRTLFACFFILSTLVCRSITADMYLCGATLANSLAFVCRGRGYYWTHSKRSGAYVTRRGRMDYNRFRRNVHSPFLSDTEATDFLSADEGPKVRVRRGIVDECCINPCNFRHLESYCAPKKEKST

>Insulin-like peptide Brachiopoda_Glottidia_pyramidata_comp32503_c0_seq1.p1

MSRITFIWRNLVNVAMVDSRQTVCILLCCICCFNSVFAGYTKVCSGDEPASGMCGKPLASMLEMVCGSVGYNGPNKRSLRVTRSASSGVLEELRASMFQPKEQALSYLSLHKRRTGITGTLICECCFNPCTMMELTQYCKSSKKRSLNQLTDLYHLYYRNNKNDKNILQNLGFQPAP

>Insulin-like peptide Brachiopoda_Hemithris_psittacea_comp15446_c0_seq1.p1

MLSPSVRTGHTSILRMMSHSSQVFLLVVAWSVSDIALVKSQRGNLHVCTEESKRESPVRFLCGAPLVNMVTKFCYPYGIHYVARREVMREKRAIPNIRGILLPKHSALSYLRKRRSQYGVGESNINCECCFNSCNTREMLQYCAADPFGPRFGGPRFRGPRRRPGRRNHPLSLRNPPRR

>Insulin-like peptide Brachiopoda_Hemithris_psittacea_comp22740_c0_seq1.p2

MAKLGVFCLICMVVLAGHAIALERVCGYQLADLLQLICGRRGFWTSSNEVGKRSVGTVRALPLLEKRVALSVLSTDEIHQMRRRKRGIVNECCHNACTYSILESYCGAPNADTLLGDDSYYQVQRRRSKMTSEKKEINREVIKRPKNKTLVSQKSLKGKATVVNIPTGRFIPRSNGILNLFFKQLLKRRQHN

>Insulin-like peptide Brachiopoda_Hemithris_psittacea_comp24911_c0_seq9.p1

GHYGRGYTMKVAFSLLVLGFLALSDGAWWRCTKNKYCGKHLHKKVIAVCHYRGKRAVSDAGFGVDVQDDDSNGKFFQDADEDDASDDTPGLPSNWNLDDKKSRTFLRNNKRGKLFHPSLHEECCSEGCCNEEVKEHC*

>Insulin-like peptide Brachiopoda_Laqueus_californicus_comp43243_c0_seq11.p2

MEKILAMMLCVLMFSVHVHSFCGSDLNTLRNHVCGGKWGGGKWGDEEAAIPMARREKANSFLKITKRGNLYEECCREGCSYREVKKHC

>Insulin-like peptide Brachiopoda_Laqueus_californicus_comp33843_c0_seq1.p1

PLRPGMKVVLLLAFLICTSEAWWAKKCARHKYCKKALTDVINKTCGLGDTEFMDKERAKVFLKKRVLFRGGLNEECCHETCCVEEVREHC

>Insulin-like peptide Brachiopoda_Laqueus_californicus_comp40605_c0_seq1.p1

MKAFGICSIVMALHCIGQSVAFERICGAKLGDLLFAICGQRGFNGQNIGKRSADITLMLPTLQKRNAFSFMSEDGFHSRRRRGIATECCEKGGCTYANIEQYCAAPKRSDPNMADNPSGKYSKRQKNGGIRKLALAIRLALLKPDKSGMYTRLEPGVKPMSYKTNHKVIANQKMKFRTKSKQHKASSVENISITPNSPVVPHNI

>Insulin-like peptide Brachiopoda_Laqueus_californicus_comp38962_c0_seq1.p1

MKVFVCTLLLAVVCMVYISEVSGGIKRCRWWKCTNKCNNPQYYCGAKLNAKIVQTCHHDGKRSIDVEKNNMNTIEEDKEDQQFLDETMKLLEDVPDEFKVNDDDARTFLTKRARPLYARLHKDCCKKKCCTEQVKEKC*

>Insulin-like peptide Brachiopoda_Laqueus_californicus_comp24991_c0_seq1.p1

MKLIRLTQSIIVMSVVIHNAIGATYCDRNLPRLIMKACGEAKRSAEDDHNVTFSLRHFSPKEMTYIRGILKSAEKFIVRRTLFKGIARKKRQISRSQTDMRVAIRKDSIMHHPGSRSVLPLKELIDICCQWGCTPEELARAC*

>Insulin-like peptide Brachiopoda_Laqueus_californicus_comp42185_c0_seq4.p1

MLKIPVVVVLFCACVLLSSVHSHYYCGSALQKKYKDVCHQVWKDGMARRGIPVMQHEQADSFLKMTKRLSNRNRRKLTEECCYEGCRWEEIRELCGR

>Insulin-like peptide Brachiopoda_Laqueus_californicus_comp37845_c0_seq1.p1

MLKMLVAVLFCACVLLPSIHSMNLCGEDLREKVDDLCFSTNPRMAKKGPLVVPTKRSLFRDKRKAESFLKDPCRKVKANSLQEECCSEGCKPEEIYEIC

>Insulin-like peptide Brachiopoda_Laqueus_californicus_comp42185_c0_seq5.p1

MLKIPVVVVLFCACVLLSSVHSHYYCGSALQKKYKDVCHQVWKDGMARRGIPVMQHEQADSFLKMTKRLSNRNRRKLTEECCYEGCRWEEIRELCGR

>Insulin-like peptide Brachiopoda_Laqueus_californicus_comp41493_c0_seq1.p1

MNQLYLFLLLVATWTTCCLKTVLAGEKVCTHESRREGTFGVCGVGLTDMLTTMCKGSGGFFSRRPSKRSIGDILLPRKFALAYLRKRRSTSRQSGEQGIVCECCYNKCSILEMAEYCTNPSKGSPFSIYSQKPSLVTTPSYLSSIRSVLSQRYPSFLQRLRSRIITRVNITG

>Insulin-like peptide Brachiopoda_Lingula_anatina_comp119156_c1_seq1.p1

MAIRFAIASLLAACLALVPRGAKTEKLCGKLLADTLDLVCHGRGVNFDRPTRPMAKLFQLKKTATPLLAVKRHAVHKDHVISTEDLGRLRRYGLVEECCINSNCNLSILEQFCDA

>Insulin-like peptide Brachiopoda_Lingula_anatina_comp126651_c1_seq2.p1

MSPITFLWRKIIAVAMVDSRQTVCVLLCCICCFNTVFASYSRVCTGDEPAKGMCGKSLSTLLDTVCKMYGGFQGPSKRSIRDTKPVAIGVLEELRESLFQPKERALSYFGLRKRRTGRTGTLTCECCYHSCAMTELTQYCASASRKRSLNQLTDLYHLYFGANKDDGSALQDLGFQPAL

>Insulin-like peptide Brachiopoda_Lingula_anatina_comp152136_c0_seq1.p1

MYKSYSLIIGTVIVLSLCHAALSENLCGKSLADTLDLVCFGRGFNYNPSAGKRLAPMFLSKRAALPFLISKRHDTDLASSTATNLSNPDGLQPRRKRGIVEECCKKSCSWSVLESYCASAGSAGPGSVNHNNGDQETFASDLGKLVDMLTSSLEMLHPRGQKRNNAH

>Insulin-like peptide Brachiopoda_Terebratalia_transversa_Ttra.rna.tri.31226.1.p1

MKVKFLLVGVLMICMISVSEAWWARKCIRHHLCNKDLQKAVEKTCGLGDTEMSLDKETANVFLKKRALKGGLREECCNEKCCYEEIKEHC

>Insulin-like peptide Brachiopoda_Terebratalia_transversa_Ttra.rna.tri.5859.1.p2

MNQLYLYLLLVAAWTICCVKTVLADDRVCTHESNREGARGSCGSDLTVKVAELCRDLGGTYSRPITKRSIQDILLPRKHALAYLRKRRSTTSAQIVCECCYNTCSRHEIMEYCLKPSNDDSHPSSQSPAVTTPSYPLNIRYMYMSRQRAYPSFIQRLRNRIITRVNMTGSRLFHG

>Insulin-like peptide Brachiopoda_Terebratalia_transversa_Ttra.rna.tri.21852.1.p1

MKLIQLTQSIIVMSVLAHNAIRATYCDRNLPRLIMKACGEAKRSAEDQSVTFSLRHFSPAEMRYIKGILKSADQFIVRTVFNNVARKKRETNRSETDRSVSIRKDSTIMHHPGRSVLPLKELIDICCQWGCTREELARAC*

>Insulin-like peptide Brachiopoda_Terebratalia_transversa_Ttra.rna.tri.12965.1.p1

MNAISIFSIVIALYCISQRVASESKTRVCGQDLSNLAYLICGKRGFFSVDGVGKRSADLTQVLPTIKKRNAFSFMREGGVRSRRRRGIATECCDKGCTYATIEQYCAVPKRSDPNMADNPFRIYSKRQKNDDIRKLALAIRLAQLKKDRANGDKYTRVNSGHTYTNIHNTVSEKPEYEFQNKIHTT

>Insulin-like peptide Brachiopoda_Novocrania_anomala_Nano.rna.tri2.1217.1.p2

MSLKSYLCVLTVMLLIVTTYTTYPRYCGRNLHARYALICHQADLHARCKRDVIIGKTVAKHFLSVRVKRSLHHECCTEGCDDEEIVEHC

>Insulin-like peptide Brachiopoda_Novocrania_anomala_comp19507_c0_seq1.p1

MHARLTAGAMVENRLMVIVFLWFTCCLGSSVAIISRKCSNADSQTTVKGICGSGLSDMLSMVCDPFGFSDGTMRKRNQGHPANQIDKYMLHNVFQPKDKALSYFNKRGTWEGVVCECCLNNCDLFEMEDYCLNPKKKKV

>Insulin-like peptide Brachiopoda_Novocrania_anomala_Nano.rna.tri2.44511.1.p1

MHARLTAGAMVENRLMVIVFLWFTCCLGSSVAIISRKCSNADSQTTVKGICGSGLSDMLSMVCDPFGFSDGTLRKRNQGPLANQIDKYMLQNVFQPKDKALSYFNKRGTWDGVVCECCLNNCDISEMADYCLHPMMKKRNSQARQKLIRNRIDNYKRLKGLQQLLP

>Insulin-like peptide Brachiopoda_Novocrania_anomala_Nano.rna.tri2.31125.1.p1

MRESTVVVLLVFAFLAAYCDLAQGRVRRICDRDLPRAVYETCSASKRSVASSAETRPFSALKRNKQDLFSILRIGKRDISPDMTTRELIDHLVSRGRRRVLPLYVILNNCCRYGCSDDMLRGLC*

>Insulin-like peptide Brachiopoda_Novocrania_anomala_Nano.rna.tri2.50645.1.p1

MPPKFYLCVVVLLLVMATCNSWWLGCKKHHYCGKDLDNKKAEICKRGKRGVFMKKLEANDFFETQAKRGFGLYEECCYEKCCDKEIYKKC*

>Insulin-like peptide Brachiopoda_Novocrania_anomala_comp71882_c0_seq1.p1

NYFKYERRLIATTTMPPKFYLCVVVLLLVMATCNSWWVGCKKHRYCGKDLDTKKAEICKHRGKRGVFMKKLEANDFFETQAKRGFFKSKGLGEECCGEKCCDHEIYEKC

>Insulin-like peptide Brachiopoda_Novocrania_anomala_comp76732_c0_seq1.p1

MSPKFYLCLTVLLLVVTTCNSWWWGKSPIRLGCTKTKYCGPAIVAKLKRTCNFRDSAGKRGVFKEKVTANDFFGAKVKRWTIGSLTEECCNESCCYEEIKEHC

>Insulin-like peptide Brachiopoda_Novocrania_anomala_Nano.rna.tri2.154919.1.p1

MPPKFCLCLTVLLLVMTPWVRSCRRTKICGDAQISDKLRSVCALAGKRGVFMEKLKANDFFGAKTKRGWLFHPSLTEECCNERCCFEEV

>Insulin-like peptide Brachiopoda_Novocrania_anomala_Nano.rna.tri2.46621.1.p1

MSPKFYLCLTVLLLTMTTCNSWWSRCKKYRYCGTQIYRPLQNACNPGKRDVFNEKATANDFFGAKVKRGTIGDLNQECCIDKCCYEEIKEHC

>Insulin-like peptide Brachiopoda_Novocrania_anomala_Nano.rna.tri2.14721.1.p1

MYPTTTTAFTTALLAFLSLQVQAEQLCGYQLADMLDMVCFGRGFHWHINGKRSAIPRLVKREAVLPFLSKREANDYLGGASRRREKRAAVGGIVEECCHKSCTYAILESYCAGSGSGYYSDDYTDDYQHDSYSSVTPDSKVVSTRSTHKKIIINAIVAPSPAPLPFQYELEHAHVHPLALRSNKAGTDKPEPTNVKSGKAKAEPRNVKSGKDKPAPKNLKSAPKTEHLAKVQHKVKPNLGNVLTASFDSLKPKQQHQRPVQRMPRPSRTGRLQGKIHH

>Insulin-like peptide Nemertea_Cephalothrix_hongkongiensis_comp8959_c0_seq1_m.4433

MDFIGTIFFTLICMILDQRVCCLERLCGKSLANVLNIVCDDRGFYVETKEDVIAKRDSVQFLSRHNEMDRQSRGVVDECCHNHCTYEILESYCASPPNYDYDYRSGLESLLNGKTDSISVVSENKNEVQNLFTVVPEIQVERFGDRSRDNYFFIGEKLTTSSISGSKREQFLKTKNLMKKHFKNRLE

>Insulin-like peptide Nemertea_Malacobdella_grossa_comp26248_c0_seq1_m.48673

MVLNFGKSNSILFMMPAVGMDRQKKRNCRSGKTRTWFPLLAVWLVLLQTRSVEAGYEHVCTTDDYNGNAMGRCGRQLRILMDMICVQGTYHELPQKREEGGLRLPAHKGSSAKRGPSPNADNNNDDDYDVTDIRKKETGKLMTKSPYYLRRPYSRYLTKQTDTEISRKALERPRKDRRIDPIFLSNEKAKSFLYSRRSLRTRRQLMIPSTRTGIVCECCVHSCGVSEIKEYCSQPLL

>Insulin-like peptide Nemertea_Baseodiscus_unicolor_TRINITY_DN62108_c0_g1_i1_m.232666

VSRVCLAVTVFTTSEASKFCGRALPRAMFIACGIAKRSAPPESALPSDLESITGSLRFRRSEEIVDNAHSMFNENERSKRGVIPLNVLADVCCRRGCSLQDLAGAC

>Insulin-like peptide Nemertea_Notospermus_geniculatus_g27942.t1

MIYQKFFILLVSLALILTFASQTDAILLDPDVPQKLCSKEIHRAFQMSCLVSKRRKRSTDADSEYFVRKARFSQFRDCCYRQCTLRQLASFC

>Insulin-like peptide Nemertea_Notospermus_geniculatus_scaffold2346.g32744.t1.p1

MGYIIYQKSLIFLVSLVLILTFTSQTDARNEDPDAAIQKCGKALIRAYQSSCQYNPQGNPPPPNEYQRRRQELLERCCRSQCTLRELAVFCQNDRRR*

>Insulin-like peptide Nemertea_Notospermus_geniculatus_scaffold213.g8565.t1.p1

MISPIIYQKPLTLLVSLMFIQTSTATPPDPYAPIQNLCNLDLTRELQRSYLVWGRVNAESDVCKYERRRADLFQRCCDWQCTLAELAYFNVHDGRVPRDDPSNSTCARHALG*

>Insulin-like peptide Nemertea_Notospermus_geniculatus_g9929.t1

MAKYTIQLATVLVLFLTFSALVRANGSRFCGRDLPRAMFIACGIAKRGAPSFDAEPDLDASSLLKGSLRYRRSAVIADAMHSAFNEHERSKRGVVPLNVLADVCCKAGCSLRDLAGAC

>Insulin-like peptide Nemertea_Notospermus_geniculatus_g2033.t1

MIGRDYKCTAAMAKTTPVALTALLLFFAALPFTKADGWPKTCTEDDVERGPVPGAVCGPRLANLLGTICGEDGYYAGDSFLKKRSGPNTHSDHNIPFISNEKAKTYLKSKRWGSDGWTGIVCECCYNRCGYTELQQYCNQPSKRGASLAKKSPTSVRSRLPETAIKNPARIVVDRWLSFGRQILDILTGEQHKSTTDGTHND

>Insulin-like peptide Nemertea_Notospermus_geniculatus_g17901.t1

MNFRRVLFIVVFKISLAYGLERLCGRTLADVLGFICQGRGFHGESSVSDDQKRSFRNQMITRRQATTFLVGSTVPPSLNHRSGVVEECCLRHCTYETLESYCAEPSATGDEYDLEEFLNPSSSSTSSTTSTTRRQSTSTAGPPTTKQYRRLPSFMQDRTFFYVRNDENQSEEQDQDQDQDEDQGQEQDKKKKREHGKTRQILKKAFGVSKQNAEPSTHSG

>Insulin-like peptide Nemertea_Notospermus_geniculatus_scaffold196.g8118.t1.p1

MTARRYHTSKGYFLRTTLPKRPEAAREGKGTNAGPRSLLLLNAALFITVSCLVHCTDARRFDHMLADFLLRSDEQWRQAWHTDCHRRCRHELDSQIRLACAKDIYRIKRSSNDNATTFNLPPTSKSPRSLLLLNAALFITVSCLVHCTDARRFDHMLADFLLRSDEQWRQAWHTDCHRRCRHELDSQIRLACAKDIYRIKRSSNDNATTFNLPPTSKNPILDRKTAKAFLHKLNRHPIRMKRGIMEECCYSKSCTWEEYAESCHHYGRSDAEDSNTCVPQ*

>Insulin-like peptide Nemertea_Notospermus_geniculatus_scaffold423.g13517.t1.p1

LLSLKRPPPIISLTNFVPRREVWSYDGTPGLTCNPNKKIWPNIPFNLPQYWSSSLHSAPSLLKGSLRYRRSAVIADAMHSAFNEHERSKRGVVPLNVLADVCCKAGCSLRDLAGAC*

>Insulin-like peptide Nemertea_Notospermus_geniculatus_scaffold1225.g24702.t1.p1

MGYTDALNEDPDAPIQKCGKALIRAYQSSCQYSPQGNPPPPSEYQRRRRELLERCCRSQCTLRELSVFCQNDRRR*

>Insulin-like peptide Nemertea_Notospermus_geniculatus_scaffold235.g9220.t1.p1

MNIAVIKGEQIRYQQCHVMVLTLLFVGHFIRPTVQYRGCGPRLANAVELVCEMRPGINNEGPGSEENDDARQIVSLVADQARLKKSSPFVSKRTANEFLSIRSRRGIVDECCRHDCKIEELEEYCPPVGGNMADKVPVVKATREANRQSEPSNAKDEVSTPREEETPAEQEQKTVGKSATAPKSNRAPELGGKVSVVTETVRKESFSQEETFTAVPAVKEVHVPPPAEPLIQSEGSSTVQKVERIQEGLTSSRDRPLNEPSLVVQRMVPPSVEQFGSGSHFTSKSVKMSNVVQAPPPDANTYKVHRLPPKHRTGASSRLTPFPYKVLESINRSRH*

>Insulin-like peptide Nemertea_Lineus_longissimus_c35163_g1_i1.p1

MNFAASGGAMIRRHEYNLIVLVFLISHFICPTVQYRGCGPHLANAVELVCEMRPGVNNEDPDDQTDDATQLVSLLTEQSRNKKSSPFVSKRTANGFLRVRSRRGIVDECCNRDCHIEELEEYCPPVVGKIPRGIEDEQQQVTAKEEEPVVEESATINGNEASLRDQETNGNVIPEKETDREVESNMALDINANEAPSVDPVQNEISETYLGVDLIPTAEPVQKETLSVEEELKDTASGQARQNIDPTVKSVLPQQIMKNVKQVAPYIGQYKPDAHAGSAAPSDATQAYKIQTVRVPPIPKVGSSRRPFPHKVLESINRWRG*

>Insulin-like peptide Nemertea_Lineus_longissimus_c37994_g1_i1_m.45130

MIYQKLLVLLSFLVLIIHFNAQTDAILLDPDVPQRLCSKDIHRAFQMSCLVSKRRKRSIDSDSEYFVRKARFSQFRECCFRECTLRQLSSLC

>Insulin-like peptide Nemertea_Lineus_longissimus_c28306_g1_i1_m.18138

MIGSDYELSAATMAKTTPIALTALLLISAALPIARANGWAKTCTEDDVERGPVGVCGPHLSSMLGTICVNGYYAGDAFIKKRSADTSEAEETEERIVPFISTDRAKTYLKSKRWGGEGWSGIVCECCYNRCTYTELQQYCNQPSSKRSASPSSVKNSVLYSNPQLAIKNPARVLNKHWLALGKRIIGMLTGSSRTGTKGPQNN

>Insulin-like peptide Nemertea_Lineus_longissimus_c35795_g1_i1_m.35566

MMNSVISKMAENILVAIPMFMLVFLLTNDKSTADDGHMKVCTEDIMERGPVDGGQCGHQLANLVDNICYPHGVYYAAGDLFMMKRLSKNGGISALSYNPFMPSNKANSYLKEKRMAQTGVSGIVCECCYHKCSHQEFHQYCNLPAKRGYGNDAMDSTLARVSRIRNLPSIKGNGHHGVTNDKAGRIPKSYRAKDDRLLARRIFRLLRGDIEERKQFSRQVIKSIE

>Insulin-like peptide Nemertea_Lineus_longissimus_c30727_g1_i2.p1

MVLRHFTVDNRRPILLARYYSYFKLFSIANFRAT

MTQSIPILLTSLLLLIDQLTSTQASGHTKTCTQEDILRGPARGALCGPHLANILSVICPNGYHTFKTDTEFTTRHYVPFISVHRAKSSVRSKKWDSSWTGIVCECCYNRCFFGQLLEYCHTSNRIPSYTKSSDSSNSARSQGSKWLALGKRILHTFSRDSRPSTK*

>Insulin-like peptide Nemertea_Lineus_longissimus_c48195_g1_i1.p1

MSARIWSGYPKQKGTTKRKEVISSGCAT

MLKVRSSHGSLILYAALFIVITCLVHCTEAERFDHMLADFLLRSEDQWRQAWHTDCHRRCRHELDSQIRLACAKDIYRIRRAADVDASNVTLPPIPKNQIFDRKTATAFLHKLHRQPIRMKRGIMEECCYAKSCSWEEYAESCHHYGRSDADSSNICGPWD*

>Insulin-like peptide Nemertea_Lineus_ruber_Lvir.rna.tri.36683.1_m.70451

MIYQKLLVLLSFLVLIIHMKAQADAILLDPDVPQKLCSKDIHRAFQMSCLVSKRRKRSIDSDPDYFVRKARFSQFRDCCFRECTLRQLAAYC

>Insulin-like peptide Nemertea_Lineus_ruber_Lvir.rna.tri.48995.1_m.89721

MAKYSLHIAITLVLFLTTSVIIGSQASQFCGKDLPRAMFLACGIAKRSAPSTEAESVPDIGSLVKEHMRFRRSTSDIADVAHSVFNENSRSKRGVIPLNVLADVCCKAGCSLRDLAGAC

>Insulin-like peptide Nemertea_Lineus_ruber_Lvir.rna.tri.54265.1.p1

MISRDYELVAATMAKTTPIALIALLLLCAALPFTQANGWAKTCTEDDVARGPAPGAVCGPRLSNLLRTICGPNGYYAGEAFIKKRSAPTSEAEESNEHMVVPFISTHRAKTYLKSKRWSGGGGWTGIVCECCYNRCGYTELQQYCNQPTSKRSVSQSFFKKAVHHSNPQLAIKNPARILGNQ*

>Insulin-like peptide Nemertea_Lineus_ruber_Lvir.rna.tri.55397.1.p1

MLADFLLRSEDQWRDAWHTDCHRRCRHELDSQIRLACAKDIYRIRRAADVNVSNVTLPSIPKNHIFDRKTATAFLHKLHRQPIRMKRGIMEECCYAKSCTWEEYAESCHHYGRADADSSNICGP*

>Insulin-like peptide Nemertea_Lineus_lacteus_comp11212_c0_seq1.p1

MAKHSLHLAISLVLILTTSAIIGSHASQFCGKDLPRAMFIACGIAKRSAPSIETESDPTIDSIMKGNIRFRRSAEFADAVHSVFNENARSKRGVVPLNVLADVCCKAGCSLRDLAGAC*

>Insulin-like peptide Nemertea_Lineus_lacteus_comp15172_c0_seq1.p1

MIYQKLLVLLSFLVLIIHFNAQTDAIVLDPDVPQKLCSKDIHRAFQMSCLVSKRRKRSIDSDSGYFVRKTRFSQFRECCFRECTLRQLASLC*

>Insulin-like peptide Nemertea_Lineus_lacteus_comp30691_c0_seq1_m.250738

MAQAIPILLASILLLIEQLISTQASGLAKTCTKEDIQRGPAHGAACGPRLANLLNTICGQKGYYTGKITDLEFATNHEEDHLVPFISVHRAKAYVRSKKWDSTGWTGIVCECCYNRCGYRQLLQYCNTASNRISSYLKTPNTVVHSHTAIEHPTRSQGSRWLALGKRILHVFSRDRTSTK

>Insulin-like peptide Nemertea_Cerebratulus_spec_TRINITY_DN64887_c0_g1_i1.p1

MEVVYLSLVSLLVLLVTAPATLAVRTTTCTEEDLKRGPVRGALCGRRLANLLGTICGGDFYTGETSEQSDPSSTPENPTEHVVPFFSHQRANAYLRARRWDSQPWTGIVCECCYHRCGYRQLLEYCRIQSNRLTSHVKKS

>Insulin-like peptide Nemertea_Cerebratulus_spec_TRINITY_DN33108_c1_g1_i2_m.4872

MIYQKLLVLLSFLVLILHYNSQTDAILLDPDVPQKLCSKEIHRAFQISCLVSKRRKRSTDTESEDYFVRKTRFSQFRDCCYNECTLRQLASFC

>Insulin-like peptide Nemertea_Cerebratulus_spec_TRINITY_DN29418_c0_g1_i1.p1

MARYTLQLAVSLTIFLTISAIIGTNASRFCGRDLPRAMFIACGIAKRSAPSFESELDNEGNSLKGNLRYRRAAELADAAHSVFNEIERTKRGVVPLNVLADVCCKAGCSLRDLAGAC*

>Insulin-like peptide Nemertea_Cerebratulus_spec_TRINITY_DN13459_c0_g1_i1.p1

MKSKDARSHGPLVLYAALFITVTCLVHCTDAKRFDHMLADFLLRSEDDWRNAWHTDCHRRCRHELDSQIRLACAKDIYRIKRATDDTTTTNTTVPPFTQNHILDRQSATAFLHKLNPHSIRRKRGIMEECCYAKSCSWEEFAESCHHYGRSDAKTSNVCV*

## Achatin

>achatin_Nemertea_Tubulanus_polymorphus_comp75777_c0_seq1.p4

RGISDKRGISDKRGISDKRGISDKRGISDKRRIKNHHKSNQQPVAPTSHVQ*

>achatin_Nemertea_Cephalothrix_hongkongiensis_comp32592_c0_seq2_m.29026 3prime_partial

MIPRYLAPALSCMLCLCLYTYSVVATHDCDSDCEETLTDLIEDGYEKRGYGEKRGYGEKRGFGEKRGYGEKRAGPDLLSICLRKQASAA

>achatin_Nemertea_Cephalothrix_hongkongiensis_comp24315_c0_seq1_m.14725 internal

HHSLVFLRKHGKDLDAALACLIQNKDYLFRTLGLYDEEKRGFGEKRGFVDKRGFGDKRGFGDKRGDKRGFGDKRGFGDKRGFGDKRGFGDKRGFGDKRGFG

>achatin_Nemertea_Lineus_ruber_Lvir.rna.tri.32576.1.p2

MTTIQTLAALLFLSFCVTCHLVIAEECSDLTKCAVKEERLLKEEEEIVDKLLEEDGKRGFGDKRGFGD

>achatin_Nemertea_Lineus_ruber_Lvir.rna.tri.23128.1_m.46781 internal

DKRGFGDKRGFGDKRTFFDKRGFGDKRGFGDKRNEAEIFEFSDPAQEYYYICEILGYPCEKAKFAKMVGEDLLEEEQRGYGEKRGFGEKRGFGEKRGFGDKRGFGDKRGFGDKRGFGDKRGFGDKRGF

>achatin_Nemertea_Lineus_lacteus_comp19714_c0_seq1_m.37326 3prime_partial

MTTIQTLAALFFLLICVTCRLVIAEECSDLTKCAVKEENILKEEEEIVDKLLEEDGKRGFGEKRGFGDKRGFGDKRGFGDKRTFFDKRGFGDKRGFGDKRGFGDKRGFGDKRGFGDKRGFGDKRTFFDKRGFGDKRGFGDKRNEADVLKFSHPAEEYAYICEILGYPCEQSKYAELAGQELLEEETRGFGEKRGFGDKRGFGDK

>achatin_Nemertea_Cerebratulus_spec_TRINITY_DN34363_c0_g1_i1_m.187789 3prime_partial

MTTIQTLTVLLFLSVCVTLCHVVLSEDCHDLAKCALKDEVLKDTLEEELVEKLEGDKRGFGDKRGFGDKRTFFDKKGFGDKRGFGDKRGFGDKRGFGDKRGFGDKRGFGDKRGFGDKRGFGDKRGFGDKRGFGDKRTFFDRRGFGDKRGFGDKRSDVMELEYSDPVQEYAAICGLLGYPCEKAAYAKTDGQVILGEDKRGFGDKRGFGDKRMF

>achatin_Nemertea_Cerebratulus_spec_TRINITY_DN34363_c1_g1_i1_m.187794 5prime_partial

DKRGFGDKRGFGDKRMFGDKRGFGDKRGFGDKRMFGDKRGFGDKRGFGDKRGFGDKRGFGDKRGFGDRR*

>achatin_Nemertea_Lineus_longissimus_c38319_g1_i2_m.46828 complete

MTTVQTMAALLFLLICVTCHSVIAEDCSDLTKCAVKEEGILKEEEEIVDKLLEEDGKRGFGEKRGFGDKRGFGDKRGFGDKRGFGDKRTFFDKRGFGDKRGFGDKRGFGDKRGFGDKRGFGDKRGFGDKRTFFDKRGFGDKRGFGDKRNEAEIFEFSQPAEEYAYICEILGYPCEPAKYAEMAARDLLEEERRGYGEKRGFGDKRGFGDKRGFGDKRGFGDKRGFGDKRGFGDKRGFGDKR*

>achatin_Nemertea_Notospermus_geniculatus_g14915.t1

MFAEDELNGVKRVFGDKRGFGDKRGFGDKRGFGDKHGFGDKRRFGDKRVFGDKRVFGDKRIFGDKRGFGDKRGLGDKRGFGDKRGFGDKRGFGDKRGIGDKRGFGDRR

>achatin_Nemertea_Notospermus_geniculatus_scaffold1248.g24900.t1.p1

MEFVRFRHRLPVLSKSVAMTTIQTLAALLFLVFCITTCHSVIAEAELCSDDGKCSIKENELVKDKIEDEIVSKLLEVDEKRGFGDKRGFGDKRGFGDKRGFGDKRGFGDKRDKRGFGDKRGFGDKRGFGDKRGFGDKRGFGDKRGFGDKRNGVEHIDFRNPVEEYAYICEILGYPCERSKYATMFAEDALSGDKRGFGDKRGFGDKRGFGDKRGFGDKRGFGDKRGFGNKRGFGDKRGFGDKRGFGDKRGFGDKRGFGDKRGFGDKRGFGDRR*

## Allatostatin A

>allatostatinA_Phoronida_Phoronis_psammophila_comp704352_c0_seq1.p1

MVSVRNAVGMVFVAAICCATADENFDQQNKSATDTRRDVGQSSDIHVTKTAGFYHQPKKPKELSTYKVVLGRREKGSDDDNENRGPNNYDEKRAANWMYHVGLGKRESEEVGGTETDKRGHNSWMFTVGLGKREGASGT

>allatostatinA_Phoronida_Phoronis_psammophila_comp343275_c0_seq1.p2

KREGASGTGKDENNKPVDSEKAFDSEDGKRAASNWMYQVGLGKRDDIEGDKRAAKQWMYHVGLGKRDFEKASDNEDDKRAAKWMYHVGLGKRDFEKAFDNEDDKRAAKWMYHVGLGKRDFEKAFDNEDDKRAAKWMY

>allatostatinA_Phoronida_Phoronis_psammophila_comp68372_c0_seq1.p1

MNSRKQLGIALCAFFINVIVVCSEDVQPSNEERDSALDKRMDSRFFGPGFGKRDDGEVGDALLNEAKRMDSRFFSSGFGKRAGDVMKRLDSRFYSAGFGKRAENSKRLDSRFYGPGFGKRLDSRFYSAGFGKRDNDEKRMDSRFFSSGFGKRLDGRFYGPGFGKRDEEH*

>allatostatinA_Phoronida_Phoronopsis_harmeri_c70643_g1_i1.p1

DSRFFGPGFGKRMDSRFFSSGFGKRLDSRFYDAGFGKRDADEEKRMDHRFFGAGFGKRLDGRFYGPGFGKRK*

>allatostatinA_Phoronida_Phoronis_australis_TRINITY_DN212158_c0_g1_i1.p1

MKEPSVLFLSTLVLVYQVSGAQFSSDGMQGFDLTEEGANKSIKELLGSDNTLKSNKRAAGSWMYRVGLGKRVGLEGSKEKWLNSLENRFDLMRRNFDREAALNLFRNFIHKNFGEGVNRGNSWQYGLGKPGTDIKEDEKRLDRWIYRVGLGKREDGVAPLIDDQSRKLDSVEDGVEKRDALSWTHQVRRGKLANENEIKRAITSWMYKVGLGKRGGNDDYKRANGWMYRVGLGKRDENGIERATKDWIYHVGLGKRDQDSTKRAASWMYRVGLGKRGNGEKRNGNWMYRVGLGKRSDVEASREVDNSH*

>allatostatinA_Phoronida_Phoronis_australis_TRINITY_DN282543_c0_g2_i1.p1

MIRKQVGIVLCGFFVHVMVCSDDLKSSELKTDDGMNKRMDARFFSPGFGKRSESDDSIDRSLLKGMELDRPFYTTGFDKDDDLQKRLDSQFYSPGFGKRLYNNDKRLDSRFFHAGFGKRLDGRFYHATFGKRLDSRFYKPVFGKRDNAEEKRMDRKFFSAGFGKRLDGRFFGPGFGKRGVRK*

>allatostatinA_Phoronida_Phoronis_ijimai_(P._vancouverensis)_comp92753_c0_seq1.p1

MPTMNEPSLLFLFMLVLVCNVPGAQFHSDGIQGLDVTGEGTHGSLNKNHGLTPQYVGSDDLFRSNKRAAGSWMYRVGLGKRDSDESEKEKRTKNWMYTVGLGKRNNDGMEDEKRSSSWMYKVGLVKRNNDEREDEKRSSSWMYKVGLGKRNGDDEDEEKRTNGWMYKVGLGKRDSDVKEDEKRLNNWMYKVGLGKREDKGTPILDDTDLSHRSWKSDDVENDAGQRDAGSWTHGVGLRKQASENEIKRAITSWMYRVGLGKREANDDYKRSNGWMYRVGLGKRDRNEGERTTKSWLYHVGLGKRDNDNAKRTSGWMYRVGLGKRSGGEKRAASWMYRVGLGKRSDVDASSKDDKNSWMYRVGLGKRGSGVNVG*

>allatostatinA_Phoronida_Phoronis_ijimai_(P._vancouverensis)_comp89964_c0_seq1.p1

MIGKEVGIVLCGFFVHAIVCSDDLKSSGLKNDDGVNKRMDSRFFSPIFGKRSESDDGYDSSLLAKDTAYLKDKPELDGSFYSAGFGKGDDLQKRLDSQFYRAGFGKRSDDYKDKKLDNRFFHAGFGKRLDSRFYHAGFGKRLDSRFYSPSLGKRDNTGEKRMDRKFFSAGFGKRLDARFFGPGFGKRGVRK*

>allatostatinA_Phoronida_Phoronis_ijimai_(P._vancouverensis)_comp56769_c0_seq1.p1

MGSAILALCTVIIIQFAVSESLFLPESRLDNLGTDFDFCTCCTEKRNTECCALCDVSSLSAVSGDGNDGLETARDHVLEKLRAKYKFLPPLLSVDAAVNAKRQRGRKGTFDERFYKFQVGGKRRPFSTIEESLDSDKRSDSFFHGMAFGKRRSEKRADPFFHSPTLGKRSVNNRLNPWSRSGKLLSPVDQHEYPKWEKRSDMFFHSAQLGKRAQMFRKQSELADVVDGTPFMEAANNDEQDSEKMANNKDNVEFTDKTNSFKTNDFVKRDLNSDGSNVIVKPGRTIPFIYDSHGFRKRSPLNRIYKMFPNSRVPYLELDSRAKK*

>AllatostatinA_related_Phoronida_Phoronis_ijimai_(P._vancouverensis)_comp91267_c0_seq1.p1

MFSRATLREELMFLIVGMLFCHIVSGLRHEGHQRPLAIHNDFPISPIPNEMRSRQSSKTLWDGGEDPLETKSRRSGFNTVVGKRRHFEDLRDARSRRKRSNFNIGKRRHFEDLQDVSSRRKRFNTNIGKRRHFEESGGVNSEQKIPNTNVGKRRHFGDPEDVTSKAKSLNFIVGKRGHFEDPEDVNSGRTSWNINVGKGGFFDDLQDAKNRRATPNDN

>allatostatinA_Brachiopoda_Novocrania_anomala_Nano.rna.tri2.17586.1.p1

MMAGRTLNLHLIVLILVKLVCQSNGFNIPLLVPEDESYISSNYPSPKRVSSILVLKPAINRPLPLAQWQGLLADNNVDHDATSMERMENEVPQEKRFDPHMMLTGIGKRGRPTIFLRAPNGRPYVPKRGLDTMLILTGVGKRRRRRSADSNNPSDTAQSLESTQELDRKRRFDSNIMLHGLGKRPTVQRRMDNKMMLYGLGKKNEGASIGDNSYEHTPFDAMPAAVPISFSSNKRMDSRVMLMGLGRRNGKNDHLQHHAFFSKLTNEQYSPLYNHLKEMHTNPNGVHVSSEDHDSEKRRMDPRFMLTGIGKRASGLVNYSDKIKGGINE*

>allatostatinA_Brachiopoda_Glottidia_pyramidata_comp42006_c0_seq1.p1

MEPKQTTLPLFRLLVTLCAVTAVSTAGYVNPEFLIRNEAAERQQSTKRMDRNMLLMGLGKRNGIDKHVLFQGLGKRNDVYLEDLQTNPYSFSKRRMSRGMLLAGIGKRMDRNMLFTGIGKRPMDRTMVIQGLGRRADPDFLLSNLDDGGALRLNRRQDPNMLLMGLGKRDGYYYSDRPYIIVPKEDEFDTNSKYGEYSFADFDPQDRNLDSALRFGGLGKKRKRRSSSVDIPAVEKAKRSTAGDSVQSPDENDVSERQITARKRGIGRSMLFSGMGKRDFATKRGMDKSMLFSGFGKRGFDRSMLFSGMGKRSDPKRVMDSQMLLAGLGKRPHDEDEDEDEDEDHTAIFDQLAAAMDRPRNNSNNENDYFIGRGDGVDRARSILPGYLYSDDNDKKEKS

>allatostatinA_Brachiopoda_Lingula_anatina_comp129410_c0_seq2.p1

MEQRTSWSPLFRLIVTLCAVPGLTAADYVSPELLIRGTNGADRQQAFAEKRMDRNMLLMGLGKRNGLDKHVLFQGLGKRNGLYVTDFGPNPYTFSKRRMSRGMLLAGIGKRMDRNMLFTGIGKRPMDRAMLIQGLGKRADHDFLLSNLDDGVAAGIERRQDPKMILMGIGKRSALLNSHPYALMPEEDEFDLNPNFSEDDIQDYDLQERDLDNALRFGGLGKKRRRRSTAAVPSLEKDVSKRAADDSVLSEDQVVDGSKQKITAKKRGIDRAMLFSGMGKRDFNKRGMDRSMLFSGVGKRGFDRNMLFTGMGKRSDPKRVMDSQMLLAGLGKRPHDEDEDEDVDHTAIFNELADAMDRAESQKNASDDGNINTVDTAEDDRLKIPYNYIYDDDDNMNEKRYSTKMLLTGLGRRQDDAAQGEFDKRGIDRSMLFSGVGKRRLSKMLLTGLGKRRLDSSMLFSGLGKRGYSKLLLTGLGKRRLDSSMLFSGLGKRRMDSSMLFSGLGKRRLSNQMLFSGLGRRETKQNVDNSKRLNDDQSAITSGSEKRTVS*

>allatostatinA_Brachiopoda_Laqueus_californicus_comp36653_c0_seq1.p1

MDSSRRSTFLQRDYLILFFVRLSVLAMLISVTTSKDIHNDAMHESFVENHLRKRNTRMSLSELGTSVKRKIRHPMIPKYHMGLYGFGKKLMEPAHSHNRMYKKMDPSYSMFGVGKKMDPSFGLGGVGKKMD

>allatostatinA_Brachiopoda_Laqueus_californicus_comp30255_c0_seq1.p1

KKMDPSYSMFGVGKKMDPSYGLFGVGKKMDPSYGLFGVGKKMDPSYGLFGVGKKTNSGASILDAKRKRSLDPV*

>allatostatinA_Brachiopoda_Laqueus_californicus_comp42699_c0_seq2.p1

ITAGEARDYLPKRIYQLFCLEISEAIIMHMLHLVASHLFLVSYVEFGVLCLFEKDYAKFGKILPPPDIYDESKMKSSYTESGTKNIPLSELLRIIFSKKESDPADLDRLSKKHVKKRIDPVQFGYGIGKRIDPAQFGYGIGKRIDPSQFGYGIGKRMDPSQ

>allatostatinA_Brachiopoda_Terebratalia_transversa_Ttra.rna.tri.14227.1.p1

MNGQASTLIHSLQYFTKLWIVFNLLSTAVATSELHKDGRVGETNLRKRNTEMSSDVSKGTNNMKRNIRLMNPAYHLGLYGFGKKLMEPSDYYHHNRFYKKMDPSYSMFGVGKKMDPSFGLFGVGKKMDPSYSM

>allatostatinA_Brachiopoda_Terebratalia_transversa_Ttra.rna.tri.11838.2.p1

PSQFGYGIGKRMDPSQFGYGIGKRMDPSQFGYGIGKRFDSSQIGKRMNSAHQTREHTITNKERT*

>allatostatinA_Brachiopoda_Terebratalia_transversa_Ttra.rna.tri.11838.1.p3

PSQFGYGIGKRMDPSQFGYGIGKRMDPSQFGYGIGKRMDPSQFGYGIGKRMDPSQFGYGIGKRMDPSQFGYGIGKRM

>allatostatinA_Nemertea_Lineus_lacteus_comp13388_c0_seq1_m.18300 3prime_partial

MGGHARISSLPVILVCVFANLVLTLCAEINLGSASQDTRQKRDTNSIAALETGRERRSLNSKTNSIEIAEAAFGKHVLKKRSLENVEDTSFLDKRAENASPLKRQAEKSLGREKRDVDKNQFAGGLGKRLDSMMYGGGLGKKLDPMMYSGGLGKKLDPMMFSGGLGKKLDPMMYSGGLGKKLDPMMFSGGLGKKLDPMMFSGGLGKKLDPMMFSGGL

>allatostatinA_Nemertea_Lineus_lacteus_comp23373_c0_seq1_m.61383 5prime_partial

GLGKKLDPMMYAGGLGKKLDPMMFSGGLGKRVDSMRYSGGLGKRYDQMMFSGGLGKRLDRMMYGGGLGKKKKRVDPMMFGGGLGKRLDSMMYGGGLGKRETDVDRFMFGGGLGKRVDPMMFSGGLGKRSKRVDPMMFSGGLGKRDDDKRVDSMRYAGGLGKRDDDKLVDSIRYAGGLGKRDDDKRVDSMQFAGGLGKRLDSMRFAAGLGKRGDDKRVDPMMFSGGLGKRNHDLESASSSSEESKSSEEES*

>allatostatinA_Nemertea_Nipponemertes_spec_TRINITY_DN11954_c0_g1_i1_m.90788 internal

DGVQDHVIDKRSAEDDSDENGGGDVVNPYLDNLDSLAKRKIDSYLYAGGLGKRKIDPYLYAGSLGKRKIDPY

>allatostatinA_Nemertea_Paranemertes_peregrina_comp18820_c0_seq3_m.14749 internal

TFFFFLARFRARACYRTLLLHTLHTSIIYASSASSIEYPVKRTKRHPTAMLLPLYVIVFCAHILVTNCAETDDVNDNALQKRAVDPYMYAGSLGKRKLDTYLYAGSLGKRKIDHYLYQGSLGKRKVDPFLYQGSLGKRAQENDEKRAVDPYLYQGSLGKRAVDPYLFQGS

>allatostatinA_Nemertea_Paranemertes_peregrina_comp18820_c0_seq2_m.14746 internal

HYGRGQTTRDTKARACYRTLLLHTLHTSIIYASSASSIEYPVKRTKRHPTAMLLPLYVIVFCAHILVTNCAETDDVNDNALQKRAVDPYMYAGSLGKRKLDTYLYAGSLGKRKIDHYLYQGSLGKRKVDPFLYQGSLGKRAQENDEKRAVDPYLYQGSLGKRAVDPYLFQGS

>allatostatinA_Nemertea_Paranemertes_peregrina_comp24231_c0_seq1_m.26791 5prime_partial

GKRAVDPYLFQGSLGKRAVDPYLFQGSLGKRRVDPYMYAGSLGKRGVDPYMFAGSLGKRKIDQYLYAGSMGKRDGEKRAVDPYLFQGSLGKRKIDPYMYAGSLGKRDANKRAVDPYMYAGSLGKRGERSAHESDLEKKESS*

>allatostatinA_Nemertea_Paranemertes_peregrina_comp312568_c0_seq1.p2

MARRAWLLVICTVIYCAAQENNKDITAETPDGEQKKRSDPDDDGKRSDYMRYAGGLGKKA

>allatostatinA_Nemertea_Paranemertes_peregrina_comp19064_c0_seq1.p1

KKADYMRYAGGLGKKSSGVENPESETKSDIIRHKLLEFFNNDYPNLGLKRARIFRKWREQVAPSTAEVGGGGSPIKSYVSSPRDSIDPYRYMFGMGK*

>allatostatinA_Nemertea_Baseodiscus_unicolor_TRINITY_DN91217_c1_g3_i1_m.230137 internal

LDSMMFPAGLGKRLDPFMYGGTLGKRSDLGNQYDEESEADNDDGQSSLEKRGGGSKYKIDPYMYGAKIGKRVDPIMFGGNLGKRTQEKRVDSMMFGGSLGRKRVDPMMFGGSLGKRTTSKRVDPIMFGGSLGKRDGDKRLDPMMYGGNLGKRGDEKRLDPMMYGG

>allatostatinA_Nemertea_Baseodiscus_unicolor_TRINITY_DN91217_c1_g4_i2_m.230139 internal

KKLDPMMYGGNLGKRVDPLMYGGTLGKRYDDKRVDPMMFGGSLGKRSGDKRLDPMMYGGNLGKRDDDKRIDPMMYGSSLGKRNTDKRLDPMMFGGSLGKRDDDKRVDPMMFGGSLGKRNGDKRLDPMMYGGNLGKRDGDKRVDPLMFGGSLGKRSIDKRLDPIMYGSSLGKRSVDKRLDPMMYGGNLGKRNTEKRLDPMMFGGSLGKRDDEKRLDSMMFGGSLGKKSADKKLDPMMYGGNLGKRGDDKRLDSMMFGGSLGKKSADKKLDPMMYGGNLGKRGDDKRLDSMMFGGSLGKRGDEKRVDPMMFGGSLGKKDSDKRVDPLMFGGSLGKRMATSGKENGHLDEGITGDGSSLSVSGANPLPLEGELSRGDDNLIYDNDDSDRG

>allatostatinA_Nemertea_Baseodiscus_unicolor_TRINITY_DN91217_c1_g5_i1_m.230141 3prime_partial

MGKPHTTSVVKAILLFILINSPFTICEVQESSKMTLDDRLISEKLGNMQERATLYHPGLAKRSVDSLISPMAMEGRRVSEKRSKLKLGPFRKKRSTTHEDHPDEINNEFDATDSLISAKKRSVDESLVQSAIKNDLHKDLSTGGKEERNKRDVDPTMYSGNLDKKRMDSMMYNFGLGKKRLDPMMYGVGLGKKRLDPFMYNSGSDKKSIDSLAYSHGQGEKGLHPLSQNGNHFDEGGLESLMYGVGLDRKRLDHMMFGSTLGKKRTGLTENDMDVQKRLDSMMFPA

>allatostatinA_Nemertea_Riseriellus_occultus_TRINITY_DN49025_c0_g1_i1.p1

SSTQGVKLKSVIYSSTQGVKLKAVIYSGPQGVKLKSVIYSSTQGVKLKPVTYSGPQGVKMKPVIYSGPQGVKLKSVIYSSTQGVKLKSVIYS

>allatostatinA_Nemertea_Malacobdella_grossa_comp210262_c0_seq1_m.155594 3prime_partial

MLLPLYIISFCAHILATNCAETDEVIDEQKRAVDPYLYAGSLGKRKLDTHLYAGSLGKRKIDPYLYQGSLGKRNNDKRAVDHYLYQGSLGKRAVDP

>allatostatinA_Nemertea_Malacobdella_grossa_comp26181_c0_seq3_m.47946 internal

PLRPGEVEALSGGSISAYKTAHLPVQLQYYRDFQYITAETPDGVDGKGTDFNGEDKRTDFMRYGGGLGKKADYMRYAGGLGKKADYMRYAGGLGKKADYMRYAGGLGKKADYMRYGGGLGKRADYMRYGGGLGKKADYMRY

>allatostatinA_Nemertea_Malacobdella_grossa_comp26181_c0_seq2_m.47942 internal

YGRGLSLSNSLSLVDGSGGSISAYKTAHLPVQLQYYRDFQYITAETPDGVDGKGTDFNGEDKRTDFMRYGGGLGKKADYMRYAGGLGKKADYMRYAGGLGKKADYMRYAGGLGKKADYMRYGGGLGKRADYMRYGGGLGKKADYMRY

>allatostatinA_Nemertea_Malacobdella_grossa_comp10664_c0_seq1_m.4717 5prime_partial

KRAVDPYLYAGSLGKREVDRYLYAGSIGKRNNDKRAVVGKRAVDRYLYAGGLGKRGADKRAVDHYLYAGSLGKRKVDSYMYAGSLGKRSVSSEGETKSS*

>allatostatinA_Nemertea_Malacobdella_grossa_comp26181_c0_seq1.p1

KADYMRYAGGLGKKADYMRYAGGLGKKSDEPKTPFESENKSESIRDKLLEFFDKDYPKIGLKRARIFRKWREQVTPSGGSSGAARSPIGSLMTLHRFGVDPYRYMFGMGK*

>allatostatinA_Nemertea_Notospermus_geniculatus_g2258.t1

MMFGGGLGKKLDPMMFSGGLGKKLDPMMFSGGLGKKLDPMMFSGGLGKKLDPMMFSGGLGKRLDSMMFGGGLGKKRVDPMMFGGGLGKKRVDPMMFGGGLGKKRVDPMMFGGGLGKKRVDPIMFGGGLGKKRVDPMMFGGGLGKKKRVDPIMFGGGLGKKKRVDPMMFGGGLGKKKRVDPMMFGGGLGKRESNIDPIMFGAKLGKRLDSMMFSGGLGKRAVDPMMFSGSLGKRDDDKRVDQMMFSGGLGKRGDNKRLDPMMFSGGLGKRSSSEENSSSQSESESKESEEES

>allatostatinA_Nemertea_Notospermus_geniculatus_g32563.t1

MGVHSRISSLPICIIAVCALANLALVICDDVRDDDVRQKRDINSKTALGNDRERRSLLSKIHNLKSESSGASHTLQKRGVDDDVYDLDKRLDSMMFGGGLGKKLDPMMFSGGLGKKLDPMMFSGGLGKKLDPMMFSGGLGKKLDPMMFSGGLGKRLDSMMFGGGLGKKRVDPMMFGGGLGKKRVDPMMFGGGLGKKRVDPMMFGGGLGKKRVDPIMFGGGLGKKRVDPMMFGGGLGKKKRVDPIMFGGGLGKKKRVDPMMFGGGLGKKKRVDPMMFGGGLGKRESNIDPIMFGAKLGKRLDSMMFSGGLGKRAVDPMMFSAVDSESDLHLRKTHHRSRSRNRKSLRRNLRMIAPKVWTRASKNP

>allatostatinA_Nemertea_Notospermus_geniculatus_g41482.t1

MTNGERGGGSGHGKRSLTKDADDGQRPGQLGKRSLTKDADDGQRPGGGLGKRSLTKDADDGQRPGGGLGKRSLTKDADDGQRPXLGKRSLTKDADDGQRPGGGLGKRSLTKDADDGQRPGGGLGKRSLTKDADDGQRPGGGLGKRSFTKDADDGQRPGGGLGKRSLTKDADDGQRPGGGLGKRSLTKDADDALRSSN

>allatostatinA_Nemertea_Cerebratulus_marginatus_comp19913_c0_seq1.p3

MRFSGGLGKRDEDKRVDSMRFAGGLGKRDDDKRVDSMRFAGGLGKRDNKRLDSMRFA

>allatostatinA_Nemertea_Cerebratulus_spec_TRINITY_DN31152_c1_g1_i1_m.87411 3prime_partial

MGGPSRISSLPIVLVCVFANLALSICGDLTHVGSVSQHTRQRRDTNSIAALESGLNSKIDTLDAQQSDHVLKKRSLENVEDTSFLDKRAENSALKRRIEEYEWRNKRGVDPNRFAGGLGKRLDSLMYGGGLGKKLDPNMFAGGLGKRPDPMMFSGGLGKKFDPNMFAGGLGKKLDPNMFAGGLGKKLDPNMFAGGLGKRPDPMMFSGGLGKKLDPNMFAGGLGKRPDPMMFSGGLGKKLDPNMFAGGLGKRPDPMMFSGGLGKKYDPNMFAGGL

>allatostatinA_Nemertea_Cerebratulus_spec_Contig1466.p1

GLGKRPDPMMFSGGLGKKLDPNMFAGGLGKRPDPMMFSGGLGKRVDSMRFAGGLGKKLDPNMFAGGLGKRPDPMMFSGGLGKRVDDLMFGGGLGKKRKRVDSVMFGGGLGKRVDPMMYNGGLGKRDSLRKRFDSMMYNGGLGKRDVDSVMFAGGLGKRSKRVDPNMFGGGLGKRANDKRVDSMRFAGGLGKRADDKRVDSMRFAGGLGKRLDPMMYSGGLGKREQEKRLDSMMYGGGLGKRSKGKSTVKDSEEESDSESSEEEDSESSEEEDSESSEE

>allatostatinA_Nemertea_Lineus_longissimus_c23865_g1_i1_m.13840 3prime_partial

MGGHARISSLPVILVCVFVNLVLTLCAEINLGSASQDSIQKRDTNSIAALETGRERRSLNSKTNSIKSAKAALGEHVLKRRSLENVEDTSFLDKRAENASPLKRQVDRLLGRDKRDVDKNRFAGGLGKRLDSMMYDVGLGKKLDPMMFSGGLGKKLDPMMFSGGLGKKLDPMMFSGGLGKKLDPMMFSGGLGKKLDPMMFSGGLGKKLDPMMFSGGLGKKLDPMMFSGGLGKRVDSMRYSGSLGKRYDQMMFSGGLRKRLDRLMYGGGLDETKKRVDSMKFGGGLGKRLDSMMFGGGLGKRDVGVDKFRFGGGLGKRVDPMMFSGGLGKRSKRFDPMMFSGGLGKRDKDKRVDSMR

>lineusLongissimus.6046 Nemertea

MGGHARISSLPVILVCVFVNLVLTLCAEINLGSASQDSIQKRDTNSIAALETGRERRSLNSKTNSIKSAKAALGEHVLKRRSLENVEDTSFLDKRAENASPLKRQVDRLLGRDKRDVDKNRFAGGLGKRLDSMMYDVGLGKKLDPMMFSGGLGKKLDPMMFSGGLGKKLDPMMFSGGLGKKLDPMMFSGGLGKKLDPMMFSGGLGKKLDPMMFSGGLGKKLDPMMFSGGLGKKLDPMMFSGGLGKRVDSMRYSGSLGKRYDQMMFSGGLRKRLDRLMYGGGLDETKKRVDSMKFGGGLGKRLDSMMFGGGLGKRDVGVDKFRFGGGLGKRVDPMMFSGGLGKRSKRFDPMMFSGGLGKRDKDKRVDSMRFAGGLGKRNDDKR

>allatostatinA_Nemertea_Lineus_longissimus_c38618_g1_i1_m.48289 5prime_partial

AGGLGKRDDKRVDSMRFAGGLGKRLDSMRFAGGLGKRDGDKRVDPMMFSGGLGKRNQGSLQSESSFSEESKSSEEES*

>allatostatinA_Nemertea_Lineus_ruber_Lvir.rna.tri.33752.1_m.65276 5prime_partial

DKRVDSMRFAGGLGKRDDDKRVDSMRFAGGLGKRDDDKRVDSMRFAGGLGKRDEDKRVDSMRFAGGLGKRLDSMQYAGGLGKRSDDKRVDPMMFSGGLGKRNQDSQSESSSSEESKESEEES*

>allatostatinA_Nemertea_Lineus_ruber_Lvir.rna.tri.49412.1_m.90348 internal

KKLDPMMYAAGLGKKLDPMMFSGGLGKRVDSMRYAAGLGKRYDQMMFSGGLGKRLDSMMYGGGLGKKKKRVDPMMFGGGLGKRLDSMMYGGGLGKREADVDEFMFGGGLGKRVDPMMFSGGLGKRSKRVDPMMFSGGLGKRDD

>allatostatinA_Nemertea_Lineus_ruber_Lvir.rna.tri.31767.1_m.61895 3prime_partial

MGGHARISSLPVILVCVFANLALTLCAEINLGSASQDTRQKRDTNSIAALETGRERRSLKSKTNNLKSAEAALGEHVLKRRSLENVEDTSFLDKRANESPLKRQVERFLGRDKRDVDENRFAGGLGKRLDSMMYGGGLGKKVDPMMYSGGLGKKLDPMMFSGGLGKKLDPMMYSGGLGKKLDPMMFSGGLGKKLDPMMYSGGLGKKLDPMMFSGGLGKKLDPMMYSGGLGKKL

>AllatostatinA_Tubulanus_Polymorphus.12415 Nemertea

MAASERLTFIVKLLMLLTYIGFIFCETIERQNLKHEDGDSKTVVVKRSVHSLRRRDVDVMRFGGRLGKRVDQFRFNGGLGKRLDNMRFGGGLGKRDHDFDLNGDGEQEKRIDNFRFNGGLGKRDGDKRVDHNRFSGGLGKRDGDKRFDNVRFSGGLGKRDVDVESDGVSSESSDQSTAEKRLDNMRFTGGLGKRLDHMRFTGGLGKRLDHMRFTGGLGKRFDSVHFTGGLGKRLDSMRFTGGLGKRLDSMRFTGGLGKRLDSMRFTGGLGKRLDTMRFTGGLGKRLDTMRFTGGLGKRMDPMRFSGGLGKRYDNLRFSGGLGKRLDTMRFGGGLGKRLDTMRFGGGLGKRYDDDEDEAGDKNDDIEDEDTKRAVDSTRFSHTLGKRFDSIRYNVGLGKRENPYSKRYDSMRFVGGLGKRMDPMRFTHGLGKRDTLADKSKDTKSSPASQSAHRDTSV

## allatostatin A2 – nemertean cluster

>allatostatinA2_Nemertea_Cerebratulus_marginatus_comp32303_c0_seq1_m.17793 internal

HMKYQTLGLGKRGIDHMKYQTLGLGKRDDEDEKRGVDHVRYQTLGLGKREDDKRGVDHVRYQTLGLGKRDGEKRGIDHVRYQTLGLGKRGIDHVRYQTLGLGKRQDIESEKRGIDHVRYQTLGLGKREGIDKKGIDHVRYQTLGLGKRDDEKRAIDHMKYQTLGLGKREGEGEKRGIDHMKYQTLGLGKRQEEDAEKRAIDHLKFQTLGLGKRSEDSQSSEHARIQRAVDYIGGYRPYKRGMDYVLYRNLGLGKRAAGDLDLDDGDLENADHPELS

>allatostatinA2_Nemertea_Lineus_lacteus_comp4978_c0_seq1_m.5250 3prime_partial

MTMRPAVICLLVMVICLARGSTVQQERNDDSGEEATMENSEKSKKDTSEYLSDDGPNDVIDSGAKRGFDRNMYLYGLGKRMDERVPDGGLKRAMDLFKYRTLGLGKRGFDYSDDEMKRGIDHLRYQTLGLGKRGIDHVRYQT

>allatostatinA2_Nemertea_Lineus_lacteus_comp25241_c0_seq5.p1

GLGKRGIDHLRYQTLGLGKRDSDDEDEKRGIDHLRYQTLGLGKRDDDKRGIDHVRYQTLGLGKRNDDKRGIDHLRYQTLGLGKRGIDHVRYQTLGLGKRGDMEAEKRGIDHVRYQTLGLGKRQGIDKKGIDHVRYQTLGLGKREGEKRGIDHMKYQTLGLGKRGDGEKRGIDHMKYQTLGLGKRDGDAAEKRGIDHMKYQTLGLGKRNDQSDSSEHTRIQRAVDFIKYRNLGLGKRAGTYQPYKRGMDYVLYRNLGLGKRGLGDLDLGENDIRSELENADLDDESYPMMDMDELGKKNEAAESRNTDLNSIKRRLRNFLRYRYMGLSKKRARLFK

>allatostatinA2_Nemertea_Cerebratulus_spec_TRINITY_DN38031_c4_g1_i1_m.109371 3prime_partial

MRPAVVCLLVMVICLVRGSTVQEKRNDESEEAVPADNTEKTKKDYMGDEESEKRAIDTNMFLYGLGKRMDENMPSDGLKRAMDLFKYRTLGLGKRGIDHMKYQTLGLGKRGIDHMKYQTLGLGKRGIDHMKY

>allatostatinA2_Nemertea_Cerebratulus_spec_TRINITY_DN38031_c4_g3_i3_m.109380 5prime_partial

GIDHMKYQTLGLGKRGIDHMKYQTLGLGKRGIDHMKYQTLGLGKRGIDHMKYQTLGLGKRGDDEEEEKRGIDHVRYQTLGLGKRDDDKRGIDHVRYQTLGLGKRDGEKRAIDHVRYQTLGLGKRGIDHLRYQTLGLGKREDMEAEKKGIDHLRYQTLGLGKREGMDKKAIDHVRYQTLGLGKREGEKRGIDHMKYQTLGLGKREGEGEKRGIDHMKYQTLGLGKREGNEEAEKRAIDHLKYQTLGLGKRSDDSDSSEHARIQRAVDYIGSYRPYKRGMDYVLYRNLGLGKRSAGDLDLDDEMENADLEDSYPMVDMDELGKKSDAAESRNSDLNSIKRRILDFLKYRYVGLSKKRARLFKMWRKFGTIKPSPQLRTRVDPYRYMFGIGK*

>allatostatinA2_Nemertea_Baseodiscus_unicolor_TRINITY_DN86636_c0_g2_i1_m.216360 3prime_partial

MRPLVISMLAFLICLSASGSAEENHAASAAESTENQHDESMVAQQIYGSKGYTKEPWTLNGKLKQSPDVAGLSAHKRDIDRMKFHYGLGKREHDDDLKRAMDYLKFRNLALGQKRALDYFKHMNIGLGKRGDSGVVGGYKRAMDHFRFQTLGLGKRGIDHLRQQTLGLGKRNTGEDEKRGIDHTRFQTLALGKRNEPISDGLKRGIDHLKFTTLGLGKREDNVNAKRGLDHLRFQTLGLGKRGFDSLKYHVGLGKRDDVTDAKRGMDHLKYQTLALGKRHDTSGSSKRALDHLKFQTLGLGKRDDSNKRGVDHLRFHTLGLGKRDISAPSLQHRILAKRDANFFGYKGSSDSSMFDPYDKKGLDYTLFRNLGLGKRGMDLFKFRNIGLGKRQTGDDVYIPDRNDIISGLDNSDETEPSDMDLPEDLDMNALDELVPLHRN

>allatostatinA2_Nemertea_Notospermus_geniculatus_g18752.t1

MSVCNDDGDSYGVKKVKTFKTWKKLSDDSNEEAIVANSEKSKKDMSQYLSDDVADDVIDSGAPKRAIDRNMFLYGLGKRADEQMPDLKRAMDLFKYRTLGLGKRESDYNDETKRGIDHLRFQTLGLGKRGNDHLRYRTLGLGKRGIDHLRFQTLGLGKRGIDHMRYQTLGLGKKDGSDNDKRGIDHMKFQTLGLGKRDDDKRGIDHMRYQTLGLGKRDGDKRAIDHLRYQTLGLGKRGIDHLRFRTLGLGKRDGEKRGIDHMKYQTLGLGKREGEGEKRGIDHMKYQTLGLGKRQGDEEKRAIDHMKYQTLGLGKRDGEEADKRGIDHMKYQTLGLGKRNDDLESAEHSRTERAVDYIKYRTLGLGKRGSEYQPYKRKMDYVLYRGLGLGKRGLDYVKYGTLGLGKRDIDSDDLEENDIRPDLDDADVDDESFPTVDENSIDELVPLVRN

>allatostatinA2_Nemertea_Lineus_longissimus_c50560_g2_i1_m.171868 complete

MTMRPAVFCLLVMVICLARGSTVQQERNDDSGEEATMENSEKSKKDTSEYLSDDSTSDVIDSGAKRGFDRNMYLYGIGKRMEERMPDAGLKRAMDLFKYRTLGLGKRGSDYSDDEMKRGIDHLRYQTLGLGKRGIDHVRYQTLGLGKRGIDHVRYQTLGLGKRGIDHLRYQTLGLGKRDGDDEDEKRGIDHLRYQTLGLGKRDDDKRGIDHVRYQTLGLGKRDDDKRGIDHLRYQTLGLGKRGIDHLRYQTLGLGKRDDMEAEKKGIDHLRYQTLGLGKREGIDKKGIDHVRYQTLGLGKREGEKRGIDHMKYQTLGLGKREDGEKRGIDHMKYQTLGLGKRDGDDAEKRGIDHMKYQTLGLGKRNDESESSEHTRIERAVDFIKYRNLGLGKRDRAYQPYKRGMDYVLYRNLGLGKRAMSDLDLGENDIRPELENADLDDESYPMLDMDELVPYIRN*

>allatostatinA2_Nemertea_Lineus_ruber_Lvir.rna.tri.37019.1_m.71028 complete

MTMRPAVFCLLVMVICLARGSTVQEERNDDLGDEATMENSEKSKKDTSEYRSDDNTNDAVDPGAKRGFDRNMYLYGLGKRMEERMPDASLKRAMDLFKYRTLGLGKRGSDYSDEETKRGIDHLRYQTLGLGKRGIDHVRYQTLGLGKRGIDHVRYQTLGLGKRGIDHVRYQTLGLGKRDGDDEDEKRGIDHVRYQTLGLGKRDDGKRGIDHVRYQTLGLGKRDDDKRGIDHVRYQTLGLGKRGIDHLRYQTLGLGKRDDMKAEKKGIDHVRYQTLGLGKREGIDKKGIDHVRYQTLGLGKREGEKRGIDHMKYQTLGLGKRDEGEKRGIDHMKYQTLGLGKRDGEDAEKRGIDHMKYQTLGLGKRNDESESSEHTRIQRAVDFIKYRNLGLGKRNGPYQPYKRGMDYVLYRNLGLGKRAMSDLDLEENAIRQELENADLDDESYPMIDMDELVPLIRN*

## Allatostatin B/Myoinhibitory peptide (MIP)

>lingulaAnatina.g3829.t1 Brachiopoda

MAAITADSLTITFFLTLMVLSSPLVHCVINSQMLRDAQNLDQAPYGRYIEEDIVIPNDSQEEENSHRYQSQAVSLKRLEYLFNKLENNIMRNEPLKRQWSKSMTMWGKRSPYFYSEDMEDPHALRLRRRSLEKTDDNNKTKEKTDFQLTNNVIRSWKSGGMKLWGKRPFDKEMEESRDADLKKLGENMDWKQKERDLKRSWSGNAMKLWGKRDNTFDDQHDKKSWSSKNMKLWGKREDDKRSWNSKSLKVWGKRDVDQDEEENNKRSWSSKNMKLWGKRNEQKDGLEYEKRPWSSKSMKLWGKRDNSVEADINEKRQWNSNGMKVWGKRMDGEIIDELSNKRKWNSKEMKLWGKRDSVQHNALNEKRGWNAKGMKLWGKRSENDEKRPWNGKGMKLWGKRDSSGYESESNNYPYLYRKHSIDVDSKRSWKSGGIKLWGKRDMVDDDVDLVSNTAGGVKRPWNSGMKLWGKKRASDSLRDLDSAKSDSWIQDGKRSWIGKNPWATKIGFDPSKRWRSMKLWGKRDSGQSQHDYEDAFNKWNSMKLWGKDLTSLIKAEVGNNPNTKTLSNEFEEKVFDHGVDNETNTETGSSPLNKEISNEHVTTRAHTLSKRSGWMPREGLRNMWG

>allatostatinB_Brachiopoda_Lingula_anatina_comp156051_c0_seq6.p1

MAAITADSLTITFFLTLMVLSSPLVHCVINSQMLRDAQNLDQAPYGRYIEEDIVIPNDSQEEENSHRYQSQAVSLKRLEYLFNKLENNIMRNEPLKRQWSKSMTMWGKRSPYFYSEDMEDPHALRLRRRSLEKTDDNNKTKEKTDFQLTNNVIRSWKSGGMKLWGKRPFDKEMEESRDADLKKLGENMDWKQKERDLKRSWSGNAMKLWGKRDNTFDDQHDKKSWSSKNMKLWGKREDDKRSWNSKSLKVWGKRDVDQADEENNKRSWSSKNMKLWGKRNEQKDGLEYEKRPWSSKSMKLWGKRDNSVEADINEKRQWNSNGMKVWGKRMDGEIIDELSNKRKWNSKEMKLWGKRDSIQHNALNEKRGWNAKGMKLWGKRSENDEKRPWNGKGMKLWGKRDSSGYESESNNYPYLYRKRSIDVDSKRSWKSGGIKLWGKRDMVDDDVDLVSNLRPAGGVKRPWNSAMKLWGKKRASYSVGDLDSAKSDSWIQDGKRSWIGKNPWATKIGFDPSKRWRSMKLWGKRDSGQSQHDYEDAFNKWNSMKLWGKDLTSLIKAEVGNNPNTKTLSNEFEEKVFDHGVDNETNTETGSSPLNKEISNEHVTTRTHTLSKRSGWMPREGLRNMWG*

>allatostatinB_Brachiopoda_Glottidia_pyramidata_comp11615_c0_seq1.p1

GHYGRGEDLSAEEDNVKRGWQSKGMKLWGKREDKMNGLEDEKRSWNSKSMKLWGKRDEEENDEKRAWNSKNMKLWGKREDEFDEKRQWKSDGMNLWGKRVADEDIDDLVNKRRWKSNGMKLW

>allatostatinB_Brachiopoda_Glottidia_pyramidata_comp31886_c0_seq1.p1

MATSKAASLAVKFFLTIMVLSTSVAQGEDKSEQLSVNHIQNPSQMPYGKYIEVDILQPHRPKEIENSRRYGTQALSLIRLEYLSNKGQGINYMNISPSKRQWSKSMTMWGKRSPYVLSEHLSYPKVLRLQKRSIEKINKTADKAKSDTPSDDKRGWKSGGMKLWGKRLFDEETDGKRGWNSKNIKLWGKRDNIGNDDLEEKRGWNSKSMKLWGKRDGSANSEVDNDLEWRQNDDLGEDFEAKRAWNSKSMKLWGKRDDDKRSWNSKSMKLWGKREDLSAEE

>allatostatinB_Brachiopoda_Novocrania_anomala_Nano.rna.tri2.151646.1.p1

MDTTMSGWLAIISFLAALSSFANGSVLEDLKLIVSSEEDTENRNTVPHPIYQRWTKELLDEALSQSWELRDLGELLKIAYREELNQDPFGDNVVDLLATKRSNGANDMTISDSLLMLLRALQKNSAAPKYTRSRYSAPERQQTKWSAIKTWGKRFLDDESQPKERRKRDIHDQAPSIRTEKWGDMTLWGKRARMTAPAVSSLELWGKGVQEDSSKPAISDKNEIKKWKDMKIWGKRGDDNLSDKAELPKDKKWQGMKMWGKRENDELSHAKKWQGMK

>allatostatinB_Brachiopoda_Novocrania_anomala_Nano.rna.tri2.175781.1.p1

WQGMKMWGKRGNDEVSKNKAWQGIKIWGKRSDIPLLDKKWQGMKTWGKRNDIPLADKKWQGMKMWGKRSDIPLWDKRWQGMKMWGKR

>allatostatinB_Nemertea_Lineus_ruber_Lvir.rna.tri.34874.1_m.67347 3prime_partial

MWGKRNADRRAWNQMGMWGKRDAEKRTNVPAAAEKDKRTWNDMNMWGKRTARDVSDVKRNWNGMNMWGKRSGDKKAWNEM

>allatostatinB_Nemertea_Lineus_longissimus_c47054_g1_i1_m.123243 5prime_partial

AWNEMGMWGKRNADRRAWNEMGMWGKRNWNGMNMWGKRSDDDSSKIMDAIIANKMEKLLNQASE*

>allatostatinB_Nemertea_Lineus_longissimus_c5118_g1_i1_m.2532 internal

EMGMWGKRNADRRAWNQMGMWGKRDAEKRTNVPAAAEKDKRTWNDMNMWGKRTARDVSGVKRNWNGMNMWGKRDGDKKAWNEMGMWGKRNWNGMSMWGKRDAEQATNEEKRAWNEMGM

>allatostatinB_Nemertea_Lineus_lacteus_comp18127_c0_seq1_m.30824 internal

KKAWNEMGMWGKRNADRRAWNQMSMWGKRDAEKRTNVPAAAEKDKRTWNDMNMWGKRTARDVSDVKRNWNGMSMWGKRDGDKKAWNEMGMWGKRAADKKAWNEMGMWGKRDGDKEKKAWNEMGMW

>allatostatinB_Nemertea_Cerebratulus_marginatus_comp1302542_c0_seq1_m.146259 5prime_partial

WGKRDGDKRAWNEMGMWGKRNWNGMNMWGKRNWNGMNMWGKRSTDDSSKIMDAILANKMAKLIDQNESE*

>allatostatinB_Nemertea_Cerebratulus_marginatus_comp631244_c0_seq1_m.124622 internal

YSGRGMWGKRDAGKRTWNDMKMWGKRDTASATAEDKDKRTWNDMKMWGKRTARDVSDVKRNWNGMSMWGKRDADKKAWNEMGMWGKRNWNGMSMWGKRDAEKRNWNDMKMWGKRDLEDATEEEKRAWNEMGMW

>allatostatinB_Nemertea_Baseodiscus_unicolor_Contig20507_m.255809 5prime_partial

HKDIPFQLRLPHILKSFQPRFSSGESLWGKRDGEMGQLADSKRNWNGMNLWGKRSDGSSKIMDAVIADKLVKLFDNNGDGTLDKDEIRSFVAWLTTLNPGSMAVVAAA*

>allatostatinB_Nemertea_Baseodiscus_unicolor_TRINITY_DN96568_c1_g2_i1.p3

QLWGKRGWTDMTLWGKRDSPQLHGVPSSFSVVITVISLAPYAQIVPASLICLGILQHVTASTFSPQPHAIPVAFTPEPHTVPVTLLLAVTSISNTSLAPQSHLVPATFFVIRSLAP*

>allatostatinB_Nemertea_Baseodiscus_unicolor_TRINITY_DN96568_c1_g2_i1_m.186568 5prime_partial

RGWNDLGIWGKRSNDDAGDADKKNTWNDMGLWGKRNWNGMNLWGKRADDKKRGWNQMGLWGKRSIRDTGDSEEKRNWNGMGLWGKRNWNGMGLWGKRAGSDMLEDAKADKRGWNDLGVWGKRDDSDDDAKRAWNTMQLW*

>allatostatinB_Nemertea_Nipponemertes_spec_TRINITY_DN28527_c1_g4_i1_m.41841 5prime_partial

KRDGDKKAWNDMGLWGKRAPERKRSWNDLNLWGKRDGDKRTWNDMQLWGKRDQAAVHQD*

>allatostatinB_Nemertea_Nipponemertes_spec_TRINITY_DN28527_c0_g1_i1_m.41835 3prime_partial

MADTRLWLRLILVIAICAVITAAEDKSDKLESDKKKRHVTDSDEKETGQWDKRAWNDMGLWGKRTWNDMQLWGKRSGDKRTWNDMQLWGKRDAEKRAWNDMGLWGKRDGDKRTWNDMQLWGKRDGNKRTWNDMQLWGKRNDDKRTWNDMQLWGKRDGD

>allatostatinB_Nemertea_Cephalothrix_hongkongiensis_comp12004_c0_seq1_m.5980 5prime_partial

LKRGWNNAGAWGKRDSNDDNKRGWDNAGAWGKRDATEKEKRGWNNAGAWGKRGWNNAGAWGKRGWDNAGAWGKRSDSKLDDAIVEKIYGIFDSNGDKCLGEDEVKEFVNWLTKLTRAKQQQETQE*

>allatostatinB_Nemertea_Cephalothrix_hongkongiensis_comp18402_c0_seq1_m.9506 internal

WDFSTICAEDSNTESENKRDSDTELKDKRQGGLKWNKHSLPWGKRGWNNNGAWGKRSMTEEERKAWTDRYENAKRVWKNINTLKNDEVSSDIEDVLKRGWNNNGAWGKRNNDNEKRGWNNAGAWGKRDAADDEKRGWNNNGAWGKRGWNNNGAWGKRDNEKRGWNNAGAWGKRGWNNA

>allatostatinB_Nemertea_Tubulanus_polymorphus_comp30192_c0_seq1_m.23203 5prime_partial

AWKTAMWGKRDSDDDEKRAWKTAIWGKRSASNKDGTGVQKRGANRLNLWGKRKWNTGLWGKRDAGQDNNIDKRKKWNNGLWGKRKWGVDMWGKRSANKMDKSAF*

>allatostatinB_Nemertea_Tubulanus_polymorphus_comp35051_c0_seq1_m.44109 3prime_partial

MGTNSFVMWLLFLVGLFSFIFAEMSEISNKSTESANEKEALLSKDQRNIQKRQAGRWTKNETPLGDRNLGNIKIWGKRDTSLNDANNVKRNKLSTLSLWGKRQSKISVWGKRSAQAPHSVSDGEKRKWETGVWGKRDGSASDKRAWKTGVWGKRDDSDSNKRAWKTALWGKRDNNDSDKRAWKTAMWGKRDDSDSNKRA

>allatostatinB_Nemertea_Malacobdella_grossa_comp23793_c0_seq3_m.28049 3prime_partial

MCECKHWIRLVLVLGLSTLIILADEAKVEKEKSEQITDKDDKRAWNDVNLWGKRAWNDMSLWGKRDAKPTWNDMALWGKRDAEKRAWNDMSLWGKRDGTKSTWNDMALWGKRSTDDKRAWNQMGLWGKRNDQKRAWNDMSLWGKRSGDKKAWNDMSLWGKRGAEKRSWRDVGLWGKRQVMSDWNRKRTWNDMNLWGRKKRTPQDSKKWNDMALWGKKEKS

>allatostatinB_Nemertea_Paranemertes_peregrina_comp29563_c0_seq1_m.57480 complete

MYESKNWIRLVLVLGLSALLTLADDTKAQDTEKREEVADNDDKRAWNDVNLWGKRAWNDMSLWGKRDAKRTWNDMALWGKRDGDRRAWNDMSLWGKREAPKRTWNDMALWGKRNGEDKRAWNQMGLWGKRDGDRKAWNDMSLWGKRGADKKAWNDMSLWGKRDAEKRSWNDVALWGKRHVMSDWNRKRTWNDMNLWGRRKRTSGDKKTWNDMALWGKRDQN*

>allatostatinB_Nemertea_Cerebratulus_spec_TRINITY_DN33662_c3_g1_i1.p2

WGKRNADRRAWNEMGMWGKRNWNGMSMWGKRSDDNSKIIDALIADKMAKLSE*

>allatostatinB_Nemertea_Cerebratulus_spec_Contig931_m.196651 3prime_partial

MWGKRAAADKRAWNEMGMWGKRNADRRAWNEMGMWGKRAAADKRAWNEMGMWGKRNADRRAWNEMGMWGKRAAADKRAWNEMGMWGKRNADRRAWN

>allatostatinB_Nemertea_Cerebratulus_spec_TRINITY_DN25947_c1_g1_i1_m.161956 internal

WNEMGMWGKRDGNTEQDKDKRTWNDLKMWGKRTARDVSDVKRNWNGMSMWGKRDADKKAWNEMGMWGKRDGDKKAWNEMGMWGKRDGDKRNWNGMSMWGKRDAEEATDGEKRAWNEMGMWGKRSGDKKAWNEMGM

>allatostatinB_Nemertea_Cerebratulus_spec_TRINITY_DN25947_c1_g1_i2_m.161961 internal

DKKAWNEMGMWGKRSGDKKAWNEMGMWGKRAADKKAWNEMGMWGKRDGDKRAWNEMGMWGKKRGADRRAWNEMGMWGKRDGNTEQDKDKRTWNDLKMWGKRTARDVSDVKRNWNGMSMWGKRDADKKAWNEMGMWGKRDADKKAWNEMGMWGKRDGDKKAWNEMGMWGKRDGNTEQDKDKRTWNDLKMWGKRTARDVSDVKRNWNGMSMWGKRDADKKAWNEMGMWGKRDADKKAWNEMGMWGKRDADKKAW

>allatostatinB_Nemertea_Notospermus_geniculatus_scaffold1654.g28498.t1.p1

LKSSRRVLFSFLWLVILVIRRVEKMACKVHLLGVVLLGLYAVSLAENAQNGPSGKLAETLDNEKAKRDNAEENKLISSDSLDKRQDSVRWTGKIPWDKRAWNEMGMWGKRNGDKKAWNEMGMWGKRNGDKKAWNEMGMWGKRNGDKKAWNEMGIQTESPSPSHGSTTSSTGGGSTAQASPSASGQSKAKIDVLLKATGDAPIMKKKKWAVDPTKKVSWIVEFIKKYIKCEPSESLFLYVNQSFCPLPDTEIGSVYDCFGSDGKLILHYCRTQAWG*

## Allatostatin C

>Allatostatin_C_spiral_Brachiopoda_Glottidia_pyramidata_comp24291_c0_seq1.p3

MNTRATNCCFLGRQDYGFALCVFLSCLVALASAAPVSTEDREPEYGTVIEVTEEDLQPASTVVQVPEIYTSGRQAYLNNFK

>Allatostatin_C_spiral_Brachiopoda_Lingula_anatina_comp116007_c0_seq1.p1

MSCSGRRCVFLRRQDWALALCAVVMSSMVLLARAAPATVETEPGYGSVIEVTDEEFRPAGTAVQVPDYFTSGRQAYLNNFKERALVASKIRELEKEIDELDKLGSKLADQISAVQRKRANLQFKKRQRIACQVWIVSCFGK

>Allatostatin_C_spiral_Brachiopoda_Novocrania_anomala_Nano.rna.tri2.30599.1.p1

MGEMQAIFLVLLASVLCSCAAVPFSRLPTEDGNVSAEQKVSEADVDTLKNKILVASYVNNIENELHKLRLLEDRLADDLQKLQVRRQKLVVNRKRAYPCLINIMSCYTL

>Allatostatin_C_spiral_Nemertea_Cephalothrix_hongkongiensis_comp122437_c0_seq1_m.98909

MDSLKCFAFTILLCGMMCFTHALNGVQDTIEDSRNSKLLLIKNMFEDALRRHMAKQALRDNLVLKRTNTHHVPCMFNIVACYGRKRK

>Allatostatin_C_spiral_Nemertea_Tubulanus_polymorphus_comp34514_c0_seq1_m.40979

MDSKASTIVFAFVLGFVCLSSAFGDEAEVSEKFPLLIRKMYEDAYRRQMDRRLDEGFEKRQSASSHHVPCFMNLAACYGRKRRSTQN

>Allatostatin_C_spiral_Nemertea_lineusLongissimus.8911

MDSRRFTLAVVLILGMVCGSLGRAWLNDEGTRGERLPQLLKEWLDDSYLRQVAAQQELKRDEGVSKRQKSVDRQCLVNLVACYGKRRRR

>Allatostatin_C_spiral_Nemertea_Cerebratulus_spec_TRINITY_DN35200_c0_g1_i1_m.92427

MDSRRFTLAVVLILGMVCGSLGRAWQNEEGARGERLPQLLKEWLDDTYMRQVAAQQDKRVNEGVNKRQKSVDRQCLVNLVACYGKRRRR

>Allatostatin_C_spiral_Nemertea_Lineus_lacteus_comp23159_c0_seq1_m.59302

MDSRRFTLAVVLILGMVCGSLGRAWLNDEGARGERLPQLLKEWLDDSYLRQVAAQQELKRDEGVSKRQKSVDRQCLVNLVACYGKRRRR

>Allatostatin_C_spiral_Nemertea_Lineus_ruber_Lvir.rna.tri.36938.1.p1

MDSRRFTLAVVLILGMVCGSLGRAWLNDEGAREERLPQLLKEWLDDSYLRQVAAQQELKRDEGVSKRQKSVDRQCLVNLVACYGKRRRR*

## Allatostatin C2 (Nemerteans only)

>Allatostatin_C2_nem_Nemertea_Riseriellus_occultus_TRINITY_DN3994_c0_g1_i1.p2

PGKMLLTLLDAAIEGLRERGDDGNAVTNLVKKRDYVRDCFFHAVSCYGRK*

>Allatostatin_C2_nem_Nemertea_Cerebratulus_marginatus_comp456841_c0_seq1_m.121536

CQKGEKHEDTGRMLLTLLDAAIDGLRERGGEGNEVNNLMKKRDYVRDCFFHAVSCYGRK

>Allatostatin_C2_nem_Nemertea_Malacobdella_grossa_comp266207_c0_seq1_m.158978

EVLLIIGWGGGGVQSKTLVRRDHDVSSRTAQDVNDAYLGKLLVQLIDNAIDDMRRQGVPEHQLRTTVRKRDYVRDCFFHAVSCYGK

>Allatostatin_C2_nem_Nemertea_Notospermus_geniculatus_g32019.t1

MRSEQCLLIVLFAFLAICCLSQARGIQKRSEGAGFSNQVATKEEHEATGRMLLTLLDAAIEGLREQGGSGKDLSNMMRKRDYVRDCFFHAVSCYGRK

>Allatostatin_C2_nem_Nemertea_Cerebratulus_spec_TRINITY_DN24391_c0_g1_i1_m.170402

MKHSEQYLWIMLFAFLAVCSIAHARGIRKRSEGSDFNNKVATKEEHEETGRMLLTLLDAAIDGLRERGGEGNDVTNLMKKRDYVRDCFFHAVSCYGRK

>Allatostatin_C2_nem_Nemertea_Lineus_lacteus_comp24706_c0_seq3_m.75737

MLRSEQYVWVVLFAFLAVCSISQARGIQKRSEGSSFTNQVATKEEHEATGRMLLTLLDAAIEGLRERGDDGNAVTNLVKKRDYVRDCFFHAVSCYGRK

>Allatostatin_C2_nem_Nemertea_Lineus_longissimus_c33290_g1_i1_m.27559

MLRSEQYLWIVLFAFLAVCSISQARGIQKRSEGSSFTNQVATKEEHEATGRMLLRLLDAAIEGLREQGDDGNAVTNLVKKRDYVRDCFFHAVSCYGRK

>Allatostatin_C2_nem_Nemertea_Lineus_ruber_Lvir.rna.tri.59799.1_m.104267

MMRSEQYVWILLFAFLAICSISQARGIQKRSDGSSFTNQVATKEEHEATGRMLLTLLDAAIDGLRERGDDGNAVTNLVKKRDYVRDCFFHAVSCYGRK

>Allatostatin_C2_nem_Nemertea_Nipponemertes_spec_TRINITY_DN45725_c0_g1_i1_m.64982

MLSSRVFVMLVLAFIGLSQAKSVAKRSYPSVAPDRDYYLGKLIVGLIDSAIDSMRDQGMGDHEIQSTMRKRDYVRDCFFHAISCYGK

## Allatotropin

>allatotropin_Phoronida_Phoronopsis_harmeri_c106860_g1_i1.p1

VFLVLVISIESIPISDYFNDGILQEKRGFRKGPHFGHGYGKRTSNADNNVELLNAEPRITSIDNGALAEVLVKFPAMTKILVADFIDKNGDGSVSQEEFQSMFKQSKGQSNERLVNRSRLFKVRR

>allatotropin_Phoronida_Phoronis_psammophila_comp70615_c0_seq1.p1

MQRRMQTITISLVFLVLVISVESLPISHYYTSAMLADKRGFRSGPKYSHGYGKRTSDIGFIDDKNESSSDTSIENGALADILVNFPSMTRVIVNDLIDMNGDGQVTEKEFRTVFYRTGSRDQHRTVSRERMFGDRQ

>allatotropin_Phoronida_Phoronis_ijimai_(P._vancouverensis)_comp93194_c0_seq1.p1

MQRRMKTITISLLYLVLVISVESLPLSQLYGSPFLQDKRFDPWSRARFSHGNGKRAGDTATAKDLHSKIRATMSIDNGAFSEILQKFPSLSKFIVDELIDSNGDGEVGAEEFRSILNLHHEKANSRTEKSGGSLFRRRR

>allatotropin_Phoronida_Phoronis_australis_TRINITY_DN291702_c1_g2_i1.p1

MQRRMKTLTISLLYLVFIISVESLPLSQLFRSTFSEDKRIKAMKGTTWFGYGYGKRSDHTAAVDELHSKTRESASIANSAFSEILQKFPSVSMFIVDELIDINGDGQVDAEELRSILNPSHEHPNRRTEKSDGSLFKRRR

>allatotropin_Brachiopoda_Glottidia_pyramidata_comp46982_c0_seq1.p2

MFTIAMHKAIFTFLVFLGLVSLLNAFPLALQRDARAGFRSHEPGYGHNFGKRKAWLETIRDRLLNTRENESPLMTNEELADLLNESSQIADAFVDNIVDINRDGVVTRYELRSKRPMRQL

>allatotropin_Brachiopoda_Lingula_anatina_comp139557_c0_seq2.p1

MFTIAMHKGILTFLVFVGLVSLLDAFPAALQRDARAGFRSHDPGYGHNFGKRKAWLETMRNRLLNTRESESPLMTNEELADLLNESSQIADVFVDRIVDTNRDGVVTRQELRSKHLISQL

>allatotropin_Brachiopoda_Terebratalia_transversa_Ttra.rna.tri.15880.1.p1

MGLLNSAVKFYVLVLVCCSMEVSCAPVPDKDPIPGDNDITKDSSEMYPYKSLLNKEKYLQNYISYPGNELAALKRGLNKGSANRFSHGYGKRRSWSKSFMDFGKKYYPTNTAERHSLNVWKLREYLRKNPKLFDIFLRSIL

>allatotropin_Brachiopoda_Laqueus_californicus_comp240727_c0_seq1.p1

MASLNSAAALLWCIVVLLCCIMEVRGMALRGKDSTLETQDGDFTDNLTGDLSEMYPSTNDDQEGYLQNDIFYPGTNESSALKRALNRGSANRFSHGYGKRQPYSFMDLAKKNSQSRPPLNVSTFREYLQKNPKLFAIFVRSMF

>allatotropin_Brachiopoda_Hemithris_psittacea_comp24095_c0_seq1.p1

MQLYRFVSLLLIAAVFVMQGTGQPLYCRRHQEIPNRPYNNVGEQFRPSNFGEKNYYVSVQRRGGFRSGMANKFSHGYGKRFWQGESLLKKLQAIASRDRQPRAIPVTEMSKLLCNSPLLSRILRKFVDVNDDGFVDRAEMLRMMDGEN

>allatotropin_Nemertea_Cerebratulus_marginatus_comp987854_c0_seq1_m.135925

MKITLYIFCMVLLVVTIRASPSLRRSKRSFKDEIDFGHGFGKRTGQFARLLKHSKEDTGDRPLMSNSDLAKLIAESPELA

>allatotropin_Nemertea_Nipponemertes_spec_TRINITY_DN26865_c0_g2_i1_m.52659

MKYLLYLSVLTLAFSYVHSRPGPLAQRTKRGFKDEIGMGHGFGKRTDQSQNTIERYFKPRPQPVDERRVLTNAQLADLVLE

>allatotropin_Nemertea_Baseodiscus_unicolor_TRINITY_DN107803_c6_g5_i1_m.42141

MRITLCIFCLVLVMVTVQTSPSIHRSKRSFKDEINFGHGFGKRNDGDQIENLLSKQDRDLGDRPLLSNSQLAKLIMTSLDLSEALVAKFV

>allatotropin_Nemertea_Lineus_ruber_Lvir.rna.tri.31157.1_m.60789

MKITLYIFCMAFVVVTIRALPSLRRSKRSFKDEIDFGHGFGKRTDHIGNLLKHHTDTSDRPLLSNSQLAKLIAESRELSEALVAKFVDISDDGYVSKSELFPEGVNL

>allatotropin_Nemertea_Notospermus_geniculatus_g38810.t1

MKITLYIFCMVLVVVTVRGSPSFRRSKRSFKDEIDFGHGFGKRADQLSKFLKHETDISDRPLLSNSDLAKLIEKSPELSRAIVAKFVDRNSDGYVSKRELFPEDVNL

>allatotropin_Nemertea_Cerebratulus_spec_TRINITY_DN36375_c2_g1_i2_m.122315

MKITLYIFCMVLLVVTIRASPSLRRSKRGFRDEIDFGHGFGKRTGQLASLLKHNTEDIGDRPLLSNTDLAKLIAESPELSEALVAKFVDSNGDGYVSKNELFPEGVNL

>allatotropin_Nemertea_Lineus_lacteus_comp24422_c1_seq1_m.72210

MKITLYIFCMAFVVVTIRASPSLRRSKRSFKDEIDFGHGFGKRTDHIGNILKQHTLSDTSDRPLLSNSELAKIIAESRELSEALVAKFVDINGDGYVSKSELFPEGVNL

>allatotropin_Nemertea_Paranemertes_peregrina_comp22956_c0_seq1_m.22909

MEMKLAFGVSVIILLCVHVRPSHSAAVHQRLKRGFKDNIAMGHGFGKRTESASDLSAMQSFLSQKRRMVSNQELSALMRKTPGLSLAIVAKFVDKNGDGYVSGRELFPNGRDE

>allatotropin_Nemertea_Riseriellus_occultus_TRINITY_DN42216_c0_g1_i3_m.5154

MKITLYIFCMAFVVVTVRASPSLRRSKRSFKDEIDFGHGFGKRTDHLGNLLKHHTDTSDRPLLSNKELAKLIAESRELSEALVAKFVDINDDGYVSKSELFPEGVNL

>allatotropin_Nemertea_Riseriellus_occultus_TRINITY_DN42216_c0_g1_i1_m.5152

MSVNSRLNITLYIFCMAFVVVTVRASPSLRRSKRSFKDEIDFGHGFGKRTDHLGNLLKHHTDTSDRPLLSNKELAKLIAESRELSEALVAKFVDINDDGYVSKSELFPEGVNL

>allatotropin_Nemertea_Tubulanus_polymorphus_comp37492_c0_seq1_m.63473

MMKISICIFCFVLLVVTINSYPAINIDTRGFKNEERFGHGFGKRTQSSNTDDLDSFLEKQDMSRLLMTNTRLASEIRSRPSLAEVIIGKFVDQNHDGYISEAELFPERTDEPGI

>allatotropin_Nemertea_Lineus_longissimus_c33006_g1_i5.p1

MKITLYIFCMAFVVVTIRASPSLRRSKRSFKDEIDFGHGFGKRTDHLGNLLKHHTLSDTSDRPLLSNSELAKVIAESRELSEALVAKFVDINGDGYVSKKELFPEGVNL*

>allatotropin_Nemertea_Cephalothrix_hongkongiensis_comp33035_c0_seq1.p1 366 1 (shows similarity to allatostatin A)

MKVSISILCITLTIVIASSQAFSIGLGQKRGFKNHASFSHGFGKRTDKNSIL

## AxxxDF

>ASWLDF_Brachiopoda_Glottidia_pyramidata_comp38042_c0_seq3.p1

FNDFKRAKFNDFKRAKFNDFKRAKFNDFKRAKFNDFKRAKFNDFKREMGEYDVLPMGYQDLGRDEKRAKFFDFKRIPGSLYVDPYEEEMKRAKFNDFKRAKFNDFKRAKFNDFK

>ASWLDF_Brachiopoda_Lingula_anatina_comp142736_c4_seq14.p1

MAAIMKLHLGALILAVLVMTCYTVDTDKAETEVKAPLKKRDITLESDIDIPDDEYKRAKFVDFKREPYYGFENPRRYLDDSGDTYDSEKRAKFNDFKRAKFNDFKRAKFNDFKRAKFNDFKRAKVNDFKRAKFNDFK

>ASWLDF_Brachiopoda_Lingula_anatina_comp142736_c4_seq11.p1

MDKALVLDVPRIQIMKLHLGALILAVLVMTCYTVDTDKAETEVKAPLKKRDITLESDIDIPDDEYKRAKFVDFKREPYYGFENPRRYLDDSGDTYDSEKRAKFNDFKRAKFNDFKRAKFNDFKRAKFNDFKRAKVNDFKRAKFNDFK

>ASWLDF_Brachiopoda_Lingula_anatina_comp142736_c4_seq1.p1

MAAIMKLHLGALILAVLVMTCYTVDTDKAETEVKAPLKKRDITLESDIDIPDDEYKRAKFVDFKREPYYGFENPRRYLDDSGDTYDSEKRAKFNDFKRAKFNDFKRAKFNDFKREMGGYGEPPYMGYHDLGLEEKRAKFFDFKRVPGTFLADPYEEDMKRAKFNDFKRAKFNDFKRAKFNDFK

>ASWLDF_Nemertea_Cephalothrix_hongkongiensis_comp14762_c0_seq1.p1

ENELLNNKRASWIDTKRDLDTKRASWIDTKRASWIDTKRGEPDKQPWFGDKQTSWDDKNRNTDFDNIKRASWIDTKRDGLDDNKRASWIDTKRASWIDTKRASWIDTKRDPGNKRASWIDTKRASWIDTKR

>ASWLDF_Nemertea_Baseodiscus_unicolor_TRINITY_DN102048_c0_g2_i2.p1

PSRLDLTSEERPRLKSNQLDFKRGSLPGDDKRATWHDFKRDHSASSDENRKRATWLDFKRTLGTFVGSNHGMKRDQDNHMDEETIKVIRDLLIKMPPELEPSTGKMIWGEGKQHTQTKVV*

>ASWLDF_Nemertea_Baseodiscus_unicolor_TRINITY_DN102048_c0_g2_i1.p1

DVKSSLDGSHHEGIKKASWLDFKRGSLPGDDKRATWHDFKRDHSASSDENRKRATWLDFKRTLGTFVGSNHGMKRDQDNHMDEETIKVIRDLLIKMPPELEPSTGKMIWGEGKQHTQTKVV*

>ASWLDF_Nemertea_Baseodiscus_unicolor_TRINITY_DN102048_c0_g5_i1.p1

MRLHLNWLLVATVLSCIYASAVPVDQREDEFDKSEERLLTEKSVSDETTEEDDGNDIDLKRASWLDFKRNGGQTYNDGAAKRASWLDFKRNFANHFGNGDKRASWLDFKRNMDNRQLGADDKRASWLDFKRSPFDGMSN

>ASWLDF_Nemertea_Baseodiscus_unicolor_TRINITY_DN102048_c0_g5_i2.p1

QNNILSLASSVEVGVGCVWAFRLLDYPVKLYLLLETIPAAVPYGQREDEFDKSEERLLTEKSVSDETTEEDDGNDIDLKRASWLDFKRNGGQTYNDGAAKRASWLDFKRNFANHFGNGDKRASWLDFKRNMDNRQLGADDKRASWLDFKRSPFDGMSN

>ASWLDF_Nemertea_Cerebratulus_spec_TRINITY_DN17276_c0_g1_i1.p1

MTNNIKISRNLLLLVAGLSYLYASATAEDNPFELNGERLSESHDEHKLTDVNSAQKRASWLDFKRDGSEEYEGAPNHDPAKRASWLDFRRDGGNKRASWLDFKRGDNSDLNAWLEGKRASWLDFKKRA

>ASWLDF_Nemertea_Cerebratulus_spec_TRINITY_DN17276_c0_g2_i1.p1

RASWLDFKRGDNSDLNAWLEGKRASWLDFKKRASWLDFKRNHGDKRASWLDMKRASWLDMKRNGDKRASWLDMKRDSDKRASWLDFKRSGDSSLADLKRSIGSWIEAKQAHKRGNDDQPMDPKILEVIREMLLKDNLELTPSERKAIFDKYGKALVNKYGKEIEPYLQE*

>ASWLDF_Nemertea_Riseriellus_occultus_Contig14208.p1

TRNGAYPDDKRASWLDFKRALGSWIEAKQTDKRGKGNPMDPETIKVIREMLLKDKTELTPSMRENLLSTFEKETQK*

>ASWLDF_Nemertea_Riseriellus_occultus_TRINITY_DN75509_c0_g1_i1.p1

VASEDKAFEVNGGRISDENKLTELDSGQKRASWLDFKRNGGQKYDESDNADQDEAKRASWLDFKRDDAAKKRASWLDFKRGNTGDLKPWLENKRASWLDFKKRASWLDFKRTHENTDASSDKRASWLDFKRGADKKASWLDFKRNTDKRASWLDFKRENDKRASWLDFKRNGASPDDKRASWL

>ASWLDF_Nemertea_Lineus_lacteus_comp21111_c0_seq1_m.44765 complete

MRKAQNVLLLVAGLFCLCAKVASENKAFEVNGDRISDSVADENKLSELDSGQKRASWLDFKRNGGQEYDESDDANQDEAKRASWLDFKRDDAAKKRASWLDFKRGNTGDLKPWLENKRASWLDFKKRASWLDFKRTHGNTDASDDKRASWLDFKRGADKKASWLDFKRNTDKKASWLDFKRDGDKRASWLDFKRSGLSSDKRASWLDFKRALGSWIEAKQTHKRGNDNPMDPETIKVIREMLLKDKSELTPSMRQNLLAAFAKDAHK*

>ASWLDF_Nemertea_Lineus_longissimus_c40125_g1_i1_m.57346 complete

MRKSQNVLLLFAGLFCLYASVASENKAFEVNGDRISDSVADESKLTEINSGQKRASWLDFKRNGGREYDESDNANQDEAKRASWLDFKRDDAAKKRASWLDFKRGNTGDLKPWLENKRASWLDFKKRASWLDFKRTHGNTDANNDKRASWLDFKRAVDKKASWLDFKRNTDKKASWLDFKRDNDKRASWLDFKRNGASPDDKRASWLDFKRALGSWIEAKQTHKRGNDNPMDPETIKVIREMLLKDKSELTPSMRENLLSAFAKDTQK*

>ASWLDF_Nemertea_Lineus_ruber_Lvir.rna.tri.33242.1_m.64400 complete

MRKSQNVLLLVAGLFCLYASVASEDKTFDVNGGRISDSLNDENKLTELDSGQKKASWLDFKRSGGQEYDESDNANQDETKRASWLDFKRDDAVKKRASWLDFKRGNSGDLKPWLENKRASWLDFKKRASWLDFKRTQENTDASSDKRASWLDFKRGAEKKASWLDFKRNTDKKASWLDFKRDNDKRASWLDFKRNGASPDDKRASWLDFKRALGSWIEAKQTHKRGGNNNPMDAETIKVIREMLLKDKPELTPSMRANLLSAFEKETQK*

>ASWLDF_Nemertea_Notospermus_geniculatus_g22050.t1

MRVSRTFLLLVVGISCLYTRVASDDKTFEVNGDRLSESVADDDEKLSGLDSGQKRASWLDFKRSGGQEYDGSDDDEDEAKRASWLDFKRAGADKRASWLDFKRGGNSGLKAWLDNKRASWLDFKKRASWLDFKRSHGDLSDDKRASWLDMKRDMDKRASWLDFKRSNDKRASWLDFKRANDKRASWLDMKRNGVSQDDQKRASWLDMKRDGLKRASWLDMKRNGGSADEQKRASCYQGDVIERQTRVKAVHAQGTFGESRQGDQKLNGPVQCR

>ASWLDF_Nemertea_Notospermus_geniculatus_g16200.t1

MRVSRTFLLLVVGISCLYTRVASDDKTFEVNGDRLSESVADDDEKLSGLDSGQKRASWLDFKRSGGQEYDGSDDDEDEAKRASWLDFKRAGADKRASWLDFKRGGNSGLKAWLDNKRASWLDFKKRASWLDFKRSHGDLSDDKRASWLDMKRDMDKRASWLDFKRSNDKRASWLDFKRANDKRASWLDMKRNGVSQDDQKRASWLDMKRDSSDGLKRASWLDMKRNGGSADEQKRASWLDMKRALGSWIEAREKQKRGGADNTMDADTIKVIREMLLKDKPELKPSMRKELLESLDKATKN

## Calcitonin

>Calcitonin_lopho_Phoronida_Phoronis_ijimai_(P._vancouverensis)_comp91794_c0_seq1.p1

MITLLNMFAGLIFLLSLTYTQAEPNTEEMLRKLQELSETRSALSKLRNSIDGMSTDYAKAHKRWGQCALGGLPGHTCRLPQLERALDANDWLNGPAYTLRRKRRSVDNSNEAKSRFTRVLDDLSSKKRALEHLRRLINSMHDNVIQEQKRHCLNLGGHCSIEGAANLHDTYRYYNSALSPGRRDLDMLNKLLAR

>Calcitonin_lopho_Phoronida_Phoronis_ijimai_(P._vancouverensis)_comp848988_c0_seq1.p1

STDYAKAQKRTCAIGGGMSHHCQLDLLNQQKQAREWLASPKSPGKRSLGFIEIEDVSSVDRVLDDLS

>Calcitonin_lopho_Phoronida_Phoronis_psammophila_comp16942_c0_seq1.p1

MRNPLAMISSFIFLLTLQEILGELNTDDVLRKLQELSETRSSLDKLRKSLDDITTDYSSAQKRWGQCALGGLPGHTCRLPQLERSIEGSDWLHGPTYNLRRKRRSLESENMRKKRFVRILDDLSSKQQALEQLRRLIDSMHSKV

>Calcitonin_lopho_Phoronida_Phoronis_australis_TRINITY_DN234721_c1_g1_i1.p1

MIKPSNMFTGLVCLLYLVYTHAEPATEDMLRKLQELSETRSALSKLQNSIDGMSADYEKAHKRWGQCALGGLPGHTCRLPQLERALDANDWLNGPAHSLRRKRRSTDNSNEANSRLTRVLDDLSSKKRALEHLRRLINSMHNNVIQEQKRHCLNLGGHCSIEGAANLHDTYRYFNSALSPGRRDLDMLKKLLTR

>Calcitonin_lopho_Phoronida_Phoronis_australis_TRINITY_DN234721_c1_g2_i1.p1

MIKPSNMFTGLVCLLYLVYTHAEPATEDMLRKLQELSETRSALSKLQNSIDGMSADYEKAQKRTCAIGGGMSHHCQLDLLHQQKQAKEWLASPKSPGKRSIDFINIEDVSSVDRVLDDLS

>Calcitonin_lopho_Brachiopoda_Hemithris_psittacea_comp21945_c0_seq1.p1

MVPSIIILQVTLCVLVAHVVKASDLETHDREIRSIEDTRAVIDILKKTIQAIDGEYKDVQQRSCAVGGGMSYHCDISDFDRKMKTLSFLRGGPGKREKTAKVDRNPGSSETKLKRSRRSVIGARVPFSKHWAGLHRHLRSVVEKRYSLHLVRQILDQMDDEMNELHVQRRSCFNLGTKCDIEGAHDYARQRSFLGSAYSPGKRSLYDAYDSTMYYR*

>Calcitonin_lopho_Brachiopoda_Novocrania_anomala_Nano.rna.tri2.92560.1.p1

MEFYHYLLFLVLWICKACAYRTDDLRSLREIDTTRHEYAALSKVLDELDSDLQLAQKRSCYVNGGMSHFCLLSNLDDKLRQKEYLSSGRGPGKRFVRSVDNEAPTKSKKACNVNGGMSPFCLLEELNNKLNTQRFLESGASPGKRGDGATEEKERKKRSLLGVGTRDLEMVGYMPEKALENIARKRSGISVMRRMITRMDQDMMVERKRACLNLGSMCSAEDAHARAGMWDYMNSPASPGRKRRSITEDDIEAM

>Calcitonin_lopho_Brachiopoda_Novocrania_anomala_comp74922_c0_seq1.p1

MEFYHYLLFLVLWICKACAYRTDDLRSLREIDTTRHEYAALSKVLDELDSDLQLAHKRSCGRIRGLSPLCLSPDVADKMRQYEYLNSGMSPGKRSCYVNGGMSHFCLLSNLDDKLRQKEYLSSGRGPGKRFVRSVDNEAPTKSKKACNVNGGMSPFCLLEELNNKLNTQRFLESGASPGKRGDGATEEKERKKRSLLGVGTRDLEMVGYMPEKALENIARKRSGISVMRRMITRMDQDMMVERKRACLNLGSMCSAEDAHARAGMWDYMNSPASPGRKRRSITEDDIEAM

>Calcitonin_lopho_Brachiopoda_Lingula_anatina_comp157085_c0_seq1.p1

MLPEWRSLAHWGMTIVFFHILVLNLTGASKLEALRSIRNLKRQRSQFSEMKRVLEEIDADLLKQQKRTCALNGGMSTFCFGENIHDKMNAYEYLGSHKSPGRRTIRSTGDLHAWRKRTCSVNGGMNPFCLISDLDAKLNAQEKLGSKFGPGKKRDSKPKSRKRRSIYWTGPCPSGTGTCSYPLVPASQPSPSIGNTGNMYGVNTGLGKPNFGGFGEPFKRAQSNSNFYLTDENPYAFYKRGNTADMDRFYFNDDESMSNFYKRDGEDGEFDMFVQGEDVGLGEDSESVLRSIARKRSGISSLKRLLSNLDARLHKQQKRACLMNLGHSCDVESAAAAADTWKYLNSVHSPGRRRRDTRAANKDRNTDEADDKREVTDDNPADEEKREEETAASDESKANKREEGVKHKGGSFYSFYPSRRSEDWQSSDGDDASRFYSKFYKLVPFY

>Calcitonin_lopho_Nemertea_Malacobdella_grossa_comp21336_c0_seq1_m.17484

MLPSRFVMLLVASHLVMISTIMAARIPTNRQRKRSDQKELSLSKNDVMALERILTDVDRVLGSVHNMQGNEDLARKRLGLKLLARYLAQKEQKLEQISYRKRNCRVNLGGHCATETVADMVDLYHFLSSADSPGRRRRRHATAHVTSDVGKMAAN

>Calcitonin_lopho_Nemertea_Baseodiscus_unicolor_TRINITY_DN96538_c0_g1_i8_m.186549

MHGDYTLKLPYLLLATVCVVASLAFEDARMQEALRQHKDFTVKRTDLAKLKEALNEIDQDLENVQKRWSDSCSVGAGMPGYRCRLDQLEDNEMYKDFLDSNDKSPGKRSGSDKSVEEIVQDLARKRVALQLIKNLLEEKDQELGTAQKRNCRVNLGGHCSTETASAMADLYHYLNSAESPGRKKRDTAGQILKKALRK

>Calcitonin_lopho_Nemertea_Cerebratulus_marginatus_comp62514_c1_seq3_m.106936

RSSLGVKRSLGVRSGAEILKDLDRKRHALEFLRSLLEEAGQQNQQKVKRNCLVNLGGHCATETAAAIADMYHYLNSAESPGRKKRSASKLLRAALKKD

>Calcitonin_lopho_Nemertea_Cerebratulus_marginatus_comp1844194_c0_seq1_m.158451

QQNQQKVKKRDCNVNLGGFCSTEHIASIVDFLNYLNDPASPGKRKRSASKLLRAALKNQVGK

>Calcitonin_lopho_Nemertea_Cephalothrix_hongkongiensis_comp8366_c0_seq1_m.4067

MPGYNCRLDQLGENADMLDYLDSNLAPGKRAVLLRRPGQKRWSESCSIGAGMPGYNCRLDQLGENADMLEFLDSNLAPGKRSLEKMPKQHPVKVIKDLSRKRLGLRFLESLLNDKDGELEAKQKRNCRVNLGGHCQAETAFSLANQFRFLNSADSPGRRRRAADLVLKNSK

>Calcitonin_lopho_Nemertea_Tubulanus_polymorphus_comp32409_c0_seq3.p1

MLMNWASWQRTVFICAAIMIAIVTADYESPDFSDGAREHWRLAVKRRDINSLKGIVQDIDTDLAHIQKRWDPSCSIGAGMPGYDCRLDQINGKKEKS

>Calcitonin_lopho_Nemertea_Tubulanus_polymorphus_comp32409_c0_seq2.p1

MNWASWQRTVFICAAIMIAIVTADYESPDFSDGAREHWRLAVKRRDINSLKGIVQDIDTDLAHIQYSDCMKDDGDKSECETPNMLVVDEISGRRRGGDLSRAIQDLTRKRVGLQFLKKLLNEQENSLMVQQKRNCRVNLGGHCQAETAYAMAKQIRYLNSPDSPGRKRRSLANLVNKRGGSEF*

>Calcitonin_lopho_Nemertea_Tubulanus_polymorphus_comp32409_c0_seq1.p1

MLMNWASWQRTVFICAAIMIAIVTADYESPDFSDGAREHWRLAVKRRDINSLKGIVQDIDTDLAHIQKRWDPSCSIGAGMPGYDCRLDQINGNQEIVDWLGNDALSPGKRSVKVAGKPDSRYANTLQAIQDLTRKRVGLQFLKKLLNEQENSLMVQQKRNCRVNLGGHCQAETAYAMAKQIRYLNSPDSPGRKRRSLANLVNKRGGSEF*

>Calcitonin_lopho_Nemertea_Cerebratulus_spec_TRINITY_DN35478_c2_g1_i2_m.90336

MHQELVIKLRYVLLAGLFAVTFAFQDSRISDALRQHKDLVAKRSEIGRVKDVLNEIDEDLENVQSKICMLSGSTDQACSGYDVKRSDASAADLFSQEDGNRIMKDLARKRAALEFLKRLLESELKQNGQVIKRNCLVNLGGHCATETAAAIADMYHYLNSAESPGRKRRSASKLLRAALKKN

>Calcitonin_lopho_Nemertea_Lineus_lacteus_comp24808_c0_seq4_m.76860

MQDKFVIKLRYVLLAGLFAVTLAFEDSRLADALRQHKELTAKRSEIGNVKAVLREIDDDLGNVQKRWNSRCSIGAGMPAYDCRIDQVTENEMAKDWLDNSDYSPGKRSSLGGKSANEILKDLARKRAALQFLRKLLMEENERNGQVIKRNCLVNLGGHCATETAAAIADMYHYLNSAESPGRKKRSASKLLRKALRKN

>Calcitonin_lopho_Nemertea_Lineus_lacteus_comp24808_c0_seq1_m.76851

MQDKFVIKLRYVLLAGLFAVTLAFEDSRLADALRQHKELTAKRSEIGNVKAVLREIDDDLGNVQSKICMLSGSNDPACSGYAVKRSDADAADMFSQEDGDRILKDLARKRAALQFLRKLLMEENERNGQVIKRNCLVNLGGHCATETAAAIADMYHYLNSAESPGRKKRSASKLLRKALRKN

>Calcitonin_lopho_Nemertea_Lineus_longissimus_c44107_g1_i1.p1

MQQEFVIKLRYVLLAGLFAVTLAFEDSRIADALRQHKELTAKRSEIGNVKAVLSEIDDDLENVQSKICMLSGSTDPACSGYAVKRSDADAADMFSQKDGNRILKDLARKRAALQFLKKLLMDENERNGQVIKRNCLVNLGGHCATETAAAIADMYHYLNSAESPGRKKRSASNLLRKALRKN*

>Calcitonin_lopho_Nemertea_Lineus_ruber_Lvir.rna.tri.38477.1_m.73563

MRQEFVIKLRYVLLAGLFAVTLAFEDTRIADALRQHKELTAKRSEIGNVKAVLSEIDEDLENVQSKICMLSGSTDQACSGYAVKRSDNAGAVDMFSQEDGNRILKDLARKRAALQFLKKLLTEENERNGHVIKRNCLVNLGGHCATETAAAIADMYHYLNSAESPGRKKRSVSNLMRRALRKN

## CCAP

>CCAP_Nemertea_Lineus_ruber_Lvir.rna.tri.51452.1_m.93180

MLAYGSVCYILLLTTLISGQESNSQNDLQTDKASGSIFPYGSADNELDVFKSLRERLENEKQSRAKRPFCNGYYGCGNGEYGKRSGELKITTSEEPSNEEGLLLKALLSNTGNSQFLEGSGKTQKRSFCNGFYGCANGKRSGMEKVIKRVLKQKTSKMAAKRPFCNAYSGCGNGKKRSVDMENGADGRQGLLRTILNRLRSVQSEGDQQDTSFTF

>CCAP_Nemertea_Cerebratulus_spec_TRINITY_DN64865_c0_g1_i1_m.120280

FAFDNDDGTQTELDVLKSLRERLEKEKQSRSKRPFCNGYYGCGNGGYGKRSEGLKMSKSEPSNEGLLLKALLSNTGSDQFVEGSGNTQKRAFCNGFYGCANGKRSGMENVIK

>CCAP_Nemertea_Lineus_lacteus_comp8101_c0_seq1_m.9589

EKQSRAKRPFCNGYYGCGNGGYGKRSQELKLSKSEEPSSEEGLLLKALLSNTGNNQLVEGSNKKQKRAFCNGFYGCANGKRSGMENVIERVLKQKTSKMAAKRPFCNAYSGCGNGKKRSVAMENGVDGNKGLLRTILSRLRSVESEGNQQEEPFTF

>CCAP_Nemertea_Baseodiscus_unicolor_TRINITY_DN88712_c1_g1_i1_m.224006

LMITIISGQETQHQNVQQKSKTDTDLFDYEKKGSQLDVLNAIKERLENVKEARSKRPFCNAFYGCGNGKRSKNSEGVRQASVNKATSGDPYFSMRPLLSKAPVWDKVPEGYQRNIGDDNEKSVKLNDIIKSLLINRKSRPTEHIVSKRPFCNAFFGCGNGRKRSADRVETDDRTVGSDIGFRKDVFEKLLSRFQSTS

>CCAP_Nemertea_Lineus_longissimus_c48685_g1_i1_m.143748

MLAYGSVCYILLLTTLISGQESNSQNDFQKNKASGSIFPYGSADNDLDVLKSLRERLDKEKQSRAKRPFCNSYYGCGNGGYGKRSEELKISKSEDPSNEQGLLLKALLSNTGNNQFMEGSGKTQKRAFCNGFYGCANGKRSGMENVIQRALKQKTSKMAAKRPFCNAYSGCGNGKKRSVDVENGAEGEKGLLQTILSRLRSVESEGNHQEEPFTF

>CCAP_Nemertea_Notospermus_geniculatus_scaffold27.g1846.t1.p1

LRYQVLFGRMLAYGSVCYILLLTTIISGQENSSQNDQQNDKISGSIFGFNGDDDTQTQLDVLKSLRERLDKVKESRSKRPFCNGYYGCGNGGYGKRSDGLNVQMPKSEPSDRDGFLLNALLGNQGDEQSLEGSGSSQKRAFCNGFYGCSNGKRSGMENVIKRIIKQKTSKMAAKRPFCNVYSGCGNGKKRSFGNGSDGNNGLLQNILSRLRKVSSEDNQRESQFNY*

>CCAP_Nemertea_Tubulanus_polymorphus_comp41061_c1_seq8_m.132554

MYSDVTPADARHTKLSRRFYKTYKMPYCRAYGCFNGSGKKQNKIKTILHCFPKDIDFIRR

>CCAP_Nemertea_Tubulanus_polymorphus_comp41061_c1_seq7.p1

HTKLSRRFYKTYKMPYCRAYGCFNGSGKKQNRGKAILHCFPKDIDYIRRWVINMGRDVGNIEKYCRNVKDDGNGDRFRLCSEHFENNCYEDDLKARLMGTTAKKRLKRDAIPTLFDHKKEKS

>CCAP_Nemertea_Tubulanus_polymorphus_comp41061_c1_seq5.p1

MYSDVTPADARHTKLSRRFYKTYKMPYCRAYGCFNGSGKKQNKIKTILHCFPKDIDYIRRWVINMGRDVGNIEKYCRNVKDDGNGDRFRLCSEHFENNCYEDDLKARLMGTTAKKRLKRDAIPTLFDHKKEKS

>CCAP_Nemertea_Tubulanus_polymorphus_comp41061_c1_seq1.p2

MTKDATLRLTLHTRENCNFFNSANIDSLVLPLPHHKIKQTILQDIIDPYKMPYCRAYGCFNGSGKKQNRGKAILHCFPKDIDYIRRWVINMGRDVGNIEKYCRNVKDDGNGDRFRLCSEHFENNCYEDDLKARLMGTTAKKRLKRDAIPTLFDHKKEKS

## CCWamide

>CCWa_Phoronida_Phoronopsis_harmeri_c131158_g3_i1.p1

MRSLLSITILLVLLQMALCSYEPAEDDSAVYPQDHAVAVDDGEFDEMDKRGNPSLKNYMSKFKCRAYDQFCDGPSVKRPRPWMRCCPKSTCRCNFLGVNCRCGRSKYGR*

>CCWa_Phoronida_Phoronis_ijimai_(P._vancouverensis)_comp81603_c0_seq7.p1

MQTIRVQVLVVVVLSLHIAISWSHQVFDLDESESAALDKLRNAKRMSWGSRMKSCASWNDYCDPWPDERNEDRIARSFTCCDNMICKCNLWVRNCRCKSRIWGR*

>CCWa_Phoronida_Phoronis_ijimai_(P._vancouverensis)_comp81603_c0_seq3.p1

MQTIRVQVLVVVVLSLHIAISWSHQVFDLDESESAALDKLRNAKRAYPNAYKLFMRICRRRNESCDPSGRNIFYRCCVKDSCKCGPFWFNNCRCIPSIFSR*

>CCWa_Phoronida_Phoronis_psammophila_comp71831_c0_seq7.p1

MRMDTKQILVVLVLCLQIAASWSKLFDKEADDSRISSLDKFRTAKRWSWSAQLKSCASWNDWCDPWPPDGSFLDPDSYEYLRAKANTCCDNLICKCSLWVRNCRCKYRLWGR*

>CCWa_Phoronida_Phoronis_psammophila_comp68251_c0_seq1.p1

MRGYLPLILLFVLIQMVLSSIEPDQDETAIYSPEDGEMEGYDDEDEIEKRGKQNFKMFFRKFKCRSYEQFCDPPTMKRRRPWMTCCPGQMCRCNFLGRNCRCGKRKYGR*

>CCWa_Phoronida_Phoronis_psammophila_comp71831_c0_seq1.p1

MRMDTKQILVVLVLCLQIAASWSKLFDKEADDSRISSLDKFRTAKRGIRYDKAVKFFLRVCRRFNDSCDPSGRNIFYRCCAKDSCKCGPFWFTNCRCIPSLFS*

>CCWa_Phoronida_Phoronis_psammophila_comp71831_c0_seq1.p2

MLCFSIIVSHRSVSFSLRWSWSAQLKSCASWNDWCDPWPPDGSFLDPDSYEYLRAKANTCCDNLICKCSLWVRNCRCKYRLWGR*

>CCWa_Phoronida_Phoronis_australis_TRINITY_DN320441_c3_g7_i1.p1

METGTSRVVLALVLSLHIALSWSHLVFDSKENELALNKLRAAKRVVWRYRVKSCASWNDYCDPWPDESSQDRLTRSFTCCDNMICKCNLWAQNCRCKSRIWGR*

>CCWa_Brachiopoda_Hemithris_psittacea_comp292757_c0_seq1.p1

LGVFRAVLVFFHAEDGIRDDQGRSLLDVFSNAKRSNKFFLKWCARWGDYCKPDTRLKYAQCCPGLDCKCGLIWTSSKCTCKGKFLGRR*

>CCWa_Brachiopoda_Novocrania_anomala_Nano.rna.tri2.31825.1.p2

MRAMSVIMLCVLVVLTTMYLSCYQALGDDTNAMLEDEDPRWENEVPDDAMNGFYSVERRRGPNRRKCQSWGYRCIPNGKTSIGRCCSGLDCKCKGWWSNGNCKCMDVFGRK*

>CCWa_Nemertea_Nipponemertes_spec_Contig14358.p1

GEENFETREGYADDNNQLDTYKDAAKRALYNIKCATATCQYRSREPTLRCCPGMRCRCGILWNAGHCKCTSFGYGYGRYW*

>CCWa_Nemertea_Paranemertes_peregrina_comp478181_c0_seq1.p2

AEDGIRDDQGRSLLDVFSNAKRSNKFFLKWCARWGDYCKPDTRLKYAQRCPGLDCKCGLIWTSSKCTCKGKFLGRR*

>CCWa_Nemertea_Paranemertes_peregrina_comp24105_c0_seq1_m.26309

MNKFVTGTLLFLVVVQIVASYPWGEENQEVREGGYDENQLDTYKDAAKRAVYNFKCATATCQYRSREPTLRCCPGMRCRCGILWNAGHCKCTSFGYGYGR

>CCWa_Nemertea_Cephalothrix_hongkongiensis_comp29558_c0_seq1_m.22361

SYGWEDQDDSDDQLDIYRAADKRAMVNLPCATISCIPFSKEISLRCCPGLRCKCGLLWSRGHCRCKPYTYGK

>CCWa_Nemertea_Cephalothrix_hongkongiensis_comp17531_c0_seq1_m.8819

MANQKIIIIQLLAAIFTILLCLSSANYIEPEDFYNDGEGEALSIYKAAAKRSRSRLVRKRCRLATQSCVPTGKAGNKWPDCCPGSKCKCSLWGSCRCREPLFGR

>CCWa_Nemertea_Tubulanus_polymorphus_comp31790_c0_seq4_m.28043

MKVSILKIHILAITFSVVLCSSISMYGDDDDDQESAIDLFQSAVKRYVRRGRKCRIWGQSCDNSSRFFCCKDYYCKCSLWNCRCNPSLFGK

>CCWa_Nemertea_Tubulanus_polymorphus_comp31790_c0_seq1_m.28037

MKVSILKIHILAITFSVVLCSSISMYGDDDDDQESAIDLFQSAVKRRAVSESHSYRRTKNWSRTCKRWNQRCDPWTGYTEMKCCPEPKLSCKCNLWGQNCKCVMKLWGR

>CCWa_Nemertea_Tubulanus_polymorphus_comp35294_c0_seq1.p1

MKVYGMIFAVLMCFLSITICYSWGEAEKRDMSDMSDGDDDDQLDVFQAAAKRNLFQFQKLKCATSQCRPRSRSHSLRCCPGLRCKCGILWNAGQCRCSTNYGYGR*

>CCWa_Nemertea_Baseodiscus_unicolor_TRINITY_DN93972_c0_g1_i3_m.211304

IIVCLVLVCVLQFVSSYPYGDENEYDGDQLEVFRDAAKRAFYNFKCAMSSCRPRSKEPTLRCCPGMRCKCGILWNSGVCKCTNYGYGR

>CCWa_Nemertea_Baseodiscus_unicolor_Contig20254_m.255538

MNHVLTICLVVSISITVVLGSSIPQYDDSDGSYQSVVDTFRAASKRGNPRCRLWGENCDPRGLNNSFICCKKYYCKCGWGNCKCSGTLFG

>CCWa_Nemertea_Baseodiscus_unicolor_TRINITY_DN93972_c0_g1_i2_m.211302

IIVCLVLVCVLQFVSSYPYGDEKAKSSEDKENEYDGDQLEVFRDAAKRAFYNFKCAMSSCRPRSKEPTLRCCPGMRCKCGILWNSGVCKCTNYGYGR

>CCWa_Nemertea_Baseodiscus_unicolor_TRINITY_DN97279_c1_g1_i2.p1

MNHVLTICLVVSISITVVLGSSIPQYDDSDGSYQSVVDTFRAASKRRNYHNNDYAYYYSGRGCKRWNDWCNPWSSDRETKCCPNEKLACKCNFFGSNCRC

>CCWa_Nemertea_Baseodiscus_unicolor_TRINITY_DN90005_c0_g2_i1_m.173135

MTTMVSGFQILVIASLIILGILIGVHGHDTEGEKIITDHKTKGRRNSRRYPCRGIKQFCERTNPNLSCCKGTYCACGITNYCRCASPRD

>CCWa_Nemertea_Notospermus_geniculatus_g41399.t1

MLPNSVVDDSGGTCSQTKKLFEKCTQGEYGDDQLEVFRDAAKRAFYNFKCATSTCRPRSMEPTLRCCPGMRCKCGILWNAGQCKCTNYGYGR

>CCWa_Nemertea_Notospermus_geniculatus_scaffold52.g3053.t1.p1

MKLYIVACLFVVCALHFVASYPYGEEKAKKSADNQEGEYGDDQLEVFRDAANDLETPPERRAFYNFKCATSTCRPRSMEPTLRCCPGMRCKCGILWNAGQCKCTNYGYGR*

>CCWa_Nemertea_Notospermus_geniculatus_scaffold6.g467.t1.p1

MDSRWIYTVLIILLLVNVSVCFVRREDDKRSEEKESATCRKKKEFCELDNPGLTCCTGLMCSCGLYDYCRCSDPMEWNEN*

>CCWa_Nemertea_Cerebratulus_marginatus_comp53129_c0_seq2.p2

KSDDNQESEYGDDQLEVFRDAANHLQTPPERRAFYNFKCATSTCRPRSMEPTLRCCPGMRCKCGILWNAGQCKCTNYGYGR*

>CCWa_Nemertea_Cerebratulus_marginatus_comp53129_c0_seq3_m.46799

KLYIVACLFLVCALQFVASYPYGDESEYGDDQLEVFRDAAKRAFYNFKCATSTCRPRSMEPTLRCCPGMRCKCGILWNAGQCKCTNYGYGR

>CCWa_Nemertea_Cerebratulus_marginatus_comp53129_c0_seq4_m.46803

KLYIVACLFLVCALQFVASYPYGDESEYGDDQLEVFRDAANHLQTPPERRAFYNFKCATSTCRPRSMEPTLRCCPGMRCKCGILWNAGQCKCTNYGYGR

>CCWa_Nemertea_Cerebratulus_marginatus_comp53129_c0_seq1_m.46791

KLYIVACLFLVCALQFVASYPYGDENKDKKSDDNQESEYGDDQLEVFRDAAKRAFYNFKCATSTCRPRSMEPTLRCCPGMRCKCGILWNAGQCKCTNYGYGR

>CCWa_Nemertea_Cerebratulus_marginatus_comp57114_c0_seq2_m.60533

MKTVLTTFVVIASISLVLSSSIPQYDDSEESGFLSELDNYKTASKRKALTRNYYNDYAYYYKRNCKPWNDWCDPWSSNPTRGCCPNERLACKCNFFGSNCRCQRKLWGR

>CCWa_Nemertea_Cerebratulus_marginatus_comp48202_c0_seq1.p2

MDTTRSAWIMLLLGMAILCQTDAWFIKRHQLDDNDLSRQERDTCRQKKDFCENNNPGLACCPFLQCSCGLFDYCRCSDPTEVRH*

>CCWa_Nemertea_Riseriellus_occultus_Contig26947.p2

MVMFVMAAFVFEDAKACGDWREPCWSSGCCDTDCHVCKCNIFGSNCKCESRPFSCWR*

>CCWa_Nemertea_Riseriellus_occultus_TRINITY_DN47939_c0_g11_i1_m.95155

ACLFLVCALHFVASYPYGDENEYGDDQLEVFRDAAKRAFYNFKCATSTCRPRAMEPTLRCCPGMRCKCGILWNAGQCKCTN

>CCWa_Nemertea_Riseriellus_occultus_TRINITY_DN42583_c0_g1_i2.p1

MDTKEQRCLVLLLLVVVLIQGSDAFLPARDTSAGGAFGRYERGACRQKKDFCEKGNPGLTCCTGFVCSCGLYDYCRCSDPTEIM*

>CCWa_Nemertea_Lineus_lacteus_comp12621_c0_seq1_m.16690

MRSAALVLIVVMFVMAAFVVEDTKACVNSGRCDGTSCCDECYKCKCGTPWGTNCTCQKKMFSCWGKR

>CCWa_Nemertea_Lineus_lacteus_comp15643_c0_seq1_m.23386

NYKTATKRQALTRNYYNDYAYYYKRNCKRWNDWCDPWSSNPTRTCCPNERLACKCNFFGSNCRCTRKLWGR

>CCWa_Nemertea_Lineus_lacteus_comp9501_c0_seq1_m.11672

MNTVLTIFVVIASISLVLSSSIPQYDEDDDGSFLNVLDNYKTATKRQNRCRRWGESCDPRRKNGYFICCEKYYCKCSIWGSCRCTGTMFG

>CCWa_Nemertea_Lineus_lacteus_comp23728_c0_seq3_m.64610

MKLYLVACLFLVCALQFVASYPYGDENEFGDDQLEVFRDAAKRAFYNFKCATSTCRPRAMEPTLRCCPGMRCKCGILWNAGQCKCTNYGYGR

>CCWa_Nemertea_Lineus_lacteus_comp23728_c0_seq2_m.64607

MKLYLVACLFLVCALQFVASYPYGDENEFGDDQLEVFRDAANHLQTPPERRAFYNFKCATSTCRPRAMEPTLRCCPGMRCKCGILWNAGQCKCTNYGYGR

>CCWa_Nemertea_Lineus_lacteus_comp23728_c0_seq4_m.64613

MKLYLVACLFLVCALQFVASYPYGDESKAKSKKSGDNQENEFGDDQLEVFRDAAKRAFYNFKCATSTCRPRAMEPTLRCCPGMRCKCGILWNAGQCKCTNYGYGR

>CCWa_Nemertea_Lineus_lacteus_comp23728_c0_seq1_m.64604

MKLYLVACLFLVCALQFVASYPYGDESKAKSKKSGDNQENEFGDDQLEVFRDAANHLQTPPERRAFYNFKCATSTCRPRAMEPTLRCCPGMRCKCGILWNAGQCKCTNYGYGR

>CCWa_Nemertea_Lineus_lacteus_comp15220_c0_seq1_m.22252

MNCNYVPLALAYVLLLCLEAVLTRSTYNNPYGDDYPGEYQPEQLSLPAEYEGQLPPVSRNNLLDIFKSTRKRARNVTKRCYSSRFCCKETADCCPGYTCGVNIWNWSCYECIKKTWGR

>CCWa_Nemertea_Lineus_lacteus_comp16594_c0_seq1.p1

MDSNGKCCLLVVLLAVVFIHASDVFARRDHDTPVDGSIERLERGSCRQKKEFCEKNNPGLTCCAGLLCSCGLYDYCRCSDPTEIM*

>CCWa_Nemertea_Lineus_ruber_Lvir.rna.tri.47634.1.p3

SYKTATKRRDNRCRRWGESCDPRRKNGYFICCEKYYCKCSIWGSCRCTGTMFG*

>CCWa_Nemertea_Lineus_ruber_Lvir.rna.tri.5188.1_m.11731

MKLYIVACLFLVCALQFVASYPYGDENGEYGDDQLEVFRDAAKRAFYNFKCATSTCRPRAMEPTLRCCPGMRCKCGILWNAGQCKCTNYGYGR

>CCWa_Nemertea_Lineus_ruber_Lvir.rna.tri.50172.1_m.91402

MNCNYMPLALAYVLLLCLEAVLTRTAYNNPYEDEYPGEYQYEELIASPAEYDGRLPVSSNNLLDIFKSTRKRSRHVSKRCYSSRYCCKTTSDCCAGYTCGVNIWNWNCYECIKKTWGR

>CCWa_Nemertea_Lineus_ruber_Lvir.rna.tri.7362.1_m.15991

MHVVDKAHKMNTVLTIFVVIASISLVLSSSIPQYDEDDDGSFLDVLDSYKTATKRQALTRNYYNNDYAYYYKRNCKRWNDWCDPWSSNPTRSCCPNERVACKCNFFGSNCRCTRKLWGR

>CCWa_Nemertea_Lineus_ruber_Lvir.rna.tri.41828.1_m.78628

MDTKGKCCILMLLLVVVFIQASEAFVRREHDTPSSGSIGRHERGACRQKKDFCEKGNPGLACCTGLLCSCGVFNYCRCSDPTEIM

>CCWa_Nemertea_Lineus_longissimus_c35901_g1_i1.p1

MDSKGKCCLLLVLLVGVLIHASGASGRRDQDRPFDGISERHERGACRRKKEFCEKDNPGLTCCPGLLCSCGLYDYCRCSDPTEIM*

>CCWa_Nemertea_Lineus_longissimus_c37514_g1_i1_m.43160

MNTVLTIFVVIASISLVLSSSIPQYGEDDDGSFLGVLDSYKTATKRQNRCRRWGESCDPRRKNGYFICCEKYYCKCSIWGSCRCTGTMFG

>CCWa_Nemertea_Lineus_longissimus_c37514_g1_i3.p2

MNTVLTIFVVIASISLVLSSSIPQYGEDDDGSFLGVLDSYKTATKRKVEFKSIHLWFLWSHCGSGRALLSCRKLVKVLNNGIHSTSDFPK*

>CCWa_Nemertea_Lineus_longissimus_c34565_g1_i3_m.31336

MKLYIVACLFLVCALQFVASYPYGDENEYGDDQLEVFRDAAKRAFYNFKCATSSCRPRAMEPTLRCCPGMRCKCGILWNAGQCKCTNYGYGR

>CCWa_Nemertea_Lineus_longissimus_c34565_g1_i1_m.31332

MKLYIVACLFLVCALQFVASYPYGDESKAKSKKSGDNQENEYGDDQLEVFRDAAKRAFYNFKCATSSCRPRAMEPTLRCCPGMRCKCGILWNAGQCKCTNYGYGR

>CCWa_Nemertea_Lineus_longissimus_c37514_g1_i2_m.43162

MNTVLTIFVVIASISLVLSSSIPQYGEDDDGSFLGVLDSYKTATKRQALTRNYYNDYAYYYKRNCKRWNDWCDPWSSNPTRSCCPNERLACKCNFFGSNCRCTRKLWGR

>CCWa_Nemertea_Lineus_longissimus_c39290_g1_i1_m.51965

MNCNYVPLALAYVLLLCLEAVLTRTTYNNPYDDEYPEVYQYEQLSLPAEYVEELPVSRNNLLDIFKSTRKRSRQVAKRCYSSRNCCKTTADCCPGYTCGVNIWNWSCYECIKKTWGR

>CCWa_Nemertea_Cerebratulus_spec_TRINITY_DN24351_c0_g1_i2_m.170545

MKLYIVACLFLVCALHFVASYPYGDENKDKKSDNQDGEYGDEQLDVFRDAAKGLEAPPERRAFYNFKCATSTCRPRSMEPTLRCCPGMRCKCGILWNAGQCKCTNYGYGR

>CCWa_Nemertea_Cerebratulus_spec_TRINITY_DN24351_c0_g1_i1_m.170543

MKLYIVACLFLVCALHFVASYPYGDDGEYGDEQLDVFRDAAKRAFYNFKCATSTCRPRSMEPTLRCCPGMRCKCGILWNAGQCKCTNYGYGR

>CCWa_Nemertea_Cerebratulus_spec_TRINITY_DN24351_c0_g1_i7_m.170551

MKLYIVACLFLVCALHFVASYPYGDENKDKKSDNQDGEYGDEQLDVFRDAAKRAFYNFKCATSTCRPRSMEPTLRCCPGMRCKCGILWNAGQCKCTNYGYGR

>CCWa_Nemertea_Cerebratulus_spec_TRINITY_DN30212_c0_g2_i1_m.171894

MNSVLTTFVALISVSLVLSSSIPQYDDSEEAGFLSELDNYKTASKRRDTRCRRWGESCDPRRKNGYFICCEKYYCKCSMWGSCRCTGTMFG

>CCWa_Nemertea_Cerebratulus_spec_TRINITY_DN30212_c0_g1_i1_m.171888

MNSVLTTFVALISVSLVLSSSIPQYDDSEEAGFLSELDNYKTASKREAVVKNYYNDYAYYYKRNCKRWNDWCDPWSSNPTRSCCPNERLACKCNFFGSNCRCQRKLWGR

>CCWa_Nemertea_Cerebratulus_spec_TRINITY_DN14586_c0_g1_i1_m.129862

MNCNYVPLALAYILLICLEAVLTRTPYNSRYEDEYPDEFQYEIAAPSEYDDGQTPFDKSNLLDIFKSTRKRQVSKRCYTSRFCCKTSRDCCSGYTCGVNIWNWNCYECIKKTWGR

>CCWa_Nemertea_Cerebratulus_spec_TRINITY_DN64514_c0_g1_i1.p2

MRSVTAVTILVVMLALASVTYQEESCGGDRCAYSEGCCNDCYSCVCKFPGSNCTCKKRMFSCWGKR*

>CCWa_Nemertea_Cerebratulus_spec_TRINITY_DN25921_c0_g1_i1.p2

MDSTRTAWVLVLLLGMAFLCETEAWLIKRHQVKDSFERERRGACRKKKEFCETDNPGLTCCRFLQCSCGLYNYCRCSDPTEIME*

## CCWamide-Prohormone 3-related

>unident_ProHo3_CCWa_related_Nemertea_Riseriellus_occultus_TRINITY_DN72621_c0_g1_i1.p1

MAAMRLQIVVIAILAAVAIQSTQSASISDKEKSDGKLLKRVRRDFWGTGMFANTDPCLKACWKTRNCCPGYYCTAEIWSNDGAVGVCKE

>unident_ProHo3_CCWa_related_Nemertea_Nipponemertes_spec_TRINITY_DN5443_c0_g1_i1.p1

VHGQSHADDQCEKQQHELQRIHRCSLLGLSGVIVSCGSLWRQACVPCRRSSECCSGYYCTRSIWSITDQVGVCRQYSTGYWGKRFSPFK*

>unident_ProHo3_CCWa_related_Nemertea_Nipponemertes_spec_TRINITY_DN5443_c0_g2_i1.p1

VCMALAVHGAAISADADSSDDDSSLLKRIRRDGDFWGSRGDKCSYPCRRSSECCSGYYCTRSIWSITDQVGVCRQYSTGYWGKRFSPFK*

>unident_ProHo3_CCWa_related_Nemertea_Lineus_lacteus_comp11616_c0_seq1.p2

MAAMRLQIVVIAIIIAVTIESTQSASISDKEKSDGQLLKRVRRDFWGTGMFANTDPCVKACYKSRNCCPGYYCTAEIWSNDGAIGACKEYNPGFWGKRSFGY*

>unident_ProHo3_CCWa_related_Nemertea_Lineus_longissimus_c35681_g1_i1.p1

MATMRLQIVVIAIIAVVAIHLTQSASISDKEKSDGQLLKRVRRDFWGTGMFANTDPCVKACWKSRNCCPGYYCTAEIWSNDGAIGACKEYNPGYWGKRSFGY*

>unident_ProHo3_CCWa_related_Nemertea_Cerebratulus_spec_TRINITY_DN27226_c0_g1_i1_m.21938

MATMCLQVVAIATVFAALAIQSAHCASISDKEDSEGKLLKRVRRDFWGTGMFANPDPCEKSCQKTQHCCPGYYCTANIWSNGPTQIGVCREYNPGYWGKRSYGV

>unident_ProHo3_CCWa_related_Nemertea_Lineus_ruber_Lvir.rna.tri.60791.1.p1

MATMRLQIVVIAIIIAAVAIQTTQSASISDKDKSDGKLLKRVRRDFWGTGMFANHDPCLKACLKTRNCCPGYYCTAEIWSNDGAIGGCKEYNPGYWGKRSFEN*

>unident_ProHo3_CCWa_related_Nemertea_Cephalothrix_hongkongiensis_comp26940_c0_seq1.p1

MNSFLPLAAICCFSLLLVVVIASRNTEAVDTQLQLQNNDIANGLDLERFRRSFWGNLNGYNPCRRYCSRHSQCCSGYYCSSPSYTSLVSGTCRLKVRGYWGDRF*

>unident_ProHo3_CCWa_related_Nemertea_Tubulanus_polymorphus_comp31546_c0_seq1.p1

MGFSIRVNIWAVVFIACLSAYFSHSLPDNKENENGDEKQDAYKDFIQNRLRRDFWGNGWLDGSRYDKCIRSCSRSSGCCKGYYCSVLSWANNRRLGKCKKFRSGYWDIEFK*

>unident_ProHo3_CCWa_related_Nemertea_Notospermus_geniculatus_scaffold818.g19974.t1.p1

MRTTMLSHSNDADTESFSWTDDSRPKFYNRTKMATMRLQLLAVAALVAALSIQTAQSASIKEKEKSGVKLLKRVRRDFWGTGMFANPDPCERACQKTRDCCSGYYCTTIIWSSRPQEIGSCREYNQGYWGKRAYGF*

## CLCCY

>CLCCY_Phoronida_Phoronis_psammophila_comp64836_c0_seq1.p1

MFQRVFRIVALCFILVVVLARTEARSSALSKLLTKRQCIPQAVPCTPYQDRCCGYMECLNILQDYCFPGEQCYCLPSLNHDHLH*

>CLCCY_Phoronida_Phoronis_psammophila_comp64836_c0_seq2.p1

MFQRVFRIVALCFILVVVLARTEARSSALSKLLTKRQCIPQAVPCTPYQDRCCGYMECLNILQDYCFPGEQCYCLPSLNHEHLH*

>CLCCY_Phoronida_Phoronis_psammophila_comp64836_c0_seq3.p2

MFQRVFRIVAPCFILVVILARTEAGSSALSKILTKRQCIPQAVPCTPYQDRCCGYMECLNILQDYCFPGEQCYCLPSLNHEHLH*

>Brachiopoda_Terebratalia_transversa_Ttra.rna.tri.5882.1.p4

MKCVSSILALISCLLLLSRSQVKGMKLDDHLHKRGGNKCIPQGNGCSYYRPEIGYLCCGEMTCANHYGDFCVPNEDCFCQPVHYNG*

## Corazonin

>CRZ_Phoronida_Phoronis_ijimai_(P._vancouverensis)_comp82847_c0_seq1.p1

MQCQDAIKILATFLLVILVTEAQQVQFSKGWHPPGKRAQTQQCTLIRTSMRRLLIDFVQFQLRRLESSGCTDEGQDSFVNGQTNKSLFEMASKLSRVLGQQN

>CRZ_Phoronida_Phoronis_australis_TRINITY_DN207679_c0_g28_i1.p1

MQYPDTIKILATLLVILLVTEAQQVQFSKGWHPPGKRTQAQQQCRVMRTSMRRLLINFVQFQLQRLEISGCARRGQDRFLNNQEQTDKSLFEMASKLSSDFAQEN

>CRZ_Phoronida_Phoronis_psammophila_comp67052_c0_seq1.p1

MVKQVVLKMVALFLAFLALTQAQQVQFSKSWYPPGKRGEGSQNSNCAVLRSAMKKLLMDFVQYQLQVFESSDCSRQDMEYLINTGIRVNKAFFQKAPKMRDDLSK*

>CRZ_Brachiopoda_Novocrania_anomala_Nano.rna.tri2.91245.1.p1

MAGLRIVAFVLLLEALCQLLPIRGQEYHYSNDWKPGKRSMEENECRVRPVVQKIVQTLLENEYRRIQTVCPYGTKRNQKPTSLKQMFTKRMAEHRPYN

>CRZ_Brachiopoda_Terebratalia_transversa_Ttra.rna.tri.23073.1.p1

MNMRLPRLAILVVISLWCIDSMHSQEYHYSNNWSPGKRYTNLLANTDHNTDCQVRPELRKLIDELIKAEYKRLSKVCLKQNKGLMEKFWKKIYNGN

>CRZ_XP_013384764.1 prepro-gonadotropin-releasing hormone-like protein [Lingula anatina]_Brachiopoda

MSKLVLSLSIIGLILMQVVLYTSAQMYHYSKGWGPSSSGKRNSPENLCQVRPFIRKLVHDVIQGEFERLKTVCAHGQWLGGAGTTQSLWEQIERRLPQDEPSLE

>CRZ_Nemertea_Baseodiscus_unicolor_TRINITY_DN53128_c0_g1_i1_m.207498

QGQGFHFSQGWLPNGKRASSEYTNIMNKLGEPVSAVQKGDKCGVTQEDARAMMRMIQEAVHRQRSFCESDVHIMTALFHVIKAEMNN

>CRZ_Nemertea_Lineus_lacteus_comp12594_c0_seq1_m.16642

METIVTKCLVFLMVTLPLMQTVHSQGFHYSPGWEPSGKRSQSSYNEFMSKVRQWVSLGSAENSGKCALTPESASAVMKIIQNEVQRSRSFCRSDVAIMSALYNVARSDIKKD

>CRZ_Nemertea_Lineus_longissimus_c38989_g1_i1_m.50268

MEAIVTKSLVLLMVTFTLTQTVHSQGFHFSQSWRPNGKRSQMNNNEIMGKVRQWASLGNVENSGKCALTPEGAGAVMKIIQDEVQRSRSFCRSDVAIMSALFNVAGTDIKKD

>CRZ_Nemertea_Lineus_ruber_Lvir.rna.tri.54447.1_m.97348

MEPIVTKYLVLLMVALTLIQTAHSQGFHFSQSWRPNGKRSQSNYNEIMGKVRQWASLGSIEKSGKCALSPGGAGAVMKMIQDEVQRSRSFCESDVAIMSALLNVARTDIKKD

>CRZ_Nemertea_Cerebratulus_spec_TRINITY_DN37499_c3_g1_i1.p2

MEPIVTKCLVVLMVTLTLMQVAHGQGFHFSQSWRPNGKRSQASYNEMMGKARQWESFGGIQSGKCAMTPESATAVMKIIQDEVQRSRSFCESDVAIMSALFNVARTDNKKH*

>CRZ_Nemertea_Tubulanus_polymorphus_comp30867_c0_seq1.p1

MMCRIIILAIALVSTCQIVVNGQGFHFSHDWKASGKRSLGGYQDDNQGNNNNQGNGNKLGLLDTAINAQSNGSKCLVNKEAAQLILRLIEDELSRRHRMCKTDALIVEALNRARLEI*

>CRZ_Nemertea_Tubulanus_polymorphus_comp30867_c0_seq2.p1

MMCRIIILAIALVSTCQIVVNGQGFHFSHDWKASGKRSLGGYQDDNQGNNNNQGNGNKLGLLDTAINAQSNGSKCLVNKEAAQLILRLIEVRIAFISFHFSLLLSCFKCVRTITSYSWP*

## DH31

>DH31_Brachiopoda_Novocrania_anomala_Nano.rna.tri2.55397.1.p1

MTKVQLIAVLHVFCVFGMVTAIPAYRRTEENRIRNSIQELEHKVSLLKYLLHQYTLQPYKRTVDMGYMSRNGAASKAGLIQLALHNAMDPYGPGRRR

>DH31_Brachiopoda_Glottidia_pyramidata_comp697727_c0_seq1.p1

MKKETIVHVYASLFCVLLVCMIATEAYSDLSSIEEEITNLDQEVDVLTNLVRYLKSRTQNGRKRQIDAGYGSRNMVAQDISGKMLALH

>DH31_Brachiopoda_Lingula_anatina_comp150897_c1_seq4.p1

MKKPMVVQVYASLFCVLLICLTVTEAYSYINGLEDQVTDLDKEVDVLSNLVRYLKSRTTNGRKRQIDAGFGSRNTLAQDISRLVLAQHKAQSYNSPGRRRRSINTNSQLRH

>DH31_Nemertea_Cerebratulus_spec_TRINITY_DN28502_c0_g1_i1.p1

MDSRNIMGLCLLAIVLPAVVMSLPAKERKRSASYLSNPVETVAVLEELIGLIKDNAAYGDLKKRNLDAGYGSRFSAANNYGSKLMALHAASDYASPGRKRREAAAEQN*

>DH31_Nemertea_Cephalothrix_hongkongiensis_comp38260_c0_seq4.p1

MKSLIAAALIAVVLPGLVYSVPAKKRAPAQISDPALTIDVLEELIRYVAKQGEYDEKVKRTFDAGYGSRFGAASNYGSKLMALQAASDFSGPGRKKRETIEEA*

>DH31_Nemertea_TubulanusPolymorphus.42223 _

MMTQVIGLCLLAIIIPTLAAPAATKKRSVDSLSNPSLTIAVLRDLIDNIVERHNLKSASKRGSFDGGYGSRFASASNYGNKLMALQAANSYDSPGRKRRDATDEQ

## DH44/ELH

>DH44_ELH_lopho_Corticoliberin_DH44_ELH_Nemertea_Riseriellus_occultus_TRINITY_DN20872_c1_g1_i1_m.41197

DSASDMRKRSGSELSVMGSLDALSDIISSQNAKHMHDRMQANHQRLLRLGKRDTSANLEKTEKEATK

>DH44_ELH_lopho_Corticoliberin_DH44_ELH_Nemertea_Nipponemertes_spec_TRINITY_DN22176_c0_g2_i1_m.36976

FQQVDELSSKNGPESPLRHSPELSDKRSGDLSIVASLDALSDFVKAQEGRRMRGALSASHMKLLRLGRRAAGEAS

>DH44_ELH_lopho_Nemertea_Baseodiscus_unicolor_TRINITY_DN17756_c0_g1_i1_m.157115

RFPRAARTALQKRTGGELSVLGSLDALSDMISAQQARKLQDMMSANHQRLLRLGKRGLVHLERMDENADGNTQKGTSL

>DH44_ELH_lopho_Corticoliberin_DH44_ELH_Nemertea_Tubulanus_polymorphus_comp30742_c0_seq1_m.24733

MHSKHGSMITLTTVFILAVIVAIVEGKPLSDEYYEPLPSEFKTNLPSENSMREKDNDLYRKVAQIAKMVLNRGKGDMSEYKYDDDSVDKRGGGSLSIVGSLDALTDMLSAQQARKFQNQLKANKMRLMRLGKRDVDSKFRH

>DH44_ELH_lopho_Corticoliberin_DH44_ELH_Nemertea_Lineus_lacteus_comp13720_c0_seq1_m.18950

MFRHLPFTIGVAIVLLSCLEARPYAIQEEYLPLDDEFETDTLGENGEEKRSMDLSVSGSLAALSDMLMWQRQQQQQQQLNALGKRSSRFNNQEIPELLKQLQRLRDSASDMRKRSGSELSVMGSLDALSDIISSQNARHMHDMMQANHQRLLRLGKRDTSANLEKTENQETKKAD

>DH44_ELH_lopho_Corticoliberin_DH44_ELH_Nemertea_Lineus_longissimus_c46913_g1_i1_m.121473

MFRHLPFTIGVAIVLLSCLEARPYAIQEEYLPLDDEFEADAFRENGDEKRSMDLSVSGSLAALSDMLMWQRQQQQQQQLNALGKRGSEFNNQETQELLKQLQLLRDSASGMRKRSGSELSVMGSLDALSDIISSQNARHKHDMMQANHQRLLRLGKRDTSANLEKTKNEETKKAD

>DH44_ELH_lopho_Corticoliberin_DH44_ELH_Nemertea_Lineus_ruber_Lvir.rna.tri.30736.1_m.60043

MFRPLPLTIGVAICIVLLNCLEARPYSIQEEYLPLDEGIEVDPFRENNDEKRSMDLSVSGSLAALSDMLMWQRQQQQQQQLNALGKRAAGFSNQEAPELLKQLQLLRDSASNVRKRSGSELSVMGSLDALSDIISSQNARHMHDMMQANHQRLLRLGKRDTSANLEKPGEDETKKVD

>DH44_ELH_lopho_Corticoliberin_DH44_ELH_Nemertea_Cerebratulus_spec_TRINITY_DN7449_c0_g1_i1_m.134876

MFRHVSLLLGATICLSLLLCLDARPYSVQEEYLPLEGGYDDDTYDESGAEKRSIDLSVSGSLSALSDMVMWQRQQEMQQQLAALGKRTSGMSNRRVPALFRYLQRLRSGSIPEIRKRSGGDLSVLGSLDALSDIISSQHAKHLQDMMQQNHQGLLRLGKRDTSASLEKAEKEETKKAE

>DH44_ELH_lopho_Corticoliberin_DH44_ELH_Nemertea_Notospermus_geniculatus_g17259.t1

LRRKFKSFLVHHQARPYAFKEEIIPLDDGYDDEDAFLENGAEKRSADLSVSGSLAALTDMLMWQRQRQMKDELAALGKRTSGLNNVPVPAIFRRFQRLSNMNKRSGSDLSILGSLDALSDIIASQQAKHLSDVNHQRLLRLGKRDTSASLDGAEKAETKKAE

>DH44_ELH_lopho_Corticoliberin_DH44_ELH_Nemertea_Notospermus_geniculatus_g10566.t1

MFRFLTLPIGVTICLSLFLCLEARPYAFKEEIIPLDDGYDDEDAFLENGAEKRSADLSVSGSLAALTDMLMWQRQRQMKDELAALGKRTSGLNNVPVPAIFRRFQRLSNMNKRSGSDLSILGSLDALSDIIASQQAKHLSDVNHQRLLRLGKRDTSASLDGAEKAETKKAE

## dkk related

>dkk_related_Phoronida_Phoronis_australis_TRINITY_DN117198_c0_g1_i1.p1

MDYVKLFLYGCLVTLQMTAPALGYIWNLAWSNSNVDSTYNDNSPHPRNVRRTDWVRGTDDIEENTTERFVPCRSDRSCGRGKFCDRHYGTCTFHREAGDVCRRDGHCQRGHDCMFGRCQKAITAGLEGARCKHEKDCGVGLCCARKHGEKVCKKKLALGQKCYVPRGGLAYSVNEVCPCDVELTCKNTSKKKTEEEEETWRFWTDFDHMRCAPV*

>dkk_related_Phoronida_Phoronis_psammophila_comp921165_c0_seq1.p1

YDDRSPHPRNIRGGDPPKRLEDLEENTTERFVPCRSDRSCGRGKYCDRHYGVCEFHRETGDVCRRDGHCKRGHDCMFGRCQKAIRLGHEGARCKHDKDCGSSMCCAR

>dkk_related_Phoronida_Phoronopsis_harmeri_c82240_g1_i2.p1

FQFPGARCKHDRDCGASMCCARKHGEKICKRKLVVGQKCYVPNGGMTYSLNELCPCEEGLRCKDTSTKNQDEEDETWRFWTDFDHMRCAAL*

>dkk_related_Brachiopoda_Glottidia_pyramidata_comp33738_c0_seq1.p1

MDLNTILTSIVVWNFLLASFSDAYIWNIAWGSLGTDEDLSNHPKNIYRDALERVQLDEPVYPFEPCTTDKSCGRHRFCDKFYGTCESHRKQGDACRRDGHCQKGFDCMFGKCQKSLKAGEIGARCKHDRDCGSSMCCARQHGEKVCKAKLRLAENCYVPKGGLDYSLNELCPCDTGLVCKDTGRKTPREEELTWQYWSSFEHMKCAEP*

>dkk_related_Brachiopoda_Hemithris_psittacea_comp7288_c0_seq1.p1

MRFVTDLALVVIVYSLQSALVVCEAYIWNFALNSPQLQQQQADYANFHPRNIATSQSIPENSIVKFKPCVSDKACGKGKFCDKHYGFCDRKRPVTKYCRRDGHCEHGLQCMFGKCKKLTRHGAIGARCRHDRDCGSSMCCARQHGEKICKAKLKRGAKCYVPIGGLDYSLNEMCPCDSGMICKATVIKEKSTD

>dkk_related_Brachiopoda_Laqueus_californicus_comp10056_c0_seq1.p1

GHYGRGRRDGQCEHGLQCMFGKCTRTLKHGTVGARCRHDKDCGASMCCARQHGERICKAKLKIHAKCYVPQGGLEYSLNEMCPCDSGLTCRPNLKQKDDSPSWRFWSTYDHMRCSPP*

>dkk_related_Brachiopoda_Lingula_anatina_comp138196_c1_seq1.p1

MSLASLMVHLSRKNVFFYGARCKHDRDCGSSMCCARQHGEKVCKAKLQLAENCYVPKGGLDYSLNELCPCDKGLICKDTGRKTPREEELTWQYWSSFEHMKCAHP*

>dkk_related_Brachiopoda_Lingula_anatina_comp138196_c1_seq2.p2

MDFNTVFTTIVVWNFFLATFCDAYIWNIAWGSIGTDEDLSNHPKNIYRDALERPQLDEPTYPFDPCTTDKSCGRHRFCDKFYGTCEPHRREGDACRRDGHCQKGFDCMFGKCQKSQKAGELGARCKHDRDCGSSMCCARQHGEKVCKAKLQLAENCYVPKGGLDYSLNELCPCDKGLICKDTGRKTPREEELTWQYWSSFEHMKCAHP*

>dkk_related_Brachiopoda_Novocrania_anomala_Nano.rna.tri2.13876.1.p1

MDIYRVFLQLVGILNFIVVTHGYIWNLAWSTGVQDDEYDETSKHPRNLHRSREITDNNPCTSDKTCGRGKYCDKHYGTCEHHREGGHTCRRDGHCQRGFQCMFGKCQKAIKSGNLGARCKHAKDCGGNMCCARQHGEFVCKARLKLNYKCYVPRGGLDYSLNEMCPCEQGLICKNTQTKEDDITWRLRRNYDNMRCAQP*

>dkk_related_Brachiopoda_Terebratalia_transversa_Ttra.rna.tri.6743.1.p1

MVSSQVLVSAIVFWIIQWRSILCSAYIWNFALSSTLNNKVDYDNFHPRNIRAFKDRISQHNAIDKQEICKTDKSCGKGKFCDRHYGFCDRKRQSGQYCRRDGQCEHGLQCMFGKCTKSLKHGTIGARCRHDKDCGASMCCARQHGERICKAKLKIHAKCYVPQGGLEYSLNEMCPCDRGLTCKPTLKQKDDSPSWRFWSTFDHMRCSPG*

>dkk_related_Nemertea_Cerebratulus_spec_TRINITY_DN19829_c0_g1_i1.p1

GRFCDRHYGTCDLHRREGQPCRRDGHCAKNMDCMFGRCVKTIPVGKVGSRCKHDSDCGPQMCCARRHGEKVCKAKLSQGHRCYVPRGGLDYSLNEICPCAEGLLCQMSGSSDQERSPQGGSYEWTDYRRMQCAPP*

>dkk_related_Nemertea_Lineus_longissimus_c28384_g1_i1.p1

MSIDGILLMLFVLALDVLCSGANIWNWALSQPFDQSAYQARRNHPRNIHQTLRQNAPEGASPIFMQCVSDRTCGRGRFCDRHYGTCDLHRREGQPCRRDGHCTKNTDCMYGRCVKTILVGQAGSRCKQDGDCGQNMCCARRHGESVCKARLSQGDKCYVPRGGLDYSLNEICPCEEGLLCRMPGVGPQERHTEDSGYEWTAYRRMQCAPP*

>dkk_related_Nemertea_Lineus_ruber_Lvir.rna.tri.73048.1.p1

MSIDGILLLLFVLALDVVCSGANIWNWAMSQPFDQSAYQARRNHPRNIHQTLRRNVPDDTAPKFLPCVNDRTCGRGRFCDRHYGTCDMHRREGQPCRRDGHCTKNMDCMYGRCVKTIPAGQVGSRCKQDNNCGKNMCCVRRHGESVCKARLSQGERCYVPRGGLDYSLNEICPCEEGLLCRKSGLGSQEEPTESSGYEWTDYRRMQCAPP*

>dkk_related_Nemertea_Notospermus_geniculatus_scaffold4967.g40387.t1.p2

GHCAKNMDCLFGRCSKTIPDGQIGARCKHESDCGKNMCCARLHGERVCKARLSRGQRCYVPRGGLDYTLNEICPCQQGFICRIPESDLQEKPTVRSPHEWSDYRRM

## EFLGamide/FSE peptide/TRH

>FSE_Nemertea_Baseodiscus_unicolor_TRINITY_DN107083_c1_g4_i2.p2

MKILHIKNRPVNLAGHLGAGKRFSEFLGAGKRFSEFLGAGKRFSEFLGAGKRFSEFL

>FSE_Nemertea_Baseodiscus_unicolor_TRINITY_DN107083_c0_g4_i1_m.22848 5prime_partial

GAGKRFSEFLGAGKRENKQQHVEYPAENRRFSEFLGAGKRFSEFLGAGKRFSEFLGAGKRQHN*

>FSE_Nemertea_Cerebratulus_marginatus_comp1270136_c0_seq1_m.143945 internal

FAGCKEGADDKRFSEFLSVGKRSPGDNKRFSEFLGVGKRNVGDKRFSEFLGVGKRNPDKKFSEFLAPGKRFSEF

>FSE_Nemertea_Cephalothrix_hongkongiensis_comp1774_c0_seq1_m.607 5prime_partial

FLPGKRFNEFLPGKRFNEFLPGKKRFSEFLPGKRFSEFLPGKKRFSEFLPGKRFGEFLPGKKRFNEFLPGKRATN*

>FSE_Nemertea_Riseriellus_occultus_TRINITY_DN43881_c0_g2_i1_m.45220 5prime_partial

VGKRNTDDDKRFSEFLGVGKRNPDKKFSEFLAPGKRFSEFLAPGKRFSEFLAPGKKRSTS*

>FSE_Nemertea_Riseriellus_occultus_TRINITY_DN43881_c0_g1_i2_m.45222 3prime_partial

MNEYLVPGKRFSEFLGAGKRFSEFLAPGKRFSEFLAPGKRFSEFLGVGKRNQDKRFSEFLLPGKRFSEFLAPGKRFSEFLGVGKRNTD

>FSE_Nemertea_Notospermus_geniculatus_g2831.t1

MDEFLVPGKRFSEFLGTGKRFSEFLGTGKRFSEFLAPGKRFSEFLAPGKRFSEFLAPGKRFSEFLGTGKRNSDDDDKRFSEFLGVGKRNSHKRFSEFLAPGKRFSEFLGVGKKRSTS

>FSE_Nemertea_Lineus_longissimus_c47877_g1_i1_m.133325 5prime_partial

KRFSEFLGVGKRTPENDKRFSEFLGVGKRNPDKKFSEFLAPGKRFSEFLAPGKRFSEFLAPGKKRSTS*

>FSE_Nemertea_Lineus_longissimus_c18169_g1_i1_m.9683 internal

KRFSEFLAPGKRFSEFLAPGKRFSEFLGVGKRASDKRFSEFLLPGKRFSEFLAPGKRFSEFLAPGKRFSEFLGVGKRASDKRFSEFLLPG

>FSE_Nemertea_Lineus_longissimus_c31466_g1_i1_m.23469 3prime_partial

MSPSYKTVGLLAIVLLQVAMVTVADDEPATGNDATELEDALLQDYVTDMAHANAISADDSPLDVQRKVKDYASKRFNKFLETENRLNGYLVPGKRFSEFLGVGKRFSEFLAPGKRFSEFL

>FSE_Nemertea_Lineus_ruber_Lvir.rna.tri.25989.1_m.51978

MDEYLVPGKRFSEFLGVGKRFSEFLAPGKRFSEFLAPGKRFSEFLGVGKRTSDKRFSEFLLPGKRFSEFLAPGKRFSEFLAPGKRFSEFLGVGKRTPEDEKRFSEFLGVGKRNPDKKFSEFLAPGKRFSEFLAPGKRFSEFLAPGKKRSSS*

>FSE_Nemertea_Lineus_lacteus_comp21558_c0_seq2.p1

DKRFSEFLGVGKRSPDKKFSEFLAPGKRFSEFLAPGKRFSEFLASGKKRSTS*

>FSE_Nemertea_Lineus_lacteus_comp21558_c0_seq1_m.47379 3prime_partial

MSPSFKTVVLLAIVLLQVAMVTVADDEPATGNDAAELEDTLLQDYVTDLIHANAIDANDSPQDVQAKVKDYASKRFNKFLATENRMNGYLVPGKRFSEFLGVGKRFSEFLAPGKRFSEFLAPGKRFSEFLGGKRTSDKRFSEFLQGKRFSEFLAPGKRFSEFLGVGKRTPED

>FSE_Nemertea_Cerebratulus_spec_TRINITY_DN36954_c0_g1_i6_m.68660 complete

MSPSYRCVVLLALVLLQVVLFTVAEDPQPATGNDAAQLEDALLQEYMTDLMNENAINENDSPQDVQTKVKDYAHKRFNQFLATDSRMNGLLVPGKRFSEFLTKRFSEFLAPGKRFSEFLAPGKRFSEFLAPGKRFSEFLAPGKRFSEFLAPGKRNADDDKRFSEFLGVGKRNADDGKRFSEFLGVGKRNPGDKRFSEFLGVGKRNPDKKFSEFLAPGKRFSEFLAPGKRFSEFLAPGKRFSEFLAPGKRFSEFLAPGKKRSTS*

>FSE_Nemertea_Gamide_Tubulanus_polymorphus_comp26466_c0_seq1_m.16229 3prime_partial

MKILITKPLALKSIRRKGGQVLLANDPKIRNTKLGQVKLDKKCRKDNVDHVHDKLHRFVNKRFSEFLGGKKRFSEFLGGKKRFSEFLGGKKRFSEFLGGKKRFSEFPGKRFSEFPGKRFSEFLGGKRFSEFPGKRFSEFLGGKRFS

>FSE_Nemertea_Tubulanus_polymorphus_comp26466_c0_seq2.p1

MKHTWKVLMVTLVAILAEVMIMSNASEESLPNPADDDDRQLSNLESELYEKYLNQLANSGQLDTEDNVDHVHDKLHRFVNKRFSEFLGGKKRFSEFLGGKKRFSEFLGGKKRFSEFLGGKKRFSEFPGKRFSEFPGKRFSEFLGGKRFSEFPGKRFSEFLGGKRFS

## EP (excitatory peptide)/CCHamide

>CCHa_EP_Brachiopoda_Novocrania_anomala_Nano.rna.tri2.109752.1.p1

MKSLDCWTILAVSIIYACFCTFVTEGGKCRQWSSHHCWGGNGKRGEQETDGSRDRIIKLIDKILQASKNRHIEDNAENIFERNTYQHKTQTESMEPRSLVHSRDQRQHMLRDWLLEVKNSRR

>CCHa_EP_Brachiopoda_Lingula_anatina_comp138060_c0_seq3.p1

MRIRLDPWVVIPLAVVLACLLSHKTDANKCKGPWRVHGCFGGNGKRTQPEPRPHHNELLAKQLNLAEIGILKKILKATRTERMDFLNSDKINSKSLSKHQHDRQAEYRTTRLPLLPLQSNSHLTPEKMFDGNLKDNHNRINLTEIYFQLKRTTLRRLFQMLQRKWNP

>CCHa_EP_Brachiopoda_Lingula_anatina_comp138060_c0_seq1.p1

MRIRLDPWVVIPLAVVLACLLSHKTDAITLDQLIARGILPPELDKCKGPWRVHGCFGGNGKRTQPEPRPHHNELLAKQLNLAEIGILKKILKATRTERMDFLNSDKINSKSLSKHQHDRQAEYRTTRLPLLPLQSNSHLTPEKMFDGNLKDNHNRINLTEIYFQLKRTTLRRLFQMLQRKWNP

>CCHa_EP_Nemertea_Tubulanus_polymorphus_comp23434_c0_seq1_m.12549

MRTWATWSVFLMAMVYLCLTFKTVSAGKCQGEWFRHSCLAGNSGKRSDSHIENRSKSEDYLKKLLSLLKTEIEMKSLPSSPVPEQEEADERPSRDYNTDKTISEPLQALWEKFLMRTREQEDMTRRK

>CCHa_EP_Nemertea_Paranemertes_peregrina_comp184981_c0_seq1_m.172293

MNLRVFWSSAFILLIVNFTSARASKCRGRWAIHACAAGNGKRSGPLYPPDDDTDSDAAADNRQKLVDLIQQLKLQAQPQ

>CCHa_EP_Nemertea_Malacobdella_grossa_comp16369_c0_seq1_m.8916

MLQRVIWTSAFILLFINFSLARRAGKCRGRWAIHACAAGNGKRSGPMYRPEDDLVELNNDRQKLVDLIQQLKSQLQTSPPEPAQIPLSSRFDVREEEPTIERSSELWKRLLSKIRSRDLLNA

>CCHa_EP_Nemertea_Nipponemertes_spec_TRINITY_DN22669_c0_g2_i1.p1

MGREDRELNMKTLASWSLILLATLFIYIASYHTANASKCKGRWAIHACAAGNGKRSESPYPSDGFDHRPGAGRQTLEDMIQELRKRIGDKARFMTSDPRDAEGNNEQPQEADSSEIWRRLYMKMRARSVMSD*

>CCHa_EP_Nemertea_Notospermus_geniculatus_g10614.t1

MAKRQVGNREQYKFYSTQGSACINLDLRTAMSGKCKGRWAIHACAAGNGGKRSDPRIQINGPIDRERNLQDMLEILRSRLLENEANEIADAELPTYETTENEDMWNRLYSRLKGRQSYIDA

>CCHa_EP_Nemertea_Baseodiscus_unicolor_TRINITY_DN87580_c0_g3_i1_m.175399

MSSNGVWSLIVLAFIYLCLGSCTVSASKCKGRWAIHACGAGNGKRSDPRLQIPDPSARRQKLEDMIELLRTRLLEEEAEEMTEELPTYETSDLTDDLWDKLYTKLKADQSYGRLQ

>CCHa_EP_Nemertea_Lineus_lacteus_comp18696_c0_seq1_m.33086

MSTYGVWSLLVLAFIYLCLGSYTVAGSKCKGRWAIHACAAGNGGKRSDPRLQIHLPLERQRNLQDMLEILRSRLLEDEANELAEEELPTYETTENDDMWNRLYSKLKERQSYGVAK

>CCHa_EP_Nemertea_Lineus_ruber_Lvir.rna.tri.23339.1_m.47236

MSTNGVWSLLVLAIIYVCIGSYTVAGSKCKGRWAIHACAAGNGGKRSDPRLQIHLPAERQRNLQDMLEILRTRLLEDEANALEEEELPTYETTENDDMWNRLYSKLKERQSFGVAK

>CCHa_EP_Nemertea_Cerebratulus_spec_TRINITY_DN37908_c2_g4_i1_m.7823

MSTYGVWSLLVLAFIYVCLGSYTVAGSSSQDGKCKGRWAIHACAAGNGGKRSDPRLQLNGPSERQRNLQDMLAILRSKLIEEEVNELEDEELPTYETTENDDMWNRLYSKLKESQSYADAK

>CCHa_EP_Nemertea_Lineus_longissimus_c46103_g2_i1_m.111745

MSTYGVWSLLVLVFIYLCLGSYTVAGSKCKGRWAIHACAAGNGGKRSDPRLQIHIPSERQRTLQDMLEILRSRLLEDEANELEEEELPTYETTENDDMWNRLYSKLKERQSYAVAK

>CCHa_EP_Nemertea_Lineus_longissimus_c46103_g2_i5_m.111760

MSTYGVWSLLVLVFIYLCLGSYTVAGNSGTDGKCKGRWAIHACAAGNGGKRSDPRLQIHIPSERQRTLQDMLEILRSRLLEDEANELEEEELPTYETTENDDMWNRLYSKLKERQSYAVAK

## FMRFamide

>FMRFa_Phoronida_Phoronis_psammophila_comp67440_c0_seq1.p1

MRVLCSLTATVLCFHLVASVRVGDVCDGVFGPVEPEDRGICSLISGASLTRRRRADVRRSPRPSSIGFTRLSRSAPWAENTSPLQQGAPMVIVSPMHSVKKRSVDAVKHTASKRSFMRFGRDPEKVYEKRHFVRFGRGGEDEDKMDDDMKEAFVKLGEAFMKLAEEKNLNGPQDIQKAFEKYGQDPESKRAFMRFGRNPEESKRAFMRFGRAFVRFGRGQELDEPSKKAFVRFGKKDVGDQQMEDEPDKRSFMRFGKRLSNDKENAMDKKAFMRFGKRGDEEPEDKRSFMRFGKRDVESAQKHVRELEHSKSTGKGNAA*

>FMRFa_Phoronida_Phoronis_australis_TRINITY_DN252244_c0_g1_i1.p1

MRAIISLVTASFCVQLSASVRIADVCAGVFGPVDDADQGICSFISGLPVGRKRRTSSTTRTPKPYGYVRLTRSSPWSRNLDYSDQYDSVIPMHRVKKRSVDAALKHTAGKRSLTNLTPTRPHDEDDKMDDEMREFLETLGEALVKMSAEEKLTDGEALKRAFVRIGRDPNNKRAFVRIGRNPEERKKAFMRLARAFVRIGRGDWGEPPSKKAFVRIGKNNDDEEINPAPEKKGWLRFGKRDLEEAPDKKGFIRFGRGDSELSMDKKAFMRFGKRDVESTKKMNASAKDKTNKTSTKM*

>FMRFa_Phoronida_Phoronis_ijimai_(P._vancouverensis)_comp92485_c0_seq1.p1

MRAIISVVAAAFCVQLSTSIRIADVCAGVFGPVDEADQGICSLISGSPVERKRRTSNPTRNPDTFGYVRMTRSSPWLRDLDYGDRYGSVIPLHRVKKRSVDAVQQHTAAKRSFMNLTPNSHKGEDVKMDDDMKQAFVTLGDALMKMSAEEKLTDGEVLKRAFLRIGRDPENKRAFVHIGRDPEQRKRAFMRFGRAFVRFGRGELEEQPSKKSEGDPAEHKKAFMRFGKSDAEMTDAPEKKGFLRFGKRVLDEVPDKKGFIRFGRSGDGELAMNKKAFMRFGKRDVESTKKMNAFAKDNKNKTSTKV*

>FMRFa_Brachiopoda_Novocrania_anomala_Nano.rna.tri2.80184.1.p1

MAKEAEMALLAAVLVIQLMVIPSGASQEKEAAKNMEFNQLRDDLSKLQQQLDDIKVLTAEVPEDKRFFDTPRYLTKRYYLRFGRGIVDPYPYEHPSDAVDGGFFDKPHYGFNKRHYLRFGRGIIDSNPHGYPSNALEGAMADGFEKNKRYLRFGRSVDRYVETPETHHLSKRSVKQKTPENGKTKEISPPKKETIKDKRYMRFGKKSIEDEKRYMRFGKKSDEDAQKRYMRFGKKSDNNEEKRYMRFGKKSDDEKEKRYMRFGKKDESPDE

>FMRFa_Brachiopoda_Terebratalia_transversa_Ttra.rna.tri.29781.1.p1

FWPSYGSQEKDMIPYKRGFFGGGGEYGEPEGLSSFVDEPYRGTIGKRDFLRFGKRRYDDNERSDQLEALNRVTRDFLRFGKRDFLRFGRSYNLRGYDSDNKRKHMRKRRSIKNESDKPSSIESNDVYNKGALLRSDKKSMSAAKARDIKRAFLRFGRLQKEYQQQDRKRAFLRFGKNTGINLDNRPTSDTKTETNSETDNGSAKTVTNDHVIDNNVDSKRAFLRFGKKCASCKRAFLRFGKSEKNLISSSNKSMKSSPLFGKEHTKRAFLRFGKSVNRE*

>FMRFa_Brachiopoda_Lingula_anatina_comp125826_c0_seq1.p1

MKTSSRIFLLAFVALYQIQLIRCSGYGNFLCNYGQELNALCNRLQEFQDSADLQAPDDSNSMWRTKHRFQLRLGRREDEGDNEDDEEEFEHLVGMLAPHLSRQRRAYLRFGRSAEWPGVDDIDENKRAYLRFGKRAYLRFGRSVDNKRTPNSGGMSNYMRFGRSAEPHHLSKRSVPVKQEATESKTKENTMKSAAVPNSEKRYMRFGKKSSEGDEKRYMRFGKKSTEDSAPETHAKEEKRYMRFGKKSQPNGEDPEKRYMRFGKKSMEQTDEDKRYMRFGKKSLDQPEEDKRYMRFGKKSAEQQEEDKRYMRFGKRSDNADDDDDAEEAVKRYMRFGKKMDPDFEKALKRYMHFGKKTDVSEAIKRYMRFGKKADDNDAEKRYMRFGKKSDSDTENEEKRYMRFGKKDKELSEIKRYMRFGKKDQPAKNEKRYMRFGKKSTV*

>FMRFa_Brachiopoda_Laqueus_californicus_comp45307_c0_seq1.p1

MNWYMLMVAIVVTCFLLDHACALYASRYIDPCSSWLKSINLKRLCYERRWLSYNDRAQDIMTDSLPGQKRYYTQPEGSSFFEDPYGGTIGKRDFLRFGKRGYGDDEGSDEKLEALNRVTRDFLRFGKRDFLRFGKRDFMRFGRSFPPLYDADTTKKKLHMRRRRSIKNELESGKTKPEVQNKRAFLRFGKNSQVDAKEVKRAFLRFGRPNKGHEQDQKRAFLRFGKKSDKIVDKKSDTKQGTHSDETKLVTDSKRAFLRFGKKCTKCLKRAFLRFGKSDKSHSYNKNSLISMPKPVSLSTHGKELMKRAFLRFGKSAK*

>FMRFa_Brachiopoda_Hemithris_psittacea_comp18193_c0_seq1.p1

FMRFGKKSNKRGFMRFGKKSDVPTSHFEMKSSSPNGMDSSKRGFMRFGKKSSSSNGMDSSKRGFMRFGKSENQLSGKLANFRNDIISKLKDSPQISADKIKAGPPMHS*

>FMRFa_Brachiopoda_Hemithris_psittacea_comp25223_c0_seq2.p1

EEEALNRLLRSNAAKRNFMRFGKRDFMRFGKRDFMRFGKRRHPMSGFEPNEESMMKRPHSRHRRSATEKQVFHDKSKRGFMRFGKREKGSDEENQILKNLLDGRGCSLEAGNSKQNLGEGSHEKRGFMRFG

>FMRFa_Brachiopoda_Hemithris_psittacea_comp25223_c0_seq1.p1

MYLIIALSICVVLCEATTSSQTGYESDLFCHSNAEKTPTVTKLCDTLMNIRNQLEKEVADEFAEMALKKRHHEIPLNFFGRTQSGSLGKRDFMRFGRKFPDVDSHYAERKRDFMRFGKREMSDQEANEEEALNRLLRSNAAKRNFMRFGK

>FMRFa_Nemertea_Baseodiscus_unicolor_TRINITY_DN112753_c0_g1_i1_m.142345 internal

EKRYMRFGKKNDGGYIRFGRESSEEDDIGEDEKRYIRFGKRDDDQKRYIRFGKRDNQEDAAEKRYMRFGRNDGINDMDKRYMRFGRNDGKNDADKRYMRFGRNDGLANTDKRYIRFGRGGGK

>FMRFa_Nemertea_Paranemertes_peregrina_comp5666_c0_seq1_m.3328 5prime_partial

ITAGEKRYMRFGRAHDEEKRYMRFGRAHEDEKRYMRFGKRSTRSKRSADETKEESPIERLQRLLPRGTGYMRFGRSSDALSDDDSQTGDKRFMPLGLRRADYSRSGRLGEDNEEKDDVSKRYMRFGRFMRFGKNINSAPSGYMRFSRDNGLMRLSRQLAEREPAAGYLRFGKK*

>FMRFa_Nemertea_Paranemertes_peregrina_comp14844_c0_seq1_m.9609 3prime_partial

MHPLNFLVLVTLAGQAYCGIDLSRLCQRRSRSLHYLMSIRLCDGYRGGSTPVYTSSRERELDLEPTATAIPENDSGKEEDSKEKKERANDGYLRFGRSVDDPTEKASDDKTKEDAKRYMRFGRAYMRFGKKSSDYLRFGQERDVDSDIANDKRYMRFGRGGEVVDDNSLHTEMDDSNGDNKRYMRFGRSNDAEKRYMRFGRAHEDEKRFMRFGKGYMRFGRSHQDEKRYMRFGRSHDEEKPYMRGG

>FMRFa_Nemertea_Nipponemertes_spec_TRINITY_DN15377_c1_g1_i1_m.42767 internal

LCNQYRTGNRPNQMSIVTSRERELELTPQPDTVTTKDDKPQTDDDSRRQTEGYLRFGRSAETPQEKSGEFAKKNEKEKSEDKRYMRFGRAGYMRFGRSEGNDAEKKAGYMRFGKKNDYLRFGRESAEDSEHNDEKRYMRFGRSEADKRYMRFGRAQEKVDANTRHTQFDPSEDDEEKRYMRFGRSEASESKDKRYMRFGRSDANDKDKRYMRFGRSDADEKDKRYMRFGKRLVHKRSIRSADQETPIERLQ

>FMRFa_Nemertea_Nipponemertes_spec_TRINITY_DN15377_c0_g1_i1_m.42765 internal

EQRERATPGGYLRFGRSGDGYIRFGRYLDGEDDDLSPVKRYMRFGRPDYLRFGRLTDEDETGEPSKRYMRFGRYMRFGKDIRETPGGYLRFGRDNRVTRFGRELGER

>FMRFa_Nemertea_Malacobdella_grossa_comp46859_c0_seq1_m.149910 internal

SGHYGRDFPRFVSPVDVEDKTTDDDEGKIGMKGGLSSPIDNDKRYMRFGRSGGEILDDNALQTDTDGGDVDTKRYMRFGRAHENEKRYMRFGRSVEDPKDFIPSGSHPKEATRRKRSVDDNNDDLTATEQGQRFINDRLLS

>FMRFa_Nemertea_Malacobdella_grossa_comp149321_c0_seq1_m.152439 5prime_partial

LQKEIPATIYQNLGKRYMRFGRANYLIFGRLSDDDDELQKRYMRFGRFMRFGKNIDSSAYRRYNRDSGLLRLSRQLAQREPAAGYIRFGKK*

>FMRFa_Nemertea_Cephalothrix_hongkongiensis_comp113981_c0_seq1_m.98382 internal

DGSDMDAEKRYMRFGKKDGSDQKEEDKRYIRFGKRDNEDEKEENKRYMRFGKRYIRFGKRYMRFGKRDGQVDEDIDEEKRYIRFGKKDGSDMDAEK

>FMRFa_Nemertea_Cerebratulus_marginatus_comp1922716_c0_seq1_m.162457 5prime_partial

DGEDSDEAEETPDKRYMRFGRDYLRFGRLLDGEEESSSDNQNTVQKRFLRFGRLFGGLGVSKEELQRRYLRNNRDRAGGYMRFGRSEPTAGYMRFGK*

>FMRFa_Nemertea_Lineus_lacteus_comp17427_c0_seq1_m.28399 3prime_partial

MYILYLVALLAGQAIATDFNALCSDPKLNSLKNFMVLCNAFRSFSDQFNDVSSDSKRHRETFIRYGRDASHVSQEGALETHLTTPSKRSPEGYMRFGRSVKDTKPAQSVENVDVENTKKEEEKKAIPSTAVDSEQKQETTAEDDKAKRYMRFGRNDYIRFGRESEEADDDAIDDAKRYMRFGKKDDAKRYMRFGKKSLGPVAEGEGEEQKRYMRFGKKDGSDDAEKRYMRFGKRYM

>FMRFa_Nemertea_Lineus_lacteus_comp22954_c0_seq1_m.57517 5prime_partial

FGKRDDDLDEDKRYMRFGKKSDDEKRYMRFGKKDGEEQDEKRYMRFGRGDQEMDADKRYMRFGKRYMRFGKRYMRFGKRDDEDATEGGDDEKRYMRFGKRYMRFGKRDMSTDEERKRNVRSVDESKAEEVTHMRYRRSEGPDGFERDARYMRFGRNPNGATVDGYIRFGRLLDGEDSDETAETPEKRYMRFGRDYLRFGRLLDGEEESSNDNQNSSPVQKRFIRLGRLFGGLGVSKEELQRRYLRNNRDRAGGYMRFGRSEPAGGYMRFGK*

>FMRFa_Nemertea_Cerebratulus_spec_TRINITY_DN31043_c0_g1_i1_m.21286 internal

KRYMRFGKRYMRFGKRDDEIEEDKRYMRFGKKSDDDEKRYMRFGKRDGEDQEEKRYMRFGKRSDEDEKRYMRFGKRYMRFGKRDDEIEEDKRYMRFGKKSDDDEKRYMRFGKRDGEDQEEKRYMRFGK

>FMRFa_Nemertea_Cerebratulus_spec_TRINITY_DN31043_c0_g3_i1_m.21289 3prime_partial

MYFFYFLALLAGQAIATDFNALCADSKLKSLQNFMVLCDAFRSFSQSLNDVSLDSKPQRETFIRYGRDASHVSQENAIDHQSHLTTPSKRHAEEGYMRFGRSVKEQEPAQNAQDIENVKKEEQKKAIPSTAEDSDQKQEAAQEDEKAKRYMRFGRNNYIRFGRESEENDDDEIDEAKRYMRFGKKSAEEAKRYMRFGKKSFGPVSEEEGDEQKRYMRFGKRDDEAEKRYMRFGKRYMRFGKRDDEIEEDKRYMRFGKKSDDDEKRYMRFGKRDGEDQEEKRYMRFGK

>FMRFa_Nemertea_Cerebratulus_spec_Contig256_m.194107 5prime_partial

KRYMRFGKRYMRFGKRDDEIEEDKRYMRFGKKSDDDEKRYMRFGKRDGEDQEEKRYMRFGKRSDEDEKRYMRFGRSDDKRYMRFGKRYMRFGRGGADDSEAGDMEKRYMRFGKRPYMRFGKRDTSAAEAQTRIARSVDSKTDDASHKRFRRSDEFERDARYMRFGRNPNGATVDGYIRFGRLLDGEDGDGTEETPDKRYMRFGRDYLRFGRLLDGEEESSSDNQNTVQKRFLRFGRLFGGLGVSKEELQRRYLRNNRDRAGGYMRFGRSEPTGGYMRFGRSEPTGGYMRFGK*

>FMRFa_Nemertea_Notospermus_geniculatus_g37951.t1

MRFGRSVKEIEPAQNTPNVDKVKKAEEKKAISSTAESTDQNDKAAQEAEAEKRYMRFGRNNYIRFGRESEEDEDDDDDNEIDEAKRYMRFGKKSAEEAKRYMRFGKKDSGMGPVVDEDGEEQKRYMRFGKRYMRFGKRDGEDDLDEDKRYMRFGKKAEADEKRYMRFGKREEEEKRYMRFGKRDEGEEKRYMRFGKRYMRFGRDSTDGSESGNDDKRYMRFGKRYMRFGKRNAEAAEGRTRVARSVDATKRFLRFAREAPSEGSEDFERAARYMRFGRNPNGATVDGYIRXIWRRVTRSSPFYDFLGRGRTF

>FMRFa_Nemertea_Notospermus_geniculatus_g2365.t1

MEPNAPPPXXXXTFIRYGRDASHASQDNALEERHLTTPSKRDTGGYMRFGRSVKEIEPAQNTPNVDKVKKAEEKKAISSTAESTDQNDKAAQEAEAEKRYMRFGRNNYIRFGRESEEDDDDDDDNEIDEAKRYMRFGKKSAEEAKRYMRFGKKDSGMGPVVDEDGEEQKRYMRFGKRYMRFGKRDGEDDLDEDKRYMRFGKKSEADEKRYMRFGKREEEEKRYMRFGKRDEGEEKRYMRFGKRYMRFGRDSTDGSESGNDDKRYMRFGKRYMRFGKRNAEAAEGRTRVARSVDATKRFLRFAREAPSEGSEDFERAARYMRFGRNPNGATVDGYIRFGRLLDGEDSDDVDETPDKRYMRFGRDYLRFGRLLDEDEPSSDNQDSNIQKRYLHFGKLFGGLGVSKEELQRRYLRNNRDNRGGYMRFGRAQPGGYMRFGK

>FMRFa_Nemertea_Lineus_ruber_Lvir.rna.tri.19128.1_m.39295 complete

MYILYLVALLAGQAIATDFNALCSDQKLSSLKNFMVLCDAFRSFSDSLNDVSLDSKRHREASVRYGRDASHVSQENALETHLTTPSKKTREGYMRFGRSVEDPQPAQNVENVDVEKSKKEEEKKAISSTAGDTKQKQETTAEDDKAKRYMRFGRNNYIRFGRESEEADDDEIDDAKRYMRFGKKDDAKRYMRFGKKSLGPVAEEEGEEQKRYMRFGKKDGSEGGDDAEKRYMRFGKRYMRFGKREGDLDEDKRYMRFGKKSDEDEKRYMRFGKKDGDDQDEKRYMRFGKKSDEDEKRYMRFGRGDEEMEANKRYMRFGKRYMRFGKRDEEDATEGGDDEKRYMRFGKRYMRFGKRDTPTDEVRKRNVRSVDESKAEEATHMRYRRSEGRDEFERDARYMRFGRNPNGATVDGYIRFGRLLDGEDSDKTDETAEKRYMRFGRDYLRFGRLLDGEEESSNDNQNSSPVQKRFIRLGRLFGGLGVSKEELQRRYLRNNRDRAGGYMRFGRSEPAGGYMRFGK*

>FMRFa_Nemertea_Lineus_longissimus_c40291_g1_i2_m.58287 complete

MYILYLVALLAGQAIATDFNALCSDPKLNSLKNFMVLCDAFRSFSDNLNDVSLDSKRHRATFIRYGRDASHVSQDNALETHLTTPSKRNTEGYMRFGRSVKDPQPAQNVENVDVENTKKEEEKKAIPSTAVDSEQKQETTAEDDKAKRYMRFGRNNYIRFGRESEEADDDEIDDAKRYMRFGKKDDAKRYMRFGKKNLGPVAEEEGEEQKRYMRFGKKDGSDDAEKRYMRFGKRYMRFGKREDDLEEDKRYMRFGKKSDDEKRYMRFGKKDGEEQDEKRYMRFGKKSDEDEKRYMRFGRGDEDMDADKRYMRFGKRYMRFGKRYMRFGKRDEEDANEGGDDEKRYMRFGKRYMRFGKRDTPTDEVRKRNVRSVDESKAEKAAHMRYRRSEGPDGFERDARYMRFGRNPNGATVDGYIRFGRLLDGEDSDETEETPAKRYMRFGRDYLRFGRLLDGEEESSNDNQNSSPVQKRFIRLGRLFGGLGVSKEELQRRYLRNNRDRAGGYMRFGRSEPAGGYMRFGK*

>FMRFa_Nemertea_Tubulanus_polymorphus_comp36563_c0_seq1_m.55247 complete

MVSLHILLFILCQTYALDVSSLCNHPKYSQVIVKLCDAFGIMGREEILNDVHMLRQPKRNRGPYVRFGRDVSNQQTPKEAQSDTSNTADNTVKGYLRFGRSVDEQHNIQKREAETPTKSDNTAILEKDGDTSKRYVRFGRSEKRYMRFGRSNDESKEDLKKRYMRFGKKSSIENEKKYMRFGKKDGSDIDNDKRYMRFGKRDDLDEAKRYMRFGKRDTQDEEKRYMRFGKRSDADNDGDDHSKENGDDTDELEKRYMRFGKRYVRFGKRNDYLRFGEPQEKRYMRFGRNNDYLRFGQAQSKRYMRFGRSGDDVDDEDNGSGDAEKRYMRFGRSGDDKRYMRFGRNGDYLRFGNEKRYVRFGRSDNNDQDEEKRYVRFGKSVEQSGNPDQDKDSEKKRYVRFGRAANAEHVETENSPSKKRVVRSASDQHTRVKRHVDENEEDEIDRLARYLKLSARDKALGDTIGGYIRFGREEDDSQEEDGDDADYGIDKRYVRFGKRYVRFGRSPSSLFSLFRGSRGYPRYIRNSSGSPVGYIRFGR*

## F[V/L/I]amide

>F[V/L/I]amide_Phoronida_Phoronis_ijimai_(P._vancouverensis)_comp88845_c0_seq1.p1

MFSTAAVLRSELKLVGLVVLLFQLSSSSPTSEDRSGSVILQDMIPRSPIFIGRRSEPSSRTIKSFIEDLEKGELPADILLGQSLEKVPLTEDRDADNEWIYGAGERLKSLAGKRRGNFIGKRHNFIGKRGRKNFIGKRSSDTVSERLRFVPIEPPVDSAESYEKSPIFIVGDNESLEKLKKMFLIDGPNKDSEIHNASERDSNRHSKQLDASDSSIWKGLFPSDNL*

>F[V/L/I]amide_Phoronida_Phoronis_australis_TRINITY_DN299273_c0_g1_i1.p1

MFSFVAVLRSELKLVGLAMLLLQFSSSSASPEDRSTPIVLNDMIPRSPIFIGRRSGESSRTIKSYIEELRKRVQDLPADVFLGRALDRLHMTENSAADNDWIYSAGDRPKSLIGKRRGNFIGKRHNFIGKRGRKNFIGKRAPDIVGERLRFVSVEPPVDSTESYEKTPIFIVGDNESLEKVKKIILNDDTTKDSEIPNASERALNQEDDRHDSSDSSMWKGLFPSDNL*

>F[V/L/I]amide_Phoronida_Phoronis_psammophila_comp730754_c0_seq1.p1

GNAYPRSPLFLGRRSGGLARDSFIHKFPIDNEMKNSPSNFAPPTYRLPKPRFPIDIDDGAYSLVVGKRAKDFIQKRRGNFIGKRQNFIGKRRGSFIGKRSSLDSADILKIPHVKFIPVITQSDS

>F[V/L/I]amide_Brachiopoda_Novocrania_anomala_Nano.rna.tri2.48459.1.p1

MTWTMKTQVHCVMVLAMCLGDIGIALSSMSTDKRAIFSEQSPRYIGKRPQFIGKRPQFIGKRPQFIGKRHGSFEKRPQFIGKREYTGKEEIIEKRPQFIGKRPLMGDEPEILEKRPQFIGKRPSFGTEPDFVDKRPQFIGKRDYFTEPMHAEKRPQFIGKRPQFIGKRPQFIG

>F[V/L/I]amide_Brachiopoda_Novocrania_anomala_Nano.rna.tri2.43476.1.p1

GKRPQFIGKRPQFIGKRPQFIGKRPQFIGKRPQFIGKRLTENEQNITPERLAARLRAAMDDLILRHTERGKNQAYFPHIYDKIGYTTPLRIPQQNM*

>F[V/L/I]amide_Brachiopoda_Novocrania_anomala_comp63653_c0_seq1.p1

MTWTMKTQVHCVMVLAMCLGDIGIALSSMSTDKRAIFSEQSPRYIGKRPQFIGKRPQFIGKRPQFIGKRHGSFEKRPQFIGKREYTGKEEIIEKRPQFIGKRPLMGDEPEFLEKRKK

>F[V/L/I]amide_Brachiopoda_Novocrania_anomala_Nano.rna.tri2.13020.1.p1

MTNTTPATLFLLLCFAVFIASEKEGVDLPEQKGALTEKHDLSEGTDAVNTPKTKADVNLSNIKNFGVNIDAVTTEYIQRCLSLLNEREENQRGLDKRRNRFFGKRLKENRLEKEHGDVDGAREKSHGGLDKRRNTFFGKRQNVYPLGKGNKDVDDTDKMDDAGSVTLTNVHDSLWPCTAFLKLTSLFREELSALQHHNFGDVLTKPNGNSSENRAIRDTSIYSDVPDYLLHCLTIWDQSGEDTDLIEQAKRRGYIGKRRDDGTDLVDESKRRGYIGKRRNGFIGKRRNGFIGKRRTGYIGKRQVSTDTWNPELELKDKICLLLQEKGLSQEVKNLAELVAPKYESNNMAHNKLFDWFTKYVLENDSTDSIDHDDKRDGGHQVFRGRQTNRGRQRYMGRRK*

>F[V/L/I]amide_Brachiopoda_Lingula_anatina_comp137267_c0_seq1.p1

MALVKGWTLLSLLAVMANCTALRKKDTDSGLKAIGKDVTDEGIREPVEYGIFVKNPDTDDTDGIASWLLTYDPVVIVDVAKDGMDEDVDGDLNVDSEDVDDRNASEKRRFKGGVRAYMLNMFRPGSSIRNNPAFLGKRDWNQHETGSSQISVDEGTRRPRFFKKRGSRMQAYLANLMRSQTAGKHNPAFLGKRQVEVPGIQTIVPQDIALQKPRFIGKRDVQDYVQSFFRSKPSTRQKPGFIGKRAAKSRSKRDLKSYVSELFRAKQGNRQKPRFIGKRDVRSYLQNMFRSYSNAKNKPRFIGKKSGDMSAFLHNMFRSQSSDRNKPSFLGKRDELTTTAAGDDMALSTSDKRSNQNKPRFLGKRNS*­

>F[V/L/I]amide_Brachiopoda_Lingula_anatina_comp124454_c0_seq1.p1

MHPPVLFCFVCAQVLVTFSLETLPVVSSLQQLPYQPQDDAKHADDISTNDENMLEDLEGIFFGREPPEQHHSLYPVVNQDTFGLYQRFDKRRDFMGKRNAEMSKRNEALAKRRDFIGKRRDFMGKRRSFMGRRSEAEDFPKSRSLSGKKSGDDAYALLLKKNRGFMGKRTRGFMGKRINFQVTEDTGFDTNGENTSEGRCRTNQDCSTDASCCTLYLPWLATTQCLPRGNEGDVCVPEHLHKPTEHYFCPCLQGLRCDVIGDSTDRGICIRT*

>F[V/L/I]amide_Brachiopoda_Lingula_anatina_comp130202_c0_seq2.p1

DDDDDDDKEDASPNGFEKRRFLGKRLDEDDDDEKSDDPEKRRFLGKRFNDDANPEDDEKRRFLGKKSDDDTDIDNEKRRFLGKRRFLGKRRFLGKRGPYEEDKRGRFLGKRLDSEKDYLLDKLKRAYQSIFPQADFLRNSADELDKRRFLGKRRFLGKRRFLGKRSAEQKRGDMLNSGQSKIHSIKKRSIMWHVQPDPSTNLFLGYPRQVMLLSNGYPSGMNPYMKRQDVLIPRWPQPDTQRVPHFIG*

>F[V/L/I]amide_Brachiopoda_Lingula_anatina_comp130202_c1_seq1.p1

MMLEVRIPWLIALFVVFLSIPALTEKTKHTQRDSIESEALWHSDVPHSEYGSKHGLGYKDDTGNPHSELPDTKLKRRFLGKKSDHDLTEKRRFLGKRFGDNMSDDPDKRRFLGKRFQDTISPDPEKRRFLGKRFGDTSYPADAEKRRFLGKRFDDDDDDD

>F[V/L/I]amide_Brachiopoda_Laqueus_californicus_comp6­-6_c0_seq1.p1

SALPTGSLISDTQGQGSPTQGEESTQLLRVAIYAIHPVPLDYFNAAKRREFVGKRREFVGKRREFVGKRREFLVKRHSSQNNDDSYMSQLPIRRTLRVLVDRNNIDDIYK

>F[V/L/I]amide_Brachiopoda_Hemithris_psittacea_comp20406_c0_seq2.p1

MRFLIFGGVCICLLLLGPDAVNSIGKQTFQEITEQKDFSVGLDTEDDELTKSNEEVQRYPNLLSKLAPAWKRREFVGKRRREFVGKRREFVGKRYYDENSWATESRLKGFLRQLIERRELDPLETMQPIQDDKRREFVGKR*

>F[V/L/I]amide_Brachiopoda_Hemithris_psittacea_comp20406_c0_seq1.p1

MRFLIFGGVCICLLLLGPDAVNSIGKQTFQEITEQKDFSVGLDTEDDELTKSNEEVQRYPNLLSKLAPAWKRREFVGKRRREFVGKRREFVGKRREFVGKRREFVGKRYYDENSWATESRLKGFLRQLIERRELDPLETMQPIQDDKRREFVGKR*

>Brachiopoda_Glottidia_pyramidata_comp44893_c0_seq8.p1

HYGRGLFCFVCAQVFVTFSLETLPDVTSLQQSPYQSQGDVKRADDIDRNADDSMLEDFSKRRSFMGKKSDGDAYERLLKKNRGFMGKRTRGFMGKRINFQVTEDTGFDTNGDSVVRCQTDADCDAETSCCSLYLPWLATTQCLPRGGEGEVCVPGRLHKPTKHYFCPCQQGLRCDAMADSEDRGICIRP*

>F[V/L/I]amide_Brachiopoda_Glottidia_pyramidata_comp44893_c0_seq5.p1

MFSQSLFCFVCAQVFVTFSLETLPDVTSLQQSPYQSQGDVKRADDIDRNADDSMLEDFSKRRSFMGKKSDGDAYERLLKKNRGFMGKRTRGFMGKRINFQVTEDTGFDTNGDSVVRCQTDADCDAETSCCSLYLPWLATTQCLPRGGEGEVCVPGRLHKPTKHYFCPCQQGLRCDAMADSEDRGICIRP*

>Brachiopoda_Glottidia_pyramidata_comp44893_c0_seq4.p1

HYGRGLFCFVCAQVFVTFSLETLPDVTSLQQSPYQSQGDVKRADDIDRNADDSMLEDLEGIFFGNDLDESPRAPRSLYPDLNQGALGDLYQKYDKRRDFMGKRRSGMSKRKDAIAKRRDFMGKRRDFMGKRGSYVDRQGVDGDAKRRSFMGRRRSSGDEDFSKRRSFMGKKSDGDAYERLLKKNRGFMGKRTRGFMGKRINFQVTEDTGFDTNGDSVVRCQTDADCDAETSCCSLYLPWLATTQCLPRGGEGEVCVPGRLHKPTKHYFCPCQQGLRCDAMADSEDRGICIRP*

>F[V/L/I]amide_Brachiopoda_Glottidia_pyramidata_comp44893_c0_seq1.p1

MFSQSLFCFVCAQVFVTFSLETLPDVTSLQQSPYQSQGDVKRADDIDRNADDSMLEDLEGIFFGNDLDESPRAPRSLYPDLNQGALGDLYQKYDKRRDFMGKRRSGMSKRKDAIAKRRDFMGKRRDFMGKRGSYVDRQGVDGDAKRRSFMGRRRSSGDEDFSKRRSFMGKKSDGDAYERLLKKNRGFMGKRTRGFMGKRINFQVTEDTGFDTNGDSVVRCQTDADCDAETSCCSLYLPWLATTQCLPRGGEGEVCVPGRLHKPTKHYFCPCQQGLRCDAMADSEDRGICIRP*

>F[V/L/I]amide_Brachiopoda_Glottidia_pyramidata_comp33987_c0_seq1.p1

MSPEIRIPWVLALVMVSLPIPAITEKMTQTQMGATETKVPWIYEVSHPDHKTTEFIGTRQEGTGAPHSKLPDARLKRRFLGKKSDQDIKEKRRFLGKRYDDNMPDDQEKRRFLGKRYDDDSSTMDEKRRFLGKRQDGETEARYAEKRRFLGKRFDDQFADDIDERERLIGKSTNEEKRRFLGKKSYDDNNQENDKRRFLGKKNYDDSKREDEKRRFLGKKEKSTD

>F[V/L/I]amide_Brachiopoda_Terebratalia_transversa_Ttra.rna.tri.12501.1.p1

MHKLYNNKLVYLKQLFHCCCLLLTASTECAYIRSEYNTPSPIVEEMQGSRGRGSSPTGSLFTRHTPRVQFIPVAIYAIQSVPFDMHYFNVDKEKRRFVEKRREWAGKRREFAGKRQYSENVGAYVDSYLSNLPNRMFHVLFDRNHFDGKFHETKDNEKRREFAGKRREFAGKRDKKLHTVVNWF*

>F[V/L/I]amide_Nemertea_Cephalothrix_hongkongiensis_comp399253_c0_seq1_m.115623 internal

VLRVINLCLVFADKRAPSFLGKRDELEEIYRDLRNPALKDDEYWGDEIEDDKRAPSFLGKRAPSFLGKRADEEMDLDEEKRAP

>F[V/L/I]amide_Nemertea_Baseodiscus_unicolor_TRINITY_DN103315_c0_g2_i1_m.101019 3prime_partial

MLNKEDFVQRVQELMEHNTGLLGDEMDAEKRRHFLGKRDGSADVELSGDKRGRPLFIGKRGPHYFIGKRRPSFIGKRRPSFIGKRRPFFIGKRDDGDNDQFESMNDGNDLAQILDKIMAQTADESRLD

>F[V/L/I]amide_Nemertea_Malacobdella_grossa_comp2037_c0_seq1_m.937 3prime_partial

MRSSVDHNLFVAFVALTSLCCALAAERTERDTNDEDKRNRAFLGKRDDMGETEMAKRAHRFLGKRQDGLKKEKS

>F[V/L/I]amide_Nemertea_Notospermus_geniculatus_g23645.t1

MRSQDILVFCFVLGSCLVSSTLSETADKRARNFIGKRDPADDLKTADEKSLNQDDFVKRVQEFIDHNDFLDNDLNEDKRRPSFLGKREDDMEEEKRRPSFLGKRARNFIGKRSHRFIGKRGRLFLGKRGRLFLGKRDDDEDFDTLDAEKRRHFLGKLRTDYHTDVRKKNVYNKR

>F[V/L/I]amide_Nemertea_Tubulanus_polymorphus_comp26466_c0_seq1_m.16228 5prime_partial

MKHTWKVLMVTLVAILAEVMIMSNASEESLPNPADDDDRQLSNLESELYEKYLNQLANSGQLDTEDNAIDFYDDEPDFMDKRRRPGFIGKRRPSFLGKRRPSFLGKRRPSFLGKRIPSFIGKRILPLFGGKRRPRFLGKRPRPGFIGKRRPSFIGKRADDSEYLNVPQQTRDMFPADQSLLIKRSASHQENNNENTNHETTSSEIDQTKRRPSFIGKRSQNSEH*

>F[V/L/I]amide_Nemertea_Tubulanus_polymorphus_comp27782_c0_seq4.p1

MMKAHELFAFLLLATLQLQNVLTDDELTKRQHDFLGKRNEISHLSKRSAELDGSHTINKRGTREFLGKGTILDNDDFNERRQLYKRRRDFLGKRVADDFDSYEDFEATKRTRDFLGKRTRDFLGKRARDFLGKRAHNLFGKRITFAQDCITDNQCGGDQCCVLTAFGIRKCRGLQDYGEKCHPNSMKAPSDTRRSFSLCPCMSHQYCSKTKYICV*

>F[V/L/I]amide_Nemertea_Cerebratulus_marginatus_comp60160_c0_seq2_m.79294 complete

MRSQDIIVFCFILGSCLVNVYSEAEEKRGHKFLGKRDSADTLNNEDAKLNGDDFVKRVQEFIDQNDFLDDELDEDKRGRPNFLGKRDDELEEEKRRQNFLGKRGHYFIGKRGPRFLGKRGRYFLGKRGRYFLGKRDGDDEDIDTFDEEKRRHFLGKREDLDESDFGDLEEEMEKRRHFLGKRVSRNFIGK*

>F[V/L/I]amide_Nemertea_Lineus_ruber_Lvir.rna.tri.14419.1_m.30478 complete

MRSQDILVFCFILGSCLVSSAYSDSADKRVRNFLGKRDSADTKNEDNKAVLNDDDFVKRVQEYMEHNDFLDDGLDEDKRGPSFLGKRADDIDEEKRRHSFLGKRRPSFLGKRGPHFLGKRGGRYFLGKRGRYFLGKRDGDDEDMDTIDEEKRRHFLGKREGLDDSELGELEDEMEKRRHFLGKRVSRHFIGK*

>F[V/L/I]amide_Nemertea_Riseriellus_occultus_Contig14112_m.140837 5prime_partial

LWSIYSWTLLTNSSLFNADKRGPRFLGKRADDIDEEKRRHSFLGKRRPSFLGKRGPHFLGKRGRYFLGKRGRYFLGKRDGDDEDMDTFDEEKRRHFLGKREDLDDTEFGDLEDEMEKRRHFLGKRVSRNFIGK*

>F[V/L/I]amide_Nemertea_Lineus_lacteus_comp22889_c1_seq2_m.57006 complete

MRSQDILVFCFILGSCLVSTAYSDSPDKRVRNFLGKRDSADTNTEEEKSALNNDEFDKRVQEYLDHSDFLDDGLDEEKRSPRFLGKRADDIEEEKRRHSFIGKRLPSFLGKRGPHFLGKRGRYFLGKRGRYFLGKRDGDDEDMDTFDEEKRRHFLGKREDLDDTAFGDLEDEMEKRRHFLGKRVSRNFIGK*

>F[V/L/I]amide_Nemertea_Lineus_longissimus_c47113_g1_i1_m.123965 complete

MRSQDILVFCFILGSCLVSTAYADSNDKRARNFLGKRDSADTKNEDDKSVLNNDDFVKRVQEYLDHNDFLEDGLDEDKRGPSFLGKRADDIDEEKRRHSFIGKRRPSFLGKRGPHFLGKRGRYFLGKRGRYFLGKRDGDDEDMDTFDEEKRRLFLGKRADLDDTEFGDMEDEMEKRRLFLGKRVSRNFIGK*

>F[V/L/I]amide_Nemertea_Paranemertes_peregrina_comp370329_c0_seq1_m.180317 5prime_partial

PLRPGGKRLEYDEKRNRAFLGKRNRSFLGKRNRSFLGKREDDLFDEEGKRNRAFLGKRYYEDDDGDDDYIKRNRAFLGKRAHRFLG*

>F[V/L/I]amide_Nemertea_Paranemertes_peregrina_comp140_c0_seq1_m.74 3prime_partial

MKTSIDHVFLVLVAITSLCCVLSEERTKRETDNEDKRNRSFLGKRADIDELELDKKAHKFLGKREDDLKTEKRMRSFLGKRYDEDQLQKRLRAFLGKRAHDFLGK

>F[V/L/I]amide_Nemertea_Nipponemertes_spec_TRINITY_DN26283_c1_g1_i1.p2

YTSSLSTSSRNRAFLGKRFYDDDDDDVDFEEKRSRAFLGKRAHAFLGKRAHRFLGKR*

>F[V/L/I]amide_Nemertea_Nipponemertes_spec_TRINITY_DN26283_c0_g2_i2_m.42098 3prime_partial

MLGRAVRRAFLGKRDDDDLDLDKKARAFLGKREDDDLEEKRARAFLGKRMDDDLRKRLRAFLGKRAHAFLGKRDDDKRAHAFLGKRDDDKRAHAF

>FSE_Nemertea_F[V/L/I]amide_Nipponemertes_spec_TRINITY_DN30785_c0_g4_i1_m.98736 5prime_partial

LTHEFAEWSLADEFTERSFSQEFIETSFSYEFVGKRPFSEFVGKRDDDKRAFSEFLGGKKRDAQ*

>F[V/L/I]amide_Nemertea_Cerebratulus_spec_Contig5994_m.214514 complete

MRSQDLLVFCFILGSCLVSSAYSETVEKRPHNFLGKRDSADNLNNDEDKLNGDDFVKRVQEFLDRNDFIDSDLDEEKRGPKFLGKRDDDLEEEKRRQNFLGKRGHYFIGKRTPRFLGKRGRYFLGKRGRSYFLGKRDGDEEDMDTFDAEKRRHFLGKREDLDESEFADLEDEMEKRRHFLGKRPSRGFIGK*

## FXRIamide

>FxRIa_Phoronida_Phoronis_ijimai_(P._vancouverensis)_comp96084_c0_seq1.p1

MTMAAKELCLILCSALILTATAEANIMTVCMARCEAHLSTRLCNHACADMVARLEGPGVPLVIKSPSSDFDRPEDKNERKKREIRLLTGDKYWKWQWYADAKRTGRRHSFFRIGRNGKQVGNIDVVQPYENLKSSSHGDLNDQLGSTIENLAYQLAIMDQNDYEQTSDESVDRLKAISHVLPDIMEAPTLKRARQRSRFMRVGRDTSAVMALMDGQNELQKRRSRFLRVGKSDGSVPMDQRRNYVERKDNEDNGFTDESDGEHLGSFTGNGSVMSEKSKRRNRFLRVGRGKKSPTNGIKVDDEIDDNAPLSYEKRRNRFLRVGRMENPDDRDKQEEKRRSRFLRVGRMKGENVENEATSKEKRRSRFLRVGRKNDESENEGNNSGKMTSADKRRSRFLRVGRGNDIGNLFDGKVTENDEKRRSRFLRVGRGDSLQMSPEFEKRRSRFLRVGRQPGGSIMDLEEKRRSRFLRVGKATDSDSLNENPEQSRHRFAYVKSDENAIEKRRSRFLRVGRGEIANDSHRKQLSSRDQAITHYNAKEIDGGVKKGDMKNMTASKTLEIQNKMARLTNSSSSMAENTDNVAARYTKA*

>FxRIa_Phoronida_Phoronis_psammophila_comp59234_c0_seq1.p1

MRSEVMGTPLMLVFVMYVTIDASVADDMSECVSSCNARGVVSMEQCNSACSQYISKYRRGGMPFPVNSYRKWLWETGFGRHQGERQMDTSDKLNRNRFGFFPPVDEMMSRVELEATVDELARQMSSPSQQQNKQYIDYDLERLHAVSPVLPDFMTARHEKRYPTRSRFLRLGRDTSAVERRLKQQNDDREKRRSRFMRLGRGKMDDDVSAEKRRNRFMRVGRPFDESSENSIENEKRRSRFMRLGRGGEDKMIDLDTGYFDGKTASRIGHFGHDFSSDEDSPVEEKR

>FxRIa_Phoronida_Phoronis_psammophila_comp54173_c0_seq1.p1

VAITPGTPSRIAHFGHESSSDDDSPVEEKRRNRFMRVGRPSDIKNKAEKRRNRFMRVGRPSEIEHAAEKRRNRFLRVGKSMDPSDIIEEQKRRNRFMRVGRGDGDENERDIASLERKLSLLSGMPLERNGGGTNPEALPEVVEEKRRNRFMRVGRGDIEHSDDGSVSKRRNRFLRVGRGHVVKNIQIEPANSGKEKDVFEEKTGSDLNSSLEKSKTT

>FxRIa_Phoronida_Phoronopsis_harmeri_c72926_g1_i1.p1

IDQYLNAAKRRSRFMRVGRPDEGYEDQGSEMNQQKRRHRFMRVGRDPSENGYEKRRNRFMPVGRGTSSQEESDLANTSDMEKRRSRFMRVGRGDNLNLDGKSRKIESVLKDFGDSLRNPTKDSETSNSPDSTTQPSIAKIPSSQKLNN*

>FxRIa_Phoronida_Phoronis_australis_TRINITY_DN315964_c0_g2_i2.p2

MSTETEKRRNRFLRVGRQKESSILKLNQKRRNRFLRVGKATDSGLLNESPEPTRHEFAIVDSDENATAKQSGVGLWAISNDSDGSQLSRGQAATHVNAKEIDSDVKKGQSTKHGTESNEIQNNMAQFTNMAKGASRLVIHHAAAGNTKA*

>FxRIa_Brachiopoda_Glottidia_pyramidata_comp21248_c0_seq1.p1

MSTQREVLLSVLFFGLLFAQQLTLAADTDTDTNEECSKKCSDDFDKVECLKICVSKTNSSEDRGDDEKRGAFWRIGRGDDKKSAFWRIGRRNDDSSDNDNVKKSAFWRIGRDPNKKSAFWRIGRANNDNDESKKSAFWRIGRGDQADEPDKKSAFWRIGRRNDDSSDNDNVKKSAFWRIGRDPNKKSAFWRIGR

>FxRIa_Brachiopoda_Glottidia_pyramidata_comp36381_c0_seq1.p1

GHYGRGSAFWRIGKKSAFWRIGKRENGDAEIEDDKRSAFWRIGKRNKEEDAKRGAFWRIGRDGANSHKEEDEKKSAFWRIGKKSGHHLQKRDTPFWRIGRSASSLEPLSGFVDDVDDLRQGLNLDDGAEKRSTFWRIGKRPNTFWRVGRSNDKRTFWRIGKRDPSFGDEESDKRMNTFWRVGKSYPSDLDHDIMLRGFWRIG*

>lingulaAnatina.g16066.t1 Brachiopoda

MPAQREVLFSIIFFVLLFAQQLTLAADTDTNEECSKKCSDDFDRIECLKICVSNSNSTENDDDDEKRSAFWRIGRGDDKKSAFWRIGRGGEDMTDSDNERKKAFWRIGRDPNKKSAFWRIGRSDNDNDESKKSAFWRIGRADQEDAPQKKSAFWRIGRSDPFDKRGAFWRIGKKSAFWRIGKKEDYGSEHDDDDKRSAFWRIGKRNDDKDMKKSAFWRIGRNEANSHTDDEDKKSAFWRIGKKNGHHIQKRDTPFWRIGRSVSPISPGFFDDSDDWRDGLVVGDDDDKRSTFWRIGKRPNTFWRVGRSNDKRTFWRIGKRDPYGDEDPTKRLNTFWRVGRSFPADTDHDLQIRSFWRIG

>FxRIa_Brachiopoda_Lingula_anatina_comp140938_c0_seq1.p1

MCFFIDYYFRNTRAKLNMPAQREVLFSIIFFVLLFAQQLTLAADTDTNEECSKKCSDDFDRIECLKICVSNSNSTENDDDDEKRSAFWRIGRGDDKKSAFWRIGRGGEDMTDSDNERKKAFWRIGRDPNKKSAFWRIGRSDNDNDESKKSAFWRIGRADQEDAPQKKSAFWRIGRSDPFDKRGAFWRIGKKSAFWRIGKKRGLW*

>FxRIa_Nemertea_Riseriellus_occultus_TRINITY_DN72213_c0_g1_i1_m.2948 3prime_partial

MRIGRGFMRIGKSVPYGMKRPSFMRIGKRSLDDNDDKRGFMRIGKSGESDKRGFMRIGKSMEDDKRNFMRIGKSDSDKRASFMRIG

>FxRIa_Nemertea_Riseriellus_occultus_TRINITY_DN67067_c0_g1_i1_m.102921 5prime_partial

DEDEKRGFMRIGKKSDKRASFMRIGKKSDDEAEKRGFMRIGKSDSEKRGFMRIGKSAELVNDEAMADNAEEKRNFMRIGKKSDDTSEPGASFLGNNLDSEDDEAKRASFMRIGRSV*

>FxRIa_Nemertea_Cerebratulus_marginatus_comp763084_c0_seq1_m.127895 5prime_partial

HCHSFMRIGKSSEEAKRGFMRIGKKDVDEEKRGFMRIGKKDDDKRASFMRIGKKSDEEMDDEEKRGFMRIGKKDDDKRGFMRIGKSSELINDDALADNISEDKRGFMRIGKKSDDSDKRASFMRIGKSMDSAEEKRASFMRIGRSV*

>FxRIa_Nemertea_Cephalothrix_hongkongiensis_comp7490_c0_seq1.p2

AFMRIGKKYDVSDSLDEEKRAAFMRIGKKDSDKRAAFMRIGRGNEDADKRAAFMRIG*

>FxRIa_Nemertea_Cephalothrix_hongkongiensis_comp22098_c0_seq1_m.12666 3prime_partial

MTKWITILLTIALTCLSHASELTCPELCQMLDDEVIDTDECVSNCNDEVKRGAFMRIGRAQFMRIGRNADDDSSDSFERYSRPSFMRIGRAAFMRIGKKSYYKRPAFMRIGKRSDEAVRRAAFMRIGKKDDEIVDEAQKKAAFMRIGKKDDAEKRA

>FxRIa_Nemertea_Nipponemertes_spec_Contig14657_m.127634 internal

RARRLVLALMLNKLSLSDATQLNCNEVCSELEVMLEMKQYNCLELCDNINQDSSNKRASFMRIGRASFMRIGRSGNEDQYGEGPDGEDTKRASFMRIGRPYMYSMKRPSFMRIGKRSLGETPEMKRASFMRIGKKSYDDGLYDDAKRASFMRIGKKASFMRIGKKDD

>FxRIa_Nemertea_Nipponemertes_spec_TRINITY_DN34748_c0_g5_i1_m.84056 5prime_partial

RIGKKDDDKRASFMRIGKKSDDEEEKRASFMRIGKKDDDKRASFMRIGKKSDDFEGEKRASFMRIGKSVADADDEKRASFMRIGRSV*

>FxRIa_Nemertea_Malacobdella_grossa_comp171429_c0_seq1_m.153726 3prime_partial

MSHNSPLRLSLLLALMITQQQQSNAKPLKCEEICKDLETRFGSKRSDCTRFCDSEESAKRASFMRIGRASFMRIGRSGLDDNDLQRDTRSYLYNFKRPSFMRIGRNSDKRASFMRIGKKNSDGEEKRASFMRIGKKDDSDMKRASFMRIGKKDRASVAEEIMDEDKRASFMR

>FxRIa_Nemertea_Cerebratulus_spec_Contig388_m.194711 5prime_partial

GFMRIGKKDDAEEDKRGFMRIGKKDAVKRASFMRIGKKSDDEMEEDEKRGFMRIGKKDNDKRGFMRIGKSSELINDDALASEEKRGFMRIGKKSDDDIIDKRASFMRIGKSLDSVEGDEAKRASFMRIGRSA*

>FxRIa_Nemertea_Cerebratulus_spec_TRINITY_DN38095_c0_g1_i1_m.109456 3prime_partial

MLVGYIPMRISLVLALMLNKLSVSESNELDCPAVCTRLSKIIDNYKCIDLCDSVNQEASQKRASFMRIGRSWPGDDVVDVNSNEGEEEDEKRGFMRIGRGFMRIGKRFPYGMKRPSFMRIGKRSLADEDEKRGFMRIGKSVESDKRGFMRIGRGDADELEQDKRGFMRIGKSMEDDKRGFMRIG

>FxRIa_Nemertea_Paranemertes_peregrina_comp22109_c0_seq1_m.20689 complete

MRMSYVPLRLSLVLALMITKQQQTEAKPLDCDDVCRELETMLGTKRYNCIRLCDSVNDNSKRASFMRIGRASFMRIGRSSDATSDDNDLQREARPYLYNIKRPSFMRIGKKSESDAEKRASFMRIGKKSFDDEEKRASFMRIGKKSEDAEKRASFMRIGKKSQAEDELENEKRASFMRIGRSSER*

>FxRIa_Nemertea_Baseodiscus_unicolor_TRINITY_DN104352_c0_g6_i1_m.62820 3prime_partial

MLVGYVPMRVSLVLALLLNKLSVSESNELDCPAVCLELAKYLKGNECNDLCDNVNRDASSKRAAFMRIGRAHFMRIGRSGQEPFDVSSVEDDDEKRNFMSDYDEKRSFMRIGKRFRY

>FxRIa_Nemertea_Baseodiscus_unicolor_TRINITY_DN104352_c0_g7_i1_m.62823 internal

IGKRFRYDDKRPAFMRIGKRSPDEDLEKKSFMRIGKRLDDKRGFMRIGRGDELYDDTSKRGFMRIGKKDAEKRNFMRIGRNNMDDKRGTFMRIGRGNFMHIGKKDTMTDEDKRTFMRIGKKAGFMRIGKKSDIPVPDNDVDVDSADEQEQDEESNFMRIGEKDDQLEENKRGFMRIGKKSSELANDEALSEALT

>FxRIa_Nemertea_Baseodiscus_unicolor_TRINITY_DN104352_c0_g7_i2_m.62826 internal

RDLHSFPTRRSSDLAFHRNRSPDEDLEKKSFMRIGKRLDDKRGFMRIGRGDELYDDTSKRGFMRIGKKDAEKRNFMRIGRNNMDDKRGTFMRIGRGNFMHIGKKDTMTDEDKRTFMRIGKKAGFMRIGKKSDIPVPDNDVDVDSADEQEQDEESNFMRIGEKDDQLEENKRGFMRIGKKSSELANDEALSEALT

>FxRIa_Nemertea_Tubulanus_polymorphus_comp37345_c0_seq1_m.62117 complete

MKLGPILYQWSLLLVLVIINKCAQGLDCREECTNTNVSVEERCVAVCENLLLQEQRKRASFMRIGRPDKNYMRTGHSKNEENAPVAGMSDGAYLYRDYQRYPRPAFMRIGRASFMRIGKRYSYKRPSFMRIGRRSMDEGGHFETDTGKSQIDDKRPSFMRIGKKNYADEKEEGKRANFMRIGRAGGTKSSFMRIGKRSDEEKELSPQLAPSVGDNEDKVSGSSEIGKNSYFNDAIKRASFMRIGKSTADRKRASFMRIG*

>Tubulanus_Polymorphus.25673 Nemertea

MKLGPILYQWSLLLVLVIINKCAQGLDCREECTNTNVSVEERCVAVCENLLLQEQRKRASFMRIGRPDKNYMRTGHSKNEENAPVAGMSDGAYLYRDYQRYPRPAFMRIGRASFMRIGKRYSYKRPSFMRIGRRSMDEGGHFETDTGKSQIDVKRPSFMRIGKKNYADKKEKSTDCDSALIPL

>FxRIa_Nemertea_Lineus_ruber_Lvir.rna.tri.37769.1_m.72365 complete

MLVGYVPMRISLVLALMLNKLSVSESNELDCPAVCTRLSKIIDNYKCIDLCDSVNQEASTKRASFMRIGRSWPGDGEDGMEVNSDEGDEDKRGFMRIGRGFMRIGKSFPYGMKRPSFMRIGKRSLDDMDDKRGFMRIGKSGDESDKRGFMRIGKSMGDDKRNFMRIGKSDSDKRASFMRIGKSSEEAKRGFMRIGKKDDDDKRGFMRIGKKSDKRASFMRIGKKSDDEAEKRGFMRIGKSDNEKRGFMRIGKSAELVNDEAMADSAEEKRNFMRIGKKNDDTGDTGASFLRNSLDSEDEAAKRASFMRIGRSV*

>FxRIa_Nemertea_Notospermus_geniculatus_g1179.t1

MLVGYVPMRISLVLALMLNKLSVSESNELDCPAVCSRLSKIIDNYKCIDLCDSVNQDASTKRGSFMRIGRGWPGAGEDVADVTSGEDDVDKRGFMRIGRGFMRIGKRYPYGIKRPSFMRIGKRSLDEEEKRGFMRIGKSVDDDKRGFMRIGRDGADFDEDKRGFMRIGKSFEDDKRGFMRIGKSDMNAEADKRGFMRIGKSAGDKKANFMRIGKSSEEAKRGFMRIGKKDIDEEEKRGFMRIGKKDDEKRANFMRIGKKSNEEEADKRGFMRIGKKASELVNDEAMADNASEEKRGFMRIGKKDDTMSHSIKENPTMTETQSQTT

>FxRIa_Nemertea_Lineus_lacteus_comp27522_c0_seq10_m.153575 5prime_partial

RFSVSATRANFRAQKEVNRTHGFT

MLVGYVPMRISLVLALMLNKLSVSESNELDCPAVCTRLSKIIDNYKCIDLCDSVNQEASIKRASFMRIGRSWPGDGQDVMDVNSNEGDEEKRGFMRIGRGFMRIGKSLPYGIKRPSFMRIGKRSLDDNDDKRGFMRIGKSADKRGFMRIGKSMEDDKRNFMRIGKSDSDKRASFMRIGKSSDEAKRGFMRIGKKDSEDDKRGFMRIGKKSDKRASFMRIGKKSDDGENHGFMRIGKSDNEKRGFMRIGKSAELVNDEAMADSAEEKRNFMRIGKKSDGSDDTAESFLGNNLDSEDEEAKRASFMRIGRSV*

>FxRIa_Nemertea_Lineus_longissimus_c45532_g1_i7_m.104867 complete

MLVGYVPMRISLVLALMLNKLSVSESNELDCPAVCTRLSKIIDNYKCIDLCDSVNQEASIKRASFMRIGRSWPGDGQDGMDVNSDEGDEEKRNFMRIGRNFMRIGKRFPYGMKRPSFMRIGKRSLDDNDDKRRFMRIGKSDESDKRNFMRIGKSMEDDKRNFMRIGKSMEDDKRNFMRIGKSMEDDKRNFMRIGKSMEDDKRNFMRIGKSDGVKRASFMRIGKSSEEAKRNFMRIGKKDDDDKRNFMRIGKKSDKRASFMRIGKKSDDEEEKRNFMRIGKSDNEKRNFMRIGKSAELVNDEAMADSVEEKRNFMRIGKKSDDTSNMGASFLGNNLDSEDGEAKRASFMRIGRSV*

## GnRH

>GnRH-2_like_Phoronida_Phoronis_australis_TRINITY_DN280848_c0_g1_i1.p1

MVRCLEVLLITVSLTLLLQQCSAQWHHSYGWGSAGGVGKRTSAESETKLCDRLQEQLTTAINFVAEINRRKQMLCENVDVFNKDKR*

>GnRH-2_like_Phoronida_Phoronis_ijimai_(P._vancouverensis)_comp461515_c0_seq1.p1

MVKCLGLLLSTACLALIIQQCVGQWHHSYGWGSAGGVGKRASADSETKLCDRLQDQLTKAITFVAEINRRKQVLCESVDVFNKDQR*

>GnRH-2_like_Phoronida_Phoronis_psammophila_comp275993_c0_seq1.p1

MNNAQICFLLAACVAALTLSRPVTGQWHQTYGWGSAGGAGKRAGQTNGIQAKLCETLHDDYADALQKLYEIRSRKRLFCDNVDVFNRKR*

>GnRH-2_like_Brachiopoda_Lingula_anatina_comp157492_c0_seq1.p1

MVTKHNMLWISVTVIVMVLQNTNCQWHQTLGWGAAGMVGKRTNPSSIFSQDSQDDSLCDDRRNSEVMKLIRDLLQAEIMRTDYCYNRKFEENRVRGFTKQDAPRV

>GnRH-1_like_Brachiopoda_Hemithris_psittacea_comp454615_c0_seq1.p1

MKNWGVILLVCVVVAYQLDMSEAQFSFSLPGRWGSGKRAAPWMRRGTGACEDIDTEVV

>GnRH-2_like_Brachiopoda_Hemithris_psittacea_comp554389_c0_seq1.p1

MNLHKLVAQTRLKMSHLQSQLVCVKRRGIEPNALLRTLLILTLLLTVAEGQWQQSLGWGSAGMAGKRALLSSKAAARNQQKMYQLRCTMAKDELVKSVYRFIENEAKKVDYCRLDHSHLFASLSGSLA

>GnRH-2_like_Brachiopoda_Terebratalia_transversa_Ttra.rna.tri.13129.1.p1

MNKILRFTTGQKNTREVNYCLKIVFVVLLIIAVANGQWQQTLGWGSAGMTGKRATNALQIRLYKLRCNLLQDEIDATRDKLHKLTSQAVQCTQPQGIKQITESEEVKGSRKTTNANLKNMTARIRQT

>GnRH-1_like_Brachiopoda_Terebratalia_transversa_Ttra.rna.tri.16355.1.p1

MRIIFTIDIFILINLVYALCSVHGQISFSPTWGKRSRLRETVKMIVDTPKFQEYSSRRFFFTRCSKGKLIALLRLISNSIKANSDEDVYSIESHWNK*

>GnRH-1_like_Nemertea_Paranemertes_peregrina_comp521582_c0_seq1.p1

MKNWGVILLVCVVVAYQLDMSEAQFSFSLPGRWGSGKRAAPWMRRGTGACEDIDTEVVYGIYKAIESD

>GnRH-2_like_Nemertea_Riseriellus_occultus_TRINITY_DN8240_c0_g1_i1.p2

...CFVLVNLAHPIQGQWSQSLGWGGAGAGKRSQGVTNKCQEHSLALREMVSKLIELEAMRLIRCQGESASQDKAR*

## HFAamide

>HFAamide_Phoronida_Phoronis_psammophila_comp386714_c0_seq1.p1

MSYTHRTTMRVSTRAIVISNYCVIIGVICLFVRCEAGKRQMEKSQFCRIICSRGGRGRGSALCMCQGRHFTGKRGTDEQLNRLDTRTLFYTDSYADVASNIPVTRVSNVFDKSDVKPRRTSSSYSKVLLTSMTTLLKEELSLKRMKSRWQSATGKVYR

>HFAamide_Phoronida_Phoronis_ijimai_(P._vancouverensis)_comp60525_c0_seq1.p1

MRLYTAYVISNYCLIIGVVCFFVHCEAGWSKSQFCKIRCRLGHRQAICACHGKHFTGKRAALEHSLYTRHRATESAASLASKPTNIPKFSESSQLFSRKTHPVRTDRPYTYLLSTLLLQKSILEQLRYRRQSKTKDNRYDQIKRLTNKNLFMD

>HFAamide_Phoronida_Phoronis_australis_TRINITY_DN291066_c0_g4_i1.p1

MRLYTAYVISNYCLIIGVICFFVHCEAGSWYKSRQLFCRMICKSGHPQAICACRARHFTGKRAAAQEHSLYTPYGASESGASLANKLTNIPKLSESSELVIKKTLPIRTDRPYTYLLSTILLQKGISEKLRYVRLSKPNDNRRDQVNSLFFDVRLSINQTTLRMIVKIQEEVVMLQFRLTKKNLFTD

>HFAamide_Phoronida_Phoronis_australis_TRINITY_DN291066_c0_g4_i6.p1

MRLYTAYVISNYCLIIGVICFFVHCEAGSWYKSRQLFCRMICKSGHPQAICACRARHFTGKRAAAQEHSLYTPYGASESGASLANKLTNIPKLSESSELVIKKTLPIRTDRPYTYLLSTILLQKGISEKLRYVRLSKPNDNRRDQVNRCIFPSSLSRRLASRVRYLCIFKGSHRIFMKFGTCASVSEMTLSSHLRMGLKYFTPSSDHLTIFTLFESIL

>HFAamide_Brachiopoda_Novocrania_anomala_Nano.rna.tri2.242620.1.p3

MNILTTVLLVLFSLCVGLNTCVGGFKLSVYCSNKCPLGQGGNVCRCNAVHFTGKRNIQ

>HFAamide_Nemertea_Cerebratulus_spec_TRINITY_DN23888_c0_g1_i1_m.46388

MGRLYLLLILTVGIFTSTDGFDQERYCRIMCRKGLGGNACGCNAVHFAGKRTSESSLYHKPPSLSQILQPKTPQKDPGSDRELAYLVHVIATLQKNKGTDSADVHGNRLQGARIHDTLTDIRRKQENRLQDQEKLKRLERYLFADWRYRPHRKSQDSDRKNGGVGNVIEPNDPGE

>HFAamide_Nemertea_Tubulanus_polymorphus_comp39208_c0_seq1_m.86770

MRPSTGSSAVSVSFCVLVLMFVLPDCSLAGRFSLDRFCSQMCPRGRGGTLCRCNHVYFAGKRASGLTDGLPDEHTLLGNIIKTDEVPTTLRTINTDKSVENWDPPTEVSLASQTDRYSNRLRHFLAGVENSNKGTINRLKQLLVEELNMKNIRKPNKKETKRRRRRRDLDWRLQNMPVKTTR

>HFAamide_Nemertea_Baseodiscus_unicolor_TRINITY_DN101039_c1_g1_i2_m.70769

ILFASIDFRQCFNKAEFCRLACNRGRGGILCGCNARHFTGKRDSKSLFNPDNHLEPSTKDRNGPREGPTDS

>HFAamide_Nemertea_Cerebratulus_marginatus_comp28381_c0_seq1_m.14813

MARLYVFLILAVGIFTSTECFDREQYCTIMCRRGRGGNACMCNAVHFSGKRTSDMSLYQPSLSQILQPKTSNDHLDDQELEYLVHVLATLRNNKGSAPAEIPRDKLPDTRIHDTLTDIRRKQADRLKDQEKLKRLERYLFADWSYRPVRKTQDS

>HFAamide_Nemertea_Lineus_lacteus_comp34371_c0_seq1_m.255213

MGMGRLHLLLIITIGIFTSLERGECLNKVRFCRIMCARGQGGDACRCNAVHFAGKRTSETSINPPQRLSNLLSLNNDKSRHKDLAYLVHVIASLHHEDKLLDSAETTRSTRQGTRIHGSRKQMRTKKENELTDLENIKKIERLIHELEQTEEGTDS

>HFAamide_Nemertea_Lineus_longissimus_c41200_g2_i5_m.64584

MGMGRLHFLLIITIGIFTSLDCGECLNKVRFCRIMCARGQGGDACRCNAVHFAGKRTSETSVHPPRRLSNLLSVNNDKSRQRELAYLVHVIANLHDDDKLLDSAETTRSTKSGTRTHGSRNHINTKKEDQLTDLEKIKKIERFITELEQTEEGANS

>HFAamide_Nemertea_Lineus_ruber_Lvir.rna.tri.53076.1_m.95446

MGMGRLHFLLIITIGILTSLDRGECLNKVRFCRIMCTRGQGGDACRCNAVHFAGKRTSELNVYPSQRLSQVLSVSNDKSRQKDLSYLVNVIANLHNEDSLLDSTATTGSTEPGTRIHGNRNHIHTTKENQLTDLEKIKQIKRFIHVLEQTEEEARS

## HIGA

>HIGA_Nemertea_Cerebratulus_marginatus_comp1840243_c0_seq1_m.158092 internal

FTDEDYIDDFEKRHLGSAMNSRFGKRYDDEDGEDIEKRHLGALMRSSWRSRHRMEEDPNADKRHLGSLMRSRFRLYPKRHLGSAMGSRFRIYDGQAAKRHLGSAMGSRFNLYQNQKRDDMDFDEE

>HIGA_Nemertea_Riseriellus_occultus_TRINITY_DN67540_c0_g1_i1_m.121639 internal

KRHLGSAMRGRVGKMKDKRHLGSLMKGKFGKKDDEFEKRHLGSLRKSQFGKKDHDELDKRHLGSLMKGKFGKRDDEEEVDKRHLGSAMKSKLGKNDEIDKRHLGSAMKTKF

>HIGA_Nemertea_Riseriellus_occultus_Contig4370_m.129696 complete

MNVRFGKRFDFDDDDQDVDKRHLGALMRSRLHHREDDDDVDKRHLGSAMGSRLKMYDKRHLGSAMNSRYRLYKDRADAMRNKKRDWDNTDIDDAEAKRFLGAAIRYRQRQQEADKDKRHLGSAMKFGKRHLGSALNYLRDSPYRVANKEQQKRSTEVSEGKRDMTHENDIKIREES*

>HIGA_Nemertea_Baseodiscus_unicolor_Contig2038.p1

MLCPCVSSPWHHVTPKPAIRYYPFLPSSDERLHYSEPKYLRDRRYLGSLLRSGIRNRKLHYPWWRDPNRSMENGEMYKYPSLYGIPDVPRDQVKREFEDYETEAWGKGHLPAWLEEGIAPGAQTRRRRDLAKGPDEVDDAETVKRHLGSIMRSRFSKNLQTNEDVGKRHIGSIMRSGFRKYRLSSKN

>HIGA_Nemertea_Baseodiscus_unicolor_TRINITY_DN89253_c2_g1_i1_m.190699 internal

QEELDSKENGVQKKHLGSALRMRQGKRPDGPDDDDSGVVVDKKHLGSALRAGRKRQDDDFGDEIDEGASGNDFDKRHLGAALNARFGRDGHKYDIFTTGDKKNLGSAMRWKGGKRDDDLDFEADKGTNKRHLGSALNSRFGKRQDNDNMNDNKRHLAAALSAQYENRDDKRHLGSAMRAMRSRLKSYPAPKRHLGSALNSRFGKRNDES

>HIGA_Nemertea_Tubulanus_polymorphus_comp33153_c0_seq1_m.33563 3prime_partial

MATLCTFLAVICVVLMGLEDIRVKAVTKRHIASLARLNWIPKPSMRYYPFIEEEVHPRAAYNDRRYERGDGLRNSLVRTTIARPWWKKPQQSYDNSYVNYEAPYVNVESVRGHRKRDNDGDYETEEWGQTDLPLQLQGPLAYTWPNNRHSSAISGYEMNKRHLGAAYNWGKRNIGAARNWGSRYRGKRHVDGKASDDVLTNHIKQTQGQTWEGINIADKPQEPEGDKGASIHVVDDAMIRSGEIDKRLISSVARYRSGNRKYDKDDYLPNNSDDTDNSGWQLSKTDKPYIGSVRYWLQDKIGDSKSDEQEEMDIKRHMGSAMKWKGATKRHIGSARAWRTGKR

>HIGA_Nemertea_Cerebratulus_spec_TRINITY_DN30207_c0_g3_i1_m.172238 internal

DKRHLGSIMRGKKDDDEMDKRHLGSIMRGKKDDDEMDKRHLGSIMRGKKDDTEMDKRHLGSIMRGKKDDDEMDKRHL

>HIGA_Nemertea_Cerebratulus_spec_Contig6558_m.215998 3prime_partial

MRGKKDDAEMDKRHLGSIMRGKKDDAEMDKRHLGSIMRGKKDDAEMDKRHLGSIMRGKKDDDEMDKRHLGSIMRGKKDDAEMDKRHLGSIMRGKKDDDEMDKR

>HIGA_Nemertea_Cerebratulus_spec_TRINITY_DN30124_c0_g1_i2_m.43587 3prime_partial

MKTFCFLVLICATICFGEENRAHKIQKRHLGSLARHNWLPKPAIRYYPFLPSDDDRLPYAEPLYMRDRRYLGSLLRSGMGTRRENYPWWKDPDRSLENTETYRYPGIFNVAETPRDQRKREFEDYETEAWGAGHMPDWLDMGVEEGPARRRRETDLDWDIADEDAEFDKRHLGSIMRGKKDDDDEEMEKRHLGSLRKWKVGKKDYDDDAEFDKRHLGSIMRGKKDDSEMDKRHLGSIMRGKKDYDMDKRHLVSIMRGKKAANKRHLGSIMRGKKDDDDE

>HIGA_Nemertea_Cerebratulus_spec_TRINITY_DN34380_c0_g1_i1_m.187755 5prime_partial

DDDEMEKRHLGSIMRGKKDDEEMEKRHLGSLRKWKVGKKDDAEMDKRHLGSLMKGRYGKRHLGAAMKGKFGKRHLGSLRKFQLGKKDSDDVDKRHLGSLMRGRYGKKDEEDQFDKRHLGSLARGRTRSKRSTDDLDLEEYKRHLGKARNWRFGKRDDFSDDGEWEDFEKRHLGSAMNSGFGKRFDGDEEVDKRHLGALMRSSWRNRYQTENDPETDKRHLGSLMRSRFTLYPKRHLGAALSTRYRTYGNQAEKRHLGAALGSRFRLYKNQKREDMDTDFGEEKRFLGSAMRYRQRPDEDVDKRHLGSAMKFGKRHLGSALNYLNSARYRLKNTKRSVDADDVETDVTSQINDENES*

>HIGA_Nemertea_Lineus_longissimus_c34720_g1_i1_m.31839 3prime_partial

MNMRVLCLFVLICVTLNRAEKAKEHRIKKRHLGSLARHNWLPKPAIRYYPFLPSDDDRLPYAEPLYMRDRRYLGSLLRSGFGTRKENYPWWKNPDGSVENTEMYKYPGVFGVGEVPRDQRKREYDDYETEVWGEGHLPDWLDMGVDDGPARRRRETEESDSDDLDDEVIKRHLGSAMKSRLGKKDDKRHLGSLMRGKFGKRNDESEFDKRHL

>HIGA_Nemertea_Lineus_longissimus_c46447_g1_i1_m.115613 5prime_partial

HLGSAMKSRPSKKDDDEFDKRHLGSAMRLGKKDNEDSGRNSESKRHLGSLMKGKFGKKDGEFEKRHLGSLRKSQFRKMDQNELDKRHLGSLMKGKFGKKDDEGEVGKRHLGSAMKSRLGKKDEIDKRHLGSAMKAKFGKKDDAEVDKRHLGSIARGRTRSRRSADVLGREESKRHLGRARNWRIGKRDDESDEIEFEKRHLGSAMNVRFGKRFNDDEDVVDKRHLGALMRSRLRHREDDDDVDKRHLGSAMGSRFRMYDKRHLGSVMTSRYRLYQNRVNGMRNKKRDWEDAEVDDAETKRFLGAAIRYRQRQREADKDKRHLGSAMKFGKRHLGSALNYLRDSRYNVANKENQKRSTEQES*

>HIGA_Nemertea_Lineus_ruber_Lvir.rna.tri.51518.1_m.93278 3prime_partial

MRVFCLIVLICVTLSKAEEAKEHNIRKRHLGSLARHNWLPKPAIRYYPFLPSDDDRLPYAEPLYMRDRRYLGSLLRSGIGRKENYPWWKNPDGSLENTELYKYPGLFGVGEVPRDQRKREYEDYETEVWGEGHMPDWLDMGVDDGPARRRRETDNSDSDDFDEEVEKRHLGSAMKSRLGKKNGDTDFDKRHLGSLMRGKFGKKDDKSEFDKRHLGSAMRLGKKDDEEFEKRHL

>HIGA_Nemertea_Lineus_ruber_Lvir.rna.tri.41549.1_m.78228 5prime_partial

RHLGSAMRGRFGKMEDKRHLGSLRKFGRNSESKRHLGSLMKGKFGKKDDEFEKRHLGSLRKSQFGKKNHDELDKRHLGSLMKGKFGKRADEGDDVDKRHLGSAMKSRLGKKDEIDKRHMGSAMKAKFGKKDDTDVDKRHLGSIARGRTRRSADDLEEAKRHLGRARNWRVGKRDDESDEVDFEKRHLGSAMNVRFGKRYDVDDDEDVVDKRHLGALMRSRLRHRENDDEVDKRHLGSAMGSRFRMYDKRHLGSAMNSRYRLYQNRVNAMRNEKRDWEDTEVENDEVKRFLGAAMRYRQRQNEADKDKRHLGSAMKFGKRHLGSAMNYLRNSRYNVANRESQKRSTEVSEGKMDMTHGNDIKTEQKS*

>HIGA_Nemertea_Lineus_lacteus_comp26283_c0_seq1.p1

MRVFCLFVLICVTLNQAEEAKEHSIKKRHLGSLARHNWLPKPAIRYYPFLPSDDDRLPYAEPLYMRNRRYLGSLLRSGFGSRKGNYPWWKNPERSVENTEMYKYPGLFGVGEVPRDQRKREYEDYETEGWGEGHLPDWLDMGVDDGPARRRRETDDSADLDEEVIKRHLGSAMKSRLGQKNDKRHLGSLMRGKFGKRDGESEFDKRHLGSAMKSRLGKKDDDEFEKRH

>HIGA_Nemertea_Lineus_lacteus_comp26283_c0_seq3_m.106842 5prime_partial

GSAMRLGKKDDDEEFEKRHLGSAMRLGKKDDDEFEKRHLGSAMRLGKKDDDDVDGNSERKRHLGSLMKGKFGKKDGKFEKRHLGSLRKSQYGKKDQDELDKRHLGSLMKGKFGKRDDEGKVDKRNLGSALKSRLGKKDEIDKRHLGSAMKAKFGKKDDAEVDKRHLGSIARGRTRSRRSADVLGLEESKRHLGRARNWRVGKRDDENDEDEFEKRHLGPAMNVRFGKRYDDDDEDVLDKRHLGALMRSRMRHREDDDEADKRHLGSVMGSRLRGKYDKRHLGSAMTSRYRLYQDRMKAMRNEKRDWGDPEVDDAETKRFLGSAMRYRQRQREDDKDKRHLGSAMKFGKRHLGSALNYLKDSRYNVANKAHQKRSTAAAEGKTGMTHEDDIKTEPES*

>HIGA_Nemertea_Notospermus_geniculatus_g12448.t1

LYLDSRGRRVVLDQTVQPQLLTEQITKRHIGSLARNNWLPKPEIRYYPFLPSNDRRLPNVKPVYMRDRRYLGSLLRAGVGTRKGNYPWWKDPDRSLENMENTEMYKYPGFYGVGEIPRDQRKREFGDYDTEAWGEGHLPDWLDMAADEGPARRRRETEYPDSSDEEQIGDIDKRHLGSAMSFGKRDVKSFGKRDDERDVDKRHLGSVMKSFGKRDDEGDVDKRHLGSVKSFGKRDDEGEVDKRHLGSVKSFGKRDDEGDVDKRHLGSLMKTKFGKREGMENEDKRHLGSLMKSKFGKKNAEIDKRHLGSLRKFGKKDTADKRHLGSVSRGRTRSKRSTELEEHKRHLGSARNWRWGKRGDEYPDVDGDYEKRHLGSVLNSRFDKRHLGALMRSMYGRRPVDDEGDDDKRHLGSAIRSQYMLYRSRPRSGKRDSEEEETEFEKRHLGSALNYLKSRRYDVQGHREKRSVETNMEHIKS

## L11/elevenin

>L11_Phoronida_Phoronis_australis_TRINITY_DN286489_c0_g3_i3.p1

MQKNLYWLTAALVIWSALILTSDAMWYRFRRPRWRKIDCRKYPFAPRCRGVWTVKRSSPAVDNVNEYVDYPSILKEDYPLIKRLRSYNARR

>L11_Phoronida_Phoronis_ijimai_(P._vancouverensis)_comp34408_c0_seq1.p2

MQKNLYCLMAVLVLWSALILTSDALWYKLKRPRWRKIDCRKYPFAPRCRGVWTVKRSSPVVDNVNENVDYPRIHKEEYSLLKRIRRYNVQR

>L11_Phoronida_Phoronis_psammophila_comp71038_c0_seq24.p1

MRFKCGLEDIMHKAFYFLVILLVVWSEVILSSDGAAFRLKKGRRNGVRRRLNCWKFPFAPKCRGAWAKRSYPSYEDLQRQKALEEASEQENAYDVFNRIQESLSDKTPYGQLSSTYRGGDVTTVIPVESHDNDDIVTLEKRKKY

>L11_Phoronida_Phoronis_psammophila_comp71038_c0_seq1.p1

MRFKCGLEDIMHKAFYFLVILLVVWSELILSSDGAAFRLKKGRRNGVRRRLNCWKFPFAPKCRGAWAKRSYPSYEDLQRQKALEEASEQENANDVFNRIQESLSDKTPYGQLSSTYRGGDVTTVIPVESHDNDDIVTLEKERLLRIILSKLRRVVSQRNSRGKEKRSERVADDRKWTLEFGTGS

>L11_Brachiopoda_Glottidia_pyramidata_comp31910_c0_seq2.p1

MHRIANIVLTTFFLVLLLSKGTDSVDCRKYIYHPSCRGIWAKREYRISDDSRLEKSRGTYANIYRQLGNRYSRWDMGEDDGNIPLETDPDEKIMTRSPRANNRRQFDIYYLPIYKVLMRRR

>L11_Brachiopoda_Lingula_anatina_comp141236_c0_seq1.p1

MDQQPCMVENWHPGMHRIANLILTTLFLVLLLSKGTGSVDCRKIIFHPSCRGIYAKRDYSISDNSHLENTLGSYANLYRQLGRRLPVWKTGDEEEEGERGMEMELETDPADDMMDNRPHAKDRRQVDVYYLPIYKVIMRRR

>L11_Brachiopoda_Lingula_anatina_comp141236_c0_seq9.p1

MSYIYSLLEYLKSTENCMHRIANLILTTLFLVLLLSKGTGSVDCRKIIFHPSCRGIYAKRDYSISDNSHLENTLGSYANLYRQLGRRLPVWKTGDEEEEGERGMEMELETDPADDMMDNRPHAKDRRQVDVYYLPIYKVIMRRR

>L11_Brachiopoda_Lingula_anatina_comp141236_c0_seq5.p1

MLLAFGKYGLYFIPSKGMHRIANLILTTLFLVLLLSKGTGSVDCRKIIFHPSCRGIYAKRDYSISDNSHLENTLGSYANLYRQLGRRLPVWKTGDEEEEGERGMEMELETDPADDMMDNRPHAKDRRQVDVYYLPIYKVIMRRR*

>L11_Brachiopoda_Laqueus_californicus_comp404667_c0_seq1.p1

MMVCVGILTINKVECRRPRRIDCRKYLYAPRCRGFAVKRNLDNMVSSSRLHSLLTHIVHQDKQIYDDNALFSGHSSSSYSKRSDTDSHKYKDNYEIKL

>L11_Brachiopoda_Terebratalia_transversa_Ttra.rna.tri.29920.1.p1

DCRKYLYAPRCRGFAVKRNLLDKQIKAITSIGSLLNHVAQVKNIDDDVVLLSEHINGDPKHTDPTHLDNYRIKQIPDNNFNIQVFALLIQRMKSQETRSWTRRKMENFDA

>L11_Brachiopoda_Novocrania_anomala_Nano.rna.tri2.51907.1.p1

MQAITRILALGYIILVFSKDNRVASIDCTKFVFAPACRGIVTKRVLDPEPVARLNDQVGLTLRDARTLYTILNHWSRYHGDQSKPSQARSAYYGPGSIYTGIKDTTMEKREPTKDNKLNLYDFLRNMR

>L11_Nemertea_Cephalothrix_hongkongiensis_comp102983_c0_seq1_m.97297

MKGIDLHTLVSTFLTILIFSTLCTSVTGRRQVDCRVYVFAPRCRGVSAKRSGDGLKLNTLSDDTEFTTRNTLLDKRLQKIWALKKIREFLNKPQDIQQIIRLSNEDTSE

>L11_Nemertea_Lineus_longissimus_c43644_g1_i1_m.85108

MKYVHQIAISIAFLLLLLIDTTCYARHIDCRKYVFAPKCRGVSAKRSSPSEQMITDSTKDETNYLLDDGSLDLGALEQLQQGSDISGKEESWFGNEEDDADRTNRLRLSKLDEIRQLLRKLQ

>L11_Nemertea_Lineus_ruber_Lvir.rna.tri.50896.1_m.92435

MKYIHQISISIAFLLLLLIDTTCHARHIDCRKYVFAPKCRGVSAKRSGPNEQMMTDSTKDETNYLLDDGSLDLGALGQLQPGSDISGKEDSWFGNEDDDVDRTNRLRLSKLDEIRHLLRKLQ

>L11_Nemertea_Cerebratulus_spec_TRINITY_DN31209_c0_g1_i1_m.103596

MKYVHQISISIAFLLLLLIDTSCHARHIDCRKYVFAPKCRGVSAKRSGPMSNEQMVPETAKETNYLLDDGNLDLSTLEQLQQASDISGKEDSWFGSEDDDADQTNRLRLSKLEEIRQLLRKLQ

>L11_Nemertea_Tubulanus_polymorphus_comp28696_c0_seq1_m.20044

MKLPHLLHPAVICFVILLHVYATSSRILNCRKYVFAPRCRGVSAKRSSQARNLDDSLEVLKSLANTLPKSVDDVGYSDARDAADVASSTETSSWLDESDSKSVGQRPERIRAILRELIRDLQNPK

>L11_Nemertea_Baseodiscus_unicolor_Contig28683_m.263806

MKPLYKVSFSLAFFILLLIDTPCLARRIDCRKYVYAPRCRGVSAKRSNQMSNQLLTATSAEGKTNLLIDEMDPKLAFEALERISYPETDITPKEEAAWLDDEMTDSDRTEQPSNELSKLAVLKRLLEKLQ

>L11_Nemertea_Malacobdella_grossa_comp1256_c0_seq1_m.608

MRRVQLVTISTVAVLVLFMTSSVNCVDCRKYVFAPACRGVWAKRSGMDPTQDYPRDVLSGVETNNIKLPNPYMKDQILEEYEDRMENKQRNRSHAAIRLLVQLQKLHRRRTSPLATLLQRLNKDQAETGDF

>L11_Nemertea_Lineus_lacteus_comp8962_c0_seq1.p1

MKYVHQLAISIAFLLLLLIDTTCHARHIDCRKYVFAPKCRGVSAKRSGPNEQMITDSAKDETNYLLDNGNLDLGTLEQLQQGSDISGKEESWYGNEDDDADRTNRLRLSKLDEIRQLLRKLQ*

>L11_Nemertea_Nipponemertes_spec_Contig4896_m.113647

MRARVQMVILTTFVIVVLLTTSTVDCVDCRKYVFAPACRGVWAKRSSNSVPDIDFIKETGTEDGDDSNALRTWLLNVLKQKLAAPEPAPSHSQLSLLNKLKRLQHLSLAGLVPDTETQDSLQQEDAPSSRGEVFY

>L11_Nemertea_Paranemertes_peregrina_comp17250_c0_seq1.p1

MRRVQFVTISTVAVVVLFMTSSVDCVDCRKYVFAPACRGVWAKRSSADALQNNDYGLKSSEPIGDLSSEAGTGDTDEMLEKYEGRMKQKQSQRLHAIRLITQLQKLHRQRNLPLSTLLQRLNKDQSRASVF*

>L11_Nemertea_Notospermus_geniculatus_g11675.t1

XXXXLLIDTTCYARHIDCRKYVFAPRCRGVSAKRSGPVTNEEILPESTKDETNYLLDDNLGDLATLAQLQQGADISGKDSAWFGDEVDTGDQTNQLRISKLEEIRQLLRKLQQ

## Leucokinin

>Leucokinin_Nemertea_Cephalothrix_hongkongiensis_comp33090_c1_seq1_m.30484 5prime_partial

KPWGGKRLSDDNKRGFKPWGGKRYSSDLNENNFINNESSNKRGFKPWGGKRSNQDDAKRGFKPWGGR*

>Leucokinin_Nemertea_Cephalothrix_hongkongiensis_comp33090_c0_seq2_m.30481 internal

FYYPLRLIRSAKSEKRPSFKPWGGKRNNLNEEDKRGFKPWGGKRISGEDDKRGFKPWGGKRVSEISDKREFKPWGGKRNSLDADKRAFKPWGGKRNSDNGDDKRAGFKPWGGKR

>Leucokinin_Nemertea_Baseodiscus_unicolor_Contig31798_m.266878 5prime_partial

HLIVLQFFQCLFLFAFSVPWGGKRFVRSAEEVEVKRGFRPWGGKKSTPSEKLLAEFEAAARYGPKRGFRPWGGKRERGPDADDDKRAFRPWGGKREDETDDKRGFRPWGGKRGFRPWGGKREGTA*

>Leucokinin_Nemertea_Lineus_longissimus_c33990_g1_i1_m.29492 5prime_partial

FRPWGGKKSEHDASSEKLIAQLKATGLGPKRGFRPWGGKREFRPWGGKRDSLKREFRPWGGKRAENDEEKRGFRPWGGKRSDQVDNV*

>Leucokinin_Nemertea_Lineus_longissimus_c22286_g1_i1_m.12407 3prime_partial

MTSHTLQPVLLLFCFASVISALQIQKDKGLDDSNLDKLIDSQGLERRGFRPWGGKRDDEQYQESLPEFLSGVDQEIAMTARGFRPWGGKRSVRSVEESHMAGDESKRGFRPWGGKRAGEDEDKRGFRPWGGKR

>Leucokinin_Nemertea_Notospermus_geniculatus_g20010.t1

MTRQAWQPIFVVCCFLVSAISSLPFGKDDEAVFDSDLANSQTLERRGFRPWGGKRDDEHFQDTLPDILSGSDQDIEMAVRGFRPWGGKRSARSTEGVADDTKRGFRPWGGKKSDQTEEAGRIFAQLEASGNFGPKRGFRPWGGKRFDVDSKRGFRPWGGKREPLKREFRPWGGKRDEEAEKRGFRPWGGKREADLVKKAKFRPWAGKRSQKA

>Leucokinin_Nemertea_Cerebratulus_spec_TRINITY_DN35838_c6_g1_i1.p1

MNRLYLQPVLVILCVAVISALPFKDDSSLDKLLDTSGLERRGFRPWGGKRDNEQYQESLPDFLSGADREILMSARGFRPWGGKRSVRSAEEKEEDEKRGFRPWGGKKSDHGAAAEKLIAQLEARNVGPKRGFRPWGGKRSDLDTKREFRPWGGKREFRPWGGKRSDDEVDKRKFRPWGGKRSDDVVEKKAGFRPWAGKRSASV*

>Leucokinin_Nemertea_Lineus_ruber_Lvir.rna.tri.54991.1.p1

MTLQTRQSALILVCFVTVISALPLQDDKELVDSNLEKLINSHDLERRGFRPWGGKRDDEQYQDSLPEFLSGVDQEHEMSARGFRPWGGKRSVRSADETHTEGDESKRGFRPWGGKRAEGDEDKRGFRPWGGKKSDQDVATEKLMAQLEATGLGPKRGFRPWGGKREFRPWGGKRDSLKREFRPWGGKRAESDEEKRGFRPWGGKKSDEVEKKKFRPWGGKRSIHI*

>Leucokinin_Nemertea_Tubulanus_polymorphus_comp35540_c0_seq1_m.47311 complete

MTSKRIHYFFVGLLAFFVGHKDGDASNPSDKRAFKPWGGKRSNNLREFDEVLNGLISRAKTDDLDDGRIVFGDVERRAFKPWGGKRAFKPWGGKRAEESDEENVGFKPWGGRRELDSSEKRAFKPWGGKRAFKPWGGKRDVEYELPLALNIRAVRSTDNISQKRSFKPWGGKRNDDRDDEKKRGFKPWGGKRDMFSQKRSFKPWGGKRDTDDGNDSLKRSFKPWGGKRDDSPMERRSFKPWGGR*

>Leucokinin_Nemertea_Lineus_lacteus_comp5080_c0_seq1.p1

MTSQTRQPVLLLFCFASVISAFPIEEGKGLDDSNIDKLIDSQGLERRGFRPWGGKRDDEQYQQGLPEFLSGVDQEIAMTARGFRPWGGKRSVRSAEESHSVGDESKRGFRPWGGKRTEDDEDKRGFRPWGGKRAEGDEVKRGFRPWGGKKSDHKPESEKLIAQLEATGLGPKRGFRPWGGKREFRPWGGKRDSLKREFRPWGGKRSEDDEEKRGFRPWGGKRSDQVDKKKFRPWGGKRSTHV*

## LKFL (Phoronid only)

>LKFL_Phoronida_Phoronopsis_harmeri_c71602_g1_i2.p1

YDDIDSEKRLKFLKRGNDDIDSEKRLKFLKRKYDDIDSEKRLKFLKRQNNDINSEKRLKFLKRDNDDINSEKRLKFLKKSTGTATHTIESDKRLKFLKRNLGEDDIESEKRLKFLKRMSG*

>LKFL_Phoronida_Phoronis_psammophila_comp320842_c0_seq1.p1

LNVMRRWLNVGARYRSLDGAAPEYRRWVNKGAYIPSDEDGTILGRRWLNSGARFRSASTDMDDEDNGFQNVDEGIFDDELVRKRRRRDVSEGSLTLKDGEETSGADSGYEESREISKRRYFIGRHLPGKSNDSPKEVTKRRYFIGRHLPQVKGKQSQGSDMDASKRLKFLGKRGKVDQKTLDVIKQILNGDYVEAEKRLKFLGKRVASGWEDEAESSPDKRLKFLKRPSGLDDELVSEKRLKFLGKRNEDTRTVTRDDSDDIASEKRLKFLKRFGDSEDIETEKRLKFLKKNFGLDSSEMPTEKRLKFLKRYEDLPDKRLKFLKKDQNPLSEDIESEKRLKFLG*

>LKFL_Phoronida_Phoronis_australis_TRINITY_DN310957_c0_g1_i2.p1

MSRIIPVQLVLCVLLMLHAEVFGVLMLQSHPKRVKFLGMTGYPPGKRDATPGDALYRYDSRFGNDDTEDAKEHTRYRSQEAYEWPSESAFNQAAGSLQDNESELRRWINPGAKFGRNVPVAGWDERRWLNTGAKYRELDDKPYRRWLNPGARIPVPDENRDVGRRWLNAGARYRSANDFNNDVENTWDDDATLLSRRRRDVLDISKNGADALMKNDKDDDKPSRAVRHASTQQQLQDSNRDRRINAALMYGSKINNVSNLWPKKSGKRHKYLKNMETPDKRLKFLKRNYVNDDHPNDEKRLKFLGKRKDEYPMTVPDLAFTETEAEKRLKFLKRQSDDTDIESQKRLKFLKRFSDDDIIESDKRLKFLKKNDSDELPDKRLKFLKRNLLKGAIASEKRLKFLG*

>LKFL_Phoronida_Phoronis_ijimai_(P._vancouverensis)_comp89324_c0_seq1.p1

MSWVIPTQVVLCGVLMFYAEVFGMLILQSQPKRVKFMGLDNFPPGKRDATPGHALYLYDSGLGNDDTEDANRYVLSRSREAYERPSESALNYAAMGLGDGESELRRWINPGAKFGRNIPGGGWDRRRRWLNAGAKYREAEDKPYRRWLNPGARIPEQDDDRDVGRQWLNAGGQYRSVDDFDNVAEDFWDDDVMLLNRRRRDVSDVSKGGIESLKGNDNADDKPSRAVRHASIEQQLQDSDKDRRKYMNKRRNFIGRENAVSIRHLRKGLLKRIVNGEARDVVRRLKFLGKTAGNSPSDDEHVHDVIQNSIDASKRLKFLGKRQWPVDDTKTDKRLKFLKRHYVNDDQPVDEKRLKFLGKRQDEYPRTFPDLAFTEAEAEKRLKFLKRQSDDNDIESQKRLKFLKRFNDDDVIKSDKRLKFLKKSASNELPSEKRLKFLKREQDDLPDKRLKFLKKSLPHGDIESEKRLKFLG*

##Luqin

>luqin_Brachiopoda_Hemithris_psittacea_comp9547_c0_seq1.p1

MASRRHAHRSNLREVGKQVLVMVVLACLMLDYSLCSARPRHWRPQNRFGKRERFEKTTFIPEEPTFLNPSRFGKLYRMIVSGGVLCLQQVTTEHISCFRLATENENEETGKEVQRISRYRYLADHN

>luqin_Brachiopoda_Glottidia_pyramidata_comp305_c0_seq1.p1

MNPLTRFLIVATLVILLGLFGLCRPQHWRPQGRFGKRTDAQNLQPFYQKANQPTSYTMSLLDPSGDSGAPPVLLIGGKTLCLKIDVEGNYKCISTRRRTRNYYRTQ

>luqin_Brachiopoda_lingulaAnatina.g29755.t1 Brachiopoda

METMNSLTRVLIVATLVILLGLFGRCRPQHWRPQGRFGKRMDTHKVQTYYQKVLPASQPSRYPASFTDSSGDGDDPPILLIGEGTLCLKVDMEGNYRCISTKRRMRNYYRTH

>luqin_Nemertea_Notospermus_geniculatus_g8373.t1

XXXLCLVFLLISQSEAQWRPQGRFGKRDPAAEGDFNSDDDLEAIDTASLEVAKTPIFATKDKICIKDANTEMYKCLRRRVRTGNAIYH

>luqin_Nemertea_Cerebratulus_marginatus_comp45345_c0_seq1_m.31503

MRSASYTYFLGALCIVLMLISHSEAQWRPQGRFGKRDPATGGEFNSEDDLEAVDTTSLEVAKTPIFATKDKICIKDANTEMYKCMRRRVRTANSIYH

>luqin_Nemertea_Cerebratulus_spec_TRINITY_DN34232_c0_g1_i1_m.164286

MRSESYIYFLGALCIVLMLVSHSEAQWRPQGRFGKRDPNTEAEFNSEDDLEAVDTHSLEVAKTPIFATKDKICIKDANTEMYKCLRRRVRTGNNIYH

>luqin_Nemertea_Lineus_lacteus_comp18583_c0_seq1_m.32473

MRSTSYTYFLAALCLVLMLISHSEAQWRPQGRFGKRDPATDGEFNTEDDLEAIDTASLEVAKTPIFATKDKICIKDANTEMYKCLRRRVRAGNNIYH

>luqin_Nemertea_Lineus_ruber_Lvir.rna.tri.45213.1_m.84196

MNSASYTYFLAALCLVLMLISHSEAQWRPQGRFGKRDPATEGEFNSEDDLEAIDTTSLEVAKTPIFATKDKICIKDANTEMYKCLRRRVRAGNNIYH

>luqin_Nemertea_Malacobdella_grossa_comp267972_c0_seq1.p1

MTRHLYVHVYVVVTLLLLFTLFISRGEAQWRPQGRFGKRTDGGDGTADGDLFNDNSELASTVAGAGGKLDLARVPIFTTENGYICVRQPETDLYRCLKKKEKS

>luqin_Nemertea_Baseodiscus_unicolor_Contig16927.p1

MIFPNEYGVLCLSG

MRTMGSIFVLGTLFIVLLLISESQQQWRPQGRFGKRDHEGDGDFTEETEIDGTKAANLEEAKTPIFATKDKICIKEGENGLYKCLRRRVRTGDLFN*

>luqin_Nemertea_Lineus_longissimus_c39300_g1_i1.p1

MRSASYTYFLAALCLVLMLISHSEAQWRPQGRFGKRDPATDGEFNSEDDLEAIDTASLEVAKTPIFATKDKICIKDANTEMYKCLRRRVRAGNNIYH*

>luqin_Nemertea_Tubulanus_polymorphus_comp146433_c0_seq1_m.146425

XXXXXXXXXXXXCVMLANSMVTTALYWRPQGRFGRQIGDNDIGDNGDIGDNDFAQLQETNLHKRQMQSLKRNQDDELA

## LXRX (LxRV)

>LXRX_Phoronida_Phoronis_australis_TRINITY_DN297370_c0_g1_i1.p1

MREIFVIVVLAISLFVFEGISAENDAKRKARDVEDVYNLNLASKHVVKRKNRRDTELELARVKRQRLDETNVELERVKRHRRNAADVDENSALERVKRNRRETDVSGVELQRVKRGRRDVELSREKRHRRDSDSALERVKRGKRNVVAAGEVELNRAKRHRRNSNHRHHKRRSSSRRDRIVKLKKRIAKLRHARHANKRKRNFSHHKP*

>LXRX_Phoronida_Phoronis_australis_TRINITY_DN297370_c0_g3_i1.p1

DENSALERVKRNRRETDVSGVELQRVKRGRRDVELSREKRHRRDSDSALERVKRGKRNVVAAGEVELNRAKRHRRNSNHRHHKRRSSSRRDRIVKLKKRIAKLRHARHANKRKRNFSHHKP*

>LXRX_Phoronida_Phoronis_psammophila_comp69869_c0_seq1.p1

MRGIFVQLVVLAVAVLIFEVSAENGAKRRERRETENVVNLKLLRKHEVKRKNKRDTTLALQRVKRQRRGIVSLNENGNSNVDVPLERVKRQRRELEKAAIELQRVKRNRRDVLNYAEADPQLERVKRNRRDDAEVELQRQKRDRRDVELQRVKRQRRDSDAELQRVKRGRRNVPGGDGAVELLWVKRQRRAVKVHHERKKERNNRRRAMEQKMKRFERRLKKLEHPHHHQWKNAKRNLHRRHFKRGHH*

>LXRX_Phoronida_Phoronis_psammophila_comp69869_c0_seq2.p1

MRGIFAQLVVLAVAVLIFEVSAENDAKSRKERETENEYTLKLLRKHEVKRKNKRDSTLELQRVKRQRRDIENGNSNVDVPLERVKRQRRELEKAAIELQRVKRNRRDVLNYAEADPQLERVKRNRRDDAEVELQRQKRDRRDVELQRVKRQRRDSDAELQRVKRGRRNVPGGDGAVELLWVKRQRRAVKVHHERKKERNNRRRAMEQKMKRFERRLKKLEHPHHHQWKNAKRNLHRRHFKRGHH*

>LXRX_Phoronida_Phoronis_ijimai_(P._vancouverensis)_comp95961_c0_seq1.p1

MKEIFVLVVLAISFFVFEGISAENDAKRKGRDVEDAYNLNLASKHVVKRKNKRDTNLELERVKRQRRDVSAVELQRVKRHRRNADENADSALERVKRNKRETEATGVELQRVKRGRRDVELSRVKRQRRDSDAALERVKRGRRNVAETGEVELQRAKRHRRNAKHGHHKRRFAGRRDRIAKLKKRIAKLRHARHVNKSKRNFRHHKA*

>LXRX_Phoronida_Phoronopsis_harmeri_c139830_g1_i1.p1

MRGTLSIVAALVVILCFVDVFANNDASRQKRDAEDVTYLGLKSIHTVKRKYKRDTGVDLQRVKRQRREAGNSVAELQRVKRHRREADASAADLQRVKRHRREAQDNAVDLQRVKRQ

## Myomodulin

>Myomodulin_Phoronida_Phoronopsis_harmeri_c97150_g2_i1.p1

TEVLRLGKRLYDQRRAMGMARLGKRPFEVDIDSDVFVPSPENRALATLRLGRSADKHTNKDNK*

>Myomodulin_Phoronida_Phoronis_psammophila_comp69409_c0_seq1.p2

MVSYRGWSWLCILAVVVMVTITTSEQSPVTNNRVRREALYERFNRAMGRVRLGKRPLHALRLGKRNNIGLFPAQEYEEDRFGYVYPADDREDLRRQLAIHPRIGRELENEEYETTILDDLFTLYKKLEAFF*

>Myomodulin_Phoronida_Phoronis_psammophila_comp69409_c0_seq2.p2

MKQFSSFVFSVVVMVTITTSEQSPVTNNRVRREALYERFNRAMGRVRLGKRPLHALRLGKRNNIGLFPAQEYEEDRFGYVYPADDREDLRRQLAIHPRIGRELENEEYETTILDDLFTLYKKLEAFF*

>Myomodulin_Phoronida_Phoronis_psammophila_comp69409_c0_seq1.p1

MKNTKRRFWMTSSLCTKSWRPFFDDSERDDDLRQASEPRLGRSKRAVPLQPLKDTEPSETKAEYGNVNPNKNTYDSFDEFLSNEKNTDDRAKRDDLLDDIRHYGIITPRLGLKLKRGSNIPAIRAESLLDIEEDKRATKLLRLGKRLFDEDDKRAMGIARLGKRPFEQDMDDALIPPPKNRAIPVMRLGRSTDKQTDKEKASS*

>Myomodulin_Phoronida_Phoronis_ijimai_(P._vancouverensis)_comp97889_c0_seq7.p1

MTGLIGNSSLKALIFFVVVALSTSEETESSRTRRDTSFERFNRAMGRVRLGKRPLHALRLGKREDSGLPLRNYAEDRFGFIFPESETEKEYERRQLAIHPRIGRELEAGLGESTILEDLYSVYKKLQLFFDDVQRDDDLRQTSEPRLGRSKRDVNQQDQSATENSKTNTPEKDLDSERFKREDLTEDFRHYGILTPRLGYRLRSNIPALGPDNEMIADKRAIAILRLGKRLNEKRAMGVARLGKRPFEATGNTIPSPENRALPVMRLGRSAEKQKRTTSD*

>Myomodulin_Phoronida_Phoronis_australis_TRINITY_DN317780_c2_g10_i1.p1

MTYLVRNFSLKALLVFAVVALASSEETSSSRTRRDTSFERFNRAMGRVRLGKRPLHALRLGKRGDSSLPLRNYVEDRFEFVYPESEIENAYERRQITKQPRFGRELEAGLDESAIIEDLYSVYKKLQFFFDDVQRDDDLRQTSEPRLGRSKRDVNQQNQPQMENSKGGDKANTLQKGIESERFKRGDLDLTEDFRHYGILTPRLGYKLRSNIPALGPENEMIANKRATALMRLGKRLNDNRAMGVARLGKRPFEVTGDTIPSPENRALPVMRLGRSANKQKRTTSD*

>Myomodulin_Brachiopoda_Novocrania_anomala_Nano.rna.tri2.77389.1.p1

MHWLVLTVCCIGLHIAKGAPLTEEDRVKRGMGMMRMGKRGLRMIRLGRSQNDDYPLNYPYHLSYLEERLRQIEKDLRESTRQAPLPRIGKDLSGLMYDDGARDKNEATSPGPRVGKDLQSQESEFPLYDVTAINDGNIRPHRYLLTRTRRSVDKRGDGGYTLPRLGQAEAKRTWQEPEDIPQSFLAHKLGLIITPTFNGDFSNEVDRAIPLPRIGRFEENLNSAQNAENNHIEHVLAQHQRQQSSTTNMQSNPTSDIQHKRKLNMLRLGKKSTGNEDGN*

>Myomodulin_Brachiopoda_Glottidia_pyramidata_comp31749_c0_seq1.p1

MHVLATATICILFQAFYGYLADGFERDTRGLGIMRMGKRASQLKMIRLGRGMRMLRLGKRGDPYMYGYPYDFFYEPSNANAEQFRRQIPTYPRIGKDLDNTPFAWLAANPRPRVGKELQNYEWTAAEEADRDAALPFVLSRKKRSADESHYDQKKGSPSLPRLGEPTAKRSDDEPYPYLPHDDYNKYVRGMIPYPRIGRYEPQEMPPAYEEGPYYDEYADKRAFRGLRLGKRMRMLRLGRNSAPKTDDISNFNTDYNQNEEKRGMRMLRLGKRSENSEISKVPSTENEKATKN*

>Myomodulin_Brachiopoda_Lingula_anatina_comp122974_c0_seq1.p1

MHVLATATICLLFQAFHGYSADGFERDTRGLGIMRMGKRANQLKMIRLGRGMRMLRLGKRGDPYMYGYPYDLFYEPRNDIAEQFRRQIPTYPRIGKDLDYAPFSEEWNWPAANPRPRVGKELQDYEWAAAEEANHDASLPFVLSRKKRSAVDRPESHYDEKKGSPSLPRLGEPTAKRSDDEPLPYLPNEDYEQYVRGIIPYPRIGRYEAYDMPPVYDEDPYFDTYADKRAFRGLRLGKRMRMLRLGRSDSQKNKRPFKRGF*

>lingulaAnatina.g16064.t1 Brachiopoda

MHVLATATICLLFQAFHGYLADGFERDTRGLGIMRMGKRANQLKMIRLGRGMRMLRLGKRGDPYMYGYPYDLFYEPRNDIAEQFRRQIPTYPRIGKDLDYAPFSEEWNWPAANPRPRVGKELQDYEWAAAEEANHDASLPFVLSRKKRSAVDRPESHYDEKKGSPSLPRLGEPTAKRSDDEPLPYLPNEDYEQYVRGIIPYPRIGRYEAYDMPPVYDEDPYFDTYADKRAFRGLRLGKRMRMLRLGRSDSQKTNDLSKEDSDFSQHEEKRKMRMLRLGKRSENSKISKVAPAKNEIASKN

>Myomodulin_Brachiopoda_Hemithris_psittacea_comp18643_c0_seq1.p1

MMKTVLKKILFVAAVTAIFCSIHGQANENGERNTRSLGILRLGRRASLDKRMRVLRMGRAFGINRVDKLGRRDDSRRQTTYPRIGKDLSSQFDLDPSVLAAYRLLYGDLRPPPRVGKEVTQDDRQAVSAINKLLRQYNSQSESGSIQKDMVRMLRSLGLDLGSQKRDVIPRLGHLTEKRDNVIIPRLLGNPSQKREGPSMLSHLGAFFRKRDVFTPRHGHFYRKRDIIPRLGSSLQKRGFIIPRLGSSKRNGITPRLGEVRRSHSDSTLFQQPNSLLGERLMKQEATDAGKTSFDRSVTYPRIGRPSAIEAYIQKRMKILRMGKKLRALRLDTK*

>Myomodulin_Brachiopoda_Terebratalia_transversa_Ttra.rna.tri.14671.1.p1

MIPDREKIWVYICVLVTITNLGSALDGLGGTAYLHSNLLRTSRGLGILRLGRRTMNMDKRLRILRMGRAMHPHKDVYNSVDGSEDSDLYRRQTIWPRIGKDLESQYQYEDAAPFISKGGSRPPPRGGKELSNDIKQLLSYAETLLRSEKFKLNSRSDRALGYSQLPRLGELTEKRSNRPILLPRLGGRPTYKRSLRKRSVADPKRASFMLPRLNGKLDQKRGRFNLPRLSGELHQKKRGGFLSPRLGQEKRAFLTPRLDGQSKRGRFLSPRLGGEANKRGSFNSPSLVKEFEERRNNVLNKNMSHREDVDKSDNANSRDRSVLYPRIGRISSLPRSYVKRLKILRMG*

>Myomodulin_Brachiopoda_Laqueus_californicus_comp40234_c0_seq1.p1

MIPHTEKPWVFICVFMTMTNLGSALDGLEGSAYLNSNFFRTTRGLGILRLGRRTMNMDKRLRILRMGRAMHPHKDFYNSLGDSDDSDLYRRQTIWPRIGKDLESQYQYEDESFMPGGGYRPPPRTGKEISSDVNQLLNYAETLLHNEKFKLNSKSDRALGDSQLPRLGELTEKKRYVLLPRLGTYKRSIRKRSADTPKRASFLLPRLNGMFDLKRGRFHLPRLSGELDQKKRGGFLTPRLGEEKRGRFLTPRLGGQAKRGRFFTPRLGGEAKRGSFINPRLGGKESDEKRNNVISDKVSSKDNSDNKNSDRSVLYPRIGRISNLSKANVQELKSYVKRLKILRMGKKSN*

>Myomodulin_Nemertea_Cerebratulus_marginatus_comp1878316_c0_seq1_m.160602 3prime_partial

MGMIRMGKKDQEMDKKSLRLVRMGRAMGMLRMGKRDDEDAATGNNNEELSKRAMHLIRMGKRAMGMIRMG

>Myomodulin_Nemertea_Cerebratulus_marginatus_comp1507703_c0_seq1_m.150694 internal

FRAIPYPRIGKDLDELEAHKRAMGMIRMGKKAMGMIRMGKRDETEGVDKRAMGMIRMGKKAMGMIRMGRAQE

>Myomodulin_Nemertea_Cerebratulus_marginatus_comp1908569_c0_seq1_m.161958 3prime_partial

MIRMGRSLGMIRMGKRDLRMLRMGRSYNEDDLSGRQVTYPRVGKDLEGEEVLRSNPSLPRLGKDIPVYNGLLLDQLLKEVHNHE

>Myomodulin_Nemertea_Cephalothrix_hongkongiensis_comp10015_c0_seq1_m.5009 internal

APEFHQSCFRLHFLYPRIGRELNKLPEKEHVKRAMGLVRMGKKRAMGLVRMGKRDFENDDEKRAMGLVRMGKRQLDDEDFDKRAMGLVRMGK

>Myomodulin_Nemertea_Cephalothrix_hongkongiensis_comp10015_c0_seq2_m.5012 internal

NSDEIREQYARIPQYPRIGRELNKLPEKEHVKRAMGLVRMGKKRAMGLVRMGKRDFENDDEKRAMGLVRMGKRQLDDEDFDKRAMGLVRMGK

>Myomodulin_Nemertea_Cephalothrix_hongkongiensis_comp370881_c0_seq1_m.114641 internal

SSDLVELSTFISSAAFILLVTGIVCATDDAAESNEKRAMGLVRMGRAMGLVRMGKKRSADDELDLYSRQPQWPRIGKDLENLELDEFRRGQQ

>Myomodulin_Nemertea_Cephalothrix_hongkongiensis_comp7309_c0_seq1_m.3723 complete

MDDEKRAMGLVRMGKRAMGLVRMGKRNDEEEVDKRAMGLVRMGKRGLDGEEEKRAMGLVRMGRAMGLVRMGRGSEKRAMGLVRMGKRSAENKS*

>Myomodulin_Nemertea_Nipponemertes_spec_TRINITY_DN27524_c0_g1_i1_m.61135 internal

WKRSFACPRYKPHVTGKSQPAID

MFYQGYRVTLLVVVLSHIQGFCRATDSRSKREIPMMRMGRSLHMIRMGRSFDNDQPAEERGTRFPGPRMGRDSSEVDDLGTNAYSRLRNAVYSQVLLNQILDDLRSHKRFARSAES

>Myomodulin_Nemertea_Nipponemertes_spec_Contig10220_m.121310 5prime_partial

GMIRMGKREDLKRALGMIRMGKRNLGMIRMGRGLGMIRMGRSDADDKRALGMIRMGKKDDYEKRALQMVRMGKRDDDKRGLSMIRMGKRDGAAEKSD*

>Myomodulin_Nemertea_Riseriellus_occultus_TRINITY_DN34238_c0_g1_i2_m.102070 complete

MFLSGMTSLSHDRHKRVLCTLRLGRGLNMLRLGRGLNMLRLGRGLVRRQEYYTEDRQKRNECQGVPLRTDCGGTCEKCWDFNDGRGPLCVNMANIPH*

>Myomodulin_Nemertea_Riseriellus_occultus_TRINITY_DN34238_c0_g1_i1_m.102066 5prime_partial

MKLFILSLVFCMFLSGMTSLSHDRHKRVLCTLRLGRGLNMLRLGRGLNMLRLGRGLNMLRLGRGLVRRQEYYTEDRQKRNECQGVPLRTDCGGTCEKCWDFNDGRGPLCVNMANIPH*

>Myomodulin_Nemertea_Riseriellus_occultus_Contig26131.p1

RRGRDGLLRRTSSLSRSLPRLGKDLEGIPVYNGLLLDQLLKEVHNHQRYARSVKEENSTHVSEEESKERAIPFPRLGKDLTELDEGDRAIPYP

>Myomodulin_Nemertea_Riseriellus_occultus_TRINITY_DN48720_c2_g7_i1_m.111674 3prime_partial

MIRMGKRDDEEAGTDEELSKRAMHLIRMGRKRAMGMIRMGRGNSDDGTDEEKRAMHLIRMGKRSASETAQS

>Myomodulin_Nemertea_Riseriellus_occultus_TRINITY_DN48720_c2_g4_i1_m.111671 3prime_partial

MPSPVVKLTLGVAILTFIQGVCLGTDSDDTSMRERRAVPMIRMGRSLGMIRMGKRDLRMLRMGRSYPDGEDLIGRQVSYPRIGKDLESEEVLRSNPSLPRL

>Myomodulin_Nemertea_Riseriellus_occultus_TRINITY_DN48720_c2_g1_i1_m.111666 internal

GDRAIPYPRIGKDLNELEAYKRAMGMIRMGKKAMGMIRMGKREEEEKRAMGMIRMGKKAMGMIRMGRAQEDPSSEEKRAMGMIRMGRSQLDDDAEKRAMGMIRMGKRDEGVNKKSLRLVRMGRAMGMIRMGKRD

>Myomodulin_Nemertea_Malacobdella_grossa_comp15339_c0_seq1_m.7854 5prime_partial

VVLMVATLSSQGFASDSRTKRSIPMLRMGRSSEWRPRMGRMSEDTDEPEQYKNHLDRLDSLYPVDFDADSNDENKDQVDRAIPYPRVGRALNMFRMGKRPLNMFKMGRGSDKRALHMIRMGRAMHMIRMGRAGNTDNGEAKKRAYNAGEAKKRALQMIRMGRNYVAEKRALNMLRMGKRDDDQKRALAMIRMGRSEQKST*

>Myomodulin_Nemertea_Cerebratulus_spec_TRINITY_DN37546_c0_g3_i1_m.89532 3prime_partial

MGMIRMGKRAMGMIRMGRAQEDPIAISERAMGMIRMGKRAMGMIRMGRAQEDPIAISERAMGMIRM

>Myomodulin_Nemertea_Cerebratulus_spec_Contig1514_m.198960 complete

MPSSAVKLTLGVAILSFIQGVCLGTESDDTSLRERRNVPMLRMGRSLGMIRMGKRDLRMLRMGRSFNEDELTDRQVTYPRVGKQIEDQATRSNPSLPRLGKDIPVYNGILLDHLLKEVRNHQGENESAPTDTKANEIEEDSMERAIPFPRLGREEEDRAIPYPRIGKDLEKTDAYKRAMGMIRMGKKAMGMIRMGKREPDEAMEKRAMGMIRMGKRAMGMIRMGRAQEDPIAISERAMGMIRMGRSGVDENEYKRAMGMLRMGKKDQEMDKKSLRLVRMGRAMGLIRMGKRDDENAAGSEELSKRAMNLIRMGRGLDKRGMGMIRMGRGDAEEQKRGMRLIRMGRSKGDEETESEKQ*

>Myomodulin_Nemertea_Lineus_lacteus_comp25533_c0_seq2_m.89885 complete

MPSPVVKLTLGVAILTFIQGVCLGTDSDDTSMREKRAVPMIRMGRSLGMIRMGKRDLRMLRMGRSYPGGEDLIGRQVSYPRIGKDLESEEVLRSNPSLPRLGKDLGGIPVYNSLLLDQLLKEVHNHQRYARSVKEENSNHVSEEDSKERAIPFPRLGKDLTELDEGDRAIPYPRIGKDLNELDAYKRAMGMIRMGKKAMGMIRMGKREEEEKRAMGMIRMGKKAMGMIRMGRAQEDPSSEEKRALGMMRMGRSQLDETDKRAMGMIRMGKRDNEMDKKSLRLVRMGRAMGMIRMGKRDDEEAGSDEVSKRAMHLIRMGRKRAMGMIRMGRGNSDEAQDEEKRAMHLIRMGKRSVSETAQNAKQ*

>Myomodulin_Nemertea_Lineus_longissimus_c36724_g1_i1_m.39104 complete

MPSPVVKLTLGVAILTFIQGVCLGTDSDDTSMRERRAVPMLRMGRSLGMIRMGKRDLRMLRMGRSYPDGEDLIGRQVTYPRVGKDLESEEVLRSNPSLPRLGKDLGGIPVYNGLLLDQLLKEVHNHQRYARSVKEENSNHVSEEDSKERAIPFPRLGKDLTELEEGDRAIPYPRIGKDLNELEAYKRAMGMIRMGKKAMGMIRMGKREEEEKRAMGMIRMGKKAMGMIRMGRGQEDPSPEEKRAMGMIRMGRSQIDDVTDKRAMGMIRMGKRDDEMDKKSLRLVRMGRAMGMIRMGKRDDEEAGSDELSKRAMHLIRMGRKRAMGMIRMGRASSDEGTDEEKRAMHLIRMGKRSASETAQNAKQ*

>Myomodulin_Nemertea_Lineus_ruber_Lvir.rna.tri.25711.1_m.51554 complete

MPSPAVKLTLGVAILTFIQGVCLGTETEDTSMREKRAVPMLRMGRGLGMIRMGKRDLRMLRMGRSYPDSEDLIGRQVTYPRVGKDLGNEEALRSNPSLPRLGKDLGDIPIYNGLLLDQLLKEVHNHHRYVRSVKEENSNHVSDEDSKERAIPFPRLGKDLTELEEDDRAIPFPRIGKDLNELEAYKRAMGMIRMGKKAMGMIRMGKREEEEKRAMGMIRMGKKAMGMIRMGRGQEDPSSEEKRAMGMIRMGRSQLDDETDVKRAMGMIRMGKRDDGVDKKSLRLVRMGRAMGMIRMGKRDDEEAGSDDELSKRAMHLIRMGRKRAMGMIRMGRGSSDEATDEEKRAMHIIRMGKRSASETAQNAQQ*

>Myomodulin_Nemertea_Notospermus_geniculatus_g8085.t1

MPSPAVKLTLGVAILTFVHGICLGTENDDTATREKREVPMIRMGRSLGMFRMGKRNIRMLRMGRSYNDVDDFLPRQVTYPRVGKDLDSEEALRSNPSLPRLGKDIESIPVYNGILLDQLLNAVHSHQRYARSVSAEGDKSNHIVTEEGDSKERAIPFPRLGKDLNEVGEVDEADRAIPYPRLGKDLGEMEEYKRAMGMIRMGKKAMGMIRMGRAQEDVKRALGMIRMGKRAMGMIRMGRAQEDPVSEAEKRAMGMLRMGRSGLDDTEYKRAMGMLRMGKKDQEKKSLRLVRMGRAMGMLRMGKRDDEQASGTDEELTKRAMNLIRMGKRAMGMIRMGRGNEGNEEEAQEEDKRAMHFIRMGKRSVNEAAQNEKQ

>Myomodulin_Nemertea_Baseodiscus_unicolor_TRINITY_DN102321_c0_g1_i2_m.119594 internal

QLLVVVGSRARGNVNLFRLLCVLKRSKVPKGRESMLFPLLKLTLGVATLAILQGACLAKDDDASDTRDAPLIRIGRSLGMVRMGRRDLNMVRMGRSYDEDNLLGRQVTYPRIGKSLQKVVEELRSNPTLPRLGKDLYNDILLDEFAKEAAEQHSRYARSTQDDSGSQERSIQFPRLGREELLADANRIDADRAIPYPRIGKNLDELDSYKRAYGMIRMGKRDENKRAMGMIRMGKKDYSDMDKKALGMVRMGRAMGMIRMGRGGEGADEEKRALGMVRMGRNQDEDKEFKRAMGLLRMGKKGAEDELQKKALGMVRMGRAMGMIRMGKRDEEQLDEVPDTANDVSKRGMHMIRMGKKALGLIRMGKKINADTIDKRAADSFLRVGKRSQASSSEKPFDAD

## ND peptides

>ND_Nemertea_Riseriellus_occultus_TRINITY_DN37159_c1_g1_i3_m.70794 internal

GDDKRHGAEERHGDDEKRLWNDKRHGDDKRNGDDKRHGGDEKRLWGHGEDDKRLWNDKRHGDDKRHGGEKRHGDDEKR

>ND_Nemertea_Riseriellus_occultus_Contig25510_m.152858 internal

HGDDEKRLWNDKRHGDDEKRHEDDKRHGDDEKRHWDDKKNGDDEKRHEDDKRHQRDDDKRLWNDKRHWDDKRHDGKEHGDDKRRHGDDKKSLWNEKRHGDDEKRLWNDKRHG

>ND_Nemertea_Riseriellus_occultus_Contig9058_m.135111 internal

NDKRHGDDKRHGDDEKRLWYDKRHENDKKHGGEKHATDDKRLWNDKRHEDDKRHDGKAREDEKRHGDDKKSLWNDKRQGFEIYAKKNKDAAKAVEQLKELQKGMHRDARRDKEILTYYKR

>ND_Nemertea_Riseriellus_occultus_TRINITY_DN37159_c1_g1_i2_m.70789 internal

CLWEHDEDDKRMWNDKRHGDDKRHGEKEPEDEKRHSGDEKRLWGHGEDDKRLWNDKRHGDDKRHGGEKRHGDDEKRLWNDKRHGDDKRHGDDKRHGGDEKRLWGHDEDDKRMWNDKRHGDDKRHGEKEPEDEKRHSGDEKRLW

>ND_Nemertea_Riseriellus_occultus_TRINITY_DN37159_c0_g1_i1_m.70783 internal

ETHANDDKRLWNDKRHGDDKRHGGKEHEDEKRHGENERHKNDKKRLWDDKRHGDEKRHEKDDKRLWGRDEDDKRLWNDKRPGEDKRHGEDKRHEEDKRHGEDKRHGRKEHEEDKRHGDKTQHKEDEKRLWGHDEDDKRLWSDKRHGDDKRHGGDEK

>ND_Nemertea_Lineus_longissimus_c35010_g1_i1.p2

MKAVHVTFALTLLAMTVASAKESSLDQKTTGVKTHSVERKSGQAVEPLEEQGIFLARRIHQKNAEGALKRLSGEKRPGENKKQEGKDKVDDKRHETDKRHEVDQSHGGETHAGDDKRLRNDKRHGDDKRHGGKEHEEDKRHGEKKETRGFKET*

>ND_Nemertea_Lineus_longissimus_c36981_g1_i1.p1

GEEKRHGEDEKRHWDDKRHGDNEKHGEEKHTKDDKRLWNDKRHGNDKRQGGKAGEDDKGHGDVKKGLWNDKRQGFEIYAKKNKNAAEAVAQLKELQKGIHRDARRDKEILTYYKRGQDHGV*

>ND_Nemertea_Lineus_longissimus_c35010_g1_i1.p1

MLKVHSSGCRERRGQERIRNRKGKTRWTTRDMKPIRDMKLIRVMAVKRMLVMINVLGMINDMGTTRDTVVKNMRKTKGMGKKKRHEDLKKHEGDEKRLSGHDEDDKRMWNDKKRGSEKHEEDAKRLWNDKRHGDDKRDGKKEHDDDKRHETQHGKDDKRQGDEERHREDKGIGDDEKRL

>ND_Nemertea_Notospermus_geniculatus_g35134.t1

MVHSIFALAVLVLATVHGEERAEDEKTLGIKRHEVERKMGGGGFKRNKADPLEEQGIFLARRRHGDDKRHGDDKRHGDDKRHGDDKRHGDDKRGLWNDKRHGDDKRHGDDKRHGDDKRHGDDKRGLWNDKRQGFGIYARKRNDAALAADQLEALQPHRDARREKEMLTYYKRA

>ND_Nemertea_Notospermus_geniculatus_g23324.t1

MKVKMVHSIFTLAVLMLATVHGEERADDEKTLGIKRHEVERKMGGGGFKRNKADPLEEQGIFLARRVQEKKNEAALKQLMDDKRHGDDKRHGDDKRHGDDKRHGDDKRGLWNDKRHGDDKRHGDDKRHGDDKRGLWNDKRQGFGIYARKRNDAALAADQLEALQPHRDARREKEMLTYYKRA

>ND_Nemertea_Lineus_ruber_Lvir.rna.tri.74630.1.p1

RLWDDKRHGGKEQNGGKRHGDEEKRLWDDKRHYGENKKHGGEKHADDKRLWDDKRQGFEIYAKKNKDAAKAVAELRELQKGKHRDVRRDKEILTYYKRGL*

>ND_Nemertea_Lineus_ruber_Lvir.rna.tri.53724.1.p1

MKAVHAIFALALLAMTVTSAKEKSLHEKILGAKTHSVVRKSGQSAVPLDEQGIFLARRIHQKNAEAALKSVRGEKSKGDEKRHEGHEDDKRHGAAKKHGDHETHGDDKRLWNDKRHGDVDNKRHGVGQKLGGKTHGAEEKRLWDDKRHGDDKIHAGREQEDDKRHGNEEKRLWD

>ND_Nemertea_Lineus_ruber_Lvir.rna.tri.18336.1_m.37836 internal

LWDDKRHDDDDKRHGNDEKRLWDDKRNNNKHSGEKHSDDDKRLWDDKRHGDDKRHGGEKRLWDDKRLWNDKRHGDDKRHGGDEKRFWGHAEDDKRLWNDKRQGHDVEHEEENRHGDDEKRLWDDKRHHGEGKRHGGETHGDDDKRLWDDKRHADEKRHGGKEREDDKRHHGEEKKHGQEKHDD

>ND_Nemertea_Lineus_lacteus_comp18680_c0_seq1.p1

MKAVHATFALTLLAMAVTGAKENSLHQKTIGVKAHSVERKSGKAVEPLEEQGIFLARRIHQKNTEGALKRLREEKGHGENKKHEGPVTEEEKRHGADKRHDDEQRGRSETHAADDKRLWNDKRREDDKKHGAKEDEEGRKRGDIERLEDDEKRLWNDKRHGDEKRHEDDERHGEERLGDEKRHMDDEKRLWN

>ND_Nemertea_Cerebratulus_spec_TRINITY_DN17706_c0_g1_i1.p1

EDDKRHGNDKRLWGHEEDDKRHADDKRHGGVDKRLWGHDEDDKRHADDKRHGGIEHEDDKRHGQDKRLWNDKRHGGANHEDVKR

>ND_Nemertea_Cerebratulus_spec_TRINITY_DN33365_c1_g1_i5.p2

RLWGHEEDDKRHADDKRHGGDDHEDVKRLWGHEEDDKRHADDKRHGGVEHVDDKRHGNDKRLWNDKRHGGEDHKDDKRHGDDKRLWGHEEDDKRHADDKRHGGDDHEDVKRL

>ND_Nemertea_Cerebratulus_spec_TRINITY_DN33365_c1_g1_i1.p2

WNDKRHGGEDHEDDKRHGDDKRLWGHEEDDKRHADDKRHGGDEHGDDKRHGNDKRLWNEKRHGGEDHEDVKRLWGHEEDDKRHADDKRHGGVEHVDDKRHGNDKRLWNDKRHGGEDHKDDKRHGDDKRLWGHEEDDKRHADDKRHGGDDHEDVKRL

>ND_Nemertea_Cerebratulus_spec_TRINITY_DN33365_c1_g1_i10_m.119972 5prime_partial

RHADDKRHGGVEHGDDKRHGQDKRLWNDKRHGGDTHGADKRHEDDKRHGNDKRLWGHEEDDKRHADDKRHGGVEHGDDKRHGQDKRLWNDKRHGGEDHEDDKRHGNDKRLWNDKRHGGDNHNTDKRQHDKRQGFEIYAKKNKEGAQAAQQLQALEKGLHRDSRRDKEILTYYKRALSAGI*

>ND_Nemertea_Cerebratulus_spec_TRINITY_DN19057_c0_g1_i1_m.93235 3prime_partial

MKAGMVPTICALSLTLVTLTLIEGKESIDAKTIGVKRHVVEKKPGHEPFPMYDRGMDRKGAKRNGADPIEEQGIFLARRFQQKNAAAALKRLWGEKRHAGDGHGDEKRHGDEKRLWGHEEDDKRHADDKRHGQDKRLWNDKRHGAEEHEDAKRHHEDDKRHGNDKRLWGHEEDDKRHADDKRHGQDK

>ND_Nemertea_Cerebratulus_spec_TRINITY_DN33365_c1_g1_i2_m.119950 internal

RHGGVEHEDDKRHGNDKRLWGHEEDDKRHADDKRHGGDEHGDDKRHGQDKRLWNDKRHGGEDHEDVKRLWGHEEDDKRHADDKRHGGVEHEDDKRHGNDKRLWGHEEDDKRHADDKRHGGVEHGDDKRHGQDKRLWGHEEDDKRHAADDKRQDKRLWNDKRHGDDKRHGDDKRLWGHEEDDTKRHADDKRHGGDTHGADKRHEDDKRHGGVEHVDDKRHGQDKRLWNDKRHGGEDHEDDKRHGNDKRLWGHEEDDKRHADDKRHGGDDHEDVKRL

>ND_Nemertea_Cerebratulus_spec_TRINITY_DN33365_c1_g1_i3_m.119953 internal

LWGHEEDDKRHAADDKRHGQDKRLWNDKRHGGDTHGADKRHEDDKRHGNDKRLWGHEEDDKRHADDKRHGGVEHGDDKRHGQDKRLWNDKRHGGDNHEDDKRHGNDKRLWGHEEDDKRHADDKRHGGDEHGDDKRHGNDKRLWNEKRHGGEDHEDVKRLWGHEEDDKRHADDKRHGGVEHEDDKRHGNDKRLWGHEEDDKRHADDKRHGGVEHGDDKRHGQDKRLWGHEEDDKRHAADDKRHGQDKRLWGHEEDDKRHAADDKRHGQDKRLWNDKRHGGDTHG

>ND_Nemertea_Cerebratulus_spec_TRINITY_DN33365_c1_g1_i6_m.119963 internal

RHGGVEHEDDKRHGNDKRLWGHEEDDKRHADDKRHGGDEHGDDKRHGQDKRLWNDKRHGGDNHEDDKRHGNDKRLWGHEEDDKRHADDKRHGGDEHGDDKRHGNDKRLWNEKRHGGEDHEDVKRLWGHEEDDKRHADDKRHGGVEHEDDKRHGNDKRLWGHEEDDKRHADDKRHGGVEHGDDKRHGQDKRLWGHEEDDKRHAADDKRQDKRLWNDKRHGDDKRHGDDKRLWGHEEDDTKRHADDKRHGGDTHGADKRHEDDKRHGGIEHGDDKRHGNDKRLWNDKRHGGEDHEDDKRHGDDKRLWGHEEDDKRHADDKRHGQDKRLWNDKRHGGEDHEDDKRHGNDKRLWGHEEDDKRHADDKRHGGDDHEDVKRL

## NdWFamide

>NdWFamide_Phoronida_Phoronis_ijimai_(P._vancouverensis)_comp19943_c0_seq1.p2

SARCHWYGKRGDTHPSLRDYINQKKEPTDSQTRTKLTFQTDADVLRYIKLVLSSWEEAQVKK*

>NdWFamide_Phoronida_Phoronis_australis_TRINITY_DN299880_c0_g2_i1.p1

MKQLLVLLLVVFVLDTVKCHWYGKRGDTHPSLRDYVNQKKESTDSQTKTKLTFQTDADVLRYIKLVLNTWEEAQAKK*

>NdWFamide_Phoronida_phoronisPsammophila.14906

MRQTCVVLLVVLTITLTVNAHWYGKRGDPNPSLRDYMNNKMDTTDRQSKMERVFRTDADVLQYIKYILNSWEEIRSKK

>NdWFamide_Nemertea_Nipponemertes_spec_TRINITY_DN26159_c0_g5_i1.p1

MSRTLIALVVLAVVCATVSGNWFGKREDESTFKDYLQLVKATVNSPYTDSTHVLKTIGYII

>NdWFamide_Nemertea_Tubulanus_polymorphus_comp24000_c0_seq1_m.13134

MSRSILFLLFLAVISVTVTQANWFGKRGDDVNAFKQYLANSRQNPLTDSAHVLRMISFIIKSYETRGSDHIHSPN

>NdWFamide_Nemertea_Cerebratulus_spec_TRINITY_DN37315_c6_g1_i2_m.72185

MSKLTIALFVMAVLIAVTQANWYGKREDNKFQDYLQAIKSSTHDSTYDAERVLKSIAYIVHAWESKERAEEVATRSS

>NdWFamide_Nemertea_Lineus_lacteus_comp5955_c0_seq1_m.6622

MSRLSIVIFVMAVLVALTQANWYGKREDNKFQDYLQAIRSSTQDSTYDAQRVLKSIAYIVHAWESKERAVEDATRSSS

>NdWFamide_Nemertea_Lineus_longissimus_c40741_g2_i1_m.61202

MSRLSIVIFVMAVLVTLTQANWYGKREDNKFQDYLQAIRSSTQDSTYDAQRVLKSIAYIVHAWESKERAVEDATRSSS

>NdWFamide_Nemertea_Lineus_ruber_Lvir.rna.tri.17489.1_m.36221

MSRISIVIFVMAALVALTQANWYGKREDNKFQDYLQAIRSSTQDSTYDAQRVLKSIAYIVHAWESKERAVEDATRSSS

>NdWFamide_Nemertea_Baseodiscus_unicolor_TRINITY_DN102586_c0_g1_i2.p1

MSRLTVVIFVIAFLVALTQANWYGKREDPNGFKEYLEAIKSTSSHNDAERIIRTIASIIHAWDQSKGDEVATSSS*

>NdWFamide_Nemertea_Paranemertes_peregrina_comp28371_c0_seq1.p1

MASRTVLVLLVLSVMCSVAMGNWFGKREDNENLKDYLEMVKASKNSPYSRSSEQVLKTIASIVRGWEEAQQTRSTRAAN*

## Neuroparsin

>Neuroparsin_Phoronida_Phoronis_psammophila_comp66987_c0_seq6.p1

VTSDGCCLVHQGCECLPDSCQTIDCPSNMHKRVVTPALGHPGFCCSKYECVNVSTMSCNHEGIEFQHEETWKLDQCTTCTCRGG

>Neuroparsin_Phoronida_Phoronis_psammophila_comp726718_c0_seq1.p1

GETCRGVAFHATVIFVFIASLASLTGARRSRDSDKLSLDSSEQTTLNCPPCDKIHCAPKKASKLKCSGGITKGICNCCPVCAKVEG

>Neuroparsin_Phoronida_Phoronis_psammophila_comp66987_c0_seq3.p1

MGWSVIFTLLFILPSVLCYLCPVCDEDLCHNVDVGSCAGGLVMDNCDCCLVCARTLNQTCGGEYGLSGKCDKGLICAYSPQHGAPLSHGHVGICIGKQDFLYSEKIQV

>Neuroparsin_Phoronida_Phoronis_psammophila_comp688178_c0_seq1.p1

LYCEAQKSRNPYGPKNDVLVGKCKKSPRHVGDEGIPGYCRPKCTAKFCKANPKAICSAVDVAEVKQHCQGDCQHTSCQACRFADYEPDCDKCSKDDFSCMKKYAKCVKRQFCTRN

>Neuroparsin_Phoronida_Phoronis_psammophila_comp66987_c0_seq1.p1

MGWSVIFTLLFILPSVLCYLCPVCDEDLCHNVDVGSCAGGLVMDNCDCCLVCARTLNQTCGGEYGLSGKCDKGLICAYSPQHGAQLSHGLIGICIECEESFEGCTVVDSLCVCDKSCVDIFEFVDEQSCQNSLKETVNNTNLHSNCSHVQCEVMFMPQCPPDSTPVKTESDTCCPQPPICQCQMDLCTRHTCAPGSDLTLIKGGLGTPGQCCDEFECTPSDNDCRDVHCPLDELGDVDCPTDSYRLPSLVTSDGCCLVHQGCECLPDSCQ

>Neuroparsin_Phoronida_Phoronopsis_harmeri_c114918_g2_i1.p1

DEGIYCRPKCTAIFCKANPKAICSAVDVGEVKQKCQGNCQHTSCQACRFADYEPDCDKCGKDDFTCMKK

>Neuroparsin_Phoronida_Phoronopsis_harmeri_c132120_g4_i2.p1

MGWLFIVFVTLCVHTAQGLVCPACEECPSPPENCPGGLALDRCDCCIVCAKVTNQTCGGKYGLNGKCDTGLICAISPEHGSAISDNEIGICQECEESFDGCTIVGSTCVCDTSCSDIFKYADEASCLNALSYNGKLSFCTLKSIQRLHFT

>Neuroparsin_Phoronida_Phoronopsis_harmeri_c113175_g1_i1.p1

MLGTVHMMLSTQRLVFYSIMTLCFIASQSSARKPNDGINLDSGRSPTPLRCPPCKQIGCPGPKKASKLKCKGGFSTDICNCCPACAKVEGEDCGGEWNYLGKCDAGLYCEAQKSMNPYGPSRENDVLVGRCKKSKYTYYTTPN

>Neuroparsin_Phoronida_Phoronopsis_harmeri_c132120_g4_i5.p1

MGWLFIVFVTLCVHTAQGLVCPACEECPSPPENCPGGLALDRCDCCIVCAKVTNQTCGGKYGLNGKCDTGLICAISPEHGSAISDNEIGICQECEESFDGCTIVGSTCVCDTSCSDIFKYADEASCLNALSYNVNSTVTESNCTGVQCGVMFMPQCPPDSLPLRQEMESCCPAPPTCHCQMDICTRQTCAPGFDLTLLRGGL

>Neuroparsin_Phoronida_Phoronis_australis_TRINITY_DN305807_c1_g2_i1.p1

MRRSGEAVVVYLLVFSLSSLTAGRKAKYSLGLSSHTERAALSCPPCKEIHCTPKKASKLKCKGGITHGVCDCCPVCAKLEGEPCGGEWNYLGKCDTGLYCEAQKNPNPYGPKNEDVLVGKCKTNPRHAGDEAIPGYCQPKCTPKFCKANPKEICSAVDVAEVKQECQGECQHTSCQACRFADYDPDCIKCSKDDFSCMKKYAKCVKKQYCTRNKFPCRKAAYRLKEAGKFQCMVPPCLKH

>Neuroparsin_Phoronida_Phoronis_ijimai_(P._vancouverensis)_comp670658_c0_seq1.p1

RIRSLISFRIRDMRRSGQAVVAFSCLLVLSLSSLAAARRAKQSLGLSSHTERAALSCPPCKEIHCTPKKASKLKCKGGITHGVCNCCPVCAKLEGEPCGGEWNYLGKCDTGLYCEAQKNPNPFGPKNEDV

>Neuroparsin_Phoronida_Phoronis_ijimai_(P._vancouverensis)_comp92621_c1_seq5.p1

MGWLFVFLSVIVLSSSHGFLCPACDESLCPTPTECPGGLVLDNCDCCLTCAKTLNQTCGGEYGLSGKCDKGLICAISPQHGSLLSSTEDGQIGICQECSESFEGCTVINSVCVCDTSCSDIFEFADESSCLNSLTYFVNETEVASNCSGVQCGVMFMPQCPPDSVAVKSLSGPCCPLPPTCECQMEFCTREVCAPGSDLTLVRGGLGTPGRCCDEFECTPPGKLVYHK

>Neuroparsin_Phoronida_Phoronis_ijimai_(P._vancouverensis)_comp92621_c1_seq4.p1

MGWLFVFLSVIVLSSSHGFLCPACDESLCPTPTECPGGLVLDNCDCCLTCAKTLNQTCGGEYGLSGKCDKGLICAISPQHGSLLSSTEDGQIGICQECSESFEGCTVINSVCVCDTSCSDIFEFADESSCLNSLTYFVNETEVASNCSGVQCGVMFMPQCPPDSVAVKSLSGPCCPLPPTCECQMEFCTREVCAPGSDLTLVRGGLGTPGRCCDEFECTPPENHCRDVHCPLDELADVDCPSDSYRLPSLVTSDGCCLVHQG

>Neuroparsin_Brachiopoda_Laqueus_californicus_comp703293_c0_seq1.p1

NPDQIPEGRCRKDPKMIGVEGAPPYCQHKCTPKYCRDHPKAICASFDIADEKRSCQGACQHTSCSACRYVND

>Neuroparsin_Brachiopoda_Glottidia_pyramidata_comp8764_c0_seq1.p2

SSSSSSSSTDLKCPSCDQLHCSPRRASKLYTKCKGGITSGICNCCPVCAKVEGEDCGGEWNYLGKCDKGLYCKPRSLQHYDDQVAFPGYAG

>Neuroparsin_Brachiopoda_Glottidia_pyramidata_comp36300_c0_seq1.p2

EKENRPPYVRIEGYNCQPKCTPDFCRDNPKAICAAFDVAEVKQSCQGECQHTSCMACRFVNDEPACGKCAKDDFGCIKKFAKCYKRHTCTRNKFPCKKKFKENGKFMCAVPACLD*

>Neuroparsin_Brachiopoda_Novocrania_anomala_comp979053_c0_seq1.p1

SGNNKIPEGICRKRWEMKVGDEGLPEITCRPKCTPTFCKENLREICAASDVAEVKQSCQGDCQHPSCRACR

>Neuroparsin_Brachiopoda_Novocrania_anomala_Nano.rna.tri2.26078.1.p1

MHLPPADIVVCFGILTLALVSPSTTKEHHQGKNLSSTSELQCPPCDRIHCPVRRPSKLKCKGGITKGVCNCCYKCAMLEGEKCGGEWNYLGKCDAGLYCEPRKIQPDDSRYRHSSGNNKIPEGICRKRWEMKVGDEGLPEITCRPKCTPTFCKENLREICAASDVAEVKQSCQGDCQHTSCSACRFVNDDPDCGKCRKDDFQCMRKFAKCFKKQICSKQKLPCRKTTMKEQGKFLCVVPKCMD

>Neuroparsin_Brachiopoda_Terebratalia_transversa_Ttra.rna.tri.5565.1.p1

MMMRLNTHVLVVLCGLVVLAISTHAYKNKHLRGSGATLKNKNANKVNNYGDAMHCPPCDQISCPTPRRVSKLRKQCLGGITTSVCDCCKVCAKIEGQICGGEYNYLGKCDKRLYCHPNPKQPNPNQIPEGRCRKDPKMIGFEGAPMYCEHKCTPKYCKDHPKAICASFDIADEKRTCQGACQHTSCSACRYINDQPDCPKCSKDDFPCMRKFAKCIKKQVCSRNKFPCNRKSGRKTKITEQGKFTCTVPKCLNS

>Neuroparsin_Brachiopoda_Lingula_anatina_comp148442_c0_seq1.p1

MWLSRDVLIAFFWLVNSCFLSHTTTFAKEHRERGSADRSQSHKKKELSTVSSSSSSSSSSMDLKCPSCDQIHCSPRRASKLYQKCKGGVTLGVCDCCPICAKVEGEDCGGEWNYLGKCDNGLYCKPRSLQHYDDQVAFPGYSGNKMNKIPEGKCTKRPGMVGIQGVPGYCHPKCTPDFCRDNPKAICAAFDVAEVKQSCQGECQHTSCMACRFVNDEPDCGKCAKDDFGCIKKFAKCYKRHTCTRNKFPCKKKFKENGKFMCAVPACLD

>Neuroparsin_Brachiopoda_Hemithris_psittacea_comp26368_c0_seq1.p1

MLVKYYIKLVVLCGFFASVLSVRNHRGRLRHGKSHKSNNNNSDNLSPVKCPPCEQISCPKPRRVSKLKKICKGGITTGVCNCCVVCAKILGEPCGGEYNYLGKCDKGLFCHSKPRMKKVQDDIPEGKCRKDPKAFRDEGSPNFCRPKCSPKYCKDHPKAICAAFDVAEQTRSCQGDCQHTSCSACRFVNEQADCGKCAKDDFGCMKRFAKCIKKQVCTRNKFPCRTKKKKV

>Neuroparsin_Nemertea_Lineus_longissimus_c55668_g1_i1_m.203387

SETNTDLICPRCQEERCRPSRKQRLNCRGGYTLDVCNCCPTCAKLEGDLCGGDYQMFGKCDRGMVCQPNSVDANRYGVYKNPVGRCERVIGTSLLQADQPKGLHCK

>Neuroparsin_Nemertea_Riseriellus_occultus_TRINITY_DN66292_c0_g1_i1.p1

ECRPINDPCAKVECEEIDPDLKCPADSFKLPSIPSADGCCLVQQGCTCRPQTDCTAPSCPEGLKVQIVEAASEIPG

>Neuroparsin_Nemertea_Lineus_lacteus_comp33643_c0_seq1_m.254380

MDCQINGFLVLVFLCCLHGDAVHGQLVCNDCDTTLCEKPVDCPGDIILDQCRCCHVCARMENDTCGGPFDAFGRCAEGLV

>Neuroparsin_Nemertea_Nipponemertes_spec_TRINITY_DN46499_c0_g1_i1.p2

SICSLRHLPPQCGPSEELSLVKPAVDEPGKCCDIFECVEKAPSTTTLAATKNCSSVTCATKVDCPPDSRPLPSIPSETSCCPASPRCACLESPCPRAECAPGLEVKVVTPGRGAP

>Neuroparsin_Nemertea_Cerebratulus_marginatus_comp1998225_c0_seq1.p1

LEAASEQPGKCCDTYQCYNGSMLMCDYQGMKLFDGEVVRVDKCTKCVCTNGLSLCEVQTCKDIPSCSWMETPKGQCCPVCLG*

>Neuroparsin_Nemertea_Cerebratulus_marginatus_comp1393766_c0_seq1.p1

KRVVIKKGTGKPGSCCDVYECRPINDPCAKVECEDPDPSFVCPSDSFKLPSIPSEDGCCLVQQGCTCRPQSDCPTPTCPEGLKVQILEAASEQ

>Neuroparsin_Nemertea_Cerebratulus_marginatus_comp34854_c0_seq1.p1

RCLEAVRYTSLQADAPLGLHCKPRCTIEYCSKNPRAICAAIENAEISQPCQGDCQHTSCKACRFISRQPDCPKCANDDFSCMRTFAKCIKKHLCKRTKGLSCKRIESSKSNGHFQCFVPECL*

>Neuroparsin_Nemertea_Notospermus_geniculatus_g3781.t1

MDPCRIYGFLGLIFLCLVRVDVVFGLICDDCDPTQCQKPVDCPGELILDQCRCCHVCGRVENETCGGPFDAFGRCGVDLVCFTSLQSPDTLVGVCKPKDFRPDVNRTAELSNILEEEKEVDPCADVRCQVVFAPKCPSDSKATGGNILPGECCPSAAKCVCNYMECLNPVCSPGYEKIVTKKGTGLPGSCCDIYECRTINDPCEDVECEETDPDLKCPQDSFKLPGIPSADGCCLVQQG

>Neuroparsin_Nemertea_Notospermus_geniculatus_g9726.t1

MLLSYLITFPTVLLQGIVLVSSTQVATKREDEHQHSNGNHGDLICPRCQEERCRPVRKERLNCRGGYTFDVCRCCPTCAKVEDELCGGDYHMFGKCDRGLGCVANGQDARRYGVYKTQVGRCQRGTYSSMQAGQPIGLHCKPKCTRKYCAKNPKAICAAIDNAEKSQRCQGDCQHTSCKACRLVSRQSDCPKCSNDDFSCMRNFAKCIKKHMCKRSKGLPCKRWESAAGDADFRCFVPECL

>Neuroparsin_Nemertea_Baseodiscus_unicolor_Contig33909_m.268943

MDSMRTNRFLLVSLFCIFLCGIKGGLSLVCDNCDKEACDAPTNCAGGLVYDLCHCCSACAQVENQTCGGPFETLGKCDEGLICFGVDPENTFSADIGVCKPKTWNLEDIE

>Neuroparsin_Nemertea_Baseodiscus_unicolor_TRINITY_DN99906_c1_g1_i3.p1

KPKTWKSDFASTPRTSTNIADVEDKCLNVRCEVVFAPRCPPDSMPVGGSISPGDCCPTAATCVCNYLECLNPVCSAGYDKVRTKKGTGEPGECCDIYECIPINDSCANVECNDPRPDIVCPSDSVRLPSIPSADGCCLERQGCTCRPKSDCPSPSCPEGLKVKVTEPALGQPAFCCDKYDCVDETLMMCSYDGLDFHEGESIKVDECTTCECRDGLSFCKKEECVDIPSCSWTTIPHGECCPVCLGCLSNTGNAYKNGEVWTEGDDCIHCRCDQGKVYCQA

>Neuroparsin_Nemertea_Lineus_longissimus_c50171

MDCQIYGFLVFVFLCGLHGGAVRGQLVCNDCDTTLCEKPVDCPGDIILDQCRCCHVCARMENDTCGGPFDAFGRCADGLVCFGSIQSLDTLFGVCKPKDFKPENRTDVISNILEEEIDPCADIRCQVVFAPRCPSDSKAIGGNILPGECCPSAAKCVCNYLECLNPVCSAGYNKVVTSRNTGVPGRCCDIYECRPINDPCAKVECEEIDQNLKCPADSFKLPSIPSADGCCLVQQGCTCRPKTACTAPSCPEGLKVQILEAASEKPGKCCDTFKCYNGSMKMCDYLGIKLLDGESITVDKCTKCRCENGLSMCQTETCNDIPLCGWLETPAGECCPVCQGCLASSGKAYKNGEQWNEGNDCVHCHCVDGEVYCQAEMCAVHCSKPRKIPGQCCQVCDDPEVVTVAPNCPRMDNCTRLCAAGWDKDDRGCYICSCRPRMYNSILC

## NKY

>NKY_Phoronida_Phoronopsis_harmeri_c13296_g1_i1.p2

LRALSKLYDTYLNVLLANENYVESDDNKSNDYEEYVNEVLQRVPVSLTKVKVDALPPSDLSERKRNARHMVLKKNSFWQSIGGPLSVETKFGSRGGGRDTASVGSKVIRWGRR

>NKY_Phoronida_Phoronis_ijimai_(P._vancouverensis)_comp85680_c0_seq1.p1

MTSILISLTLAIVSLRLSQCLNPAENLSSASLKSDRGVIRRLRVLLNRLESKERTLFDYIPRISGDNAFTESDYAEYLKEVMERVPPALERVKIDTQPYSAGGDGMEQQMLEKKSFWQNLGGGPLSIETKFGSRGGGGDSSSLTGKKPMRYGK

>NKY_Phoronida_Phoronis_australis_TRINITY_DN291734_c0_g1_i1.p1

MTSLLIPLAIAIISMRLTQCLHPAENSPSASQKSDSGVMRRLLLDRLESKDRTLLNKISSTNNENAFLESNHAERLKELLDRVPPALERVKIDRTQPNSAGAGSDGIGHTVLEKKFWSSLGGGPLSAETKFGPIELEHAKSNAGNQIFRYGK*

>NKY_Phoronida_Phoronis_australis_TRINITY_DN291734_c0_g1_i2.p2

MTSVLIPLAIAIISMPLTQCLHPAENSPPASQKSNRDVMRRLISDRLESNLWTLFDKISDTNNDNAFSESDNAEYLKEVLDRVPPALERVKIDRTQPYSAGAGSDRMGHKVLEKKSLWHIIGGGPLPIETKFGSRGGEDFISSKGRPTKLLRYGK

>NKY_Phoronida_Phoronis_psammophila_comp66427_c0_seq1.p1

MLQQRFKMMSILTTAIVFISSMRHVCGLSLGGVTLDNANDRRQQNERLLSSLVTRLEQEQLEDLIGYRDTNEALTENEQYLNELLERVPPSLTRVQVYSPPIVRDAKRDADRMLRQKQSFWQSLGGPLSVETKFGSRGGGKGSSFRPLKVVRYGK*

>NKY_Brachiopoda_Novocrania_anomala_Nano.rna.tri2.138125.1.p1

MKLKMKDIMCVIVIMYSVSLSETRTIWDDIHTEEKTHTRERNVKQITNSEHVSQMLKALYAAKHPDNRGGAEGREIVLTLADLLKKADRITKSLRADLKRKNAIILEYKKQHGDIPLNTGLNRRLVQVFKKPSFWNPMGQLPPVRVRLGLGASATTDAVEGKGSEILRYGR

>NKY_Nemertea_Cephalothrix_hongkongiensis_comp36790_c0_seq6_m.45454

SSEENFQKFLARKKRSVSRHSELELLKDIVSKMQHILEAEDEDDQERRKKSNYWPSMGILPVETRLSSFGSQIGGTSNGIGGHKPFRYGRK

>NKY_Nemertea_Lineus_longissimus_c38886_g1_i1_m.49687

MNKYICGCAFLVVLSICYVNSENVDDLHKFLIRRRRSAHNGLRQESELKNLVRELMDKLQNIIDTDRALSSALEEESNEVRHKKTGFWPSMGPLPVETRLSSFGSQIGGATNEQASHKPFRYGRK

>NKY_Nemertea_Paranemertes_peregrina_comp535798_c0_seq1.p1

DRLQRDAELKELVHDLIEKLDDMIEADHALEALVKSGGPIKRTGFWPSIGPLPVETRLSSFGSQIGSGSGSRNKQKTFRYGK*

>NKY_Nemertea_Baseodiscus_unicolor_TRINITY_DN101264_c0_g2_i3_m.46284

ALSTALENDIANNEIRHKKTAFWQSMGPLPVESRLSSFGSQIGGGNNDHASHKPFRYGK

>NKY_Nemertea_Baseodiscus_unicolor_TRINITY_DN10499_c0_g1_i1.p1

MKTLYGCAFLVLLAVDQASCERSKQAVRSYTARQGRSIPEHKLLVSKLITKRENVMGRDKALSSTLEKDVINNKIQYKRIAGVKPTSSPRLFIGK*

>NKY_Nemertea_Tubulanus_polymorphus_comp32293_c0_seq1_m.30020

MKTSSQAVLLVVLIAVVYVTSEKAGDAMAKRWIQRRRRSTTAGEVEALRDIVSKIQNILEEYDDETTSRRKKNSFWPSMGPLPVSTRLSSFGSQIGGEGGHVRVAQKPFRYGK

>NKY_Nemertea_Cerebratulus_spec_TRINITY_DN37986_c0_g1_i1_m.7615

MNKYICGCAFLVVLSICYVSSENADDLHKYLIRRRRSAHNGLRQDAELKNLVRELMDKLQNIIDTDRALSTALEEESNEIRHKKTGFWPSMGPLPVETRLSSFGSQIGGANNDQASHKPFRYGRK

>NKY_Nemertea_Lineus_lacteus_comp25455_c0_seq1_m.88512

MNKYICGCAFLVVLSICYVSSENVDDLHKFLIRRRRSAHNGLRQDSELKNLVRELMDKLQNIIDTDRALSSALEEESNEVRHKKTGFWPSMGPLPVETRLSSFGSQIGGATNEQASHKPFRYGRK

>NKY_Nemertea_Lineus_ruber_Lvir.rna.tri.11786.1_m.25141

MNKYICGCAFLVVLSICYVSSENVDDLHKFLIRRRRSAHNGLRQETELKNLVRELMDKLQNIIDTDRALSSALEDESNEVRNKKTGFWPSMGPLPVETRLSSFGSQIGGANNEQASHKPFRYGRK

## NKY-2

>NKY2_Nemertea_Notospermus_geniculatus_g14521.t1

MAKEMAWWKQTAILLTVASLCSLLPSIVDAKDSAELPDAVKNDNIVAYLSHFAKPNRRQMIDPYGMQKMKKRNDDSVWLWVPSQGYVSVPVDEAGSSGANSGKLMRYGRR

>NKY2_Nemertea_Tubulanus_polymorphus_comp34212_c0_seq1_m.39371

MKTSSRYQFMGLAFLILLVVNTIADSDIEQTEKFDPTIARLLQMAGRPHRSDNSLADVKANRPMKRGNTAWIWVPGQGFAEVPAELAAEVAQGITDERVANKLMRYGRRR

>NKY2_Nemertea_Baseodiscus_unicolor_TRINITY_DN94194_c0_g3_i1_m.184146

MRSPAMTWWKHLIVLVTMSSLVLLLPSVSEAKTSDGADDSFKNDNLVSYLSRIAKSNRGQMTDPLYLSQPSKRSNQGVWVWLPSEGYVSVPMEEASNGGAGSNGKLMRYGRR

>NKY2_Nemertea_Lineus_lacteus_comp18240_c0_seq1_m.31192

MAQDMAWWKQAAILMTVASLCSLLPSIAEAKNSDEIPDSIKNDNLVAYLSQIAKSNRRQMIDPLGPYGMRKMKKRNDDSVWLWVPSQGYVSVPVDEAGSSGANNGKLMRYGRR

>NKY2_Nemertea_Lineus_longissimus_c35966_g1_i1_m.36171

MAQEMAWWKQAAILMTVASLCSLLPSIAEAKNSDEIPDSIKNDNLVAYLSQIAKSNRRQMIEPLGPYGMRKMKKRNDDSVWLWVPSQGYVSVPVDEAGSSGANNGKLMRYGRR

>NKY2_Nemertea_Lineus_ruber_Lvir.rna.tri.33795.1_m.65390

MAQEMAWWKQAAILMTVVSLCSLLSSIADAKNSDEIPDSIKNDNLVAYLSQIAKSNRRQMIDPMGPYGMRKMKKRNDDSVWLWVPSQGYVSVPVDEAGSSGANNGKLMRYGRR

>NKY2_Nemertea_Cerebratulus_spec_TRINITY_DN35340_c2_g1_i2_m.177379

MTMARDMAWWKQAAILMTVASLCSLLPSIADAKNSDEVSDSIKNDNLVAYLSQIAKSNRRQMIDPVGPYGMRKMKKRQDDSVWLWVPNQGYVNVPVEAGSSGANNGKLMRYGRR

>NKY2_Nemertea_Nipponemertes_spec_TRINITY_DN46647_c0_g1_i1.p2

MDQGLSSLRQVLFMCLALSVVVATLATPPDLAPGLAPGRKAALMDYLASVMERAARSGQVHYQKKAPHKRDEGNWIWMPGQGYVPVPEGSMPGAAAGANGKLMRYG*

## NpY/NpF

>NpYF_Phoronida_Phoronopsis_harmeri_c93241_g2_i1.p2

FVFDVCGQDRMPSPPRRPKAFANTAELRSYLKSLSNYYSIIGRPRFGKRRQLEPSLWHAVRADPSYQDSFPSYFDY

>NpYF_Phoronida_Phoronis_psammophila_comp66213_c0_seq2.p1

MRTVLVLSAFTLVAMLLVREVCCDDRMPSPPQRPKQFSSTAELKSYLKSLSNYYSILGRPRFGKRSRSYRPENSLWQVLRPNDDIYQDVIPLYGYDY

>NpYF_Phoronida_Phoronis_australis_TRINITY_DN321228_c0_g11_i1.p1

MRTYITLSILTVALTTLVYQVSCGDQMPSPPRRPTVFSSTAELKKYLKSLSNYYSILGRPRFGKRNQHQKSEMSLLQLLSPDDVLDEEYVPLYRMY

>NpYF_Phoronida_Phoronis_ijimai_(P._vancouverensis)_comp91706_c0_seq1.p1

MRTYITLSVLTVAFTTLVYQVCCDDRMPSPPRRPTVFSSTAELKKYLKSLSNYYSILGRPRFGKRNQVHKSEVSLLQLLSPDDVLDEEYVPVYRVY

>NpYF_Brachiopoda_Novocrania_anomala_Nano.rna.tri2.33928.1.p1

MNLEMNLCRYVHSAGNLLWLSVFVLAILSTVCRSEPIPGPPHRPARFSNAIELRRYLSKLNDYYAVVGRPRFGKRSSVIYTPRYYPRWPETLDIDGDGKVSSAELNYYAQHLQHMLDFRK

>NpYF_Brachiopoda_Terebratalia_transversa_Ttra.rna.tri.456.1.p2

NDRTNGQAKPFPPRPTLPEQFSNADEVREYMRQLASYYAQVGRPLYGKRGYWNENIPNYYIKHKEYKDMS

>NpYF_Brachiopoda_Laqueus_californicus_comp31889_c0_seq1.p2

MMHLLFSKHELCSTSRKVTTMTMLVLFLTLHHSTCANQQRRPPIRPKEFESDSQVQKYMAAISEYYAVIGRPRFGKRWTGSSNQNQRISDNPGILKDMINHLVTRHVKSNNVESGQLIVVVGGR

>NpYF_Brachiopoda_Hemithris_psittacea_comp12060_c0_seq1.p2

MSCRATTNTCACIWIGIVLLVQVCAAGSKLRPPARPARFESAAQVRRYLEKVREYYGVISRARFGRDVGNLHRHF

>NpYF_Brachiopoda_Hemithris_psittacea_comp22948_c0_seq1.p1

MNQHKHYITASLLILATVLASVTNASVPEPPTRPKFFSSHTQLWNYMTELSEYYAIIGRPRFGKRLSPVGIGTLNRKNVFTDRNIGNLRSENMLLSLKLVRDLKTTEKDLSLLLYHLLVKKDSKKTYQSSENLRVF

>NpYF_Brachiopoda_Glottidia_pyramidata_comp35872_c0_seq1.p1

MRSQKDGQSRTCHTSHRSNRLTLTSLAIVLILLTQVLTPCVADHLTKPPARPTSFKNKGELADFLRKLNAYYDIVGRPRFGKRALGSLDNGGFYDDALNNVRSSVLDMLDFNGDGKISSAELSMAYYMDGMAPQKRRR

>NpYF_Brachiopoda_Lingula_anatina_comp119695_c0_seq2.p1

MRSINTNPSSSKLTLSCIAIALIILTQVLAPCFAHQLTKPPTRPSSFKNKAELTEYLRRLNAYYDVVGRPRFGKRAMGPSEYGGGLDDAVNNVRSSVIDLLDFNGDGKLSSAELGLAYYLDEIAPQKKRR

>NpYF_Brachiopoda_Lingula_anatina_comp146792_c4_seq2.p2 (no poly-proline N-terminus)

MVTSKRSLIVAMILMLAVAQMTDVADASSPRTLGQLKSLLRRAYAIIEVQSRPKFGKRGILPSPVTGYGDSVCLDKKAPVALEGDGLEDAI

>NpYF_Brachiopoda_Lingula_anatina_comp118535_c0_seq1.p1 (no poly-proline N-terminus)

MASSKWCLTVALLFLLTVTQVTLAEGDKQVRTLGQLKGALRRAYAIIEMQSRPKFGKRKISSYPIADADADDSLCLGVDKKAIARGALGEANLEEDM*

>NpYF_Nemertea_Cerebratulus_marginatus_comp16956_c0_seq1_m.8058

VYGSPDGIPGPTRRPPVFSSPEELKEYLQALNEYFAIVGRPRFGRSVSKRSAFRRSNAEESLFDAPYGDNAMSADDYESEDYIPSYIRYLRRR

>NpYF_Nemertea_Tubulanus_polymorphus_comp34940_c0_seq1_m.43503

MNKFLVALFALFVSLGITVYGHNLPGPPERPAVFTSPEALRNYLRQLNEYFAVVGRPRFGRSIHKINPVKRERRSSSIYE

>NpYF_Nemertea_Cephalothrix_hongkongiensis_comp22430_c0_seq1_m.12888

MNKYLVCFGVLLLSIAIVLVSGHDGLPGPPERPTVFRSPEALRDYLRQLNEYFAVVGRPRFGRSINKRQAAFKREINPNVFE

>NpYF_Nemertea_Cerebratulus_spec_TRINITY_DN30327_c0_g1_i2_m.166155

MDKFIIGFFCVVFVALIADVYGSPDGIPGPPSRPRVFSSPEELKDYLQALNEYFAIVGRPRFGRSVNKRSAFRRSNADESLFE

>NpYF_Nemertea_Lineus_lacteus_comp24893_c0_seq2_m.78466

MDKFIIGFFCVIFVALIADVYGSPDGIPGPPRRPAVFTSPDELKDYLQALNEYFAIVGRPRFGRSVNKRSAYRRTSAGESIFE

>NpYF_Nemertea_Lineus_longissimus_c36500_g1_i1_m.38225

MDKFIIGFFCVIFVALIADVYGSPDGIPGPPRRPAVFTSPDELKDYLQALNEYFAIVGRPRFGRSVNKRSTYRRNNAGESIFE

>NpYF_Nemertea_Lineus_ruber_Lvir.rna.tri.5013.1_m.11407

MDKFIVGFFCVIFVALIADVYGSPDGIPGPPRRPAVFTSPDELKDYLQALNEYFAIVGRPRFGRSVNKRSAFRRNNAEESIFE

>NpYF_Nemertea_Riseriellus_occultus_Contig13590_m.140268

MGKFIIGFFCVIFVALIADVYGSPDGIPGPPRRPAVFTSPDELKDYLQALNEYFAIVGRPRFGRSVNKRSAFRRHNAEESIFE

## PDF/Cerebrin

>PDF_Cerebrin_Phoronida_Phoronis_australis_TRINITY_DN278513_c0_g1_i1.p1

MACFKRNGFVTACSLILTLFVIVRGGPVPQSYEKVLAPSYGNLRKGLLLGLLKTKIPTAIYLYDTDERRNYVQQPRSAPSFLKDYPIVISDGHDIDESELQANKMNMASNGGATIDDETNGYKYMVNRLDNLHSLIHDPEYILQGMEEKRNAGTRDMMYNLPDFHKIGK

>PDF_Cerebrin_Phoronida_Phoronis_ijimai_(P._vancouverensis)_comp421263_c0_seq1.p1

MAPFNINTFVLACTFLPTLSVIVYGGPVPQGYEKELAPSYATLRKDLLLDLLESKIPAVEDLRDVDEIRNYVSQARSVPGFLADDTVIISRDHDIDESEFQADELNMANEGRTSIDDETNAYLAQSLDYLHSLMHETKARIQAMKNKRNAGIRDMLYNLPDFTKIGK*

>PDF_Cerebrin_Brachiopoda_Novocrania_anomala_Nano.rna.tri2.80018.1.p1

MKISHHAVTVMLLVWITLISTVFSSYQHHKFIQRGGSETSKDEIVDLAFRIMRIALNHQRRAKTYITSAKRNQWTIDSLINLPDLSMIGKKR

>PDF_Cerebrin_Brachiopoda_Glottidia_pyramidata_comp429590_c0_seq1.p1

MRPGRCQPSYSLLAVLFILLLSVLTRAAEYQQFGNNKYMEDKDANEISSLAFKIMRLALKPHPDRLIQRWNTKRNQWTVDSLYNLPDLTAVGRRRRR

>PDF_Cerebrin_Brachiopoda_Lingula_anatina_comp133642_c1_seq2.p2

MRSRRCQPSSYGVFIALLIVLLSVLITSATDFQQLDNTNYLADKDANDISTLAFKILRLALKPQRDRTLMRNTKRNQWTVDSLYNLPDLTAVGRRRRR

>PDF_Cerebrin_Nemertea_Cerebratulus_marginatus_comp46573_c0_seq3_m.33337

MVFFVAFSLVAASPARSFNSIDEKERQEIMASAAKILRIALAGSEKMAYKRGTGVIDSLFNLPSNLEAVGRK

>PDF_Cerebrin_Nemertea_Paranemertes_peregrina_comp22303_c0_seq1_m.21110

MKYFAFFFFFLVLVSATYSAPTNSRELSEKTRQEIALNAGRILRLVMDQQRGAEKRGGVVDSLWTLPDLARVGKK

>PDF_Cerebrin_Nemertea_Lineus_ruber_Lvir.rna.tri.20471.1_m.41792

MNTTCLVAILLVVFSLVAASPARSNSIEDKERQEIMASAAKILRIALASSEKPYKRGAGVVDSLWNLPNNLEAVGRK

>PDF_Cerebrin_Nemertea_Malacobdella_grossa_comp20431_c0_seq1.p1

MKYSAVLAIFVMLIAATLSAPTNTLELSGKDRQEIALSAARILRLVTSYSGNKAEKRRGGVVDSLWTLPDLGRIGR*

>PDF_Cerebrin_Nemertea_Tubulanus_polymorphus_comp32296_c0_seq1_m.30032

MKHKCGLVLLGIVAVAMVMMPAYAAPSRSVDDIEKQNILNSASRIIKIIMASDSVNKRNSGTIDSLWNLQDFNRVGRR

>PDF_Cerebrin_Nemertea_Notospermus_geniculatus_g489.t1

MRTTCLIAVFLIVFSLVAASPARSPNSIADKERQEIMASAAKILRLALAGSDRVPYKRGAGVVDSLFMLPENLEQVGRK

>PDF_Cerebrin_Nemertea_Cerebratulus_spec_TRINITY_DN27352_c0_g1_i1_m.166848

MRTVCLVAILLVVFSLVAASPARSINSIDDKERQEIMNSAAKILRIALAGSEKLAYKRGAGVVDSLWNLPSNLEAVGKRK

>PDF_Cerebrin_Nemertea_Lineus_lacteus_comp20382_c0_seq2.p1

MNSTCLVAILLVVFSLVAASPTRSNSIDDKERQEIMTSAAKILRIALAGAEKLPYKRGAGVVDSLWNLPNNLEAVGRK*

>PDF_Cerebrin_Nemertea_Lineus_longissimus_c32437_g1_i2.p1

MNTTCVVAILLVAFSLVAASPARSNSIADKERQEIMASAAKILRIVLASSEKLPYKRGAGVVDSLWTLPNNLEAVGRK*

## PedalPeptide-1

>PedalPep1_Phoronida_Phoronis_psammophila.20561 Phoronida

MKSLVASVLILLCALCSYALPIDDKIEDDGQKLNNVEKMTVMSKVLEQLRKTENDSDRRRQLDSLGGANIHGRRQLDSLGGANIHGRQLDSLGGANIHGRQLDSLGGANIHGRQLDSLGGANIHGRQLDSLGGVNIHGRQLDSLGGVNIHGRQLDSLGGVNIHGRQLDSLGGANIHGRRQLDSLGGANIHGRRQLDSLGGVNIHGRQLDSLGGVNIHGRQLDSLGGANIHGRQLDSLGGANIHGRRQLDSLGGANIHGRRQLDSLGGVNVHGRQLDSLGGANIHGRRQLDSFDPPNVASFDGAKIQEGEWDDGKVDFDDEDEKRGFDRFSQGSFDSFAKRPFNRFSQGGFDSFAKRPFNRFSQGNMDMFAKRPFDRLGTGSFDTFAKRPYDSFDTDRTDSFAKRPFGRIGQGSMDTFAKRPFDRLSSSSFNSFYKRSGDEEK

>PedalPep1_Phoronida_Phoronopsis_harmeri_c118833_g1_i3.p1

GSMDSFAKRPFDRLGHGSMDSFAKRPFDRFSQGSIDSFAKRPFDRLSHGGMDSFAKRDATEQQ*

>PedalPep1_Phoronida_Phoronis_australis_TRINITY_DN246374_c0_g1_i1.p1

DRIGHGFGSLAKRPFDRIGHGFDSFGKRPFDRIGHGFDSFGKRPFDRIGHGFDSFGKRPFDRIGHGFDSFGKRAVESGVSGERK*

>PedalPep1_Phoronida_Phoronis_australis_TRINITY_DN261857_c0_g19_i1.p1

MRLWEVCAFVVVMVACAKALPVKDDGEKEAEQGKLNELEKLTVMTKVLEQLSDNENDSDRRRQLDDLGGGNIHLRQLDDLGAGNIHGRQLDSLGGGNIHGRRQLDDLGGGNIHGRQLDSLGGGNIHGRRQLDDLG

>PedalPep1_Phoronida_Phoronis_australis_TRINITY_DN261857_c1_g2_i1.p1

AEQGKLNELEKLTVMTKVLEQLSDNENDSDRRRQLDDLGGGNIHLRQLDDLGAGNIHGRQLDSLGGGNIHGRRQLDDLGGGNIHGRQLDSLGGGNIHGRRQLDNLGAGIIHGRELDEADGQRQQLDGDENDRYLDDFQKRPFDRIGHGFDSFGKRPFDRIGHGFDSFGKRPFDRIGHGFGSLAKRPFDRIGHGFDSFGKRPFDRIGHGFDSFGKRPFDRIGHGFDSFGKRPFDRIGHG

>PedalPep1_Phoronida_Phoronis_ijimai_(P._vancouverensis).35823 Phoronida

MRLWEACAFVVVMVACAKALPVKDNGENQGEKGKLNELEKMSVMTKVLEQLSDNENDSDRRRQLDNLGGGNIHLRQLDNLGGGNIHLRQLDSLGGANVHGRRQLDNLGAGIIHGRQLDDLGGANIHGRRQLDNLGAGIIHGRQLDDLGGATIHGRRQLDSLGAGIIHGRELGEADAQRRQLDNLGGGNIFLRQLDSLGGGSIHGRRQLDDLGGANIHGRDIGYDKNDLTGVEEKKAFDRISHGSLASLSKRPWDRIGSGSLNDFKKRPFDRISHGFDSFAKRPFDRISHGFDSFAKRPFDRISHGFDSFAKRPFDRISHGFGSFAKRPFDRIDHGFASFAKRAAESGVPGDKK

>PedalPep1_Phoronida_Phoronis_ijimai_(P._vancouverensis)_comp94075_c0_seq1.p1

MRLWEACAFVVVMVACAKALPVKDNGENQGEKGKLNELEKMSVMTKVLEQLSDNENDSDRRRQLDNLGGGNIHLRQLDNLGGGNIHLRQLDSLGGANVHGRRQLDNLGAGIIHGRQLDDLGGATIHGRRQLDSLGAGIIHGRELGEADAQRRQLDNLGGGNIFLRQLDSLGGGSIHGRRQLDDLGGANIHGRDIGYDKNDLTGVEEKKAFDRISHGSLASLSKRPWDRIGSGSLNDFKKRPFDRISHGFDSFAKRPFDRISHGF

>PedalPep1_Brachiopoda_Terebratalia_transversa_Ttra.rna.tri.13739.1.p2

FAKKSFDKVGYGGLSNFAKKSFDKIGYGYLSNFGKKPFDAREYRVFSHTIGKEGQLRPDKKT*

>PedalPep1_Brachiopoda_Laqueus_californicus_comp33163_c0_seq1.p1

MLHSMRTFVILVTLYYCSAIALRRSSVEKEISSPQEEQARDEKVRQFDSLGGAQVHGINGRDVNELRDYIVQYLSNFDKRSFDTIGYGGLSNFAKRPFDAIGYGGLSNFAKKSFDRIGHGGLSNFANK*

>PedalPep1_Brachiopoda_Glottidia_pyramidata_comp41013_c0_seq1.p1

MNFISGTVIFGILVAFTVKADIKAADDDSEALAPYPRQLDNLGSGFVHGMYGRQLPGADDSVKYEEADDLSPMDKRPFDRISGGNMASFAKRPFDRIAQGSMSSFVKRPFDRISNGKMASFAKRPFDKIAGGRMSSFAKRPFDKIAGGRMSSFAKRPFDRISSGRMSSFAKRPFDRIGVGRMASFVKRDSGSKDASQKENTSA*

>PedalPep1_Brachiopoda_Lingula_anatina_comp146495_c1_seq1.p1

MKFATGYVTFGMMVVLALADVKSGDDAGSDDVSRELDTLGQGYAPSMYGRQLDNLGQGFVHSMYGRESEDGDEANAEPVDEAEEWGNVAKRPFDKIGSGRMASFVKRPFDRIQSGRMSSFVKRPFDRINAGRLASFVKRPFDKIGSGRLASFVKRPFDRIGAGRIASFAKRPFDRIGAGHMSSFVKRPFDRIQSGAMASFVKRETSKEISQKETAKA*

>PedalPep1_Brachiopoda_Hemithris_psittacea_comp20155_c0_seq1.p1

MEFHAHIKAVLLLTLVFFCHGFPTSEKAKKDKRSLSDEEINKLTKELRALRIALNQVDPERDESRRQFDSIGAGYVHGLHGRQLDNPLESKRYVDSINNGRLSSFYKRNFDSLNNGGRFHTFYKKSFDSIGNGGLHGFVKKRSFDRINNGGMSRFIKKRFDTIGSSGKLHSFFKKSLQSIGNGRFDKLDKKSFDAIGNGRLNNFHKKNQ*

>PedalPep1_Brachiopoda_Novocrania_anomala_comp18130_c0_seq1.p1

AITAGSFNKRRLDSIAYKGLSGFAKRPADDDSEVSKRRLDSIAHGSDFSNFAKRKFDSIGYGNFQGFAKRDDDNSAEPSKRRLDSIAHGAFSSFAKRPLDSIAHGGAFHGFKRRFDSISHAGNFGSFAKRRLDSIAHGGFSSFDKKEKS

>PedalPep1_Brachiopoda_Novocrania_anomala_Nano.rna.tri2.22344.1.p1

MTNVTHVLALCAAVLVMVVNALPMAEDMANEVRVVPGKHDVTIEKAIDDALNDIENSDQLNGSEKNLDLSKEDDERNLDTLGGGNVHGLYGRQLDALGGGSVHGLYGRQLDSLGKGAVHGFYGRDLDEKEKRRLDSIAFNSGMNGFQKKAFDSLAYNSGMNNFHKRRLDSIAYKGLSGFAKRPADDDSEVSKRRLDSIAHGSDFSNFAKRKFDSIGYGNFQGFAKRDDDNSAEPSKRRLDSIAHGAFSSFAKRPLDSIAHGGAFHGFKRRFDSISHAGNFGSFAKRRLDSIAHGGFSSFDKKALDSIAHGGDFNSFAKRPAYEKRRMDSIAHGGMAGFEKRDSS*

>PedalPep1_Nemertea_Cephalothrix_hongkongiensis_comp39437_c1_seq9.p1

ELRLKDLMVGHLERISSMTPRRIQERKFLEIRLEAEEICLGLYLPGSRLPFITRNRTTVKRDLSDVSAERQDRYLDRLGGGNVHMSGRQLDTLGNAAVHGGRRYLDNLGGANVHQSGRDLENEKRRFDSIGGNDFSSFNKRRFDSIGGGDGFSSFNKRRFDSIGGNGGFSSFNKKKRRFDSIGGGDGFSSFNKRRFDSIGNGGFSAF

>PedalPep1_Nemertea_Baseodiscus_unicolor_TRINITY_DN103934_c0_g6_i6_m.145312 3prime_partial

MRRRETHTVLCTAAYSTPLTEFFLPESCNVFLPSVANMRRCTVLCVFLFVAWTCATPASEHENQEVKKRSVESDVIEQFIDSSKHKSNEADDEETGEGRRHLDTLGGAVIHPVRELIDSLGGGEDHPYRRELFDTLKGGQVHPLRREYLDTLGGAVIHPVRD

>PedalPep1_Nemertea_Baseodiscus_unicolor_TRINITY_DN103934_c0_g6_i6_m.145311 complete

MRRRETHTVLCTAAYSQHLEGKRYRIREERIQSKESNMRRCTVLCVFLFVAWTCATPASEHENQEVKKRSVESDVIEQFIDSSKHKSNEADDEETGEGRRHLDTLGGAVIHPVRELIDSLGGGEDHPYRRELFDTLKGGQVHPLRREYLDTLGGAVIHPVRDETGAMEKKPFDPISHCH*

>PedalPep1_Nemertea_Baseodiscus_unicolor_TRINITY_DN103934_c0_g8_i1_m.145314 internal

DKRPFDRISGGFSSLDKKAFDRISGNDFSSFKKRNFDRIGQSFAGLEKRRFDRIGGMDFSNFRKRTIENTGQDGSSGLDKRAFDAIGAGDFSSLDKRNFDRIG

>PedalPep1_Nemertea_Lineus_lacteus_comp14934_c0_seq1.p1

MFGRGALCLLFLGLACAAPAVDKVEKRSVDGNRLEQLIDTDDKNAAEREINTLGGGSIHPESERELLDTLGGSAIHPEREALTSQELLESLEEADPEERRALLDSLSGGEIQPSRDLDKRTFDQIGHGGFSSFNKRNFDQIGHGGFSAFNKRNFDQIGHGGFSAFNKRNFDQIGHGGFSAFNKR

>PedalPep1_Nemertea_Lineus_lacteus_comp4754_c0_seq1.p1

FDQIGHGGFSAFNKRNFDRIGGGSLSSFDKRNFDSIGHGGFSSFNKRNFDKIGAGSLSGFAKRPFDQIGAGSLSSFAKRSFDKIGSGALSSFN

>PedalPep1_Nemertea_Lineus_lacteus_comp23132_c0_seq1_m.59076 5prime_partial

GALSSFNKRPFDKIGGGSFSSFAKRPFDKIGGGSFSSFAKKNFDSIGHGGFSSFNKKNFDSIGHGGFSSFNKKSFDSIGNGPLSSFVKRSADKTTEEKKDAE*

>PedalPep1_Nemertea_Nipponemertes_spec_TRINITY_DN43802_c0_g1_i1.p1

GFSDFSRRSFDPIGYASGFSSFNRKRSTKRNFDPISYGSSFSSFSKRNFDPISHVSSLDGFRKRNFDPIAYSSGFSQFVKRSKAENNDGLSRYASELASFSKPNKDHLTGQLRHSQ*

>PedalPep1_Nemertea_Tubulanus_polymorphus_comp38740_c0_seq2_m.79931 5prime_partial

HYGRGN

MEINFHILSAILLLLFNYTYAVPVAGKETKTQIKRSVSNDELSTDIKVIDSLIKEKNQEKEKDDARRYLDTLGGGAIHGGRRELDTHPDRRYLDQLGGAVIHGGRSLDKRPFDSIGHGSFSNFQKRVLAKRPFDAIGHSAFSSFQKRFDPIGHGEFSSFHKRPFDSIGHGSFSNFQKKPFDSIGHGSFSNFQKRPFDSIGHGDFSSFQKRPFDSIAHSDYKRGFDSIGHGEFASFNKRPFDSIGHGDFSSFNKKRGFDSIGHGEFSSFVKRSAGDKQQKENNQ*

>PedalPep1_Nemertea_Notospermus_geniculatus_g22953.t1

MWRRGVLCLLIFGFACAAPAVEKVEKRSVDSAALDQLIDNTDEKSDEDRGLLDTLGGASIHPVRDLLDTLGGASIHPGRRQTLDSLGLEIHPDQDEERRFLDTLGGANVHPLRREMLDTLGGASIHPGRDVDKRNFDSIGHSSFSSFTKRNFDPIGHSSFASFTKRNFDPIGHSRFASFKRNFDQIGSGTFSRFAKRNFDKIGSGTFSSFAKRPFDKIGSGAFSSFAKRPFDKIGSGAFSSFAKRPFDKIGSGAFSSFAKRSNFDDSEQEAEKKEE

>PedalPep1_Nemertea_Notospermus_geniculatus_g20615.t1

MPNSVCKGIETVIKRWQFVVEKRSVDSAALDQLIDNTEEKSDEDRGLLDTLGGASIHPVRDLLDTLGGASIHPGRRQTLDSLGLEIHPDQDEERRFLDTLGGANVHPLRREMLDTLGGASIHPGRDVDKRNFDSIGHSSFSSFTKRNFDPIGHSNFASFTKRNFDPIGHSRFASFKRNFDQIGSGSFSRFAKRNFDKIGAGTFSSFAKRPFDKIGSGAFSSFAKRPFDKIGSGAFSSFAKRPFDKIGSGAFSSFAKRPFDKIGSGAFSSFAKRPFDKIGSGAFSSFAKRSNFDDSEQEAEKKEE

>PedalPep1_Nemertea_Lineus_ruber_Lvir.rna.tri.14245.1_m.30137 complete

RGVLCLLILGVAWAAPAVENKVEKRSVDDNVLEQLIDTDAKNAAERELDTLGGTVIHPARDFLSSQEFLESLEEAGPEERRDILDSLGGAQIHPSRDLDKRTFDQIGHGGFSSFNKRNFDKIGHGGFSAFNKRNFDQIGHGGFSAFNKRNFDQIGHGGFSAFNKRNFDRIGSGSLSSFDKRNFDPIGHGGFSSFNKRNFDKIGSGSLSGFAKRPFDQIGGGALSSFNKRPFDKIGGGSLSSFNKRSFDKIGGGSFSSFAKKNVDSIGHGGFSSFNKRVFDRIGHGPLSKFDKWSADTKEEKNDPE*

>PedalPep1_Nemertea_Cerebratulus_spec_Contig3019.p3

MADGVEIPLSKTTEVATPNFVEWSFCETAESSIADFIKRPLCEATECSTPNFIKRSLGETAECTASNFIKRSLGETAECTASNFIKGSLRETAEGTTADFIKGPFVEAGESAVSDLVEIPLVEA*

>PedalPep1_Nemertea_Cerebratulus_spec_Contig3019_m.203831 complete

MFRRGVLVLLCMGLACAAPAVDKVEKRSVEDKVLEQLINDKDVERDNFDSLGGGAIHPERELLDTLGGAAIHPGRREMLDTLGDTSIHPGRRDMLDTIGAGVIHPNRDLDKRTFDRISHGGFSSFNKRNFDQIGHGGFSSFNKRNFDQIGHGGFSSFNKRTFDKIGGGSFSSFAKRPFDKIGGGAFSSFAKRPFDKIGGGAFSSFAKRPFDKIGGGAFSSFAKRPFDKISDGAFSSFAKRPFDKIGGGDFSSFAKRNFDPISHGGFSSFAKRNFDQIGHGGFSSFAKRNIDSINNGPLSGFVKRSADNTKEEKKDEE*

>PedalPep1_Nemertea_Lineus_longissimus_c46699_g1_i1_m.118901 complete

MMFGRGALCLLFLGLACAAPAIDKVEKRSVDRNVLEQLIDSDEKNAAERELDTLGGGSIHPETERALLDTLGGGAIHPGREVLSSRELLESLEEADPEERRALLDSLGGGAIHPSRDLDKRTFDQIGHGGFSSFNKRNFDQIGHGGFSAFNKRNFDQIGHGGFSAFNKRNFDQIGHGGFSAFNKRNFDQIGHGGFSAFNKRNFDKIGSGSLSSFDKRNFDPIGHGGFSSFNKRNFDRNGDGSSSGDFAKRPFDKIGAGAFSSFSKKNFDRIGHGGFSSFNKKNFDHIGHGGFSSFNKKSFDSIGDGPLSGFVKRSADKTKEEKKDAE*

>PedalPep1_Nemertea_Cerebratulus_marginatus_comp62379_c2_seq1_m.104638 complete

MIGRGVLVLLCLGLACAAPAAEKVEKRSVDEKVLEQLINADDKNARDTFDALGGSAIHPERELLDTLGGSAIHPGRREMLDDLGVGSIHPGRREMLDTLGGAAIHGRRDMLDSIGAGVIHPARDVDKRTFDKIGHGGFSSFNKRNFDQIGHGGFSSFNKRNFDQIGHGGFSSFNKRNFDAIGHGGFSSFNKRNFDKIGAGSFSSFAKRNFDKIGAGSLSSFAKRPFDKIGAGDFSSFAKRPFDQIGSGALSSFAKRNFDKIGASDFSSFAKRNFDSIGHGGFSSFAKKRNFDSIGHGGFSSFAKRTFDSIGNGPLSGFVKRSADNTKEDKKAEE*

## PedalPeptide-2

>PedalPep2_Phoronida_Phoronis_psammophila_comp68986_c0_seq1.p1

MVKRQLVALFLSAVIYSCSAKSFGKESEINGDNDQINDEALKDSAMIKRNLDSLGSGMLKRNLDSLGSGMLKRLDTLSSNMLKRNLDSLGSNMLKKNLDTLGSGMLKRLDTLSSNMLKRRLDSLGSGLLKRNLDSLGSGMLKRNLDSLGSGLLKRNLDSLGSGLLKRQSDYLRTKPLDDAELEEARRTLDSIGGGLLGRRHLDMIGGGLLGRRELGGYEDDEQVARALDRIGGGIIGRRQLEKLENGDIDRRFVDRIGGSILGRRQLIDRIGGNLVGRDISKSDKDSDEDRK*

>PedalPep2_Phoronida_Phoronis_australis_TRINITY_DN249692_c0_g1_i2.p1

MEKLHLQALLFLTVFHLCATESQKEAGTVRADRRSLDSLGSGMLKRHIDILGSDMIKRFDSLAKKNIDSLGSNMLKKNLDSLGSGMLKRKVDALKRLDSLGSNMLKKSLDSLGSGMLKRHIDTLGSNMLKRHMDYLQSKSNPLAVDGKGSNVLKRPLDQDESLGEARRTLDTIGGSLIGRRQLDTIGGGLLGRRESGAEAQNFERALDTIGGNLIGRRQADEMENSEMERRYLDSIGGSLLGRRHVLDRIGGNLIGRDLSEANETEQ*

>PedalPep2_Phoronida_Phoronopsis_harmeri_c116560_g3_i1.p1

FSIPLLLVVIIYNCVAKSFDNGEAEKDDHIDGGGLKRKIDSLDSGLFKRNLDSLGSGMLKRNLDSLGSGMLKRRLDSLSSGMLKRHLDSLGSNMLK

>PedalPep2_Phoronida_Phoronopsis_harmeri_c116560_g2_i1.p2

KRRLDSLSSGMLKRRLDSLGSSMLKKNIDSLGSGMLKRNLDSLGSGMLKRRLDSLGSGMLKRQLGYGTGDQALDEMLDENPRELDAIGGNLIGRRHLDTIGGHLLGRREEDAKQFERALDRIGGNLIGRRQLDSMQDDDLERRYLERIGGGLLAGDKL

>PedalPep2_Phoronida_Phoronis_ijimai_(P._vancouverensis)_comp89034_c0_seq1.p1

MILEATMDKLHLQALLLLTVFNFCATESQKEAETLHADRRSLDSLGSGMLKRQLDPLGSDMMKRLNSLSSAMTKKQLDSLGSSMLKKSLDSLGSGMLKRKVDALKRLDSLGSNMLKKSLDSLGSGMLKRNLDMVGSNMLKRHMKYLQSNRIPLDDNGSNLLKGDSLGDSRRTLDSIGGSLIGRRHLDTIGGGLLGRRESGAESQNFERALDTIGGNLIGRRQVEKMENGEMERRYLDRIGGSLIGRRQLLDRIGGNLIGRDLSEANESEQ*

>PedalPep2_Brachiopoda_Hemithris_psittacea_comp24689_c0_seq2.p1

AITAGEFSIGGRRITISPFPELFKTKMNAVSCILFLTSVLIACSTATLNKNAEKRNLDPLNSAFFKKRVLDTLGGYELKKRVLDSLNNAFWKKDNQHRANTNEKSRGLHDIGSGLIGRREIQDAITDMVLERIAARRGLDKIGSGLIGRDLIQLQAGKHQKSNNA*

>PedalPep2_Brachiopoda_Glottidia_pyramidata_comp40788_c0_seq1.p1

MNLNSVAMSFACFLILVITKANSLDHSNKNLAGKRDLDTLTSSLFRKRTMLDTVGSNLIKRRMPLDTVGTGVLRKRMLDTLGSDLLRKRMLDTVGSSLIKKSGNDKEIYNERALDEIASNFIGRRALDEIASTMFGRRALDEIASSMFGREVKNLKPHQRRALDEIASQMFGRRALDEIASRMFGRALDEIASSMFGREAGRNMKEEDREELKKEIRAHLQQMPPLEEDSN*

>PedalPep2_Brachiopoda_Lingula_anatina_comp118813_c0_seq2.p1

MQWNNLVIPIVCCLIAIVTRVNSLDDSKKKVVERRDLDTLGSSLLRKRTMLDTVGSDLLKKSSEDKRTVDTLDSSFLRKRMLDSLGSNLLRKRMLDSLGSNLLRKRMLDTLGSAFIKKSIPNHGDVEMFDERALDEIASNLIGRRALDEIASSMFGRRALDEIASSMFGREVKNLKPSQRRELDEIASRMFGRRALDEIASSMFGRALDEIASSMFGREAGKNRKQEDEEGFKATRNRLQQTPPMEEERS*

>PedalPep2_lingulaAnatina.g30274.t1 Brachiopoda

MQWNNLVIPIVCCLIAIVTRVNSLDDSKKKVVERRDLDTLGSSLLRKRTMLDTVGSDLLKKRSEDKRPVDTLDSSFLRKRMLDSLGSNLLRKRMLDSLGSNLLRKRMLDTLGSAFIKKSIPNHGDVEMFDERALDEIASNLIGRRALDEIASSMFGRRALDEIASSMFGREVKDLKPSQRRELDEIASRMFGRRALDEIASSMFGRALDEIASSMFGREAGKNRKQEDEEGFKATRNRLQQTPPMEEERS

>PedalPep2_Brachiopoda_Novocrania_anomala_Nano.rna.tri2.203060.1.p3

MRHLDSVGRRPVKRRLDSVCQRPVKRRIDSVGRRLVMRRLDSVRRRPVRRRLDSASRRPVSRHLDSGGR

>PedalPep2_Brachiopoda_Novocrania_anomala_Nano.rna.tri2.158834.1.p1

DSGLFKKRMLDTLSAGIFKKRLIDSIGSDLLRKRFVDALRKRSRSNSKASQGKTHSDDDKRELDEISSSLIGRRALDEIANGLIGRRELEDAMYDIASMGIGRRTLDEISSGLIGRRALDEISSGLISGRALDEIANGLIGKKALDEISSGLIGREAVAQNTKLAAAKKLVSSKHASARSH*

>PedalPep2_Brachiopoda_Novocrania_anomala_Nano.rna.tri2.111424.1.p1

MDLKTVVFYAALVTLASAASLKPVYKSTEHSPKEKREFDTLGSDVLRKRDLDTTGVSLDSDSKRTLDTLNSGLFRKRMLDTLDSGLFKKRMLDTLDSGLFKKRMLDTLDSGLFKKR

>PedalPep2_Brachiopoda_Laqueus_californicus_comp37988_c0_seq3.p1

MSWFIICLSAAIGLISAKSVQENEVKRTIDPLSNSFFKRTLDPLAHFDLKKRLDPLADSFFKKRALDPLTDSFFKKRGMDPLADSFFKKRVMDTLTSHFFRKRIIETLGDSLMKRADKNDKDSSKRYLDGIGSGLIGRREIEEALENLILNRFIEKWALEKIAADHRELKTKPNKQQSIGDKSKENGI*

>PedalPep2_Brachiopoda_Terebratalia_transversa_Ttra.rna.tri.9200.1.p1

MTYTLWLILCLPAISLTSAKSVQENASKRTIDSLNNSFFKRALDRPLADFDLKKRQLDSLTGSFFKKRAIDPLADSFFNKRGLDPLADSFFKKRGLDPLTNSFFKKRVMDSLARHFLRKRVIETVGDKRASKNDDNKRYSLDGIGSGLIGRREIEEVLEDMINSIMEKWALDRIAEDHKELKAKTNTQSIAGKEKSNGK*

>PedalPep2_Nemertea_Cerebratulus_spec_Contig420_m.194814 complete Contig7083_m.218182 complete

MSGRVPLSLLGLTFVACLLSNGVSAEEKRMLDAVSGGLLKRSEGEEQKRMLDSISGGLLKRSGEEQKRMLDSIGSGLLKKKRMLDSIAGDLLKRSGEDQKRMLDRVSGGLMKRGDEKRMLDRVSGGLMKRDNEKRMLDRVSGGLMKRENEKRMLDRVSGGLMKRDDEKRMLDRVSGGLMKRDDQKRMLDSIGSGLLKKRWLDAVGGGLLKKRMLDRIGSGLLRKRFMDPIDEGLLKKKDADSSEEERRYIDSLNDDLLRRRELNEAVAAEMLNRRHIDPISAALLRRHLDSIDNGLLRRRYIDELGSDLLRREEENEAEASEEEKEE*

>PedalPep2_Nemertea_Baseodiscus_unicolor_TRINITY_DN98607_c0_g2_i1.p1

MSGYIPLTGLGMVLACLCLCGTSAGDDKRMLDAIGSGLLRKRKMEETPDKRMLDSIGSGLLKRSNEQDKQTLDSMENGLLKKRMLDSIGSGLLKKRMLDSIGSGLLRKRFLDPIGTGLLRKRMLDSIGSGLLKRSDGETDDERRYVDTIADDLLRRRYINDLLERRDLDAVADDLLTRRHLDPIANSLLRRHLDRISDNLLRRRYIDQIGSDLLRREQGDTMEDSVVDKAK*

>PedalPep2_Nemertea_Lineus_ruber_Lvir.rna.tri.43529.1_m.81413 3prime_partial

MSGRVPLSILGLALVASLLSNGVSGEEQQSGSNGGQEHKRMLDRIGGGLLKRSGDGEQKRMLDSISGGLLKKRMLDSIAGDLLKRSGDDQKRMLDRVGGGLLKRDEEKRMLDR

>PedalPep2_Nemertea_Lineus_ruber_Lvir.rna.tri.5525.1_m.12417 5prime_partial

DEEKRMLDRVGGGLLKRDEEKRMLDRVGGGLLKRDEEKRMLDRVGGGLLKRDEEKRMLDRVSGGLIKRDEEKRMLDRVSGGLIKRDQEKRMLDRVSGGLIKRDQEKRMLDRVGGSLLKKRWRDQVGGSLLKKRMLDRIGSDLLKKRLMDPIEEGLLKKKDSAGTDEERRYIDSLSDDLLRRRELNELINTRHIDPINANLLRRHLDTIDNDLLRRRYIDALGSDLLRREEATDEESAEEKEE*

>PedalPep2_Nemertea_Lineus_longissimus_c19168_g1_i1_m.10393 3prime_partial

MSGRVPLSILGLALMACLLSNGVSGEEKRSGSMAGEGQDHKRMLDRIGSGLLKRNGDGEQKRMLDSISGGLLKKRMLDSIAGDLLKRSGDDQKRMLDRV

>PedalPep2_lineusLongissimus.21741 Nemertea

LSILGLALMACLLSNGVSGEEKRSGSMAGEGQDHKRMLDRIGSGLLKRNGDGEQKRMLDSISGGLLKKRMLDSIAGDLLKRSGDDQKRMLDRVGGGLLKRDEEKRMLDRVGGTLLKRDEEKRMLDR

>PedalPep2_Nemertea_Lineus_longissimus_c19168_g1_i1_m.10393 3prime_partial

MSGRVPLSILGLALMACLLSNGVSGEEKRSGSMAGEGQDHKRMLDRIGSGLLKRNGDGEQKRMLDSISGGLLKKRMLDSIAGDLLKRSGDDQKRMLDRV

>PedalPep2_Nemertea_Lineus_longissimus_c37531_g1_i1_m.43255 5prime_partial

EKRMLDRVGGRLLKKRWLDHVSGSLLKKRMLDRIGSGLLKKRLMDPIEEGLLKKKDAASNDGERRYIDSLSDDLLRRQELNEIIKTRHIDPINANLLRRHLDSIDNDLLRRRYVDTLGSDLLRREEAKDEESADEKEE*

>PedalPep2_Nemertea_Lineus_lacteus_comp20144_c0_seq1_m.39316 3prime_partial

MSGHVPLSILGLALVACLLSNGVSGEEKRSGSMADQGQEHKRMLDRIGGDLLKRNGDGEQKRMLDSIGSGLLKKRMLDSIAGDLLKRSGDDQKRMLDRVGGGLLKREE

>PedalPep2_Nemertea_Lineus_lacteus_comp23983_c0_seq1_m.67101 5prime_partial

RDQEKRMLDRVSGGLIKRDEEKRMLDRVSGGLIKRDDEKRMLDRVGGGLLKKRWLDRVGGSLLKKRMLDRIGSGLLKKRLMDPIEEGLLKKKDAASTDEERRYIDSLSDDLLRRRELNELINTRHIDPINANLLRRHLDSIDSDLLRRRYIDAVGSDLLRREETNNEESAGETEE*

>PedalPep2_Nemertea_Riseriellus_occultus_TRINITY_DN15078_c0_g1_i1.p1

ISFESVDYKDLKRNHRIIGLFIGLQQRVKISTMSGRVPLSILGLALMACLLSKGVSGKESGSMADEGQEHKRMLDRIGSDLLKRGGNGEEKRMLDSISG

>PedalPep2_Nemertea_Tubulanus_polymorphus_comp37803_c0_seq1_m.67207 complete

MALTSTSWFNQMLLSLTICILISVIASHEDEKRMLDPIGSSLFRKRDLEDAPSKRMLDRVGSSLLKKRMLDRVGSSLIKRDEESADKRMLDRVGSSLLKKRMLEPSQFRKRALSRRTLDEIGDDLLRRQIANAILRKHEMQEVSDSLLRRTLDEIGNGLLRRRVNNEERSDSEGH*

>PedalPep2_Nemertea_Notospermus_geniculatus_g23770.t1

MADTAAIKKRLNVADTAANKRRLNMADTAAIKRRLNMADTAAIKRRLNMADTAAIKRRLNMADTAAIKRRLNMADTAVIKRRLNMADTAAINRRLNMADTAAIKRRLNMADTAAIKRRLNMADTAAIKRRLNMADTAVIKRRLNMADTAAID

>PedalPep2_Nemertea_Notospermus_geniculatus_g3841.t1

MDKYHLSASSTRKHPSGCNKRSQFSTMSGRVPLSILGLALVACLFLNGVSAEEKRMLDSIGNGLLKKRMVDDIGEEQKRMLDRVSGGLIKRDQEKRMLDRVSGGLMKRDEEKRMLDRVSGGLMKRDEEKRMLDRVGQGLIKRDDEKRMLDRVGGRLLKKRYLDAVGHGLLKKRMLDRIGSGLMRKRFMDPIDQGLLKKKDADTADEERRYIDTLEDELLRRRELNAALAQDLINRRHIDPISVALLRRHLDNIGDGLLRRRYIDTIGNDLLRREEENEAEAANEKEE

>PedalPep2_Nemertea_Nipponemertes_spec_TRINITY_DN14149_c0_g2_i1_m.21074 internal

ISHGMMKRDEPEHELAKRRLDAISGGMFKKRMLDPISGGMFKKRMLDPISGGMFKKRSLDRINGGLIRKRKEVDEVPKGTRMLDPIADDLLRRRFLDAAIADDYL

>PedalPep2_Nemertea_Cephalothrix_hongkongiensis_comp36180_c0_seq1_m.42325 5prime_partial

KYQFCWKCPFCVLSTEAVEKRMLDAISSGFIKKRALDSIASGFIKRDGESSKRGFDAIGSGFVKREEGKRSLDRISSGFIKRDEMAKRRLDSIGAGFIKKSAEKRHLDSISSGFVKRNLHKRVLDGISSGFIKRGVDVPRTLDSLADDLLRRHIDSKEEREKATEEVLRRELDDIADALLRRRQIDSLADDLLRREVAKEHGN*

>PedalPep2_Nemertea_Paranemertes_peregrina_comp27111_c0_seq2_m.39039 5prime_partial

HYGRGLQLPVTTVTAKNHKRISVSPNAT

MTMNQLLAALGTLLLVCVVKSSLAADKRMLDSISSGFVKKSAEKRMLDSISSGFVKRELPNMDEPEHEQSKRMLDPIGGSMFRKRMLDPISGGLFKKRMLDSIGRGLIRKRKELDEPSQKQIRTLDTIADGLLRRRYLDAIADDLLRRDIGTRQLDSIADGLLRRRELDGLADELIHRRLNAIKKEQH*

## PedalPeptide-3

>PedalPep3_Brachiopoda_Lingula_anatina_comp151030_c0_seq1.p1

MQGNVFSVWCALLMALVLLCHKAASQDPVEKLGTCEDDTSPRASTCALCDTFGVTKQEDCCTDGEILQECADALKAVVYHVGAQKPQNGGSSEETIQKNMVTKAIYKALRGHEDNNEDSEEGVWGDFASDVQKNDGDEDDTDEQSNDMAADKRYGSVFRFPYGKRYGSIFRMPSGKRYGSVFSMPYGKRYGSVFQLPAGKRYGSVFRLPGGKRYGSVFRLPSGKRYGSVFRLPGGKRYGSVFNLPAGKRYGSVFRLPYGKRYGSVFRMPIGKRYTSELQDPYSKRYGAILRFPWAKRDENWGQDEESDLENDAGPYYYDFDESTDEDNEPDTNELDKRYGAILRYPWGDHWKRTVSMSSFPWRPHESAVRRNNDIDDVDADYPHNSQRQNGEREEIVAKRYGLLLGWPWASTGKIRTSYYGRY*

>PedalPep3_Brachiopoda_Novocrania_anomala_Nano.rna.tri2.44829.1.p1

MRGLEIVLLLGLLTYLTQAQEASQEDLCSNNPDILVACKVCEAFQIPRESCCEKRDDFFACREQLKVLVDKYADRADGDLEQNGGEADDISENEDADDSYGSDNADARKALVDAYLKGRDDFDVNPDGEWGNFQPDEENSPDKRYGNMMSFPWQRLNTGGSATEEKRFGSLMSFPWRNPNTAKELPDKRYGSVIGFPWSRFSATDSDYEKRADQAAEKRYGQVFSTPWRNYVSSHYHKREPTDADDQPAVDKRFGSVFKFPWHSNSKRFGPLFSFPTNSNEK*

>PedalPep3_Brachiopoda_Hemithris_psittacea_comp27159_c0_seq1.p1

MFSKGSSVLVILTLQIILVECGPLQDGCADARLCGICSRAFDINVKDCCKGGELFTKCADVIEYVIQESKETPPKSRPEPGEHDDKDEQREEKRYGSVFWRPAGKRYGNLFVRPAGKKNEGLRRSDFDGLDKRYGSMWARPVGRKRSGGKSREGDGVDQLDGDWGMKRKRYGGLWRNPAGK*

>Tubulanus_Polymorphus.14240 Nemertea

MKSVVASMLVLATCLLAHKGADEISSNEVDKRPFGSFSKIDKRIPPHDDSERRRARWYPQDVIDRLTYRIMLKRDSDDSSDPSNEIDTAKRPFGTFSHADKRPFGTFSHADKRPFGTFSHADKRPFGSFSRAADKRPFGGFSGADKKRAFGPVYRPPYKRPFGTFSARPAKRPGFGRMTGRVKKPFGPVTSRLDKRPFGTFSGVNKRPFGSFTSRFDKRPFGPVTHWNKREPDEDGEQVYY

>PedalPep3_lineusLongissimus.37940 Nemertea

MRVLPNIVLLSLQLLLISRLTFQEKIQTDSGVDIKRVERSVDDGENSFGSEDKRVFGSITKPVGKHGSARRPPATREEHVRVSDIKREDEPDYYKLLDELMDRRIESKRAAYDANAGDEGDGGSPDDEGNNMLYLALKQGEHNDNGDDDGVLHSYDKRPFGSFTRKLSKRPFGSLTGGFHKRPFGSFTRKLQKRPFGSMTGQMKRPFGSIISGLSKRPFGGFTSRMKKPFGSLSKVLSKKPFGSFSRPRSFNKREDEDVEKRPFGSFSRPRWNTKRDDDNDVEKRPFGSFSRPRWNTKRDDDNEVEKRPFGSFSRPRWNTKRDDDNEVDKRPFGSFSLPRSYQKREGDDMAKRPFGSFTRPNTRSFPTEDDTDDIIEKRPFGSFTRWRSRTRGKRDGADEVEHDTPVYY

>PedalPep3_Nemertea_Malacobdella_grossa_comp201934_c0_seq1.p1

DDDDVDDDEDSEKRYFDTDEDIDKRPFGSLSGRYKRPSVPMWGRVGKRASPMLGRVGKRPVPMWGRVGKSPAPMG

>PedalPep3_Nemertea_Cerebratulus_marginatus_comp1850328_c0_seq1.p1

ESKRSPYDEDGEVDRGSPDEERESMLHLALMQGEQGEDGDDDQIFQAYDKRPFGSFTRKLSKRPFGSLTTRFRKRPFGSFTRK

>PedalPep3_Nemertea_Cerebratulus_marginatus_comp1493205_c0_seq1_m.149157 internal

RTGVSGLSKRPFGSFSSRPKKPFGSFSRPRSYNKRDDEIDDDVEKRPFGSFSRPWKSKRDEDEEIEKRPFGSFTRPRNYDKRDGDDIEKRPFGSFTRHFTRSFPKR

>PedalPep3_Nemertea_Baseodiscus_unicolor_TRINITY_DN58587_c0_g1_i1.p1

GKRPFAPMSGVDSPADSLSGYELEAKSNEKRPFGSFTKWRTNKRPFGPIHGVVGKRNQAEHADDGDVNYPVFY*

>PedalPep3_Nemertea_Baseodiscus_unicolor_Contig19103_m.254491 5prime_partial

FTGRVGKRPFGPFTARVGKRPFGPFTSRVGKRPFGSFTSRVGKRPFGSFSGRPRKRPSGITMPGETDKRPFVLHLVKEPKGRFSLLFASSS*

>PedalPep3_Nemertea_Baseodiscus_unicolor_Contig19103.p1

GKRPFAPMSGVDSPADSLSGYELEAKSNEKRPFGSFTKWRTNGRLSVSPGIVIPDGRFRGRPENEPNGRLPTRLVNEPNGRLPTRLVNGPNGRLPTRAVNGPNGRLPTRPVN

>PedalPep3_Nemertea_Nipponemertes_spec_TRINITY_DN13666_c0_g1_i1_m.60797 5prime_partial

KRPMGPIYGKIGKRPFGPSRGRIGKRPMGPIYGKIGKRPFGPSIGKIGKRPGPIYGKIGKRPMGPIYGKIGKRPFGPSSGRIGKRSSSNKRPFGPIHGVIGRRSTE*

>PedalPep3_Nemertea_Nipponemertes_spec_Contig10158_m.121197 internal

FGPSSGKIGKRPGPIYGKIGKRPFGPSRGRIGKRPMGPIYGKIGKRPMGPIYGKIGKRPFGPSRGKIGKRPFGPSSGKIGKRPGPIYGKIGKRPFGPSRGRIGKRPMGPIYGKIGKRPFGPSIGKIGKRPGPIYGKIGKDRKSGSAGMPRPISY

>PedalPep3_Nemertea_Notospermus_geniculatus_g29987.t1

MHRQIERKRFVNDVDEDEENNLEREEGDGNSLLRLALWREGMDDDDDDDSENETFQPYEKRPFGSFTRKFSKRPFGPLTARFLKRPFGSFTRKYQKRPFGSMTGGVKRPFGSMISGLSKRPFVGTLSKRPFGSFSRWRSVREDNEDVEKRPFGGTFSRKWRFPKRSNYDDVEKRPFGSLTRPIDYQKRDGDDIEKRPFGSFTRSWSRNFPKRDGESVVDKRPFGSFTRWKSGRIPGKRADSSAGLEEVEMKTPLYY

>PedalPep3_Nemertea_Lineus_lacteus_comp17592_c0_seq2_m.28916 5prime_partial

NEVDKRPFGSFSRPRSFQEREGDDMEKRPFGSFTRPNTRSFPTEDDTDDVIEKRPFGSFTRWRSRIRGKRDATDHVENDTPLYY*

>PedalPep3_Nemertea_Lineus_lacteus_comp17592_c0_seq1_m.28911 3prime_partial

MKVMPNVVLLSLQLLLISRISLQEKIRTDSGGDIKRVERSLDDDDNSFGGEDKRVFGSITKPVGKHGSGRPPASREEAVQVSEIKRENGQDYYKLLDELMDRRIESKRAAYDADEANEGSPDDEGNYMLYLALKQGEHDDDGDDDNVFQSYDKRPFGSFTRKLSKKPFGPLTARFRKRPFGSFTRKFQKRPFGSMTGQMKRPFGSIISGSSKRPFGSFTGRVKKPFGPLSKVLSKKPFGSFSRPRSFDKREDDDVEKRPFGSFSRSRWNTKRDDDDEVDKRPF

>PedalPep3_Nemertea_Cerebratulus_spec_TRINITY_DN37437_c0_g1_i1_m.10471 complete

MRVLTTTILLSLQLILMGHVTKQENAKSVAETDGEIKRVARSLGGEEGSFNDEEKRVFGSITKPVGKHRSGRQPSTRDETIQVSDIKRDDDSDYYRLLNELMERRIESKRSSYADDIDDANGGDPDDERESMLHLALMQGEQDDDADFDDIFKTNEKRPFGSFTRKLTKRPFGPLTGRFRKRPFGSFTRKFSKRPFGSMTGLPKKRPFGSMISGLSKRPFGSFSARWKKPFEPFRRQRSYIKRDEEEESADDVEKRPFGSFSRPQSKWKRDDDEENVEKRPFGSFTRPRIFQKREQEDGVEKRPFGSFTRPFTRSLAKRPFGSFTRWRTNLPGKRRVADEGLEGEIDTPLYY*

>PedalPep3_Nemertea_Lineus_ruber_Lvir.rna.tri.29307.1_m.57659 complete

MRLLPNVVLLSLQLILISHFTLQEKIETDSDGDIKRVERSLDDDDNSFRGADKRVFGSITKPVGKQGSGRPPARREEHVQVSEIKREEDPDYYKLLDELMDRRIESKRTLYDADEGDEVDGGSPDDEGKSMQYLALMQGEHDDDRDDDNVFESYDKRPFGSFTRKFSKKPFGSLTRRLDKRPFGSFSRKFSKRPFGSMTGQMKRPFGSIISGLSKRPFGSFTGRVKKPFGSLSKILTKKPFGSFTRPRSFNKRDDNDVEKRPFGSFSRPRWNTKRDDDNEVDKRPFGSFSRPRSYGKREGDDVEKRPFGSFTRPYTRSFLTESDTDDVIEKRPFGSFTRWRSHIRGKRDGADEVEKDTQLYY*

>PedalPep3_SWD_Nemertea_Lineus_longissimus_c51536_g1_i1.p7

ERSARPTQKRQYEGIFITDGSQKRSWDALGIPDKRSWDALGIPDRRSWDALGIPDRRSMGLNEKDVKALASFIASRNNRRQ*

>PedalPep3_SWD_Nemertea_Notospermus_geniculatus_scaffold99.g4987.t1.p1

MTVLWAVTLISVLLLELFLLCLSHVNAAPLHETDVQDDKDISRNKRALARWGGIPDPHKKRQYEGIFITDGSQKRSWDALGIPDRRSWDALGIPDRRSWDALGFPDRRSLDLSEQEARALSSFFASRQGQRRQ*

>PedalPep3_SWD_Nemertea_Cerebratulus_marginatus_comp59832_c0_seq8_m.76605 complete

MASLMNRTLISVLLLELVLLCISHVNAAPLLDAEDQKVAEQDVSRRKRAFSAAAWDDIPNPHKKRQYEGIFITDGSQKRSWDALGIPDKRSWDALGIPDRRSWDALGIPDRRSLGLSGQDVKALASYFANRQRRQ*

>PedalPep3_SWD_Nemertea_Cerebratulus_spec_TRINITY_DN36502_c1_g1_i1.p1

MASLINRTLISVLLLELVLLCISHVNAAPLLDAEDQKVADQDISRRKRAFSAAAWDDIPNVHKKRQYEGIFITDGSQKRSWDALGIPDKRSWDALGIPDRRSWDALGIPDRRSLGLNGQNLKAALASYFASRQRRQ*

## Pleurin

>pleurin_Brachiopoda_Glottidia_pyramidata_comp88098_c0_seq1.p1

VAITAGGLVRVLLSTLAKTHGFYFTDSKEYDYPRIGRRNQIVSTKAHFDIENKGLYGSNIPIPGAAQVDLANKNIEALLQKLSQSLAYDDAQRFGSPWLRKRRSLLAQRKKKKV

>pleurin_Brachiopoda_Lingula_anatina_comp14311_c0_seq1.p1

MILKFTCYVGLACVLLSALQGTYCFYFTDSKEHDYPRIGRRNQIVSTKAHFDFENKGLVNSNIQLPGSSDVIFANRNIEALLQRLSVPMVSYSDASRLSDAWLRRRRSLLSYQKSQ*

>pleurin_Nemertea_Baseodiscus_unicolor_TRINITY_DN72441_c1_g1_i1.p1

ERDSKENEYPRIGRRNFVQHGPNTIEDSDLTEQIAADAGEFYNQRLLANALLKELAARGTKPFPFSKPQTGAIDNDIHSTYGSR

>pleurin_Nemertea_Notospermus_geniculatus_g3565.t1

MESRLVLAQALLVVCLVHFAHGIFFTSSKENEYPRIGRRNFVARADSTDLNDSRDLYEQRLPVLAKALYRELSARGVKPYQFKGMRTENGADDFDTKTRHILGED

>pleurin_Nemertea_Lineus_ruber_Lvir.rna.tri.59676.1_m.104115

MESRHILVQALLVVCLTHLAHGIFFTSSKENEYPRIGRRGFVAPRPDIDLNDSRDLYDNRLLAKALLRELTSRGAKPFALKVQTPPLHGADNFDTKTRQILTDENGYSQN

>pleurin_Nemertea_Lineus_lacteus_comp7686_c0_seq1_m.8969

KKSRHILVQALLVVCLTHLAHGIFFTSSKENEYPRIGRRGFVAHRPDFDFNDSSDLYDNRLLAKALLRELAARGGVKPFKLQTPTGQGADDFDTKTRQILSEDTHNGYPQN

>pleurin_Nemertea_Cerebratulus_spec_TRINITY_DN37573_c0_g3_i1.p1

MESRHILVQALLVVCLTHLAHGIFFTSSKENEYPRIGRRGFAPSRQDTDLNDSRDLYDNRLLAKALFRELSARGMKPFKAQAAAEHGTDQFDTKTRQILNEATGYSQD*

>pleurin_Nemertea_Lineus_longissimus_c46106_g1_i3.p3

MESRHILVQALLVVCLTHLAHGIFFTSSKENEYPRIGRRGFVAPRPDFDLNDSRDLYDNRLLAKALLMELSARGVKPFKLPTQVEHGADDFDTKTRQILNEDTHNGYPQN*

## Proenkephalin related

>Proenkephalin-related_Brachiopoda_Hemithris_psittacea_comp27159_c0_seq1.p1

MFSKGSSVLVILTLQIILVECGPLQDGCADARLCGICSRAFDINVKDCCKGGELFTKCADVIEYVIQESKETPPKSRPEPGEHDDKDEQREEKRYGSVFWRPAGKRYGNLFVRPAGKKNEGLRRSDFDGLDKRYGSMWARPVGRKRSGGKSREGDGVDQLDGDWGMKRKRYGGLWRNPAGK*

>Proenkephalin-related_Brachiopoda_Novocrania_anomala_Nano.rna.tri2.44829.1.p1

MRGLEIVLLLGLLTYLTQAQEASQEDLCSNNPDILVACKVCEAFQIPRESCCEKRDDFFACREQLKVLVDKYADRADGDLEQNGGEADDISENEDADDSYGSDNADARKALVDAYLKGRDDFDVNPDGEWGNFQPDEENSPDKRYGNMMSFPWQRLNTGGSATEEKRFGSLMSFPWRNPNTAKELPDKRYGSVIGFPWSRFSATDSDYEKRADQAAEKRYGQVFSTPWRNYVSSHYHKREPTDADDQPAVDKRFGSVFKFPWHSNSKRFGPLFSFPTNSNEK*

>Proenkephalin-related_Brachiopoda_Lingula_anatina_comp151030_c0_seq1.p1

MQGNVFSVWCALLMALVLLCHKAASQDPVEKLGTCEDDTSPRASTCALCDTFGVTKQEDCCTDGEILQECADALKAVVYHVGAQKPQNGGSSEETIQKNMVTKAIYKALRGHEDNNEDSEEGVWGDFASDVQKNDGDEDDTDEQSNDMAADKRYGSVFRFPYGKRYGSIFRMPSGKRYGSVFSMPYGKRYGSVFQLPAGKRYGSVFRLPGGKRYGSVFRLPSGKRYGSVFRLPGGKRYGSVFNLPAGKRYGSVFRLPYGKRYGSVFRMPIGKRYTSELQDPYSKRYGAILRFPWAKRDENWGQDEESDLENDAGPYYYDFDESTDEDNEPDTNELDKRYGAILRYPWGDHWKRTVSMSSFPWRPHESAVRRNNDIDDVDADYPHNSQRQNGEREEIVAKRYGLLLGWPWASTGKIRTSYYGRY*

>Proenkephalin-related_Nemertea_Cerebratulus_marginatus_comp4745_c0_seq1.p1

AEGLPAASADYESLFRKIDACKKPSERLMCTQCADELQLGLTEEDCCSSEVGYNVCDFCVNDIEECLIIRDEFYAGIMQKREEKRKRFGRLFFNKKSTE

>Proenkephalin-related_Nemertea_Cephalothrix_hongkongiensis_comp205560_c0_seq1.p1

LEWLISIRTHCRTDSLARATCTQCGEMPVFVSTDECCSNLLAYNVCDYCVNNPTDCFGLLDDFVNGVQIVKKRYGRILSDNEEKKRSVKRQYGRLFTGGWGGNNRELFGRPSK*

>Proenkephalin-related_Nemertea_Lineus_longissimus_c41631_g1_i1.p1

MQNFLSLILFLCVAVGLTAAASDYEALFDQIDACKKPAEKLMCVRCAEKFELGLTEQECCTTEVGYNVCDICVNDMEECLAIRDEFYSGVLQKRQEKKRFGRLFFNKKGDDMRKRFGTVFFGGKERPYFGKAKREVKKSQ*

>Proenkephalin-related_Nemertea_Lineus_ruber_Lvir.rna.tri.56754.1.p1

MQSLISLILSLCVAIGVTAATSDYETLFNQIDACKNPSEKLMCARCAGHFQLGLTEEECCTTEVGYNVCEICVNDIDDCLAIRDEFYSGVLQKRQEKKRFGRLFFNKKTRSDDMRKRFGTVFFGGKGRSYFGKTKREVKKSA*

>Proenkephalin-related_Nemertea_Notospermus_geniculatus_scaffold79.g4233.t1.p1

MQSLVSLFLALGIALGLTAATSDYEHLFTEIDACKKPSDRTMCGRCEELQLGLTEQDCCTTEVGYNVCDYCVTDVEDCIAIRNEFYSGIVQKRQEKKKRYGRLFFNKKSSSDDMRKRYGTLFLGRHGRSAEAKREVKKSS*

>Proenkephalin-related_Nemertea_Cerebratulus_spec_TRINITY_DN29339_c0_g1_i1.p1

MQSLLSVILSVFLAAGLTAASADYEALFKKIDACKKPTDRMMCTRCADDFQLGLTEEDCCTSEVGYNVCDFCVNDIDECLAIRDEFYAGIMQKREEKRKRFGRLFFNKKTGSDDMRKRFGTVFFGGRGRSYAGKAKREVKKSS*

>Proenkephalin-related_Nemertea_Tubulanus_polymorphus_comp36140_c0_seq1.p1

MRIIVAAVLVIYGVAVFVSLCSGADEGKSRKRRESRIWEDSPCNTDPGAKNMCRSCNSLPLGLTTEKCCGSEMDFEVCDYCVENVSDCISIMKEYFELESGVSRRKRFGRLLAERSHAEDDGTTDVRKRFGHLFLSRPSSNRGYITYRRKPKRFGKLFIGGYRG*

>Proenkephalin-related_Nemertea_Lineus_lacteus_comp21592_c0_seq2.p1

MTKKMQTLLALILSICIAAGLTAAASDYEALFNQIDACKKPTEKLMCARCAENFELGLTEEECCTTEVGYNVCDFCVNDIEECLAIRDEFYSGVLQKRQEKKRFGRLFFNKKGDDKRKRFGTVFFGGKSRSHFGKAKREVKKSP*

## Prohormone-2/GNXQN

>ProHo2_GNXQN_Brachiopoda_Glottidia_pyramidata_comp9797_c0_seq1.p2

MMLLKCAVVASLLGVLAAAPALTEKRSEETIFFGNHQNKVREKKSDPGMEAKKKKV

>ProHo2_GNXQN_Nemertea_Baseodiscus_unicolor_TRINITY_DN51134_c0_g1_i1.p1

MIYRFIIVIGVVWYVSHALPLPKANDALIKEVTKAAKETKESKIVKRSEESVIVGNHQNKAREKKSDPAVETKEENNRDENARIEVKAEDIDPSKINEKAKEDENEILQEAVEKQAEVGQSSPEEEDEVEEDRKVIEVEDDEDGDDDDDDDSISEADREKIKIVEEEKELEKLPKDGPIVFGHETPGEREDDGRIYEPESQDSYDVIDSQEVIDENNAKEVEDDHVSKEMMSADEEELEAEDEVMNGPNSPLLQGYGSSRYEPYDFLNRETYYEYPIPYPWRQSKTRNTEGLDRTKREAAMLGLDRTKREAAMLGLDRTKR

>ProHo2_GNXQN_Nemertea_Cerebratulus_marginatus_comp883509_c0_seq1.p1

MMVYRALLALTLVLCLTQALPIKKANDLSKEISDVAKENTRIVKRSEESVIIGNHQNAPREKKADTDVNVKDEQNRDENGKIEVTENEIDKSNIVDSSEN

>ProHo2_GNXQN_Nemertea_Cerebratulus_spec_TRINITY_DN37572_c0_g1_i2.p1

MMVYRALLALTLVLCLTQALPVKKANDLSKEIADVAKENTKIVKRSEELVQIGNHQNAPRTKKDDPTLKVKDEQNRDENGKIVIKENEIDKTKIVDSSETDNTPLETEAMEKAVMDLAKVADTPSDDDLEEKVKVVEEEKEIEKLPKEGEVEEDKETEQDEVFQPQDIFRLGAREEEVNDVNSDEVPDEESEVLDSSEEVPPEALMTANMEMAQDRPAGQDYQYDEDYNSLYSDPYRWLDRKKRDANRQKREALQGSEAGMSMAANEEDTKSRSKRDTEYPLSLNDLYNLYYKYKALKDEEEAEEEAMEPVYEVPEYQDEYGAEAPAAAYDYGVEQEMAPPSEEWEPEAVNNAIQEELQLEAMMQPSVEAEAPYYQADVLPEDIEEELEEEEELPAWEVPSSESAYYNPMEYAPVSRPMEDEYVPNKRTDPELLSLMPGIKRGDDFYPAYTEEQEPWQALIPPAAEKRGVMEEYARLYRLARALKRSREDSVEDRWESLLGEYKKK*

>ProHo2_GNXQN_Nemertea_Cerebratulus_spec_TRINITY_DN37572_c0_g1_i4.p1

MMVYRALLALTLVLCLTQALPVKKANDLSKEIADVAKENTKIVKRSEELVQIGNHQNAPRTKKDDPTLKVKDEQNRDENGKIVIKENEIDKTKIVDSSETDNTPLETEAMEKAVMDLAKVADTPSDDDLEEKVKVVEEEKEIEKLPKEGEVEEDKETEQDEVFQPQDIFRLGAREEEVNDVNSDEVPDEESEVLDSSEEVPPEALMTANMEMAQDRPAGQDYQYDEDYNSLYSDPYRWLDRKKRDANRQKREALQGSEAGMSMAANEEDTKSRSKRDTEYPLSLNDLYNLYYKYKALKDEEEAEEEAMEPVYEVPEYEDEYGAEAPAAAYDYGVEQEMAPPSEEWEPEAVNNAIQEELQLEAMMQPSVEAEAPYYQADVLPEDIEEELEEEEELPAWEVPSSESAYYNPMEYAPVSRPMEDEYVPNKRTDPELLSLMPGIKRGDDFYPAYTEEQEPWQALIPPAAEKRGVMEEYARLYRLARALKRSREDSVEDRWESLLGEYKKK*

>ProHo2_GNXQN_Nemertea_Cerebratulus_spec_TRINITY_DN37572_c0_g1_i6.p1

MMVYRALLALTLVLCLTQALPVKKANDLSKEIADVAKENTKIVKRSEELVQIGNHQNAPRTKKDDPTLKVKDEQNRDENGKIVIKENEIDKTKIVDSSETDNTPLETEAMEKAVMDLAKVADTPSDDDLEEKVKVVEEEKEIEKLPKEGEVEEDKETEQDEVFQPQDIFRLGAREEEVNDVNSDEVPDEESEVLDSSEEVPPEALMTANMEMAQDRPAGQDYQYDEDYNSLYSDPYRWLDRKKRDANRQKREALQGSEAGMSMAANEEDTKSRSKRDTEYPLSLNDLYNLYYKYKALKDEEEAEEEAMEPVYEVPEYQDEYGAEAPAAAYDYGVEQEMAPPSEEWEPEAVNNAIQEELQLEAMMQPSVEAEAPYYQADVLPEDIEEELEEEEELPAWEVPSSESAYYNPMEYAPVSRPMEDEYVPNKRTDPELLSLMPGIKRGDDFYPAYTEEQEPWQALIPPAAEKRGVMEEYARLYRLARALKRSREDSVEDRWESLLGEYKKK*

>ProHo2_GNXQN_Nemertea_Lineus_lacteus_comp26151_c0_seq1.p1

MMVYRALLALTLVLCLTQALPLKKANDLNKEIADVGKENTQKRLVKRSEETVIIGNHQNAPRVKKGDPAIELKNRDENGKIEVKENEVDKTEIAKSDVADNTPPDDEVMEKVVMDLAKVAEEVPEADENKDSDLEEKVKVVEEEKVMDEVPNNDEVAEEKEVEEDAVFQPQEIFNLGAQEKEINDVISSEVPDQESEVTESSDEVPPEALMTGNMEMAQEQPAADEYPYDEYNAQYSDPYQWLDRKKRNVNRLKRESALQGSEAGMSMVANEDTKSRSKRDTEYPLSLNDLYNLYYKYKEMEDEKQQEEELEPVYELPEYEENYAANPEYDNYGVEEELAPPSEEWEPAAVSDDIQRELELEAMMEPAMEEEAPYYLTDVDSLTPEEELEKEMEEEMEEQMEEDQLPAYDVPSEPVYYPMEYNPIAPQPDKRNQEMLSMMPGIKRADDFYPSYTEDREPWQALIPPAAEKRGVMEEYARLYRLARALKRSREDSVEERWESLLGDYDEKK*

>ProHo2_GNXQN_Nemertea_Lineus_longissimus_c47214_g2_i1.p1

MMVFRALLALTLVLCLTQALPLKKANDLSKEIADVAKDNTQKRLVKRSEETVIVGNHQNAPRPALELKNRDENGKIEVKENEVDKTEIAKSDVADDTPPDDEVMEKVVMDLAKVAEAVPETDNNSDLEEKVKVDEDEKVLDEVPNNDEVAKEKEVIEEDAVFQPQEIFNLGAQEKEINDVNSSELPDQESEVTESSEEVPPEELMSGNMEMAQEQPAADEYQYDEYNALYSDPYQWLDRKKRNVNRQKREAALQGSEAGMSMVANEDDTKSRSKRDTEYPLSLNDLYNLYYKYKEMEDEKQQEEELEPVYELPEYEENYAANPEYDNYGVEEELAPPSEEWEPAAVNDDIQQELELEAMMEPAMEEEAPYYLADDETPEEELEKEMEEELEEQMEAQQLPAYDVPSEPYYYPMEYNPIAPQPDKRNQEMLSMLPGIKRADDFYPSYTEDREPWQALIPPAAEKRGVMEEYARLYRLARALKRSREDSVEERWESLLGDLDEKKK*

>ProHo2_GNXQN_Nemertea_Lineus_longissimus_c47214_g2_i3.p1

MMVFRALLALTLVLCLTQALPLKKANDLSKEIADVAKDNTQKRLVKRSEETVIVGNHQNAPRPALELKNRDENGKIEVKENEVDKTEIAKSDVADDTPPDDEVMEKVVMDLAKVAEAVPETDNNSDLEEKVKVDEDEKVLDEVPNNDEVAKEKEVIEEDAVFQPQEIFNLGAQEKEINDVNSSELPDQESEVTESSEEVPPEELMSGNMEMAQEQPAADEYQYDEYNALYSDPYQWLDRKKRNVNRQKREAALQGSEAGMSMVANEDDTKSRSKRDTEYPLSLNDLYNLYYKYKEMEDEKQQEEELEPVYELPEYEENYAANPEYDNYGVEEELAPPSEEWEPAAVNDDIQQELELEAMMEPAMEEEAPYYLADDETPEEELEKEMEEELEEQMEAQQLPAYDVPSEPYYYPMEYNPIAPQPDKRNQEMLSMLPGIKRADDFYPSYTEDREPWQALIPPAAEKRGVMEEYARLYRLARALKRSREDSVEERWESLLGDLDEKKK*

>ProHo2_GNXQN_Nemertea_Lineus_ruber_Lvir.rna.tri.17462.1.p1

MMVYRALLALTLVLCLTQALPLKKANDLSKEIAEVAKDNTQKRLVKRSEETVIIGNHQNAPRVKKGDPAVELKNRDENGKIEVKENEIDKTEIAKSDVGDNTPPDDEVMEKVVMDLAKVAEAVPATDKNSNSDLEEKVKVVEEEKMLDEGPNNDDVAEEKEVEEDTVFQPQEIFNLAAQEKEINDVNSSEVPDQESEVTESSDEMPPTALMTGNMGMPQEKPAAEEYQYDDYNALYSDPYQWLDRKKRNANRQKREAVVQASEAGMSMVANEDDTKSRSKRDTEYPLSLNDLYNLYYKYKEMEDEKQQEEDLEPVYELPEYEENYAASPEYYNGVEEELAPPSEEWEPAAVNDDIQQELELEAMMEPQMEEEAPYYLADDETPEEELEKELEEEMEEEMEAQQLPAYDVPSEPVYYPMEYNPIAPQPDKRNQEMLSMMPGIKRADDFYPSYTEDQEPWQALIPPAAEKRGVMEEYARLYRLARALKRSREDSVEERWESLLGDYNEKKK*

>ProHo2_GNXQN_Nemertea_Malacobdella_grossa_comp8387_c0_seq1.p1

ETKEQRLVKRSAEVVIIGNHQNHERVKKSGPPHFLQEEDEEEADTKTIVHEPETKVDATRDESAKVKVETSHASEEAHVDETSEENRGEDIGDEAEPETEAKKEKS

>ProHo2_GNXQN_Nemertea_Nipponemertes_spec_TRINITY_DN34350_c1_g10_i1.p1

DHSKMTSQQHDMAKRSAERVLVGNHQNKPREKKSDPQMDVKEDETRDENAKVTISDSKTDEMKPEQVEATLTKVAEALHSQELQAAQAAEAEEAQDVQSPEVESTKPEQIPEAPESPSESSEEEDAAVGEEDQPTMDEILSQLHLPENSPFRNHRRRTDPREYRWNPLSRKRRDAVSTARESSMSMVKKRHAARAKRETPYPFLLDNVYKRQDE

>ProHo2_GNXQN_Nemertea_Notospermus_geniculatus_scaffold676.g17895.t1.p1

MVFRALLTLTLVLSLTQALPIKKANDLSQEIADVANENIQKRSVKRSQELLGVGNHQNKPRLKKADPAVELKNEQNRDENGKIEVVSENEIEKSDVPNSNSDESTPTETGTMEQAVVDLANVAKDVADDTADDSNDSDLEEKVKVIEEEKELEKIPKDGKIVEETETKEDEVLEPNEAFKVDEPEDVNSIEVPEEGEVDDSEEMSSEDVMNNIDVTPQRPAVTDYQYDDYNGLYADPYQWLDRKKRDADRKKRESALQGAEAGMSMVVNEDAKSRSKRDTQYPLSLRDLYNLYVKYQIIKDQDEPEEEDLQPVYELPEYEEDYDNSPEYDYGGEDVAPPSEDWEPDAVNEEIQKELAYEAMMQPALEAEAPYALENDLAEEDEDEDIDDDDEVPAWEVPAEPAYYPMEYVPISNPSEEDEIYVPDKRAELLSMMPGIKRAEKSFYPAYTEDQEPWQALIPPAAEKRGVMEEYARLYRLARALKRSRDEDALEGRWESLIGDYDEKKK*

>ProHo2_GNXQN_Nemertea_Notospermus_geniculatus_scaffold2758.g34781.t1.p1

MPPTAEVVKRSQELLGVGNHQNKPRLKKADPAVELKNEQNRDENGKIEVVSENEIEKSDVPNSDADESTPTETGAMEQAVVDLANVAKDVADDTADDSNDSDLEEKVKVIEEEKELEKIPXMVFTTKEYILKIETSVRLPGI*

>ProHo2_GNXQN_Nemertea_Paranemertes_peregrina_comp29979_c0_seq1.p1

MEGWISVLVLASLALVQALPLSGTVEHKNVKRSAEAVLIGNHQNHERVKKSGPPHFPEEEKKENSDAVTAEPEHAEHPDSKHDTTRDESAEVTVETNAASEEPRVDDETESESHETSAADESHESSKVSEEESKQETPSEEASETEAAELAELEEELPKLTQKERNEIDQLLSELNLPPSSPLYRDFKRSNSRVSRWHSSRSRRAHASALESSMSLKPRSVRSKRETWYPMMVQSKRDDGDYPEYVDYGEYDNGITEPAMPSYYPGEDYIPNEGYDVPMTEEEADELLKEIALKMAEEEAEKEAEEGYPAGLGRGDEYMTPDKRQYLSMTNGFKRSGTFYPYQEMEQSPWAAAIREDAEKRNIQDQYDRLYRLASALRGEERSQRQWEQ*

>ProHo2_GNXQN_Nemertea_Riseriellus_occultus_TRINITY_DN74194_c0_g1_i1.p1

SLLRSLAFFNGELAESAKENTQKRLVKRSEETVIIGNHQNAPRVKKGDPAVELKNRDENGKIEVKENEIDKTEIAKSDVADNTPPDDEVMEKVVMDLAKVAEAVPETDKNNNSDLEEKVKVVEEEKVLDEVPNNDEVAEEKEVEEDAVFQPQEIFNL

## Prohormone-3

>Prohormone3_Phoronida_Phoronis_psammophila_comp437865_c0_seq1.p1

PLRPGDGTLVSKQKSRGESCEDTSECSEEERLCCQQLVFFRRAKVRVCEKISRISKCILPDLND*

>Prohormone3_Phoronida_Phoronis_psammophila_comp86935_c0_seq1.p1

MQTSFYLSVIAVLISCVHAGWRRGPSQYYSGRTYSRPSPLDFLSSSRVRKCSGTDEMCFQNSQCCQGLICTTSTGFDDRLPIGVCAEPEPKSASQLLDGTLLSKKKKV

>Prohormone3_Phoronida_Phoronopsis_harmeri_c130453_g8_i1.p1

QSSNRRKLALCGCSTNVGDMCFQNNQCCQGLVCATPSGYEGGLSLYGVCEPLYRNYGLTRGDSCQDSSDCSTRDNLCCQEVRFFRRGTERMCDRITGISKCIKPSEDSNEISRGRK*

>Prohormone3_Phoronida_Phoronopsis_harmeri_c130453_g8_i2.p1

MQGQLYVLAAIACCVHSWGRPSSRFSASSYERPRSRPVSLSRVSSMSKLTRCTDVGDMCFQNNQCCQGLVCATPSGYEGGLSLYGVCEPLYRNYGLTRGDSCQDSSDCSTRDNLCCQEVRFFRRGTERMCDRITGISKCIKPSEDSNEISRGRK*

>Prohormone3_Phoronida_Phoronis_ijimai_(P._vancouverensis)_comp97001_c0_seq2.p1

MLARVCVLLVAVSSVYSWGRSPGSYYRPYNRHEKLERKCSARGDICFVDRNCCQGLACETSNNHRGRLSFGLCADPEPKSASQILESSKTATKGKGNTPVYNCTCFSSRHRGI*

>Prohormone3_Phoronida_Phoronis_ijimai_(P._vancouverensis)_comp97001_c0_seq1.p1

MLARVCVLLVAVSSVYSWGRSPGSYYRPYNRHEKLERKCSARGDICFVDRNCCQGLACETSNNHRGRLSFGLCADPEPKSASQILESSKTATKGKGEICSDSSECSPDPYGKVCCQDVTYFRRGSSTICGKVTRLSKCL*

>Prohormone3_Phoronida_Phoronis_australis_TRINITY_DN316501_c0_g4_i1.p1

AFYYKTNWVQFPVSESSLYSRVTLLQASSVITMLTRVCLLLIVVSSVYSWGRSPGSNYRPHNRHENLERKCSARGQICFDDRNCCQGLACETTNNYRGRLSYGSCTDPKPQSASQILETSDTGTKGRGEKCDDSSECSNTYYRLCCQTVRFFRAGSSRICDKVTGLSTCL*

>Prohormone3_Brachiopoda_Lingula_anatina_comp143633_c2_seq2.p1

MLPTAFHTGILLTVLSCVCAYRAMRFRDELPRVYQYPIQFPETDCSEEGSRCFQTSDCCEGSVCVTDNGLAGTCVPATPRRSGSYLCFTNTDCDPGFECTEVRSQSLRVCKVKGTRKEGEDCEDSSECRPGSCCQDINIFRRGTFRKCGPYYGISKCVRAKSDNSVVFRR*

>Prohormone3_Brachiopoda_Lingula_anatina_comp143633_c2_seq3.p1

MLPTAFHTGILLTVLSCVCAYRAMRFRDELPRVYQYPIQFPETDCSEEGSRCFQTSDCCEGSVCVTDNGLAGPYTFERGFCMELGERRKSGYKKNGEDCEDSSECRPGSCCQDINIFRRGTFRKCGPYYGISKCVRAKSDNSVVFRR*

>Prohormone3_Brachiopoda_Glottidia_pyramidata_comp48192_c0_seq1.p1

MSPTSLQAGILLSVLYCVCAYRPMMFRDGPARNYYNPIKFKEVECAEEGSRCFQTSECCTGSVCVTDNGLAGTCVSATPRRTDSDLCFTNTDCDPGFECIAVKARSLRVCRARGTRLVGETCEDSSECGPGTCCQDIKIFRRGTFRRCAPYYGISKCVQSKTENGVFYRRR*

>Prohormone3_Brachiopoda_Glottidia_pyramidata_comp48192_c0_seq3.p1

MSPTSLQAGILLSVLYCVCAYRPMMFRDGPARNYYNPIKFKEVECAEEGSRCFQTSECCTGSVCVTDNGLAGPHSYERGYCMELGSRLLEGGYKKNGETCEDSSECGPGTCCQDIKIFRRGTFRRCAPYYGISKCVQSKTENGVFYRRR*

>Prohormone3_Brachiopoda_Glottidia_pyramidata_comp48192_c0_seq16.p1

GHYGRGVLRTRKSAYLYFPQQFLSFRLTYKMSPTSLQAGILLSVLYCVCAYRPMMFRDGPARNYYNPIKFKEVECAEEGSRCFQTSECCTGSVCVTDNGLAGTCVSATPRRTDSDLCFTNTDCDPGFECIAVKARSLRVCRARGTRLVGK*

>Prohormone3_Brachiopoda_Novocrania_anomala_Nano.rna.tri2.40027.1.p1

MRSIYSVALLCALVGYVYTWGDMMRTDDTYESAWCIQENKLCIRSDSCCGDMVCIVKDKSYRYGTCQMDYTQERKKKGQKCVDSSDCADPYCCQWVPIFRRGPMRVCDKVYGGMTKCIPAKDYVRSNEVYYR*

>Prohormone3_Brachiopoda_Novocrania_anomala_comp70707_c0_seq2.p1

AITAGGYVYTWGDMMRTDDTYESAWCIQENKLCIRSDSCCGDMVCIVKDKSYRYGTCQMDYTQEKKEKS

>Prohormone3_Brachiopoda_Novocrania_anomala_comp70707_c0_seq1.p2

MRSIYSVALLCALVGYVYTWADMMRTDDTYESAWCIQENKLCIRSDSCCGDMVCIVKDKSYRYGTCQMDYTQEKKEKS

>Prohormone3_Nemertea_Paranemertes_peregrina_comp29433_c0_seq2_m.56303 complete

MKALLCLAVLTVVVGCVRGWGRILADNGLSYNSWIPARRSWGCGRDRDVCYQSSQCCHGFYCQRIDPNDITSIGECSYATKGATQGESCLDTTDCATGLCCQIIRTFRSGPLRMCSQITPFSKCLKANEGLGDE*

>Prohormone3_Nemertea_Paranemertes_peregrina_comp29433_c0_seq1_m.56298 complete

MKALLCLAVLTVVVGCVRGWGRILADNGLSYNSWIPARRSWGCGRDRDVCYQSSQCCHGFYCQRIDPNDRSVPGLCRAWRMEKTWTCLTDTDCRQGQTCKKQGPNSVQLGKCADTKVGRGKGESCLDTTDCATGLCCQIIRTFRSGPLRMCSQITPFSKCLKANEGLGDE*

>Prohormone3_Nemertea_Cerebratulus_marginatus_comp48972_c2_seq1_m.37624 5prime_partial

LWSPVLTFQNEDVVSMMKAVLCFGLLAILIGCVRGWGRILSGENNIGYNNWIPARRSWRCGGNKDLCWQNSQCCKGHYCATEDEGSIGECETYALNKGSRIGDPCEDNSECSMADGLCCQYVQVFRRKPKKMCHQISGLNKCIKSTGYGNNIIKK*

>Prohormone3_Nemertea_Cerebratulus_marginatus_comp551261_c0_seq1.p2

YCATEDEGKSGFCRADDQEQIPVCENDSDCPGWTKCTTVAQVGAVKLRMCKEPTDKKSKTGQPGDPCEDNS

>Prohormone3_Nemertea_Tubulanus_polymorphus_comp36515_c0_seq3_m.54785 5prime_partial

RRSWCGKQGDLCFQSSQCCKNFYCASIDSAEGKAVAFGKCRVDVQSKEKSGGSRGEDCEDSYDCDISQGLCCQYITQHRRRPSRRCDEITGYSKCIKPTKNTRHNRI*

>Prohormone3_Nemertea_Tubulanus_polymorphus_comp36515_c0_seq1_m.54775 complete

MNRIACLGVLSIVIVCVQGWNRTPTYSRHRSYFTAYGYPSRRSWCGKQGDLCFQSSQCCKNFYCASIDSAEGKQSPMGMCNPEPDICNTDADCDQREECKLTPVVEGHPALRMCTEKQEEVKAGRGEDCEDSYDCDISQGLCCQYITQHRRRPSRRCDEITGYSKCIKPTKNTRHNRI*

>Prohormone3_Nemertea_Cerebratulus_spec_TRINITY_DN29428_c0_g3_i4_m.47382 complete

MMKAVLCFGLLAILIGCVRGWGRILSGENSIGYNNWIPARRSWRCGGNRDLCWQNSQCCKGYYCATVDDGSIGECETYALNKGSRIGDPCEDNSDCSIADGLCCQYVQVFRRKPKKMCHQISGLNKCIKSTSYGNNIIKK*

>Prohormone3_Nemertea_Cerebratulus_spec_TRINITY_DN29428_c0_g3_i2_m.47377 complete

MMKAVLCFGLLAILIGCVRGWGRILSGENSIGYNNWIPARRSWRGLYRHGGWRTAPAKCGGNRDLCWQNSQCCKGYYCATVDDGSIGECETYALNKGSRIGDPCEDNSDCSIADGLCCQYVQVFRRKPKKMCHQISGLNKCIKSTSYGNNIIKK*

>Prohormone3_Nemertea_Cerebratulus_spec_TRINITY_DN29428_c0_g3_i3_m.47380 complete

MMKAVLCFGLLAILIGCVRGWGRILSGENSIGYNNWIPARRSWRCGGNRDLCWQNSQCCKGYYCATVDDGKSGFCRADDQEQVPVCENDSDCPGWTKCITVAQVGAVKLRMCKEPTDKKSKTGQPGDPCEDNSDCSIADGLCCQYVQVFRRKPKKMCHQISGLNKCIKSTSYGNNIIKK*

>Prohormone3_Nemertea_Cerebratulus_spec_TRINITY_DN29428_c0_g3_i1_m.47375 complete

MMKAVLCFGLLAILIGCVRGWGRILSGENSIGYNNWIPARRSWRGLYRHGGWRTAPAKCGGNRDLCWQNSQCCKGYYCATVDDGKSGFCRADDQEQVPVCENDSDCPGWTKCITVAQVGAVKLRMCKEPTDKKSKTGQPGDPCEDNSDCSIADGLCCQYVQVFRRKPKKMCHQISGLNKCIKSTSYGNNIIKK*

>Prohormone3_Nemertea_Lineus_lacteus_comp21130_c0_seq1_m.44836 complete

MMKAVLCFGLLAILIGCVRGWGRILSGENSVGYNSWIPARRSWRCGGNRDLCWQNSQCCKGYYCATVDDGKSGFCRAEDQEQIPVCETDGDCPGWTKCSTVAQVGAVKLRMCKESADTDAGAGKKAESGQPGDPCEDNSDCSIADGLCCQYVQVFRRKPKKMCHQISGLNKCIKSTGFGNNVIKK*

>Prohormone3_Nemertea_Lineus_lacteus_comp21130_c0_seq2_m.44843 complete

MMKAVLCFGLLAILIGCVRGWGRILSGENSVGYNSWIPARRSWRGLYRHGGWRTAPATKCGGNRDLCWQNSQCCKGYYCATVDDGKSGFCRAEDQEQIPVCETDGDCPGWTKCSTVAQVGAVKLRMCKESADTDAGAGKKAESGQPGDPCEDNSDCSIADGLCCQYVQVFRRKPKKMCHQISGLNKCIKSTGFGNNVIKK*

>Prohormone3_Nemertea_Lineus_ruber_Lvir.rna.tri.18939.1_m.38956 complete

MMKAVLCFGLLAILIGCVRGWGRILSGENSIGYNSWIPARRSWRGLYRHGGWRTAPAAKCGGSRDLCWQNSQCCKGYYCATVDDGKSGFCRADDQEQIPVCETDSDCPGWTKCSTVAQVGAVKMRMCKEPTDTDTDTGAGKKAESGQPGDPCEDNSDCSIADGLCCQYVQVFRRKPKKMCHQISGLNKCIKSTSFGNNVIKK*

>Prohormone3_Nemertea_Nipponemertes_spec_TRINITY_DN30024_c0_g3_i1_m.1594 5prime_partial

AHHPEDGQPLHSNFRRPLEPLDLNFKMRALLCLGVLTIVVTCVKGWGRILSDNSLGYNSWIPARRSWGCGRNRDICYQSSQCCRGYYCASIDPNDSKPGVCRAWRQEPDTCLGDEDCQAGHTCVPLGQVGSVRLGKCTKVKPSHRRGTMGDECTDSGDCADGLCCQLVRAFRRGPVKMCHQVSAFSKCIKAGQRVENMKDRR*

>Prohormone3_Nemertea_Notospermus_geniculatus_g2378.t1

MMKAVLCFGLLAILIGCVRGYGRILSGDNSIGYNNWIPARRSWRGLYRGGWRTAPAKCGGSRDLCWQNSQCCKGLYCATVDEGKSGICRAEDQEQIPVCDTDSDCPGWTKCSTVAEVGAVTLRMCKEPTDKKTPSVQPGDPCEDNSDCSLADGLCCQYVQVFRRKPRKMCHQISGINKCIKTNSYGNNVVKK

>Prohormone3_Nemertea_Notospermus_geniculatus_g30114.t1

MMKAVLCFGLLAILIGCVRGYGRILSGDNSIGYNNWIPARRSWRGLYRGGWRTATPKCGGSRDLCWQNSQCCKGLYCATVDEGKSGICRAEDQEQIPVCDTDSDCPGWTKCSTVAEVGAVTLRMCKEPTDKKTPSVQPGSIGECETYAYNKGSRIGDPCEDNSDCSLADGLCCQYVQVFRRKPRKMCHQISGINKCIKTNSYGNNVVKK

>Prohormone3_Nemertea_Baseodiscus_unicolor_TRINITY_DN86026_c0_g3_i3_m.217879 complete

MFTRAAEPSCDGGFPMTLCTFCIANMRTVLCLGILTVLIGCVRGWGRILSGENSIGYNNWIPVRRSYECAGPGGICYQNRDCCRNSYCAIVDGGNKGYCREETPFCKTDDDCDEWYECTTVARVGAVKLRKCQERSEQKDDKKRPARSSGGLKGDPCKDSSDCSLADGLCCQELPLFRRKPIVMCFHMSGRSKCIKESRFLGNEIVKRK*

>Prohormone3_Nemertea_Lineus_longissimus_c40405_g1_i1_m.58966 5prime_partial

CGGNRDLCWQNSQCCKGYYCATVDDGSIGECETYALNKGSRIGDVCEDNSDCSIADGLCCQYVQVFRRKPKKMCHQISGLNKCIKSTSFGNNIIKK*

>Prohormone3_Nemertea_Lineus_longissimus_c40405_g1_i3_m.58978 complete

MMKAVLCFGLLAILIGCVRGWGRILSGENSIGYNSWIPARRSWRCGGNRDLCWQNSQCCKGYYCATVDDGKSGFCRAEDQEQIPVCETDSDCPGWTKCSTVAQVGAVKLRMCKEPTDTAADTGTGKKAESGQPGDVCEDNSDCSIADGLCCQYVQVFRRKPKKMCHQISGLNKCIKSTSFGNNIIKK*

>Prohormone3_Nemertea_Lineus_longissimus_c40405_g1_i2_m.58972 complete

MMKAVLCFGLLAILIGCVRGWGRILSGENSIGYNSWIPARRSWRGLYRHGGWRTAPAKCGGNRDLCWQNSQCCKGYYCATVDDGKSGFCRAEDQEQIPVCETDSDCPGWTKCSTVAQVGAVKLRMCKEPTDTAADTGTGKKAESGQPGDVCEDNSDCSIADGLCCQYVQVFRRKPKKMCHQISGLNKCIKSTSFGNNIIKK*

## Prohormone-3-related

>unident_ProHo3_related_Brachiopoda_Laqueus_californicus_comp196645_c0_seq1.p1

IALLFLGLIAMTWAKSSSIRSLFKRARDDRGECSALGDKCSWTKWDRCCNELQCHKPHWTRKGTCQICKFVDSLCSKDAQCCPGLECSWGRCVRDD*

>unident_ProHo3_related_Nemertea_Nipponemertes_spec_TRINITY_DN6916_c0_g1_i1.p1

MRCCGDLRCHKPDMFKSGTCAVCKFSSSYCLHDYECCPGLTCSVGSCRGSPQL*

>unident_ProHo3_related_Nemertea_Nipponemertes_spec_Contig5821.p2

VTASPWKRDDDDNCNALGAPCANVAWDRCCGNLLCSKTNWFGKGRCSICKFRNAWCVKNKECCPGLHCHWGACKPEN*

>unident_ProHo3_related_Nemertea_Malacobdella_grossa_comp20240_c0_seq2.p1

PLRPGAEPIARQKRSCIPLGSPCGYTLFHRCCGQLRCHIVSIWRKGYCSICKFRGSICLRDYECCPGFTCNMSTCG*

>unident_ProHo3_related_Nemertea_Malacobdella_grossa_comp23764_c0_seq1.p1

MKSLCTMLTLLVVFVAVTQAYSIEKRSACKRLGQSCPYFVTSKCCGNLVCSKSGITGSGVCSVCKFRDSWCARDSQCCPGLEGKWFRKCK

>unident_ProHo3_related_Nemertea_Malacobdella_grossa_comp20240_c0_seq1.p1

AITAGRGPAIILWLLIILFGGSLTLAEPIARQKRSCIPLGSPCGYTLFHRCCGQLRCHIVSIWRKGYCSICKFRGSICLRDYECCPGFTCNMSTCG*

>unident_ProHo3_related_Nemertea_Riseriellus_occultus_TRINITY_DN44488_c0_g2_i1.p2

CSWTVWDRCCNELQCHKVHLTKKGTCQICKFLDSYCSKDSQCCPGLQCNWRGKCVRDD*

>unident_ProHo3_related_Nemertea_Riseriellus_occultus_TRINITY_DN21923_c1_g1_i1.p1

GMMKSALLLVILWTVAVTVLAMPSKRSSCNMLGQECSLTPWDKCCGDLMCSKTSYLGKGRCSVCIFKGAWCSRD

>unident_ProHo3_related_Nemertea_Riseriellus_occultus_TRINITY_DN21923_c0_g1_i1.p1

MMKSALFLVILWTVAATVLAMPGKRSSSCNLLGQECGLSPWDKCCGNLLCSKTGYISKGRCSVCKFTDAWCSRDSQ

>unident_ProHo3_related_Nemertea_Riseriellus_occultus_TRINITY_DN38880_c0_g2_i1.p2

FLGLVALTWAKSTGSSSFRSLFETKRDNGYVCEGEGKGLGEECSWTAWDRCCDDLQCSKKHLTRKGTCRFCKANDSYCTKDSQCCSGECNWRGKCIPA*

>unident_ProHo3_related_Nemertea_Riseriellus_occultus_TRINITY_DN38880_c0_g2_i2.p1

VNATSPRNIRTIKVFILVYFLSMNALLKNVLEPRWEASVLSEECSWTAWDRCCDDLQCSKKHLTRKGTCRFCKANDSYCTKDSQCCSGECNWRGKCIPA*

>unident_ProHo3_related_Nemertea_Cerebratulus_spec_TRINITY_DN26010_c0_g1_i1.p1

MKTIIALVLLGLVAVAWAESSSIQNLFRRKRARDDKGECNAFREKCSWTAWDRCCNNLQCHKTSLTRKGTCQICKFVDSYCTKDSQCCKGLQCNWRGKCVIDD*

>unident_ProHo3_related_Nemertea_Lineus_ruber_Lvir.rna.tri.1270.1.p2

MKTLIALLFLGVVAVAWAKSAGSSSAHSIFKRGRDKRGECNALGDKCSWTAWDRCCNELQCHKVHLTKKGTCQICKFLDSYCSKDSQCCPGLQCNWRGKCVRDD*

>unident_ProHo3_related_Nemertea_Lineus_lacteus_comp23803_c0_seq1.p2

MKTLIALMFLALVAVTWARSTGTSDVHSIFKRGRDKRGTCNALGAKCSWTVWDRCCNELQCHKVHLTRKGTCQICKFLDSYCSKDSQCCPGLQCNWRGKCVRDT*

>unident_ProHo3_related_Nemertea_Lineus_lacteus_comp23803_c0_seq1.p1

MKTLIALLFLGLVALTWAKSTSNRNLRSLFKTKRDNNYVCKGDGNGYGEDCSWTAWDRCCDDLQCSKKHLTRKGTCKFCKETDSYCSKDSQCCSGECNWRGKCIDV*

>unident_ProHo3_related_Nemertea_Lineus_ruber_Lvir.rna.tri.965.1.p1

MKILIALMFLGLVALTWAKSTGSSSRNFLSLFKRDGSYVCKGDGNALDEECSWTAWDRCCDELQCSKKELIGKGTCKICKETDSYCTKDSQCCSGECNWRGKCIDV*

>unident_ProHo3_related_Nemertea_Paranemertes_peregrina_comp24621_c0_seq1.p1

MRCSLVCMTLILVVATSHASVVKRGSCNNLGSECGYNAWDKCCGNLHCSKSGMISKGVCSVCKFRNAWCLNDGQCCPGLQCNWYRKCKPTS*

>unident_ProHo3_related_Nemertea_Paranemertes_peregrina_comp29800_c0_seq1.p1

MEHVKDRSTIIVWLLIILFGGLCAAEPIRLSRQKRGCISLGSQCGYTPFSRCCGELRCHIISMWRRGFCSVCKFEGSLCLRSNECCPQHSCRMGHCRMVSQTQSLDA*

>unident_ProHo3_related_Nemertea_Lineus_longissimus_c33402_g1_i1_m.27829 complete

MKTLILLMFLGLIAITCAKSTGSSNVHSIFKRGECNALGEACPWTFWDRCCNELQCHKIHMTEAGTCQICKFIDSYCSKDSQCCPGLQCNWLGKCVRDT*

>unident_ProHo3_related_Nemertea_Lineus_longissimus_c36235_g1_i1.p1

MKTLIALTFLGIVALTTAKSTGSNSFRSLFETKRDNDYVCKGEGRPFGAECSWVPWDRCCDHLLCSKTHLNSKGECAFCKALDSLCSWDSECCSGECNWKRRCIEA*

>unident_ProHo3_related_Nemertea_Lineus_longissimus_c33527_g1_i1.p1

MLAMDNLCKTATMQLLTVLSIIYISTTNATPVKRGEGDDVQTGEILPAGSNHCYDLGDSCLGTFIKPCCGWLYCGKWDLFKRGTCVTCKPSGQWCAKDRECCSGHCGSTWGCS*

>unident_ProHo3_related_Nemertea_Cephalothrix_hongkongiensis_comp38471_c0_seq1.p1

MKFFCVALIVLVGCIYTEAFLENTIQLVKKAVEEVQKRELAEGAEEEIIEKRGECTRTEGHKCDGSVFMRCCGDLQCDKPGLTGQGRCRRCVSKNNFCWKDDECCSGDCSGLLKRKCK*

>unident_ProHo3_related_Nemertea_Baseodiscus_unicolor_TRINITY_DN95708_c0_g3_i3.p1

MREIISLFVAVVLLVPTDGVPPPSNENAYKALLRQRRDCGDIGDSCSWTIVDRCCGQLQCHKTKMFRAGSCQICKFRDSYCYKDSQCCPGLECSWRGKCVDED*

>unident_ProHo3_related_Nemertea_Baseodiscus_unicolor_TRINITY_DN107869_c0_g4_i1.p1

MGFKIGAFVALLVAMLIFLMGEAVPSTNGDKALLRQRRACKKSGDWCSWTVFSRCCGNLQCDKPYLLRAGVCRECKRKGSYCLRDSQCCFGLQCDWRKICVKKD*

>unident_ProHo3_related_Nemertea_Tubulanus_polymorphus_comp40897_c0_seq15.p1

MKRFLCLLVIVGFVCVDQTESGCSRHLNQECTIFSHCCDPLSCNKPYFFRSGKCAACRRKNSYCFRNKQCCSGYCRWWFKCN*

>unident_ProHo3_related_Nemertea_Tubulanus_polymorphus_comp37651_c0_seq1.p1

MKRFLYLLVIVGFVSVVFADTDASDADQTERHSDLQNERLFELLRKRLFTKCRDVNEECTLFHYCCDPFSCNKPNLFKSGRCSSCRKNGSYCIKKSQCCSGNCSWLKCK*

>unident_ProHo3_related_Nemertea_Tubulanus_polymorphus_comp40003_c0_seq1.p1

EEEQDKRNGGGEVEVDEVKEDEPDKRNEEEAEEEEEELIRMVGAKEEDKRNEFPRRPMDLEETGDNINIKRYLESAQRRSEDGDEDDDDAIDEVESDEPGDDEKRSAEALDVAAILSRRGWNPFKCRGLNKPCKGTAFDRCCDPMRCDKPNRFSDGSCQLCKSENTLCARDKECCLGLKCHVLKCVRRT*

## Prokineticin/Astakine

>prokineticin_related_Phoronida_Phoronopsis_harmeri_c33912_g1_i1.p1

GCCLSTFRPRGKRDSHFPGTCQPLGIEGSKCFVNQQSMSLKYDVFYDSCPCESGLKCQDTGHVDMPLGPIGFC

>prokineticin_related_Phoronida_Phoronopsis_harmeri_c147743_g1_i1.p1

MKTLLFSLAVLAACTCLCSGLRTCKENRECQPSECCLQQVISKSGFHVKTIKTCRPKAGPSQRCRPQPRYYGWDEMEDYYYKQCPCANEALTCRFDPSFRDANDEDYQYFASLMDPSSLSSLMDSQYGRCLPPVTKKN*

>prokineticin_related_Phoronida_Phoronis_psammophila_comp63816_c0_seq2.p1

MVMTKSTGFLIVWALYAISCGAIGEKPCENNIDCGANECCLQRIQRPGKEITKTCQPKAGHTQRCSPYPKYYNIEGKQDYYYNNCPCENEAMTCRYSHHFDDAGAEDYEYYSMMMGPEGVNQVMDL

>prokineticin_related_Phoronida_Phoronis_australis_TRINITY_DN303636_c4_g5_i1.p1

MRVTLIVLSTWLLVCDTFARPCENNRDCGSNECCLQRMEGKKLLKTCQPRARQSQRCTPKPKYHGIPGRQDYFYSHCPCEHEAMVCRFPGTFHSLTSDVETQDYYFYSSLMGPQDVHQVMDKLHGRCLVAAGEKKHASQSFLKYPAQIIEE*

>prokineticin_related_Phoronida_Phoronis_ijimai_(P._vancouverensis)_comp96275_c0_seq2.p1

MRLALIVVGTLLLVCDAFARPCENNSDCGSNECCLQRMEGKRLLKTCQPRAHRSQRCTSKPKYHGIPGMQDYFYSHCPCEHEAMVCRFPGTYHSLTSDVETQDYYYYSSLMGPQDVVQVMDKIHGRCLVPAAEKKQAAQSFIKHSREAFEE*

>prokineticin_related_Phoronida_Phoronis_ijimai_(P._vancouverensis)_comp96275_c0_seq5.p1

MRLALIVVGTLLLVCDAFARPCENNSDCGSNECCLQRMEGKRLLKTCQPRAHRSQRCTSKPKYHGIPGMQDYFYSHCPCEHEAMVCRFPGTYHSLTSDVETQDYYYYSSLMGPQDVVQVMDKIHGRCLVPAAEKKHAAQSFIKHSRETSEE*

>prokineticin_related_Brachiopoda_Terebratalia_transversa_Ttra.rna.tri.3592.1.p1

MENLALMLIMVAMICTSGAKPSSDLARRCNTNSDCNSGIAAGITACCVDDIDRKGKRSTYTYTGQCALLGKIDDACLVNNASPPLDSDAWPGVSFQMECPCKDGLRCQGNGNVIIPQGETGHCKI*

>prokineticin_related_Brachiopoda_Glottidia_pyramidata_comp17226_c0_seq1.p1

AITAGCEANECCVTQLQMKGKRQLILPSMWGTCRKMGVENTACYVNAAKDRSALHVMRCPCAEGFICTGTVQIEVPVGEIGICKKI*

>prokineticin_related_Brachiopoda_Hemithris_psittacea_comp21207_c0_seq1.p1

MWVKIVLMMVAVVVCLVSAGSVTSNRRCKTSRDCRWGHCCVGDSTRKGKRSVSGINPINRRCERLGVYNSPCLVNNNNMRITPGSVFEEHCPCFTFWRISCVGSGNITPPQGERGMCGWGGFRKHTRRSKSRDK*

>prokineticin_related_Brachiopoda_Hemithris_psittacea_comp27335_c0_seq4.p1

MWAKISLTIFGTMCLVFIVASSACKTSGDCEWGQCCVGDSTRKGKRSISGINPINRRCEQLGGYKSSCLVNNNNIPVTSDSVFENHCPCFSSPFWQLTCIGNGDRTIPLGENGSCGWGPIKRGK*

>prokineticin_related_Nemertea_Malacobdella_grossa_comp25982_c0_seq1_m.45564 5prime_partial

MFRLIVISALLTVCFAGFHGLGSRFERCSNDGDCLAGQCCAKYGLTGLVKFCRRLGYIGHPCGKSMTGVCPCAPELTCVAHPWWQVVKVGTGKCERREIQVTKPLPEEGSGDIDDGFLF*

>prokineticin_related_Nemertea_Nipponemertes_spec_TRINITY_DN21147_c0_g1_i2_m.18350 5prime_partial

NSGECLAGECCARYGVTGWVHFCRPLSHENQPCGDSFIGVCPCERGLACVPYGFFDFFKVGDGTCRRTNNLDVSVPDEGSGDEI*

>prokineticin_related_Nemertea_Nipponemertes_spec_TRINITY_DN4565_c0_g1_i1_m.60744 5prime_partial

QKLVIVLSLLVFLLNQAHAGKCLYDGECAQGECCASILISGYFKFCRPLGLEDQPCGKSISGMCGCHEGLVCDEHKGWTAIFKTGTGICRPEEVEGSGGVEQ*

>prokineticin_related_Nemertea_Tubulanus_polymorphus_comp37493_c0_seq1_m.63479 complete

MKVIALTLTVILLVQGVSAWFCSDDNDCRPSECCVKSLFIRYCSSRGNDGDRCYNYDTPLTRTCPCNEGLVCKVNRGLDTSTNNNFRRGRCTVEETGSGYVEE*

>prokineticin_related_Nemertea_Tubulanus_polymorphus_comp39340_c0_seq1.p1

MIATSVKLFAFLFLIELAAGFNPCFSHNDCESNECCKADSFMSSCEWKGMNGERCYDVASTGGVSVHCPCGSGLTCVVLDPSPSNKRRAGFCMRTK*

>prokineticin_related_Nemertea_Tubulanus_polymorphus_comp31825_c0_seq1_m.28160 5prime_partial

MKSAIIIVIFCLTGIVSGWYCKKTEDCSSDECCAKVFFIGRCRKLSIEGQSCSSIDGSLGIWDTCHCRPGLECVPHTSMAGFKPGKGTCQTPEVEGSGLLED*

>prokineticin_related_Nemertea_Cephalothrix_hongkongiensis_comp37299_c1_seq2.p1

DSDCGRWECCAPVGSTGIFGNWCKDLARPGGNCGKSYHGLCPCTQGYYCKPHEKDSIFNIGEGKCIEEDLGSGDAEE*

>prokineticin_related_Nemertea_Cephalothrix_hongkongiensis_comp37299_c1_seq1.p1

SQRPQSESSLHPHAEIDCGRWECCAPVGSTGIFGNWCKDLARPGGNCGKSYHGLCPCTQGYYCKPHEKDSIFNIGEGKCIEEDLGSGDAEE*

>prokineticin_related_Nemertea_Cephalothrix_hongkongiensis_comp33462_c0_seq2_m.31664 internal

MRYIVILICLITVFGANSGMILRRGFGDCKSDSHCGNGFCCVRIGQEALFGHMCKPLGKTGEKCGKSYFGLCHCSEGYSCKPYYKTSINDFAD

>prokineticin_related_Nemertea_Cephalothrix_hongkongiensis_comp33462_c0_seq3_m.31666 internal

KNNYVSHGSIVLSSVLLTDFLVRKCLTTVFGANSGMILRRGFGDCKSDSHCGNGFCCVRIGQEALFGHMCKPLGKTGEKCGKSYFGLCHCSEGYSCKPYYKTSINDFAD

>prokineticin_related_Nemertea_Cephalothrix_hongkongiensis_comp351291_c0_seq1_m.113552 internal

LKSFSIETCELDSACGPDYCCAPTVKGSPIRVCKRLQGIGHKCHPASILVPSPFPRVHHICPCSTDLECE

## PSLamide

>PSLamide_c28466_g1_i1|m.18377 Nemertea Lineus_longissimus 3prime_partial

MGVYDKRWLVSWHNMVLAALYCLFLATTAVTSSEVSNGENWDTVLSDSEMANSPAESQENSIEDSLNLTNNDYDMDIENDSAEFPIGGANDESSAAKRFSLTAPSLGKRFSLTAPSLGKRFSLTAPSLGKRFSLVTPSLGKRFSLTAPSLGKRFNLVSPSLGKRLRHALVSPAFGKRDEEEYADRLDSDDEPRDESKRYSLVTPSLGKKMSGIVAAALGKRDAEKRFSLTAPSLGKRFSLTAPSLGKRFSLTAPSLGKRFSLTAPSLGKRFSLT

>PSLamide_Nemertea_Lineus_longissimus_c28466_g2_i1.p1

SLGKRFSLTAPSLGKRFSLTSPSLGKRFSLTAPSLGKRFSLTSPSLGKRFSLTAPSLGKRFSLTSPSLGKRFSLTAPSLGKRFSLTSPSLGKRFSLTSPSLGKRFSLTSPSLGKRFSLTAPSLGKRWSLTSPSLGKKSFGLTSPSLGKRFSLTAPSLGKRFSLTAPSLGKKNAEREIDDLADELSRNKRNADDKLRHSLIRRSSGWRLPSRQHLFRAGALGKRASQ*

>PSLamide_Nemertea_Lineus_lacteus_comp8823_c0_seq2.p2

MTGLVPAPPRTTPHEKRFSLTSPSLGKRFSLTNPSLGKRFSLTSPSLGKRFSLTSPSLGKRFSLTAPSL

>PSLamide_Nemertea_Lineus_lacteus_comp35850_c0_seq1.p1

PSVGKRFNLVTPSLGKRFSLTAPSLGKRYNLVSPSLGKRLRHALISPAFGKRDEEEYMDGLDSDNEAGDENKRYSLVTPSLGKKMSGIVAAALGKHKDRKST

>PSLamide_Nemertea_Lineus_lacteus_comp33852_c0_seq1.p1

MGVYNKRCLVCWHNMVLAALYCLCLATMAVTSSEVSNGENWDNVLSDSEMANAPSESRGNSIEDSLSLTNNDYDMDIENDSAEFPIGGANAESSAAKRFSLTSPSLGKRFSLTSPSLGKRFSLTAPSLGKRFNLV

>PSLamide_Nemertea_Lineus_lacteus_comp8823_c0_seq1.p1

GKRFSLTAPSLGKRFSLTSPSLGKRFSLTSPSLGKRFSLTSPSLGKRFSLTAPSLGKRWGLTSPSLGKKSFGLTSPSLGKRFSLTAPSLGKRFSLTAPSLGKKNVEREIDDLADELSRNKRNANDKIRHSEIRRSSGWRFPLRRHLFRAGALGKRASK*

>PSLamide_Nemertea_Baseodiscus_unicolor_TRINITY_DN25885_c0_g1_i1.p1

MQTQPSLGKRYNLFAPSLGKRFRLNPPSLGKRFNFKPPSLGKRFNLQLAPSLGKRLRHSLFAPALGKRSYSGGDDQDKRYRMVSPSLGKKFSGLVAAALGKRDDEKRFSVPSPPSLGKRYQFIPPSLGKRFSFMPPSLGKRFNFMAPSLGKRFSGLLTPSLGKRFIIGAPSLGKKSASTELEEN

>PSLamide_Nemertea_Lineus_ruber_Lvir.rna.tri.81589.1.p1

PSLGKRFSLTSPSLGKRWSLTSPSLGKKSFGLTSPSLGKRFSLTSPSLGKRFSLTAPSLGKKNAEEEIDDLADELSRNKRNANDRLRHSAFRRSSSWRFPSRQHLFRAGALGKRASL*

>PSLamide_Nemertea_Lineus_ruber_Lvir.rna.tri.82195.1.p1

MGEYNKHWMVGWHNMVLAALYCLFLATTAVTSSEASNGENWDTVLPDSEMTNPPGETRENSIENSIENSLNLINNDYDMDIENDSAEFPLGGADDESPAAKRFSLTSPSLGKRFSLTSPSLGKRFSLTSPSLGKRFNLVTPSLGKRFSLTSPSLGKRFNLVSPSLGKRLRHALVSPAFGKRDYEEYMDGPDSDDDSEESKRYSLVTPSLGKKMSGIVAAALGKRDAEKRFSLTSPSLGKRFSL

>PSLamide_Nemertea_Cerebratulus_spec_TRINITY_DN28660_c1_g1_i1.p1

SLGKRFSLTSPSLGKRFSLTSPSLGKRFSLTSPSLGKRFSLTSPSLGKRFSLTSPSLGKRFSLTSPSLGKRFSLTSPSLGKRFSLTSPSLGKKSFGLTSPSLGKRFSLTSPSLGKRFSLTSPSLGKKSAEEEIDDLADELSRNKRHADVIKRHEVKRSSAWLRKTLRAPLRQHLFRAGALGKRSAGQ*

>PSLamide_Nemertea_Cerebratulus_spec_TRINITY_DN26369_c0_g1_i1.p1

MGMCNKHWLVGWRNMVLAAIYCLFLASTAVTSSESNDGNWDNVLSESDVINSPSDSGENSIEDSLNLRNNDYMDIENDSAELPIGGADDESSAAKRFSLTSPSLGKRFSLTSPSLGKRFSLTSPSLGKRFNLVSPSLGKRFSLTSPSLGKRFNLVSPSLGKRLRHALVSPAFGKRAAEEYEEMDDDIPLDAESKRFNLMAPSLGKKMSGIVAAALGKRDGDKRFSLTSPSLGKRFSLTSPSLGKRFSLTSPSLGKRFSLTSPSLGKRFSLTSPSLGKRFSLTSPSLGKRFSLTSP

>PSLamide_Nemertea_Notospermus_geniculatus_scaffold1800.g29544.t1.p1

MSNKCWLVGWQKMSLAAVYCIFLASIAVTSSEASNDRSWDKELPENDVINSPSDTSDDNSIEDALNFRNDYDMDIENDSAELPNSADEDSSVEKRFSLTAPSLGKRFSLTAPSLGKRFSLTAPSLGKRFSLTAPSLGKRFSLTAPSLGKRFSLTAPSLGKRYNLVSPSLGKRLRHALVSPALGKRGDEDDGDFDDVSKRYNLVSPSLGKKMSGIVAAALGKRDEEKRFSLTAPSLGKRFSLTAPSLGKRFSLTAPSLGKRFSLTAPSLGKRFSLTAPSLGKRFSLTAPSLGKRFSLTAPSLGKRFSLTAPSLGKRFSLTAPSLGKRFSLTAPSLGKRFSLTAPSLGKRAFGLTAPSLGKRFSLTAPSLGKRFSLTAPSLGKKSAEETIDDLSKELSRKKREAHDKLRHLAVKRSSSWRFPLRQHLFRAGALGKRSE*

## PTTH

>PTTH_Phoronida_Phoronis_australis_TRINITY_DN276539_c0_g2_i1.p1

MCLLTLKRFQAFVVITLFIIFCEVTCAPVRPKMTHECQKLEESDLKKLMGSAFDISSMAHVLPDVTATRNQNISTETESTDESSIRQRRDTEAQNSRHLSGRRNPGRRRRKHRRRLSWKCKCKQQWTDLGDQYYPRYVKEKICEQTKCWRGNYRCKARTYVIKVLKQNMDGCHDKELPTVLRTGWQFVKVHVRVGCECVSKKDWS

>PTTH_Phoronida_Phoronis_psammophila_comp65283_c0_seq1.p1

MITLCSIVCLQVLAAIVVFVILPEARGTPTKPKMTVQCPKVRESDLQKLMGAEFDASAMAHTLPATKTVDEWTNVQTNATAHRILPTDRRTRLRRSTRKDGRRHPLHKGRRRKHRGKPAWRCKCRTVWTDLGDQYYPRFVKEEVCDQSKCWRKNHRCRPRRYTIQVLKHNMDNCYDTRLPEELRTGWHFVQVHVRVGCDCVPKKRHVRL

>PTTH_Phoronida_Phoronopsis_harmeri_c78809_g1_i5.p1

LTVWQDMDDGIFPRSIKTGRCHGRCSLSSRSCTPKYYFIKVMKRLVGACVPLPSIDENQVYEELWELKNIPVVVCCS

>PTTH_Phoronida_Phoronis_ijimai_(P._vancouverensis)_comp85230

MQGVQTVISLLVVQSILSGQCVSKRARRRLTGKRSISLSGIQILNQTVPCSPVDSDDSNYVNTTNGTRLRRLALDDDHMMEVLRQKGGAFLPSYMATNNPVRAKKDFMKKFRSRDRQRDPSFHPRPFDENDDNYRAKEMLLKYLKKRKFHRRTRSSLQKVVEDVVSNDRLDVERSKRTSTINNPSINGHTDVGYCQGRGAKTTDGYVHMCTVCTATTTLPPNRFPRSIFEEVCDATDQQCLFTGPTAHGACKQRTLTVNLLRKKEGACAIAVVEGQQVLVDEWELYTHPLRVGCECMIDSRSMFAPLLGTRSPIRRR

>PTTH_Phoronida_Phoronis_ijimai_(P._vancouverensis)_comp93767_c0_seq8.p1

PLRPGTATVSRNQNISTETESTDDSLIRQRRDTEAKAQNSRHLSGKRKPGRRRRKHRHRLSWKCKCKQQWTDLGDQYYPRYVKEKICEQSKCWRGNYRCKARTYVIKVLKQNTDGCDDKELPADLRTGWQFVKVHVRVGCECVAKKDRA

>PTTH_Phoronida_Phoronis_ijimai_(P._vancouverensis)_comp93767_c0_seq1.p1

PLRPGMGSAFDISSMAHVLPTATVSRNQNISTETESTDDSLIRQRRDTEAKAQNSRHLSGKRKPGRRRRKHRHRLSWKCKCKQQWTDLGDQYYPRYVKEKICEQSKCWRGNYRCKARTYVIKVLKQNTDGCDDKELPADLRTGWQFVKVHVRVGCECVAKKDRA

>PTTH_Phoronida_Phoronis_ijimai_(P._vancouverensis)_comp40176_c0_seq1.p1

SKNLDHQRFPRNTSKRRVRKRHKHKKPQLIMRIRCSDKESKTDDYKHLMGPVFDIRYLSFEKPQELVDSEQNRSVANNNISMVRSDNVGDINTDFDETHEAVMDKSHESASGRLLRHLRSTNKRKTLAKPWDCESRVIWRDLGDNYLPRFLRSVQCSSDRCWNRLFYCVPRVFTVPVFKRKSLSCKEQIVRNAERKRTGKKRKHKLEEQWELSNTNVTFCCECSQRPGHLQT

>PTTH_Brachiopoda_Hemithris_psittacea_comp26295_c0_seq1.p1

MWLTACLIFCIITFWRPSDMAPSIGNRDSTTRSTRGNDTGEIMLNDHRGANRTNSKRHHRHRKIIKTDCAQTNPSKLKKLLGPSFNARYMSINAPNEDSSTLDGTIGMSGRARASNDQPITTGSNVTVPPFEVASGYRRDLPSGYFPWLASQKLLDNISRKKRSSKERFLLRIKRARVSPKKGKPASPMTGGKTKFPWQCDKQIIWVDLGDNHFPRFLRTIKCTTDKCWFGHFRCVPRAFTVNVLRRKTDKCKPVYGRNGRGAKPIRYEHEWVYEERSVTFCCDCAEL

>PTTH_Brachiopoda_Terebratalia_transversa_Ttra.rna.tri.14659.1.p1

MWVKVLTITILAMMRSPSVKVAPASNIDNKPSEETIDTHEAAPHNVKSIYHSTSNSYTNTHVTIQKYKRSVDSKNDTFNDDIMVNDHRKNRKRGHRKKSKTIIKTECKSESKDKLRQLLGPAFNARYMAIDKPDDEPGTIANIGVAGDHNISSDEGTRFDSSTHPPFEVSADYKRDLPKNFPFPGYVEKTSLGDSMVTPMSSSKRFLLRVKRARIDPRKKAAKAPSSSGKKKSFPWQCNSKIIWQDLGDDHFPRFLRTIKCITKDCWFGHFQCVPRAFTLNVLRRKTDKCVPVYGRKHGQKTGTIIRYEHEWIFEERSVTFCCDCAAV

>PTTH_Brachiopoda_Novocrania_anomala_Nano.rna.tri2.92882.1.p1

MGQSTFYAYALWDYMLWTLFCFSSLPATREVTVGDRTFVVTRRCQPLERTELRRRLGRDLRYADRTPTVLTQEETSVFDLKLQKLSVSDADVESQRLQHTSVDISTLKHSLSSSHRQKRIVKIRHRFIRKSSIDKYSILFNSFKSVGARRGRKRRLRHKRDTPSPWTCEMETNWRRMSPGVYPRYILSGQCVNRRCMGSHFYECQPVQRRLKLLRHTPGQCNPIPRTEFNSTYEEAWETISVNVTVFCQCSRRQRLGRI

>PTTH_Brachiopoda_Novocrania_anomala_Nano.rna.tri2.141365.1.p1

MHLVYSILAATALLGTLPCFTTAPVETKTDASVNETMEYKLRVLDDIVSVEKARNQNKNALKVRKVRKNTKTRAKSDKYPEVAERTKKGDNVWDGVTGNDKIRDTLTERMQKDESDATSGRELASGLTSVKIECEMTTTETLRRILGPAFNARYMSVERPSPTDHSAVLDTTGNPRLQDQPFPWRHIPRTHHGSFVVKEGFKRDLPKGYFKPIHRRNNRRTKRSATEKFLRRIKRARSRARARNHGARRSEDATELPWQCQSRVTWRDLGADHFPRFLRTVECTSDQCWFGHYSCIPRAFTVTVLRRRTDACVPVYATEDGTGPAIRYEHEWVFEENSVTFCCDCANE

>PTTH_Brachiopoda_Lingula_anatina_comp148377_c0_seq3.p1

MISPYFRNNSQVNPQILWVVVGLITIMSVRIIKTAPLQEDPGVETRSLASLSGQTRRGQILENEKHLRTNANKNASAHMTAEDLTAIQADDASVARAAYHTSRVSNRDNDRHMPSVKIECEPTSTETLRQMLGPAFNARYMSISRPYLRPSVGMSLAGGMVGDEPIEIRRRHDPANHHGQFRVPEGFERDLPSDYVSSQHRRHHHGQHRRSASERFLSRVKRAKTSPRRPGGGRGGSPPWQCDSRIVWDDLGSDHFPRFLRTVECTQQDCWFGHYSCVPRAFTVNVLRRKTDACVPVYGNPTGQDDRREIIRYEHEWIFEERSVTFCCDCSAE

>PTTH_Brachiopoda_Lingula_anatina_comp125978_c0_seq4.p1

VYTNTTQMAGAAAETLICVAETLSENLREGVATAMVLFSLSSYVSAAPRRWKCDVVEGVQTGSRVPEMGAIIGASLSTTNFSARSQIALRRSGEEQFEPERPSMRPRWMERQVRAERGVGNLAPCECMSETHFRDLGQGFDPRFIRDVLCKGKCSNEHGVCVPRNRTVTVLKRLPQTWHLECENGFRPVGLTGGWGKTKVMVTTHCDCVL

>PTTH_Brachiopoda_Laqueus_californicus_comp25928_c0_seq1.p1

NTKRGHKKRNKSIIKTECHSESADKLRKLLGPAFNARYMAIDKPDDEPETIANIGVASDPNINSDEANKVDSSTHPPFKVDADYKRDLPKNFPFPGYAEDTPSLDHDIVTPMTSSEQFLIRVKRARINPRKKAVKSPASPVKKKTFPWQCNSKIVWQDLGDDHFPRFLRTIQCTTKDCWFGHFRCMPRAFTLNVLRRKTDKCVPVYDKKHGKKTGTIIRYEHEWIFEERSVTFCCDCAAV

>PTTH_Nemertea_Lineus_longissimus_c49535_g1_i1_m.155992

IRPIFLRGPHVCTYIMIALAFGVATPKRWNNIDIGLSSKSNRTLMNYKDRQCLPRSEDDLVRLMDSHFMSNKMATKLPRGPGRELGLTSKPTSRAGRTSRHIYNDDRGPTEGLRTVSEDSLYSHETVDFKYPVGDKDSPTINLQDFTVSLPSQIDEVDNLKRVSSSDSAERGGGDERTTQKFTKRRLLQRTRRKATPPRERRRANRHKGRDRTGIGKTNLEEPPWHCELKTEWVRMPTGYFPPYVQTGRCEQSNCMFGMYQCKPKHYVIRVLRRNPEQCNPLPVLGVNTTYEEAWDFAKYKITICCECSRSRQFGRQKSRRKNKNKNKNKNKGFAGIGVE

>PTTH_Nemertea_Paranemertes_peregrina_comp167071_c0_seq1_m.171933

MEKKQIRPNITWGWFYVRVLMMIVLAVGASAASRKRNGYVVDETKNSTSLILFHRKCLSKPEVELRKQLDASPGDEMMADDDNQARQLRSIVNEVGFVDEMDSIENIGHIADIHQAQADAVSAQYSRKLFGGVSANADGSDDYQSDDDLVDDAPRVTKVLTEVELSSKRRRRLERQRLRTLKRKRRRKRKKSRKKDIDDFPPWHCEMKTEWLRMPDGYFPQYVQTGQCSRSKCMFGMYKCRAKRYVIRVLRKNPL

>PTTH_Nemertea_Tubulanus_polymorphus_comp31589_c0_seq1_m.27280

MKINFSPITWFYIAIAAVSGVTRPTVRKSCRSYKVRGETEDDLILLMKEKGADYIPNMMAMSEKSSYRQFTNLIPTLTKYRVPLYKKQIFDSLNDSDASVWQSVQEKYFKRTNRRKRSATTRKTRRKKFKRDYVQPVHAGFKDTVIRGADGSKTKLEYCQIRGTKTDDGYMNLCTSCAATTKLPDNRIPKYINEVICVPDTSCFQNAEGISHGKCKQQVYRLRMIRTRGHKSCKISDKYGKTIVMDDWELYTQTIRTSCECLIDSRSRFAPFVRQ

>PTTH_Nemertea_Baseodiscus_unicolor_TRINITY_DN28154_c0_g1_i1_m.210602

FPPYVQTGRCEQSECMFGMYQCKPKHYVIRVLRRNPVQCNPLPVIGVNTTYEEAWDFAKYKITICCECTRSRRSSRRSGRRRKNKGRNLAEIGVQ

>PTTH_Nemertea_Cephalothrix_hongkongiensis_comp6562_c0_seq1_m.3379

WHCEMKALWLQMPKGYFPPYIRSGKCTQTTCMLNMYQCTPKYYVIKVLRRNPIQCNPIPSVGTNTTYEEVWEFVRQKIIVCCECSRKTIHTRGRLP

>PTTH_Nemertea_Nipponemertes_spec_Contig18028_m.132460

MSEGYFPQYVQTGKCSQSKCMFSMYQCRAKRYVIRVLRRNPLRCNPIPALGFNSTYEEAWEVLKYKITICCECTFINSGSRTRGRKGKRKKTKKVRGGGLQ

>PTTH_Nemertea_Cerebratulus_marginatus_comp1298868_c0_seq1_m.146053

APTAIGKTNLEEPPLHCELKNEWVRMPTGYFPPYVQTGRCEQSNCMFNMYQCKPKHYVIRVLRRNPEQCNPLPVLGVNTTYEEAWDFAKYKITICCECTRSRLLGRN

>PTTH_Nemertea_Riseriellus_occultus_TRINITY_DN15878_c1_g1_i1_m.123675

PSCERRRANRRKGRDRTGIGKTNLEEPPWHCELKTEWVR

MPTGYFPPYVQTGRCEQSNCMFGMYQCKPKHYVIRVLRRNPEQCNPLPVLGVNTTYEEAWDFAKYKITICCECSRSRQFGRQKSRRKNKNKNKNKSKNKGFAGIGVE

>PTTH_Nemertea_Cerebratulus_spec_TRINITY_DN22517_c0_g1_i1.p1

MQIRPIFLRGPHVCTYIMIALAFGVATPKRWNTIDIGVKSDSNRTLSKFKDKDCLPRSEDDLVQLMDSHFMSNKMAMKLPRGPQRDPDSQSKSSTRGSRKSRRHIFDKNNNENERETIRTVSEDSLYSHETVDFKYPIGGDSGKPTIN

>PTTH_Nemertea_Cerebratulus_spec_TRINITY_DN22517_c1_g1_i1_m.182138

SGKPTINLQDFTISSPSEIDEVDNLKRVSSSDRGAKGRDSRTSQKFTRRRLLQRTRRKADTPRRDRDRGNRRRGKARTAIGKANLEEPPWHCELKNEWVRMPTGYFPPYVQTGRCEQSNCMFGMYQCKPKHYVIRVLRRNPEQCNPLPVLGVNTTYEEAWDFAKYKITICCECSRSRQFSRSKSRRKNKNRNRNRNRDKGLVGIGIQ

>PTTH_Nemertea_Notospermus_geniculatus_g8521.t1

MIQIRPIFLRGPHVCTYIMIALAFGVATPKRWNNIDIGLKSDSNRTHVSYKDRECLPRSEDDLVRLMDSHFMSNKMATRLPRGPGGGLDTDIKPRRRGGRQSRHIDHNDREKIRTVSEDSLYSHETVDFKYPIGNEDTPTISLDDFTVSLPSQIDEVDNLKRVSSSDISKDKGGSRSSQKFTRRRLLQRTRRKASPRRKGNRHKGRAPTGIGKTNLEEPPWHCELKTEWVRMPTGYFPPYVQTGRCEQSNCMFGMYQCKPKHYVIRVLRRNPKQCNPIPVLGVNTTYEEAWDFAKYKITICCECSRSRQFGRQKSRRKNKNRKNRGKGYAGIGVE

>PTTH_Nemertea_Lineus_lacteus_comp15989_c0_seq1_m.24249

MQIRPIFLRGPHVCTYIMIALAFGVATPKRWNNIDIGLNSKSNRTLMNFKDRQCLPRSEDDLVRLMDSHFMSNKMATKLPRGPGRGFGSTSKSTSRGGRTSRHIYDNDRGPTAGLRTVSEDSLYSHETVDFKYPVGGENSPTINLQDFTVSLPSQIDEVDNLKRVSDSAERRGGDKRTTQKFTKRRLLQRTRRKASTPRERRRANRHKGRDRTGIGKTNLEEPPWHCELKTEWVRMPTGYFPPYVQTGRCEQSNCMFGMYQCKPKHYVIRVLRRNPEQCNPLPVLGVNTTYEEAWDFAKYKITICCECSRSRQFGRQKSRRKNKNKNKGFAGIGVE

>PTTH_Nemertea_Lineus_ruber_Lvir.rna.tri.60039.1_m.104579

MQIRPIFLRGPHVCTYIMIALAFGVAAPKRWNNIDIGLNSKSNRTLMNYKDRQCLPRSEDELVRLMDSHFMSNKMATKLPRGPGRELESTSKPSSRGGRTSRHIFTDDRSPMAGLRTVSEDSLYSHETVDFKYPVGDKDSPTINLQDFTVSLPSQIDEVDNLKRVSSSDSAERGGGDKSTTQKFTRRRLLQRTRRKATNTRDRRRANRRKGRDRTGIGKTNLEEPPWHCELKTEWVRMPTGYFPPYVQTGRCEQSDCMFGMYQCKPKHYVIRVLRRNPEQCNPIPVLGVNTTYEEAWDFAKYKITICCECRSRQFGRQKSRRKNKNKNKNKNKNKG

## QSGamide/iPTH

>QSGamide_iPTH_Phoronida_Phoronis_australis_transcriptome_TRINITY_DN313826_c2_g1_i1.p1

MRSRDFVVFAVLFVIVVVFSSAKSLPLSLRRLQRRQADVRLADMLASINQQGASPTGCAEVGCGLNLDEVSKRSNADQRLAEMQAFLSLYGHGSTMPDPLRAGKRSVEKQRSPMIYPDERDRETEIVYRLLRRLAHEMDERL*

>QSGamide_iPTH_Phoronida_Phoronis_australis_transcriptome_TRINITY_DN313826_c2_g1_i2.p1

MRSRDFVVFAVLFVIVVVFSSAKSLPLSLRRLQRRQADVRLADMLASINQQGASPTGCAEVGCGLNLDEVGKRSVEKQRSPMIYPDERDRETEIVYRLLRRLAHEMDERL*

>QSGamide_iPTH_Phoronida_Phoronis_australis_transcriptome_TRINITY_DN313826_c2_g1_i3.p1

MRSRDFVVFAVLFVIVVVFSSAKSLPLRSKRSNADQRLAEMQAFLSLYGHGSTMPDPLRAGKRSVEKQRSPMIYPDERDRETEIVYRLLRRLAHEMDERL*

>QSGamide_Phoronida_Phoronis_australis_TRINITY_DN313826_c2_g2_i1.p1

MRSRDFVVFAVLFVIVVVFSSAKSLPLSLRRLQRRQADVRLADMLASINQQGASPTGCAEVGCGLNLDEV*

>QSGamide_iPTH_Phoronida_Phoronis_psammophila_comp63713_c0_seq1_len_1334.p1

MRSRDLLILTVFVVIVIVNSTARSLPGFRRLQRRQSYVRLSDMLANLNLQGIAPTGCAEIGCGSLNAKQIGKRSSEVQRPAYLYPDERERETEILYMLLQRLAEEVDERL*

>QSGamide_iPTH_Phoronida_Phoronis_psammophila_comp63713_c0_seq2_len_1145.p1

YKRSNADQSLANLQALLGLYTDSPSRPDPNEVGKRSSEVQRPAYLYPDERERETEILYMLLQRLAEEVDERL*

>QSGamide_iPTH_Phoronida_Phoronis_ijimai_(P._vancouverensis)_comp54816_c0_seq1.p1

MRCRDFVILTIFFIVVFVFSSAKSLPLSLRQLQRRQADVRLADWLASINQQGAQPTGCAEVGCGLNINRASKRSNADQRLAEMQALLSLYGHRTSMPDPAVAGKRSVEKQRPSMIYPDERDRETEIVYRLLRRLAHEMDERL*

>QSGamide_iPTH_Phoronida_Phoronis_ijimai_(P._vancouverensis)_comp54816_c0_seq2.p1

MRCRDFVILTIFFIVVFVFSSAKSLPLRSKRSNADQRLAEMQALLSLYGHRTSMPDPAVAGKRSVEKQRPSMIYPDERDRETEIVYRLLRRLAHEMDERL*

>QSGamide_iPTH_Brachiopoda_Glottidia_pyramidata_comp40355_c0_seq1.p1

MQKAQSCSALLLATIVIMVLASTSAKSISSSQHSSRVRRQWGDQRMAELLPLIGLMRDGPGSVGHGMVDPMANGKRKRDSTSADYYYDSLENSFGDLRKERMLQRLTQLLLEGRK*

>QSGamide_iPTH_Brachiopoda_Lingula_anatina_comp131389_c0_seq2.p1

MQKTHSCAALLLATLAFMMLAFTSAKSISSVQHSSRVKRQWADARMAELLPLMGLMRGGPGSVAHGMVDPAVNGKRKRESTDYYYNNGESSFSDLRKERILQRLTQLLIEGNQ*

>QSGamide_iPTH_Brachiopoda_Lingula_anatina_comp131389_c0_seq3.p1

MQKTHSCAALLLATLAFMMLAFTSAKSISSVQHSRHRVRRQGGDMTIADRLAWLSQNIRQPVGCSDAACGFVDTDKIGKRKRESTDYYYNNGESSFSDLRKERILQRLTQLLIEGNQ*

>QSGamide_iPTH_Brachiopoda_Lingula_anatina_comp131389_c0_seq4.p1

MQKTHSCAALLLATLAFMMLAFTSAKSISSVQHSRHRVRRQGGDMTIADRLAWLSQNIRQPVGCSDAACGFVDTDKM*

>QSGamide_iPTH_Brachiopoda_Novocrania_anomala_Nano.rna.tri2.29012.1.p1

MCKVQVLLFALVAVIFVSSISARNIPEHRRQKRQHVEIDAAEILALITRNRKPPSCVNVACGQGDPNERGKRASRNLVEYYPPDNESRRYRLLQRLVEDYLQENS*

>QSGamide_iPTH_Nemertea_Baseodiscus_unicolor_TRINITY_DN95747_c0_g1_i1.p1

MDRSQVIFIACILTVGFITVYSKSISKETHRSKRHTTDLEGNEYSEKLALGNQDNDCGSFGSCISKIERRASSDQRVAELQALIALSRGNGLVGHGQVDPLLAGKRKRAHSFSRLDKMEEERLLETLLERLLLDNVMADSE*

>QSGamide_iPTH_Nemertea_Cephalothrix_hongkongiensis_comp27568_c0_seq1.p1

MQTFRVVIVTCVFALVVAGITANSAQSVRHRQRRASSDQRIAELQALIALSRGNGIVGYGHVNPFIAGKRKRGMVNLKLTSEEHVMFK*

>QSGamide_iPTH_Nemertea_Cerebratulus_marginatus_comp25166_c0_seq1.p1

CSELQALIALSRGSGLVGHGHIDPFMAGKRKRGNSLLALNPTEKDRLLSSLIERALAVQDNEQS*

>QSGamide_iPTH_Nemertea_Cerebratulus_sp_Contig6988.p1

MEKAQLILFVCVCAVTLLTISGKSIGNDNHREKRASSDQRVAELQALIALSRGSGLVGHGHIDPFMAGKRKRANSLFNAAENKDRLLQSLIERALAVEGNQQS*

>QSGamide_iPTH_Nemertea_Lineus_lacteus_comp19318_c0_seq1.p2

MEKGQVILFVCVFAVILITASSKSIENDKNRDKRASSDQRVAELQALIALSRGSGLVGHGHIDPFMAGKRKRAELLSALNPTEKDRLLSTLIERILAAQANEDS*

>QSGamide_iPTH_Nemertea_Lineus_longissimus_c41121_g1_i1.p1

MEKGQVILFVCVFAVILITASSKSIENDKNRDKRASSDQRVAELQALIALSRGSGLVGHGHIDPFMAGKRKRAELLSALNPTEKDRLLSTLIERVLAARANEDS*

>QSGamide_iPTH_Nemertea_Lineus_viridis_Lvir.rna.tri.53968.1.p1

MEKGQVILFVCVFAVILITASSKSIENDKNRDKRASSDQRVAELQALIALSRGNGLVGHGHIDPFMAGKRKRANLLSSLNPTEKDRLLNTLIDQVLAAGGSDDS*

>QSGamide_iPTH_Nemertea_Malacobdella_grossa_comp20890_c0_seq1.p2

MSQIYHLMVISLICFVLTVSLPTHTNALSSRTKRASSDQRMAELQALIALSRGGSVGHGQIDPYIVGKRSRTIWKQSGANSEQILRYLRAHQVYYGGDE*

>QSGamide_iPTH_Nemertea_Notospermus_geniculatus_scaffold375.g12542.t1.p1

MEKGHVILFVCVFATILITVSSKSIEHDIHREKRASSDQRVAELQALIALSRGRGLVGHGHIDPYMAGKRKRPITGNADTASSGRKRGGRVISLVASAHHCQDD*

>QSGamide_iPTH_Nemertea_Paranemertes_peregrina_comp26023_c0_seq1_len_733.p1

VTLLCFTLIVSLPTHTDTDAISSRTKRASADQRIAELQALIALSNGGSVGHGQIDPYVVGKRSQRKEATEKMLRYLLSHQGATYYGDE*

>QSGamide_iPTH_Nemertea_Paranemertes_peregrina_comp26785_c0_seq1_len_643.p1

MSRMHQILVVTLLCFTLIVSLPYRSRTKRIMAESEIDEKRATIGLNNRADTTYLHGQLDPEEIGKRSLPREATQS*

>QSGamide_iPTH_Nemertea_Riseriellus_occultus_TRINITY_DN78284_c0_g1_i1.p2

ALSRGSGLVGHGHIDPFMAGKRKRANLLSSLNPTEKDRLLSTLIERVLAAQGSEDS*

>QSGamide_iPTH_Nemertea_Tubulanus_polymorphus_comp30090_c0_seq1.p1

MYKIQVVIFASAFTVILVTASSVGSAHAQQRYKRASSDQRIAELQALIALSRGRSALVGHGELDPRIVGKRNGDMSDMQKEQRIFEARQIIRRLLDDLGNKNL*

## RGWamide

>RGWa_Brachiopoda_Terebratalia_transversa_Ttra.rna.tri.13054.1.p2

QRRNSDGLGKRGWGKRGWGKRNGLLGWGKREDLGWGKRSEVLQDLSKDLSRGWGKRGWGKRGWGKRADQTTDYFNVGHQREPFYVKKGWGKRAIQNRELEGAFFKVSHPVVWSSQSRLAMKRLYVKALMCSISKMLSVTGNSRLFKGKDFVKKIMDMCIL*

>RGWa_Brachiopoda_Novocrania_anomala_Nano.rna.tri2.122202.1.p2

MRFLSTASVLCWVGAAVVLCNASDRATDDTTRSGWGKRNSDGAEQISEQTKQGKEMPFVDFSTKRGWGKRGWGKRAQETEGPGLSVDDPKLNDERRRAFVEREMRRMGW

>XP_013419038.1 uncharacterized protein LOC106179816 [Lingula anatina] Brachiopoda

MKSFLVIVELLVYSLLFSSCYVQGGDRYRRRMGWGKRSEPQVSDISYLLSAVDSLSQNPPGNNGDDMDRPNRGWGKRFSGSDMASTMDKRGWGKRSTTLDLEEVKRAMGWGKRSFFNESPVRRAMGWGKRERAPAGWGKRNQILVPELKKGLGWGKRILKSDDVADVEDAPQMDYNRRFLPGNSSASATDLSSPVSNKESTDIRDKSAQCQNNIEAFSRMLDMFVKILTYESCDLEREVISKMLAQVRMSLLID*

>RGWa_Nemertea_Tubulanus_polymorphus_comp19184_c0_seq1_m.8746 complete

MQKIIVAVAFLGLVVLELVLPAFADTQPPADDMDKRGWGKRGWGKRDDNSYDQLEDALKRGWGKRGWGKRGWGKRGWGKREDCDGDLLDDVIRNIYSAVQIEVKRVYACAGASASDNTQ*

>RGWa_Nemertea_Malacobdella_grossa_comp4840_c0_seq1_m.2104 internal

FAAEPEPQASDMDKRGWGKRGWGKRGWGKRDAEESSSLDEVLKRGWGKRGWGKRGWGKRGWGKRGDESNVCEQLSDESLYHLQKI

>RGWa_Nemertea_Nipponemertes_spec_TRINITY_DN14574_c0_g2_i1_m.70191 5prime_partial

TETQASDMEKRGWGKRGWGKRDGDEYSSLDDMVKRGWGKRGWGKRGWGKRGWGKRSDDGNMCEQLSDDTIYYLQKILEIETKKQDACNIEPIAA*

>RGWa_Nemertea_Riseriellus_occultus_TRINITY_DN45998_c0_g1_i2_m.79938 5prime_partial

FASAAAVETPDQDMDKRAPGWGKRGWGKRDDDSDAALDDYLKRGWGKRGWGKRGWGKRGWGKRDMDGSSCEDLNQGVIYYIYKAVEAEAQRINACAGAEN*

>RGWa_Nemertea_Cephalothrix_hongkongiensis_comp26190_c0_seq2_m.17073 complete

MKTYIAFALLGIVLLDVCTRAFAEEQSDEGLEKRGWGKRGWGKRDGENDDALEEALKKRGWGKRGWGKRGWGKRGWGKRGEDSCFDAVNDEVMYNVLKAIQNEAVRLNACTSESK*

>RGWa_Nemertea_Notospermus_geniculatus_g26977.t1

MKFYIAITLVCLLVFDIFASAAAAENADQDLDKRAPGWGKRGWGKRDDESDASLDDYLKRGWGKRGWGKRGWGKRGWGKRDDTPSCDDLNQGVLLFIYKAIEAEAQRITACSSQEN

>RGWa_Nemertea_Paranemertes_peregrina_comp243158_c0_seq1.p1

MKLYIALALLAIVLLDLATSAFAADAEPQTAELDKRGWGKRGWGKRGWGKRDEDDASSLDEVLKRGWGKRGWGKRGWGKRGWGKRV

>RGWa_Nemertea_Cerebratulus_spec_TRINITY_DN31779_c0_g1_i2_m.37985 complete

MKVYIAITLVCLLVFDVFASAAAAENADQDLDKRAPGWGKRGWGKRDDDSNVDASLDDYLKRGWGKRGWGKRGWGKRGWGKREDGPSCDELKEGVFYYIYKAVEAEATRINACASSEN*

>RGWa_Nemertea_Cerebratulus_marginatus_comp32101_c0_seq2.p1

MKFYIAITLVCLLVFEIFASAAAAENADQDLDKRAPGWGKRGWGKRDDETADSSLDDYLKRGWGKRGWGKRGWGKRGWGKREDGPSCDELKEGVFFYIYKAVEAEAQRINACSSSEN*

>RGWa_Nemertea_Lineus_longissimus_c38836_g1_i1_m.49381 complete

MKLYIAITLVCLLVFEIFASAAAAETADQDMDKRAPGWGKRGWGKRDDDSEGALDDYLKRGWGKRGWGKRGWGKRGWGKRGWGKRGWGKRDIDGAPCEDLNQGVIYYIYKAVEAEAQRINACASTDN*

>RGWa_Nemertea_Baseodiscus_unicolor_TRINITY_DN97413_c0_g3_i1.p1

MKQYIALSLVALFFVEIFTSALAAESQNDDLEKRVPGWGKRGWGKRVDDDGAGLDDYLLKRGWGKRGWGKRGWGKRGWGKREDGPACDELSEDVLFNIYKAVKAEAYRINSCPSSQEN*

>RGWa_Nemertea_Lineus_lacteus_comp19718_c0_seq2.p1

MKFYIAITLVCLLVFEIFASAAAAETPDQDMDKRAPGWGKRGWGKRDDDSESAVDDYLKRGWGKRGWGKRGWGKRGWGKRDVDGASCEDLNQGVIYYIYKAVEAEAQRINACASTEN*

## sCAP

>sCAP_Nemertea_Lineus_ruber_Lvir.rna.tri.122851.1_m.147439

QKANANYISLDDNVKARPLDPYAECCPIGYKSELQLDDFGKEVVYNHCKVTTCCMGLKEILKLGSNGLTYSMCVRDEDRSLRDLKGLAALARILKRYYGDEPMQK

>sCAP_Nemertea_Lineus_longissimus_c44656_g1_i1_m.95005

MSHRRQHRSVCGAIGFLAICCSLSQKANANYRGPADNMNLRSLDPYAECCPIGYKSEVRLDDFGKEVVYNHCKVVNCCIGLKEILKLGSNGLTYSMCVRDEGRSMHDLKGLAELARILKRYYGDEPMQKRTRLAFPSNDYY

>sCAP_Nemertea_Notospermus_geniculatus_g12504.t1

MISGRRIGMAFRVFGTLTVCCSLLTITNATSTNLGAMFKRQPFDILYQREEECCPIGRKSEVQLDSSGDQIIVNRCRVLKCCKGLKEVLRLGSNELTYSACVHEGPTMQDLKGLAILAKYLKRYYRDESAEGKRSTSWQFEDSY

>sCAP_Nemertea_Notospermus_geniculatus_g32017.t1

MISGRRIGMAFRVFGTLTVCCSLLTITNATSTNLGAMFKRQPFDILYQREEECCPIGRKSEVQLDSSGDQIIVNRCKVLKCCNGLKEVLRLGSNELTYSACVHEVNNVYHENRQVNRVLKCCKGLKEVLRLGSNELTYSACVHEGPTMQDLKGLAILAKYLKRYYRDESAEGKRSTSWQFEDSY

## SIFamide/FF peptide

>SIFa_FFa_Lopho_Nemertea_Cerebratulus_spec_TRINITY_DN25676_c0_g2_i1_m.100766

MDRYIKLSIILLFCSLLVCGQASQGIDKLLKSQSLLFGRKRSPDPAGSNLFFGRRSYEPQQYGRQFCANVIQTCRYWYDQSNDVTGNGQ

>SIFa_FFa_Lopho_Nemertea_Lineus_longissimus_c40583_g1_i1_m.60109

MNCYVKLSIILCFCSLLVCGQASQGIDKLLKSPNLLFGRKRSPDPAGSNLFFGRRSFEPQQYGRQFCANVIETCRYWYEQNNDVSATEQS

>SIFa_FFa_Lopho_Nemertea_Lineus_lacteus_comp35113_c0_seq1.p1

MACYIKLSIILCFCTLLVCGQASQGIDKLLKSPNLLFGRKRSPDPAGSNLFFGRRSFEPQQYGRHFCANVIETCRYWYEQNNDVSSTEQS*

>SIFa_FFa_Lopho_Nemertea_Lineus_ruber_Lvir.rna.tri.52961.1.p1

MDRYIKLSIILCFCSLLVCGQASQGIDKLLKSQNLLFGRKRSPDPADSNLFFGRRSYEPRQYGRQFCANVIETCRYWYEQNSDVSATEQN*

## sNPF/LFRFamide

>sNPF_FRFa_Phoronida_Phoronopsis_harmeri_c687_g1_i1.p1

AIAHAASVSDARQGLEPSLEKLKGFLKTASIPDAENSNNYYTDDILPPVYLSKTIDAGDEDKRGTLFRYGKRGSIFRFGK

>sNPF_FRFa_Phoronida_Phoronis_australis_TRINITY_DN283642_c0_g1_i1.p1

MELTSLLVLFISSVLALSHSTIGLETSHLEPAIEKLKGLLKNAEEKPTEDGESYYTSDVIPPVYVTGNVEESDVPDKRGTLFRYGKRGSIFRFGKRGYVFRYGRGRDEGFNDGLDEGYGTRLDDKRAIFRYGKRGARLHAPFRFGKSLEQESRKR*

>sNPF_FRFa_Phoronida_Phoronis_psammophila_comp64771_c0_seq1.p1

MECSGLLMLFVSSVLLAITQAASVAEARQQLEPSLEKLKGYLKNAQESAASSNHDSYFTDDVLPPMYISRTVDEGDEDNDEGYAKRGSIFRFGKRGYIFRYGRGDDDGFADGLEYYPDKRTIFRYGKRRIHAPFRFGKDLDESGSRRK*

>sNPF_FRFa_Phoronida_Phoronis_ijimai_(P._vancouverensis)_comp84809_c0_seq1.p1

MELTSILVVFISSVLALSQSTMGLETSHLEQGIEKLKGLLKNAEDKTNEDGEGYFTSDVIPPVYISETVDDSDAADKRGTLFRYGKRGSIFRFGKRGYVFRYGRGRDEGFNDGLDEEYRTRLADKRAIFRYGKRGARLHAPFRFGKSLEQESRRR*

>sNPF_FRFa_Brachiopoda_Laqueus_californicus_comp42905_c0_seq1.p1

MERTYITITFTIAVVLCYAATILCDDADEQQSFIKAKGSGQRSRSLFRYGKRSGIENEKRTSVFRYGRNLGILLHQLALRPYTYDSDSLANLPPNNNKQYLVADTNSKERRGFSAIHLGKRKIGYNYNFGHLLSYLNQIPAEEYPKTETAQHKERQIQAHVPFRLGRYVKR*

>sNPF_FRFa_Brachiopoda_Terebratalia_transversa_Ttra.rna.tri.5620.1.p1

MERTTYRITIIAVIVCYVTSLSWCDETDERRKFLIQAGAQRARSLFRYGKRTGIENEKKASVLRYGRKMRLLLHHLSHQPYYNAYADPENISKNSQYKDADSNSKLVRRGFSAIHLGKRTLGYNYNFDDLMTYLNRLRDDEFPKTETGQDKERQIQAHVPFRLGRFIKDDDM*

>sNPF_FRFa_Brachiopoda_Novocrania_anomala_Nano.rna.tri2.26164.1.p1

MGNVRLYCVVTVLCALMAFTSAQKEESRRLADMISHWKKMYGKISPFPKDDQPFAKSADGPQDEDNFFSNLKEDSGDTKNLLDDKQYSLDNFDKRGTLFKYGKRSSLFKYGKRGSLFKYGKRLQPCIPFYRTINTRDTTKRDDDNSVPNDLMKRVLCYRPGLQEVQIRQAAHVPFRFGRAMEDDNA*

>RFa_MPCs_Brachiopoda_Glottidia_pyramidata_comp46828_c0_seq1.p1

MNQTRLFLWACVLCVVILAAEVFGDAEISENVGAFKRGPIWRYGKRGMWRYGKRGVWRYGKRGIWRYGKRGMWRYGKRDPEDDDEVSTELDLPEEDKRAMLRWGKRVFHFGKKSNDDSQQ*

>lingulaAnatina.g8124.t1 Brachiopoda

MTGQNIMIMDQTRLFLWACFLCSVVLTAAVFGDAEISEDIGAFKRGPIWRYGKRGMWRYGKRGVWRYGKRGIWRYGKRGMWRYGKRGPEDEEMTSDDLEPISELDTPDDKKAMLRWGKRVFHFGKKSDESSQ

>sNPF_FRFa_Nemertea_Cerebratulus_marginatus_comp33051_c0_seq3_m.18454 complete

MFRFGRGALFRFGKRGNGLFRYGKRGGSLFRFGKRGGSLFRFGKRDEDDMNTEEKRKSMFRYGRGDMMEDEKRKSMFRYGKRFVRDTKRQKVHTPFRFGREEDEEA*

>sNPF_FRFa_Nemertea_Riseriellus_occultus_TRINITY_DN22115_c0_g1_i1_m.43056 5prime_partial

EDLEEALEPYYNDYGNEDLDKRGSMFRFGRGAMFRFGKRGKGFRYGKRGGSIFRFGKRGGSLFRFGKRQDSSDLNTEEKRKSMFRYGRGDMMDEEKRKSMFRYGKRFVRDTTKRQKVHTPFRFGREEDEEV*

>sNPF_FRFa_Nemertea_Baseodiscus_unicolor_TRINITY_DN101569_c0_g6_i1_m.76098 internal

YILLASIAALVLVVIATAEDIDTNKLEHLQDIIQDEPAMASTEDREDPYYNEYGWDDVDKRGTLLRYGRGSLFRFGKRGGLFRYGKRGGSIFRFGKRGGSIFRFGKRSDDDDSEAEKRNSIFRYGKRDDDDSQKRSMF

>sNPF_FRFa_Nemertea_Cephalothrix_hongkongiensis_comp32203_c1_seq2_m.28043 5prime_partial

FLSAKQATGSKLGALIESLKEDPTISSESDENFPISSEEYDMDKRGNLMRYGRANIFRYGKRGAIFRYGKRGNLMRYGKRQNLFRYGKRDEEDSYDAAEKRGQLMRYGKRDDEMTEQEKRANLMRYGKRFARDTLTMKRQNNKPHVPFRFGRDEEELLA*

>sNPF_FRFa_Nemertea_Lineus_ruber_Lvir.rna.tri.53449.1_m.95969 complete

MEPKQLIYLSIAVLVTVFTVSAEELDNKVEQALQDEPTMASTEDLEEALEPYYNEYGNDDLDKRGAMFRFGRGAIFRFGKRGKGFRYGKRGGSIFRFGKRGGSIFRFGKREDNSDMNTEEKRKSMFRYGRGDMMDEEKRKSMFRYGKRFVRDTKRQKVHTPFRFGREEDEEA*

>sNPF_FRFa_Nemertea_Lineus_lacteus_comp25296_c0_seq2_m.85560 complete

MEPKQLIYLSIAVFMAVLTVSAEQLDNKVEEALQDDPTMASTEDLEEELEPYYNNYGDDDLDKRGSMFRFGRGAIFRFGKRGKGFRYGKRGGSIFRFGKRGGSLFRFGKRQDNSDTNTEEKRKSMFRYGRGDMMDELKRKSMFRYGKRFTRDTTKRQKVHTPFRFGREEDEEV*

>sNPF_FRFa_Nemertea_Paranemertes_peregrina_comp14914_c0_seq1.p1

MDIRPVICLFLWVVLAVSAESSPQDKINGYGDDPSALDDVTLERILAGYDEPSKRGTMLRYGRGTIFRFGKRPSTFRYGKRGTIFRFGKRDEDDDSSYDDAVEKRGMLFRYGKRMDETVKRGSLLRYGKRFARDTKRQKVHKPFRFGRELEEDEV*

>sNPF_FRFa_Nemertea_Notospermus_geniculatus_scaffold1880.g30064.t1.p1

MPQDRQDKAIFAVCISRPRSHRRCDVLVKKEALQDEPTMTSTEDLEDDLEPYYNDYGYNNDVDKRGSMFRFGRGALFRFGKRGNGLFRYGKRGGSIFRFGKRGGSLFRFGKRQDSDTEEKRKSMFRYGRGDMMDADKKSMFRYGKRFVRDTKRQKVHAPFRFGRQEDDEE*

>sNPF_FRFa_Nemertea_Notospermus_geniculatus_scaffold693.g18085.t1.p1

MASGFRLRKPDEMAKQGLGENTNMETKQLLCLSIAVLVTVFTVSAEELDNKVEEALQEEPTLTSTEDLEDDLEPYYNDYGYNNDVDKRGSMFRFGRGALFRFGKRGNGLFRYGKRGGSIFRFGKRGGSLFRFGKRQDSDTEEKRKSMFRYGRGDMMDADKKSMFRYGKRFVRDTKRQKVHAPFRFGRQEDDEE*

>sNPF_FRFa_Nemertea_Malacobdella_grossa_comp26443_c0_seq2.p1

MSQPQNVTGTFVDDPSSLDDDTLESLIGYEEPSKRGTLLRYGRGTIFRFGKRPSTFRYGKRGTIFRFGKRADELADGDFEEAEKRGMLFRYGKRMEDGFEKRGSLLRYGKRFARDTKRQNVYKPFRFGRELDQYDV*

>sNPF_FRFa_Nemertea_Malacobdella_grossa_comp26443_c0_seq3.p1

FWCCILFNAGTFVDDPSSLDDDTLESLIGYEEPSKRGTLLRYGRGTIFRFGKRPSTFRYGKRGTIFRFGKRADELADGDFEEAEKRGMLFRYGKRMEDGFEKRGSLLRYGKRFARDTKRQNVYKPFRFGRELDQYDV*

>sNPF_FRFa_Nemertea_Malacobdella_grossa_comp26443_c0_seq1.p1

MDLRPIICLCLWAVIAVYAQSSSEDKLAGTFVDDPSSLDDDTLESLIGYEEPSKRGTLLRYGRGTIFRFGKRPSTFRYGKRGTIFRFGKRADELADGDFEEAEKRGMLFRYGKRMEDGFEKRGSLLRYGKRFARDTKRQNVYKPFRFGRELDQYDV*

>sNPF_FRFa_Nemertea_Cerebratulus_spec_TRINITY_DN35403_c2_g2_i4_m.91042 complete

MESKQIIYLSIAVLATVFAVSAAELDNKVEEALSDDPTIASNELEESLEPYYDYYGDNDLDKRGSMFRFGRGALFRFGKRGSGMFRYGKRGGSIFRFGKRGGSLFRFGKRDDDDMNTEEKRKSMFRYGRGDLMEDEKRKSMFRYGKRFVRDTKRQKVHTPFRFGREEDEEV*

>sNPF_FRFa_Nemertea_Cerebratulus_spec_TRINITY_DN35403_c2_g2_i1_m.91033 complete

MESKQIIYLSIAVLATVFAVSAAELDNKVVDMLLGRLRQFDRRDSLDEARYRLLQEALSDDPTIASNELEESLEPYYDYYGDNDLDKRGSMFRFGRGALFRFGKRGSGMFRYGKRGGSIFRFGKRGGSLFRFGKRDDDDMNTEEKRKSMFRYGRGDLMEDEKRKSMFRYGKRFVRDTKRQKVHTPFRFGREEDEEV*

>sNPF_FRFa_Nemertea_Lineus_longissimus_c19275_g1_i1_m.10461 complete

MEAKHLIYLSIAVLVTVFTVTAEELDNNVEEALQDDPTMASTEDLEDSLEPYYNDYGNGDLDKRGSMFRFGRGALFRFGKRGKGFRYGKRGGSIFRFGKRGGSLFRFGKRQDNSDMNTEEKRKSMFRYGRGDMIDGEKRKSMFRYGKRFTRDTTKRQKVHTPFRFGREEDEEV*

>sNPF_FRFa_Nemertea_Lineus_longissimus_c19275_g1_i2_m.10463 complete

MEAKHLIYLSIAVLVTVFTVTAEELDNNVVDLLIGRMRESGSRNSLDDSYQMALQEALQDDPTMASTEDLEDSLEPYYNDYGNGDLDKRGSMFRFGRGALFRFGKRGKGFRYGKRGGSIFRFGKRGGSLFRFGKRQDNSDMNTEEKRKSMFRYGRGDMIDGEKRKSMFRYGKRFTRDTTKRQKVHTPFRFGREEDEEV*

>sNPF_FRFa_Nemertea_Nipponemertes_spec_TRINITY_DN24536_c0_g2_i1.p1

FGLVRWMQTIPAGCLSQTVLETELTRRQRSAEGSRAVNTKESIVMDVRSILCLTVWALLAVWVTAETQHSSKINDIDTSSSEDDAALESLLGYEDPSKRGTMLRYGRGSLFRFGKRPRTFRYGKRGSLFRFGKREDFDKREDFDEDVEKRGLFRYGKRFDVDEKRGNLFRYGKRFARDTKRQKVHTPFRFGRELESDEI*

## Sulfakinin

>Sulfakinin_Lopho_Phoronida_Phoronopsis_harmeri_c103818_g1_i1.p1

WSPFSKPSTAYSVVTFGKRGPSNYFYGGGRFGRSVGDFDHVDPETEDAFD

>Sulfakinin_Lopho_Phoronida_Phoronis_psammophila_comp65363_c0_seq1.p1

MKNLIRSLLPLIFVCQMVYVDSLPLFTRVPHHTIRNFVDIIKDLAKQQRQQEADINNNLQNTNPFVYPLMTGDTTQEKFARLNTDLYQEKTDGSAVNTQHSFDLPSDKRDYGIGGGRFGKRGPSNYFYGGGRFGRSVENFDHVDPDAEDAFE

>Sulfakinin_Lopho_Phoronida_Phoronis_ijimai_(P._vancouverensis)_comp98771_c2_seq5.p1

MKNLIRSILPLIFVCQLVHVANSAPFFTKVPQHTIMSFADIIKDLSRRQQHSKELDQRPQQQQEMGAKDSEYVAKDLLYPILVHNKAGKMLEQLNQRTLFGQKLSKPSEAKQVVDNPYDIPNDKRDYGIGGGRFGKRKSPTKTPRSRYFYGGGRFGRSVANFDHVDPETEEAFD

>Sulfakinin_Lopho_Phoronida_Phoronis_australis_TRINITY_DN301908_c0_g4_i1.p1

MKNLIRSVLPLISICQLVHVANSAPFFTKVPQHSIMGFVDILKDFSSRHPHTKELDQRPEQPQEMGPKQGEYGAKDMLYPILVSSKAEERFEPLSQRTLFGQKLSKPSEANQDVENLYDIPNDKRDYGIGGGRFGKRKSPTKKTPRSRYFYGGGRFGRSVANFDHVDPETEEAFD

>Sulfakinin_Lopho_Brachiopoda_Novocrania_anomala_Nano.rna.tri2.60784.1.p1

MKMVYLSRSALFCLTVCTFGFGYSSQRALNKDGQDSVRRILQQLQHTLPNTWQMDLIAGLRGVELRNTLSDDSSNKKVLSKNTDWQELVIPVDFSKEKTKREFGDYGYGGGKFGKRGDVDGQKKDTPDYWIGGGRFGRDVDHVDVEHLTMDED

>Sulfakinin_Lopho_Brachiopoda_Lingula_anatina_comp114177_c0_seq1.p1

MKSVFFVCIFFTTIVINVDCAPWQIGNRLRTSHISQAMHKLQGSLHRIFYLGRKKPNSGQYFTNSFSTLKGGEPITNIYPMKGYIQVDQTKPVAASMQLKAFNGMDSPTTGKQEKRAWDDYGLGGGRYGKREWKDGTSGLTLTQDATKHSNGNTNSKRSDIYWLAGGRFGRDVSHVTDHVDPENDDDIEN

>Sulfakinin_Lopho_Brachiopoda_Glottidia_pyramidata_comp39614_c0_seq1.p1

MKPVFFVCIFFTCVVLNVDCAPWQLTKRLQSSHISHAMHKLQGSLQRIFYLGRNKPHSGQQYFTNSFSTLEQSADPITNMYPMNGFIQVDQTKPAAATSIQLKAYPDLDSSLNDKQEKRAWDDYGLGGGRYGKRDLKDGLEGLTLHRDPKNKVEDTNSKRSDIYWLAGGRFGRDLSHVTDHVDPDNDDDIGY

>Sulfakinin_Lopho_Brachiopoda_Terebratalia_transversa_Ttra.rna.tri.13421.1.p1

MIVMDQLLPLLLWILCTLPRIPALPLRDHTIDNDIREKLLDNFQHKDIRYNSRISNIIEYFINSKTASDGRYDDDTTSVKEHSKLLHILSRLRNRNSSGSTNTLYHELERPRFVPKLEKEQVQKRDYGQYSDYGYAGGRFGKRAADKRMFMDDYGFAGGRWGRSVQDDDDNDHVDIEH

>Sulfakinin_Lopho_Nemertea_Tubulanus_polymorphus_comp25099_c0_seq1_m.14183

MNSLPILVLLLLIGLTISLPSNRHPHYRTESQRQSSTDIDQSRLDSKRSSRLESLGSIMPLISKNNKMDEFVRILRLLKTTEQLKRHRNELKQQLSNEVRLLREKLNSEYIDRKHQVQNNVKIIHGAEKRQYEDYGWSGGRYGKRSLDEDHINTQSF

>Sulfakinin_Lopho_Nemertea_Riseriellus_occultus_TRINITY_DN27881_c0_g1_i1_m.99014

ILSVFLRSFYKDDSASMWSSSQTKHFSSRPHQIEKVYSALNWKETDDLGDLLALVAATGDQESPAKRQKDMDYGWSGGRFGKRSIDENNDHIF

>Sulfakinin_Lopho_Nemertea_Malacobdella_grossa_comp26044_c0_seq2_m.46369

MNQLILATLIFAVIATITVPSNGNAIPRTSEHPHIPMSTHEFLKLFSLFKQYLAAHKQQQENDEKQPEKRQMNVDYGWSGSRFGKRGVDDYDVIV

>Sulfakinin_Lopho_Nemertea_Notospermus_geniculatus_g30351.t1

MNQALIIGVALFVVCASALPHKRTENAAGAKWSPSHTNHYQPEQFEKVFNALKGKDTDRYDGLLALVAAAGDEQLAEKRQVDMDYGWSGGRFGKRSVDNNDHIF

>Sulfakinin_Lopho_Nemertea_Lineus_lacteus_comp10986_c0_seq1_m.13954

MKHAMVIALALILVSFASAAPHRRSQNDDSAALWSSSKTKHLNFKPQQIEKAYNALNGKETDDFRDILALVAAAEDQQIPAKRQKDIDYGWSGGRFGKRSIDDNNDHLF

>Sulfakinin_Lopho_Nemertea_Lineus_longissimus_c40512_g1_i1_m.59677

MNHAIVIVVALVVVTFASAAPHRRSQNDDSASQWSSSKTKHLSITPQQIEKAYSAMKGKGTDDFGDILALVAAAGDQHVPAKRQKDMDYGWSGGRFGKRSIDDNTDHIF

>Sulfakinin_Lopho_Nemertea_Lineus_ruber_Lvir.rna.tri.44446.1_m.82941

MNHVIVIAVALFVVVFASAAPHRRSQNDDSASQWSSSQTKHFNIRPQQIEKVFNALKGKDTEDIGDLLALVAATGDQQIPAKRQKDMDYGWSGGRFGKRSIDDNNDHLF

>Sulfakinin_Lopho_Nemertea_Cerebratulus_spec_TRINITY_DN32597_c0_g1_i1_m.101435

MNHVIAICIALLVVVCASSAVPHRRSQSDESATQWSSSQTKHFNIRPQQIEKVYNALKAKDTENLGDLLALVATGEDHMAEKRQKDMDYGWSGGRFGKRSISDENNDHIF

>Sulfakinin_Lopho_Nemertea_Cerebratulus_marginatus_comp50500_c0_seq1.p1

MNQLLVICVALLVVVCASAVPHRRSQSDDSASKWAASQTKHFNIRPQQIEKVYNALKAKDTENLSDLLALVATGDEHIAEKRQKDMDYGWSGGRFGKRSIDDDNNDHIF*

>Sulfakinin_Lopho_Nemertea_Baseodiscus_unicolor_TRINITY_DN36768_c0_g1_i1_m.200942

MNYLLIAVLALLTVLEIHASPHGRSRADNSVGQWAAGTGKKYPTDKAKNAGNFYKLFNMMKSVKSSNKADLDKLISFVLNDDGSFAGMDRSKSSDSMLLDSAEKRQVDMDYGWSGGRFGKRSLENRDHIF

## Tachykinin

>tachykinin_Nemertea_Tubulanus_polymorphus_comp21864_c0_seq1_m.11049

MKMIDITRSLAGLIMVLYVVTAQEVDINEIDVLSELKLTNSHLSAIKDMLAALLNSEIPQVQSRRTQSQEMSPFPAPSNNMVLNELTRLMELKHSSDYDRLKTSKRQPPQGFYAVRGKRASEEIISEKRPTGFHAMRGKRSSKEI

>tachykinin_Nemertea_Baseodiscus_unicolor_Contig22050.p1

MDYRISILFLSALICLVTSIPLVSDTGDEDRTTAVLLRIASHLSDIKKLLKTALSDELAFGNGDNSLDEDDSDTGISKTKLLRQLNSFLDLRRYPNSIGP

>tachykinin_Nemertea_Cerebratulus_spec_TRINITY_DN35735_c1_g1_i1_m.102637

MIMDYRISMLVLALCACLVTSIPLGPASSVEDRTTALLMTIAENTSEIKEMLQTVLSSADMIEENNMDDDSDTKAKLLHQLKSYLDLRNYPQSSPSIKKRQPDLGFYAVRGKRNTKTSD

>tachykinin_Nemertea_Lineus_lacteus_comp17312_c0_seq1_m.28042

MAIMDYRISIVFLALSTCLVTSKPLGPSASVEDNTLALLMKIAENTSNIKDMIQTVMISRDMAHGNGIGDDADTKTELLQQLKSYLDLRNLAQTSRSMRKRQPDLGFYAVRGKRNTKSSE

>tachykinin_Nemertea_Lineus_longissimus_c46063_g1_i1.p1

MAIIDYRISIVLLALSTCFVTSKPLGSAASVADSTIALLMKIAENVSNIKDMIETVMIRRDMADGNGIGDNANTKTKLLQQLKSYLDLRNYAQSSQSMKKRQPDLGFYAVRGKRNTKSSD*

>tachykinin_Nemertea_Lineus_ruber_Lvir.rna.tri.75324.1_m.119845

MAIMDYSVSIFVLALSTCLVTSIPLGPAASAEDRTIALLIQIAENISNIKDMIQTINMIRNDMVDQNGTGDEADTKTKLLQQLKSYLDLRSYAQSSKSMKKRQPDLGFYAVRGKRNTHTSE

>tachykinin_Nemertea_Nipponemertes_spec_TRINITY_DN6634_c0_g1_i1_m.33834

MDCRLCTLIAALVTLVACQALSESPLDQDSKSARILLFEMSQDVAEIKSMLKSLLAEERAPRNDENSGRFDDEESSNKLYNEMRRLMHLKQLAPMLSSQLAAKRQPPIGFYAVRGKRGTSA

>tachykinin_Nemertea_Paranemertes_peregrina_comp19598_c0_seq2.p1

MNSRLSCLLFVSAFLAHSSSQTLSDEQSDDSKSTRILLYQMSQDIAEIKSMLHAVLREQSLERQDEDDVPAEMSTNHKLMDQMRKLLLLKNMEKRKNYRP

>tachykinin_Nemertea_Paranemertes_peregrina_comp19598_c0_seq1_m.15794

MNSRLSCLLFVSAFLAHSSSQTLSDEQSDDSKSTRILLYQMSQDIAEIKSMLHAVLREQSLERQDEDDVPAEMSTNHKLMDQMRKLLLLKNMGKELSSRLAVKRDPPVGFYAVRGKRAVAAA

>tachykinin_Nemertea_Cephalothrix_hongkongiensis_comp40066_c0_seq1_m.71495

MNISSATEMTYKSLIFFVFGLSCCLAYPSLENTDTSEEAIILLRQMSEDISDIKDMLSSILKPSMQSSYRNDNGFNPILSEINKLIEIKSNRHLGNTLKAEKRQPPDGFYAVRGKRSLMQQKNQ

>tachykinin_Nemertea_Notospermus_geniculatus_g8442.t1

XXXLASCEEFHKNRRRVTVRVDYLLRAERKITKHGHEDICSGAGAKTTALLAKIADNISDIKSMLQTVLVGGELADDSSISDLSDTKAKLLQQLKTYLDLRNYPRSSQSIKKRQPPQGFYAVRGKRNTKESD\

## Vasotocin-related

>vasotocin-related_2_Phoronida_Phoronopsis_harmeri_c146046_g1_i1.p1

MKVSQVYWVVCLSIYASETFGCYLWNCARIAGKRQDHVERLQPEQHVYRECMACGLFNQGRCVSETSCNHPDLGVVKFPLCSRENDIPTPCYVRGRACSSNIMRHGKCVAESTCCGEGGCVDDPGCVLLNTPDSDILQYVYVD*

>vasotocin-related_2_Phoronida_Phoronis_psammophila_comp65207_c0_seq1.p1

MNSVYWTICISVCITEGLGCFIWNCPQRGKRVQQHQDMLGLSQCFPCGPLHLGRCVSEAHCNHPALGVIPSTECGNEDKINRPCFVRGRGCSSEIMRYGRCVTDTTCCGEGGCLEDSSCVFGASSEDNMDNRQQTVKRAWRIY

>vasotocin-related_1_Phoronida_Phoronis_ijimai_(P._vancouverensis)_comp30606_c0_seq1.p1

MTPVNGMPISFYVVLLCCVMCCSACFWRNCPAGGKRGLYEMPPANHGRPCTSCGPRNDGQCFGPDICCTPTSGCHIGSEFSRVCSRENENITPCVVAGKPCGDQGNCASDGVCCNTDSCKVDAKCKVNRDAIRLEFLNFLEKLLKQRAHKKSQDKR

>vasotocin-related_2_Phoronida_Phoronis_ijimai_(P._vancouverensis)_comp76163_c0_seq1.p1

MNPITLLACLTALCSTPSLGCFLWNCPRDARGKRVQIPTRREFNQCLPCGLFDAGRCVTESHCNHPTLGVFEFGRCQAENEIGMPCYVRGRGCSSADMRYGKCVADGMCCGEGDFGLILVRQQDIH

>vasotocin-related_2_Phoronida_Phoronis_australis_TRINITY_DN239299_c0_g1_i1.p1

MNTVILIACLAALFSVPSLGCFIWNCPIQRVGKRIQISTTQREVVQCLPCGIFGAGRCVTENHCNHPTQGVFEFERCVAENEIERPCHVLGRDCSSADLRFGKCVADGMCCGEGGCMKDTKCVNVMTSLENEYTT

>vasotocin-related_1_Phoronida_Phoronis_australis_TRINITY_DN239556_c0_g1_i1.p1

MTPTNGISTCVPVVLLCFVMCCSACFWRNCPSGGKRGLYEMPPTNSGPPCASCGPRNEGQCFGPDICCTPTSGCLIGSEYSRVCSRENENIIPCVVAGKPCGDQGNCASDGVCCNIDGCKVDSKCKINRDATRMQFLDYLEKLLKERAQ

>vasotocin-related_2_Brachiopoda_Novocrania_anomala_Nano.rna.tri2.167183.1.p1

MFRNFHRQFQRETGKTRTYVLLHGVLLVLVTISLASSCYVRDCPTGGKRNMDVKGSKRCHRCGPFLIGQCISPELCCSALIGCDKLDGYRKNCQDVNSYVTSDCKLLGQTCGDGAPGRCVMNG

>vasotocin-related_1_Brachiopoda_Terebratalia_transversa_Ttra.rna.tri.6316.1.p1

MKLLSVYSIYLLVAMSNACYLYDCINGKRTIDLRSQRQCQPCGDGKGQCVGRNICCGPSMGCHMGTDVSETCSKEDDISTPCVVKGKPCGTEGNCVVRGICCNAVMCTADKSCSDHIAYSKRLISFMKELMKDDIRK

>vasotocin-related_1_Brachiopoda_Laqueus_californicus_comp44487_c0_seq1.p2

MNLLCVCSMYVVLVSMTNACYLYDCINGKRLIDVRSQRQCQPCGDGKGQCIGRNICCGPSMGCHIGTDVSATCSKEEEISTPCIVKGKPCRAEGNCVIDGICCNAVMCTTDKNCNDHIAYNKRLISFMKELMKNDIRK

>vasotocin-related_1_Brachiopoda_Hemithris_psittacea_comp22826_c0_seq1.p1

MARQRHLIMLHLCVLIIVVSDYASGCYFLDCINGKRGLHSNSKEIHVQKKCGTCLDGLGQCVGPNICCGTTIGCHIGTEVSYHCARENESREPCNVEGKDCGDHGNCVASGICCNSEMCMKMDKCKTSPRYSKKLLIFVRTLIKNRKTKTI

>vasotocin-related_1_Brachiopoda_Glottidia_pyramidata_comp44014_c0_seq1.p1

MLPYPTKMLMKHILVILCALTIASGCFIRNCPSGGKRTMDNFDTSHHRQCMSCANGQGQCVGPSICCVEKGGCHVGTSEAEVCKKENESNTPCIVKGKSCDSSLFMGKCTADGICCNSESCVMDDNCRTVLSSKYSREFLLFVKNLLESAKKEQH

>vasotocin-related_1_Brachiopoda_Lingula_anatina_comp136984_c0_seq1.p1

MATFRCTMLPCSTNMLLKHILALMCALTIASGCFIRNCPSGGKRTMEDFDTSHHRQCMSCADGRGQCVGPSICCVEKGGCHVGTSEADVCKKENESNTPCSVKGKSCDSSLFMGKCTADGICCNSESCVMDDNCRTAFSSKYSKEFLLFVKNLLESAKKDQH

>vasotocin-related_2_Brachiopoda_Glottidia_pyramidata_comp43882_c0_seq1.p1

MNNSQNLVSCGKTWSKLLNRDLMLWLLSLSLLCHCTTGCYVRNCPLGGKRSQLECAACGYMSLGRCMSPGVCCSPSFGCIHADRRGSNGCYEWNSLLTSDCDTGGAHCVTSTRPMVLGKCVSKDTCCSKDGCVRSHICAKPKDTSQSQHIFNERGNDQLKKLTKVDNMDPISVETAGRLMANLLRSSRAQVPNNEVEEDGTDSAYEYGF*

>vasotocin-related_2_Brachiopoda_Lingula_anatina_comp88699_c0_seq1.p1

MNNSHSLVKCGKTWSKLLNRDLILWLLSLSLLCHFTSGCYVRNCPLGGKRSQQLCGACGYLSLGRCMAPSTCCSPSFGCISVGSEADGCYEWNSLLTSGCDISGPRCVSSQSPLVFGKCISKHMCCSKNGCVRSR

>vasotocin-related_1_Brachiopoda_Novocrania_anomala_Nano.rna.tri2.22231.1.p1

MVVRYGAFLLLFFGTVASACFFKNCPTGGKRTMEGVKSDKPETIAKHPMETKDWRQLRKCMSCADGQGQCIGSTICCGHSIGCHIGTELSEHCSKENDNPNPCVITGTKCGDRILRFPGNCVAKGWCCNSESCVMDSHCDTITIDDFRKKYSPEFIRFIRRLIEADSISDQ

>vasotocin-related_1_Nemertea_Cerebratulus_marginatus_comp42993_c0_seq1_m.28251

MKSIPSLWQLSFLVFLCVIISCSGCFIRNCPPGGKRGLNDHELGQRRCMSCAQGAGICVGPDICCGPDIGCHMGTKLSSVCAKENESTVACSVPGRQCGPNNEGKCVANGICCTSDTCASDGLCQMGEDLKPELVKLLQALLAKRAR

>vasotocin-related_1_Nemertea_Cerebratulus_spec_TRINITY_DN33699_c0_g1_i1_m.158313

MKSIPSLWQLSLLVFLCVILSCSGCFIRNCPPGGKRGLNDNALERRQCMSCAQGAGICVGPDICCGPEIGCHMGTKLSSVCAKENESTVACKVPGRQCGPNNEGKCVANGICCTSDTCASDGLCQIGEDIKPELVKLLQALLAKRAR

>vasotocin-related_1_Nemertea_Lineus_ruber_Lvir.rna.tri.4673.1_m.10709

MKSIPSLWQLSFLVFLCVIISCSGCFIRNCPPGGKRGLNDHDLGQRRCMPCAQGAGICVGPDICCGPEIGCHMGTKLSSVCAKENESTTACSVPGRQCGPNNEGKCVANGICCTSDTCASDGLCQTRGDIKPELVKLLQALLEKRSR

>vasotocin-related_1_Nemertea_Paranemertes_peregrina_comp25385_c0_seq1_m.30918

MTSTNQISAILLLTTLTLLVATSSGCFFRNCPRGGKRGLSSSQNVAQRQCMSCMETGVCVGPSICCGHDIGCHIGTEQSSVCAKENESPTPCKVPGAHCGPEGNCVADGICCDSETCTTDSNCKKTDQLKPELIELLKRLLASKRADSSDAR

>vasotocin-related_1_Nemertea_Lineus_longissimus_c46215_g1_i1_m.113050

MKSIPSLWQLSFLVFLCVIISCSGCYIRNCPPGGKRGLNDHVLGQRRCMSCAKGAGICVGPDICCGREIGCHMGTQLSSVCAKENESPTACSVPGRQCGPNNQGKCVASGICCTSDTCASDGMCQGGEDFKPQLVQLLQALLEKRSR

>vasotocin-related_1_Nemertea_Baseodiscus_unicolor_TRINITY_DN95342_c0_g3_i1.p1

MKSVPCLWYHTLAIVFLCTVLNSSGCYIRNCPPGGKRGLTDSEHAFSQRKCMSCANGAGICVGPNICCGSDIGCHMGTKLSGVCAKENDSTTPCLVPGPLCGPNKEGTCVADGLCCTSETCTNDRLCATKSDSLKPELVEL

>vasotocin-related_1_Nemertea_Tubulanus_polymorphus_comp35306_c0_seq1_m.45706

MDRVIAGIRSLACFLAVLTLISLCSGCFVTNCPPGGKRGLPDSTYATRQCMSCMGSGTCVGENICCGVNIGCHIGTKESKVCNKENDSRTPCQVPGKTCGPEEQGNCVANGICCTSDACALDNACKQRSLTQQQRKDELVELLKRVLDRRIAINRRD

>vasotocin-related_1_Nemertea_Notospermus_geniculatus_g10069.t1

MKSIPSLWLLSLLVLLYVVLTSGCFIRNCPPGGKRSLNDHAVGQRRCMACAKGQGICVGPDICCGRDIGCHMGTKLSSVCAKENESTTACFVPGRSCGPSNQGKCVASGICCTSDTCANDGTCRTTDTGMKPELVRLLEELLQRKSR

>vasotocin-related_1_Nemertea_Lineus_lacteus_comp26250_c0_seq1_m.105839

MKSIPSLWQLSFLVFLCVIISCSGCFVRNCPPGGKRGLNEQILGQRRCMSCAQGAGICAGPDICCGREIGCHMGTKLSSVCAKENESTTACTVPGRKCGPNNEGKCVANGICCTSDTCASDGMCHTEGDLKPELVQLIQALLKERSR

>vasotocin-related_1_Nemertea_Malacobdella_grossa_comp24424_c0_seq1_m.32120

MMTTNQLSAMLLLATLSLLVTTSSGCFFRNCPRGGKRGLSENVAPRQCMSCMGSGVCVGPNTCCGHDIGCHIGTEQSKVCAKENESLTPCKVPGMRCGTSGNCVANGICCDSETCTTDATCQRNEQLKPELVELLKRLLASKNFDNSR

>vasotocin-related_2_Nemertea_Tubulanus_polymorphus_comp25948_c0_seq1_m.15412

MVSSKANSEMEIYQLCLLTVVVLSTFLSMTESCVIRTCPRRPGKRSQIVQTPQKECMRCMGDGYCVGLDACCGSFGCVMGTSATVKCRAEANSDEPCVIPGEFKTCGSTGSCYADRICCDRTSCTTDDTCSTPTADQDDRTTRLYKIRSLLRRILAKKAFHA

>vasotocin-related_2__Nemertea_Notospermus_geniculatus_g302.t1

MQDMYRLVPALLVIVCMMVTSSQGQILRTRYPDKKSIDPLKSQLEGASACQRCFNDEGYCVGRGVCCLTNNCIVGSIAAKECSVVNSDCSVPGDHKHCGSNGKCYVDGVCCENDGCKMDDSCSRDNEKLIDLLKRLIHVKHRQLIRRLRIITTSRFIDCFKGCPPLFGKSLLIITISTIKMQDMYRLVPALLVIVCIMVTSSQGQILRTRYPDKKSIDPLKSQLEGASACQRCFNDEGYCVGRGVCCLTNNCIVGSIAAKECSVVNSDCSVPGDHKHCGSNGKCYVDGVCCENDGCKMDDSCSRDNEKLIDLLKRLLQVERPAHEKR

>vasotocin-related_2__Nemertea_Lineus_longissimus_c41389_g1_i1_m.66162

MQDMYRFVPAVVVILCIIVTSSQGQILRTRYEKKGIVQPNSEAQVATECRQCFNGEGYCVGHGVCCLKNNCIIGTIAAKECFGQTTQCRVPGDHKPCGASGKCYAEGVCCEKDGCKMDDTCSNQNEKLIDLLKRLLSYERPNRR

>vasotocin-related_2__Nemertea_Lineus_ruber_Lvir.rna.tri.8134.1_m.17549

MQDMYRFVPAVVVILCMILMSTQGQVLRTRYEKKGINPSSSEAQVMSGCKQCFNGEGYCVGHGVCCLKNNCIIGTIAAKECTAQTTQCRVPGDHKSCGTSGKCYVEGVCCEGDGCKMDETCSNKDEKLINLLKRLLSYERPNRR

>vasotocin-related_2__Nemertea_Cerebratulus_spec_TRINITY_DN35366_c0_g2_i1_m.177882

MPNMYRVVPALVVLICILVTSSEGQLFRIRYPGPDKKSIDPLKSQLEGASECVRCFNGEGYCVGRGVCCLKSNCIVGTIAVKECSQPVSQCHVPGDHKPCGANGKCYVDGVCCENDGCKTDDMCSGDNEKLIDLLKRLLSYEKPAAQERR

>vasotocin-related_2__Nemertea_Cerebratulus_marginatus_comp61910_c1_seq1_m.97726

MQNMYRVVPALLVLICIMLTSSEGQLLRIRYPDKKAIDPLKSELEGVSECPRCFNDEGICVGRGVCCLKDNCIVGSVAKKECSVDASACRVPGVHRPCGAGGKCYVDGVCCENDGCKIDDMCSGDNEKLI

>vasotocin-related_2__Nemertea_Nipponemertes_spec_TRINITY_DN2037_c0_g1_i1_m.41465

MNLLTTAAFVVNILACCHGQVARNRDGRLIEGDAAVNVPKPQQAKPHSGIMPAAKTTTKTGQNRTQRGDPDTDLMNEVALSKMKPRKVEESEKLCRTCLNGKGTCLGRSVCCTKSFCTVGTVECTRVPSEVCLKSKRACSQNGVCGSDGVCCEGRGCFVDHSCSSLRTMLVKLLRYVCANDECHAP

>vasotocin-related_2__Nemertea_Paranemertes_peregrina_comp23429_c0_seq1_m.24224

MDLVQTCVCVLAIMIPMMNCQVIRSRDGRGGEKTETIGDALAEPETSKTDDNFQAGKEGDGSCPSCLGDGHCIAPGICCHSNSCSVGVATCSRAVPEQCLMERKRKVCGRKGFCWTSSVCCEKRGCYLEPNCESAEDRLVRILKRLCKNGC

>vasotocin-related_2__Nemertea_Malacobdella_grossa_comp27320_c0_seq1_m.62525

MDSIQICFCLLAIMLPINCQIIRSRDGRGAEKTEPIVDALADLETNNGQDKQCAQCLGSGRCVASGICCHRTSCTVGVSVCDRVPERCFSSQRRKACGGTGVCGASGVCCERSGCYREMECETTEERIKRILYRLCKDGC

## MS peptide-1 (WWS peptide)

>WWS_peptide (MS-peptide_01) Lineus_longissmus_Nemertea_c45085_g3_i2|m.99829 complete len:136 loc:2053-2460(-)

MMRQCFGPFLIGLIGILICFTQDARGSPLPDNEEMVNEILNKRNAWWSKKSDPALADLSGERMDGENSEQPCLSQAHRIDCYINRCVPDFVECGRLARSRSGFGACKQQHHECALECYPDTNKGPALSLFSGLQG*

>WWS_peptide (MS-peptide_01) Nemertea_hongkongiensis_comp66734_c0_seq1.p1

...KKSTTKTSKDFQEQNSNICQEESSRIDCYVNNCVPAFVACGRMTHLKYDFETCKIQHQQCATSCDQRISAGYI*

>WWS_peptide (MS-peptide_01) Nemertea_Riseriellus_occultus_Contig183.p1

...SDPALTDLSGESMDGENSEQPCLSQAHRIDCYINSCVPDFVECGRLARSRAGFGACKQQHHECALRCYPVVLRRGLRQVL

>WWS_peptide (MS-peptide_01) Nemertea_Baseodiscus_unicolor_TRINITY_DN83064_c0_g2_i1.p1

MKRQCFGPFLIGLVGMLICFTQDARGSSLPDREEMVNEILNKRNAWWTKKSSPSIPDITSGESSEDNSEEPCLSQARRIDCYINNCVPDFVECGRLARSRHGFKQCKSQHHECAVQC

>WWS_peptide (MS-peptide_01) Nemertea_Lineus_lacteus_comp18149_c0_seq1.p1

MMRQCFGPFLIGLVGILICFTQDARGSPLPDNDEMVSEIFNKRNAWWSKKSDPALTDLAGESVDRENSEQPCLSQAHRIDCYINRCVPDFVECGRLARSRSRFGACKQQHHECALQCYPDTNKGPVLSLFSGIQG*

>WWS_peptide (MS-peptide_01) Nemertea_Lineus_ruber_Lvir.rna.tri.35473.1.p2

MKRQCFGQFLIGLVGILICFTQDARGSPLPDNDEMVNEIRNKRNAWWSKKSDPALTDLSGESMDGENSEQPCLSQAHRIDCYINSCVPDFVECGRLARSRAGFGACKQQHHECALRCYPDTNQGPALSLFSGLQG*

>WWS_peptide (MS-peptide_01) Nemertea_Tubulanus_polymorphus_comp27720_c0_seq1.p1

MRLLKHLTGPVLLAITAHLISFISVVHSIPLTDNSDDNIIQDVLQKRNAWWSKKSAPSVPLLQNDAENPYEDICHAESERIDCYIETCVPTFVACGRKTRTELGFNECKVSHHRCAETCGSRIKDLVDSIVDALN*

>WWS_peptide (MS-peptide_01) Nemertea_Cerebratulus_spec_TRINITY_DN32883_c3_g1_i1.p1

MTRQCFGPFLIGLVGILICFTQDARGSPLPDNDEMVNEILNKRNAWWSKKSDPALSDLTSGESMDSGNSEEPCLSQAQRIDCYINNCVPEFVECGRLARSRAGFKLCKQQHHQCALRCYPDTNKGPALSLFSGLHG*

>WWS_peptide (MS-peptide_01) Nemertea_Notospermus_geniculatus_scaffold1173.g24118.t1.p1

...MGEVLDARGSPLPDHNEMVSEILNKRNDARGSPLPDHNEMVSEILNKRNAWWSKKSDPAMTDLTSGESDTSEEPCLSQAQRIDCYINDCVPDFVECGRLARSRGSFNLCKKQHHQCALRCYPDTNKGPALSLFSGLHR*

>WWS_peptide (MS-peptide_01) Nemertea_Notospermus_geniculatus_scaffold74.g3974.t1.p1

...MKEKTISLRDISPSSQNDGMVDKRSFQSLKRQHDETLLWAVFYRSCRITHMLHTSQDQYAAWWSKKSDPALTDLTSGESDTSEEPCLSQAQRIDCYINDCVPDFVECGRLARSRGSFNLCKKQHHQCALRCYPDTNKGPALSLFSGLHR*

>WWS_peptide (MS-peptide_01) Phoronida_Phoronis_australis_TRINITY_DN297286_c2_g2_i1.p1

MKPMDFSVFIGVLGILLDCLKGTCSEGNPLVDSASQPLQNDVYRVLALPEDGRLRKRQAPYGFGGIWFTKRTMPINAAVQHECRFGNCLKKYLDCEREGSTTEEYWACRRYRSACISECALMSKRKL*

>WWS_peptide (MS-peptide_01) Phoronida_Phoronis_psammophila_comp56770_c0_seq1.p1

MKPVDVSIVVGVLGILLECLKGADGKPFRSISTERGDGNQNVEPLIHRIQKRQYSYGGVWFSKRTHPVVSKNVETDTDASRFTTRDIEVKTQATIECYLRKCLEKLLDCGKNVRSRDYQTCRRLRSTCVLECAISGMHLL*

>WWS_peptide (MS-peptide_01) Phoronida_Phoronopsis_harmeri_c56856_g1_i1.p1

MKPLDVSLFLGLIGVLLECFEGSYGKSISNISQKKTDGGRSTNFFFPGIHQEQKRQYSYGGVWFTKRTQPALKKTKSAFSEKELDRLLARDNEVRIESAVVCYLETCVSRFFECGKTATTAEAYLSCRQVRQACILECGRAEKPSS*

## MS peptide-2 (DMF peptide)

>DMF_peptide (MS-peptide_02) Lineus_longissimus_Nemertea_c45028_g1_i1|m.99192 5prime_partial len:139 loc:1619-2035(-)

SYLVACIVVLCACLVLGKPAPSGTKVNSQDISKSINRLIEDLRTHKAAEDHDQKELVKIVLAFAGKLQELQEGIQHIDEVEGQAAKATAKREGSEGGDFLERLDKRGQYYDAELDDGFDMFKKRNQKLNRARNGWLEQ*

>DMF_peptide (MS-peptide_02) Lineus_longissimus_Nemertea_c45028_g1_i2|m.99195 complete len:157 loc:1606-2076(-)

MMKSYLVACIVVLCACLVLGKPAPSGTKVNSQDISKSINRLIEDLRTHKAAEDHDQKELVKIVLAFAGKLQELQEGIQHIDEVEGQAAKATAKREGSEGGDFLERLDKRGQYYDAELDDGFDMFKKRNQKQEKKAAARGEQTKEYASELEKAAKAD*

>DMF_peptide (MS-peptide_02) Lineus_longissimus_Nemertea_c45028_g1_i5|m.99203 5prime_partial len:85 loc:1618-1872(-)

VLCACLVLGKPAPSGTKVNSQDISKSINRLIEDLRTHKGSEGGDFLERLDKRGQYYDAELDDGFDMFKKRNQKLNRARNGWLEQ*

>DMF_peptide (MS-peptide_02) Lineus_longissimus_Nemertea_c45028_g1_i7|m.99206 5prime_partial len:116 loc:1553-1900(-)

VLCACLVLGKPAPSGTKVNSQDISKSINRLIEDLRTHKGSEGGDFLERLDKRGQYYDAELDDGFDMFKKRNQKQEKKAAARGEQTKEYASELEKEKPISEELTKLLDLLDAKKGQ*

>DMF_peptide (MS-peptide_02) Nemertea_Riseriellus_occultus_TRINITY_DN48120_c0_g6_i1.p2

DKRGQYYDAELDDGFDMFKKRNQKQEKKAAAQGEKTKEYANELQKEEPSEELTRLLDLLDAKKGQ*

>DMF_peptide (MS-peptide_02) Nemertea_Cerebratulus_marginatus_comp61943_c1_seq12.p1

MMKTYLVACIVVLCACLVLGKPTSTGSKVNSQDISKSINRLIEDLRLHKGSEGDFLERLDKRGQYYDAELDDGLDMFKKRNQKPAKAN*

>DMF_peptide (MS-peptide_02) Nemertea_Cerebratulus_marginatus_comp61943_c1_seq7.p1

AEDRDQKELIKIVLAFSGKLQELQEGIRHIDQAEGQTAQSAAKREGSEGDFLERLDKRGQYYDAELDDGLDMFKKRNQKLNRARNGWLEQ*

>DMF_peptide (MS-peptide_02) Nemertea_Cerebratulus_marginatus_comp61943_c1_seq16.p1

MMKTYLVACIVVLCACLVLGKPTSTGSKVNSQDISKSINRLIEDLRLHKGSEGDFLERLDKRGQYYDAELDDGLDMFKKRNQKLNRARNGWLEQ*

>DMF_peptide (MS-peptide_02) Nemertea_Cerebratulus_marginatus_comp61943_c1_seq19.p1

MMKTYLVACIVVLCACLVLGKPTSTGSKVNSQDISKSINRLIEDLRLHKVAEDRDQKELIKIVLAFSGKLQELQEGSEGDFLERLDKRGQYYDAELDDGLDMFKKRNQKPAKAN*

>DMF_peptide (MS-peptide_02) Nemertea_Cerebratulus_marginatus_comp61943_c1_seq27.p1

MMKTYLVACIVVLCACLVLGKPTSTGSKVNSQDISKSINRLIEDLRLHKGSEGDFLERLDKRGQYYDAELDDGLDMFKKRNQKPVHEDVVTPQKAQTLGEKGNPEHAAEVIGEQESVSEDLTKLLNLLDEKKGN*

>DMF_peptide (MS-peptide_02) Nemertea_Cerebratulus_marginatus_comp61943_c1_seq8.p1

MMKTYLVACIVVLCACLVLGKPTSTGSKVNSQDISKSINRLIEDLRLHKVAEDRDQKELIKIVLAFSGKLQELQEGIRHIDQAEGQTAQSAAKREGSEGDFLERLDKRGQYYDAELDDGLDMFKKRNQKPAKAN*

>DMF_peptide (MS-peptide_02) Nemertea_Cerebratulus_marginatus_comp61943_c1_seq31.p1

MMKTYLVACIVVLCACLVLGKPTSTGSKVNSQDISKSINRLIEDLRLHKVAEDRDQKELIKIVLAFSGKLQELQEGSEGDFLERLDKRGQYYDAELDDGLDMFKKRNQKLNRARNGWLEQ*

>DMF_peptide (MS-peptide_02) Nemertea_Cerebratulus_marginatus_comp61943_c1_seq29.p1

MMKTYLVACIVVLCACLVLGKPTSTGSKVNSQDISKSINRLIEDLRLHKVAEDRDQKELIKIVLAFSGKLQELQEGSEGDFLERLDKRGQYYDAELDDGLDMFKKRNQKPVHEDVVTPQKAQTLGEKGNPEHAAEVIGEQESVSEDLTKLLNLLDEKKGN*

>DMF_peptide (MS-peptide_02) Nemertea_Cerebratulus_marginatus_comp61943_c1_seq5.p1

MMKTYLVACIVVLCACLVLGKPTSTGSKVNSQDISKSINRLIEDLRLHKGSEGDFLERLDKRGQYYDAELDDGLDMFKKRNQKPVHEDVVTPQKAQTLGEKGNPEHAAEVIGEQGVQEDVIAPQTAQTLGDKGNPEHAAEVLGDGDNEEESVSEDLTKLLNLLDEKKGN*

>DMF_peptide (MS-peptide_02) Nemertea_Cerebratulus_marginatus_comp61943_c1_seq4.p1

MMKTYLVACIVVLCACLVLGKPTSTGSKVNSQDISKSINRLIEDLRLHKVAEDRDQKELIKIVLAFSGKLQELQEGIRHIDQAEGQTAQSAAKREGSEGDFLERLDKRGQYYDAELDDGLDMFKKRNQKPVHEDVVTPQKAQTLGEKGNPEHAAEVIGEQESVSEDLTKLLNLLDEKKGN*

>DMF_peptide (MS-peptide_02) Nemertea_Cerebratulus_marginatus_comp61943_c1_seq14.p1

MMKTYLVACIVVLCACLVLGKPTSTGSKVNSQDISKSINRLIEDLRLHKVAEDRDQKELIKIVLAFSGKLQELQEGSEGDFLERLDKRGQYYDAELDDGLDMFKKRNQKPVHEDVVTPQKAQTLGEKGNPEHAAEVIGEQGVQEDVIAPQTAQTLGDKGNPEHAAEVLGDGDNEEESVSEDLTKLLNLLDEKKGN*

>DMF_peptide (MS-peptide_02) Nemertea_Cerebratulus_marginatus_comp61943_c1_seq11.p1

MMKTYLVACIVVLCACLVLGKPTSTGSKVNSQDISKSINRLIEDLRLHKVAEDRDQKELIKIVLAFSGKLQELQEGIRHIDQAEGQTAQSAAKREGSEGDFLERLDKRGQYYDAELDDGLDMFKKRNQKPVHEDVVTPQKAQTLGEKGNPEHAAEVIGEQGVQEDVIAPQTAQTLGDKGNPEHAAEVLGDGDNEEESVSEDLTKLLNLLDEKKGN*

>DMF_peptide (MS-peptide_02) Nemertea_Cerebratulus_marginatus_comp61943_c1_seq18.p1

MMKTYLVACIVVLCACLVLGKPTSTGSKVNSQDISKSINRLIEDLRLHKVAEDRDQKELIKIVLAFSGKLQELQEGIRHIDQAEGQTAQSAAKREDALKESGMTASSAGGADNAPHVVQQVVLRDPEDEKTSNKIDNLKFVKLLLAAMEKMKARKGKDKKDKKSKGRPSKPTKVLEGSKGSKEISKKGSEGDFLERLDKRGQYYDAELDDGLDMFKKRNQKPAKAN*

>DMF_peptide (MS-peptide_02) Nemertea_Cerebratulus_marginatus_comp61943_c1_seq23.p1

MMKTYLVACIVVLCACLVLGKPTSTGSKVNSQDISKSINRLIEDLRLHKVAEDRDQKELIKIVLAFSGKLQELQEGIRHIDQAEGQTAQSAAKREDALKESGMTASSAGGADNAPHVVQQVVLRDPEDEKTSNKIDNLKFVKLLLAAMEKMKARKGKDKKDKKSKGRPSKPTKVLEGSKGSKEISKKGSEGDFLERLDKRGQYYDAELDDGLDMFKKRNQKLNRARNGWLEQ*

>DMF_peptide (MS-peptide_02) Nemertea_Cerebratulus_marginatus_comp61943_c1_seq28.p1

MMKTYLVACIVVLCACLVLGKPTSTGSKVNSQDISKSINRLIEDLRLHKVAEDRDQKELIKIVLAFSGKLQELQEGIRHIDQAEGQTAQSAAKREDALKESGMTASSAGGADNAPHVVQQVVLRDPEDEKTSNKIDNLKFVKLLLAAMEKMKARKGKDKKDKKSKGRPSKPTKVLEGSKGSKEISKKGSEGDFLERLDKRGQYYDAELDDGLDMFKKRNQKPVHEDVVTPQKAQTLGEKGNPEHAAEVIGEQESVSEDLTKLLNLLDEKKGN*

>DMF_peptide (MS-peptide_02) Nemertea_Cerebratulus_marginatus_comp61943_c1_seq1.p1

MMKTYLVACIVVLCACLVLGKPTSTGSKVNSQDISKSINRLIEDLRLHKVAEDRDQKELIKIVLAFSGKLQELQEGIRHIDQAEGQTAQSAAKREDALKESGMTASSAGGADNAPHVVQQVVLRDPEDEKTSNKIDNLKFVKLLLAAMEKMKARKGKDKKDKKSKGRPSKPTKVLEGSKGSKEISKKGSEGDFLERLDKRGQYYDAELDDGLDMFKKRNQKPVHEDVVTPQKAQTLGEKGNPEHAAEVIGEQGVQEDVIAPQTAQTLGDKGNPEHAAEVLGDGDNEEESVSEDLTKLLNLLDEKKGN*

>DMF_peptide (MS-peptide_02) Nemertea_Cerebratulus_spec_TRINITY_DN32339_c1_g4_i3.p2

MMKSYLVACVVVLCACLVLGKPASSGSKVNSQDISKSINRLIEDLRLHKGSDGGEFLERLDKRGKYFDAELDDGMDMFKKRNQ

>DMF_peptide (MS-peptide_02) Nemertea_Cerebratulus_spec_TRINITY_DN32339_c1_g4_i2.p1

MMKSYLVACVVVLCACLVLGKPASSGSKVNSQDISKSINRLIEDLRLHKVAEDRDQKELVKIVLAFSNKLQELQEGSDGGEFLERLDKRGKYFDAELDDGMDMFKKRNQ

>DMF_peptide (MS-peptide_02) Nemertea_Cerebratulus_spec_Contig4170.p1

MMKSYLVACVVVLCACLVLGKPASSGSKVNSQDISKSINRLIEDLRLHKVAEDRDQKELVKIVLAFSNKLQELQEGIQHIDLTEAQAVRSAVKRQGSDGGEFLERLDKRGKYFDAELDDGMDMFKKRNQKRAPVDDFIQPQQAQVLGDKGNPEHAAEVTGEQGVDEDIIMPQTVQTLGENGNPHHATEVLGEDGDSTEGVDEDIIMPQTVQ

>DMF_peptide (MS-peptide_02) Nemertea_Cerebratulus_spec_TRINITY_DN32339_c1_g4_i1.p1

MMKSYLVACVVVLCACLVLGKPASSGSKVNSQDISKSINRLIEDLRLHKVAEDRDQKELVKIVLAFSNKLQELQEGIQHIDLTEAQAVRSAVKRQDEFKEAGMSATSTGLGSAPHVVQQVVVKAPEDEEKTAHKMDNAKFVKLLLAALEKMKKGKGKGKKDTAKGKGSKPSKEGSKEISKKGSDGGEFLERLDKRGKYFDAELDDGMDMFKKRNQ

>DMF_peptide (MS-peptide_02) Nemertea_Cerebratulus_spec_Contig4169.p1

MMKSYLVACVVVLCACLVLGKPASSGSKVNSQDISKSINRLIEDLRLHKVAEDRDQKELVKIVLAFSNKLQELQEGIQHIDLTEAQAVRSAVKRQDEEKTAHKMDNAKFVKLLLAALEKMKKGKGKGKKDTAKGKGSKPSKEGSKEISKKGSDGGEFLERLDKRGKYFDAELDDGMDMFKKRNQKREDVIVPEKASQTNDEGNLEYLAELLRREKQIEEGIVPQRASQTGQDHGGENLAYLKELLSDSPGVDEGMIMPETAQPLGDK

>DMF_peptide (MS-peptide_02) Nemertea_Lineus_ruber_Lvir.rna.tri.6025.1.p1

MMKTYLVACIVVLCACLVLGKPAPSGTKVNSQDISKSINRLIEDLRTHKAAEDHDQKELVKIVLAFAGKLHELQEGIQHIDEVESQAAKTAAKREDVLTGSGVKASSTNEGDVPHVVQQVVVKAPEAPDNKSKLDNIKFVKLLLTALEKANKEKGKGKPKKEKNGPKKTSKTIEMSKKELDKGSEGGDFLERLDKRGQYYDAELDDGFDMFKKRNQKQEPISEEVTELLSLLDAKKGQ*

>DMF_peptide (MS-peptide_02) Nemertea_Lineus_lacteus_comp26551_c0_seq17.p1

KPAPSGSKVNSQDISKSINRLIEDLRTHKAAEDHDQKELVKIVLAFAGKLQELQEGGQGSDFLERLDKRGQYYDAELDDGFDMFKKRNQKPAKAN*

>DMF_peptide (MS-peptide_02) Nemertea_Lineus_lacteus_comp26551_c0_seq15.p1

AEDHDQKELVKIVLAFAGKLQELQEGGQGSDFLERLDKRGQYYDAELDDGFDMFKKRNQKQEKKAAQGEKAKEYANQLEQEKPISEDVNRLLTLLDAKKGQ*

>DMF_peptide (MS-peptide_02) Nemertea_Lineus_lacteus_comp26551_c0_seq7.p1

KPAPSGSKVNSQDISKSINRLIEDLRTHKGGQGSDFLERLDKRGQYYDAELDDGFDMFKKRNQKQEKKAAQGEKAKEYANQLEQEKPISEDVNRLLTLLDAKKGQ*

>DMF_peptide (MS-peptide_02) Nemertea_Lineus_lacteus_comp26551_c0_seq6.p1

MKSYLVACIVVLCACLVLGKPAPSGSKVNSQDISKSINRLIEDLRTHKGGQGSDFLERLDKRGQYYDAELDDGFDMFKKRNQKLNRARNGWLEQ*

>DMF_peptide (MS-peptide_02) Nemertea_Lineus_lacteus_comp26551_c0_seq23.p1

MKSYLVACIVVLCACLVLGKPAPSGSKVNSQDISKSINRLIEDLRTHKGGQGSDFLERLDKRGQYYDAELDDGFDMFKKRNQKQKPISEDVNRLLTLLDAKKGQ*

>DMF_peptide (MS-peptide_02) Nemertea_Lineus_lacteus_comp26551_c0_seq5.p1

MKSYLVACIVVLCACLVLGKPAPSGSKVNSQDISKSINRLIEDLRTHKAAEDHDQKELVKIVLAFAGKLQELQEGGQGSDFLERLDKRGQYYDAELDDGFDMFKKRNQKLNRARNGWLEQ*

>DMF_peptide (MS-peptide_02) Nemertea_Lineus_lacteus_comp26551_c0_seq25.p1

MKSYLVACIVVLCACLVLGKPAPSGSKVNSQDISKSINRLIEDLRTHKAAEDHDQKELVKIVLAFAGKLQELQEGIQHIDDVESQAVKAAAKREGGQGSDFLERLDKRGQYYDAELDDGFDMFKKRNQKPAKAN*

>DMF_peptide (MS-peptide_02) Nemertea_Lineus_lacteus_comp26551_c0_seq2.p1

MKSYLVACIVVLCACLVLGKPAPSGSKVNSQDISKSINRLIEDLRTHKAAEDHDQKELVKIVLAFAGKLQELQEGIQHIDDVESQAVKAAAKREGGQGSDFLERLDKRGQYYDAELDDGFDMFKKRNQKLNRARNGWLEQ*

>DMF_peptide (MS-peptide_02) Nemertea_Lineus_lacteus_comp26551_c0_seq24.p1

MKSYLVACIVVLCACLVLGKPAPSGSKVNSQDISKSINRLIEDLRTHKAAEDHDQKELVKIVLAFAGKLQELQEGIQHIDDVESQAVKAAAKREGGQGSDFLERLDKRGQYYDAELDDGFDMFKKRNQKQEKKAAQGEKAKEYANQLEQEKPISEDVNRLLTLLDAKKGQ*

>DMF_peptide (MS-peptide_02) Nemertea_Lineus_lacteus_comp26551_c0_seq22.p1

MKSYLVACIVVLCACLVLGKPAPSGSKVNSQDISKSINRLIEDLRTHKAAEDHDQKELVKIVLAFAGKLQELQEGIQHIDDVESQAVKAAAKREDVLTGSGVKASSTNEEGVPHVLQQVVLKAPEADDNLSKLDNPQFVKLLLTALEKLNAKKGKKEKPSKPVKPAKPVKLGTSKGVIKKEEKKGGQGSDFLERLDKRGQYYDAELDDGFDMFKKRNQKPAKAN*

>DMF_peptide (MS-peptide_02) Nemertea_Lineus_lacteus_comp26551_c0_seq1.p1

MKSYLVACIVVLCACLVLGKPAPSGSKVNSQDISKSINRLIEDLRTHKAAEDHDQKELVKIVLAFAGKLQELQEGIQHIDDVESQAVKAAAKREDVLTGSGVKASSTNEEGVPHVLQQVVLKAPEADDNLSKLDNPQFVKLLLTALEKLNAKKGKKEKPSKPVKPAKPVKLGTSKGVIKKEEKKGGQGSDFLERLDKRGQYYDAELDDGFDMFKKRNQKLNRARNGWLEQ*

>DMF_peptide (MS-peptide_02) Nemertea_Lineus_lacteus_comp26551_c0_seq9.p1

MKSYLVACIVVLCACLVLGKPAPSGSKVNSQDISKSINRLIEDLRTHKAAEDHDQKELVKIVLAFAGKLQELQEGIQHIDDVESQAVKAAAKREDVLTGSGVKASSTNEEGVPHVLQQVVLKAPEADDNLSKLDNPQFVKLLLTALEKLNAKKGKKEKPSKPVKPAKPVKLGTSKGVIKKEEKKGGQGSDFLERLDKRGQYYDAELDDGFDMFKKRNQKQEKKAAQGEKAKEYANQLEQEKPISEDVNRLLTLLDAKKGQ*

>DMF_peptide (MS-peptide_02) Nemertea_Notospermus_geniculatus_scaffold1217.g24625.t1.p1

MWYLSFLPYLAFASKIMVNEDQYRATAEVRATVCGLDRLNQGAARNPCQYKTDSMMRSYLNPCQYKTDSMMRSYLVACIVVLCACLVLGKPAPSATKQDISKSINRLIDDLRLHKAAETEDEKELIKIVQAFSAKLRELQEGIQRIDEAKPQEAQNDAKRADVSHGSPTSTAKEGSSPHVIQQIVVKAPEDTDIPSPKPQKDNVEFVRLLLTALEKLNIKKEEGKTNRGKGKKPSSKKAPKGSKKVPNVAKKEVKKGSDGGDFLERLDKRGQYYDAELNDGFDMFKKKRNQKREEELVPQAATETGTGGGNSDYMKELFGDSKRKREEEITPQTTTETGTGGGNSDYMKELFGDSKRKREEEITPQTATETGTGGGNSDYMKELFGDSKRKREEEITPQTATETGTGGGNSDYMKELFGDSKRKREEEITPQTATETGTGGGNSDYMKELFGDSKRKREEEITPQTATETGTGGGNSDYMKELFGDSKRKREEEITPQTATETGTGGGNSDYMKELFGDSKRKREEEITPPTATETGTGGGNSDYMKELFGDSKRKREEDDAFRQGFERLSRMLKKPGSC*

## MS peptide 3 (AGEamide)

>AGEamide (MS-peptide_03) Lineus_longissimus_Nemertea_c41235_g1_i1|m.64909 complete len:254 loc:1203-1964(-)

MTRIYALLAVALLVTQVLSAPVAKQDVGERKAKNKEAANLEEELHKQAGEGKKDTPETSEPSPGHKPRGALGQLMQAAAEEEESQEEKAATTKTEEDAKLQNVIYSIYNNPEVLKALLRAGETKESNEEVAPPMQNGIKRDDEEEELRSNIIKKDGDDLYQPASDPNMDMLLDRYEDLPDDSALYEDDRQYNNVADPDYPDDRLMAEEVHALEGMEPNMNGIEVEDENEKDEDDSEDHDDNEEESTTKSASKQ*

>AGEamide (MS-peptide_03) Nemertea_Cerebratulus_marginatus_comp1386598_c0_seq1.p1

MQRIYALLTIALLITKVLSAPTAKQDVGERKAKNKEAENLEEELHKQAGEGKKDTPETNEPSPGHKPRGALGQLMEAAAEAEKEQAATTKTEDDAKLQNVIYSIYNNPEVLKALLRAGE

>AGEamide (MS-peptide_03) Nemertea_Riseriellus_occultus_TRINITY_DN8824_c0_g1_i1.p1

MTRIYTLLAIALLVTQVLSAPAAKQDVGERKAKNKEAANLEEELHKQAGEGKKDTPETSEPAPGHKPRGALGQLMQAAAEEEESQEEKAATTKTEEDAKLQNVIYSIYNNPEVLKALLRAGETKETNKEVAPPMQNGIKRDEEEEELRSNIIKKDGDDLYPNMDMMMNHYADLPDDSALYEDDRRYNNIPDPDYPDDELMAEEEALEEMEPNMNGIET

>AGEamide (MS-peptide_03) Nemertea_Lineus_lacteus_comp25423_c0_seq6.p1

MTRIYALLAVALLVTQVLSAPVAKQDVGERKAKNKEAANLEEELHKQAGEGKKDTPETSEPSPGHKPRGALGQLMQAAAEEEESQEQKAATTKTEEDAKLQNVIYSIYNNPEVLKALLRAGETKETNEEIVPPMQNGIKRDDEEGELRSNIIKKDGDDLYQPPNMDMQDIPSDRALYEDDQQYNNVADPDYPDDKLMAEEQQALEEMEPDMNDIEAEDEEGEDEEDDSEDHDDEEEESTTKSSQQ*

>AGEamide (MS-peptide_03) Nemertea_Notospermus_geniculatus_scaffold88.g4545.t1.p1

MRNKGENGLQSNVLSAPTVKQNVGERKATNKEAANLEEELHKQAGEGKKDTPETSEPSPGHKPRGALGQLMEAAAEEEESAEEKGATTTTEDDAKLQNVIYSIYNNPEVLKALLRAGETKDTNKEVLPPKTPNGLKRNSDDDDEPLRSNIIKKDGDDLYEPMIASKKIMSDMPIMLQNKYGDVYGENEIFDEDGDVDDDKETGDYPGEPLTADEEAALEAMEDANGIKTDDEQSNESPDDEEQEEDVDEDEEPDDESTTTKRSAQ*

>AGEamide (MS-peptide_03) Nemertea_Notospermus_geniculatus_scaffold425.g13543.t1.p1

MTRIYLLLALALLTTQVLSAPTVKQNVGERKATNKEAANLEEELHKQAGEGKKDTPETSEPSPGHKPRGALGQLMEAAAEEEESAEEKGATTTEDDAKLQNVIYSIYNNPEVLKALLRAGETKDTNKEVLPPKTPNGLKRNSDDDEDDEPLRSNIIKKDGDDLYEPMIASKKIMSDMPMLQNKYGDVYGENEIFDEDGDLDDDKETGDYPGEPLTADEEAALEAMEDANGIKTDDEQSNESPDDEEQEEDVGEDEEPDDESTTTKRSAQ*

>AGEamide (MS-peptide_03) Nemertea_Lineus_ruber_Lvir.rna.tri.14477.1.p1

MTRTYALLAIALLVTQALSAPVAKQDVGERKAKNKEAANLEEELHKQAGEGKKDTPETSEPAPGHKPRGALGQLMQAAAEEEESQEEKAATTKTAEDAKLAQNVIYSIYNNPEVLKALLRAGETKETNKEVAPMENYIKRDDDEEELRSNIIKKDGDDLYQPPSYPNMDMLLDRYDDMPGDRTLYGEDDRRYNNVPDSEYPDDKLMTEEDVKAMKEMKPNMTGIKEDENENEDDNGEDNGKEESTATKSLAQ*

>AGEamide (MS-peptide_03) Nemertea_Baseodiscus_unicolor_TRINITY_DN94851_c0_g5_i2.p1

MARFSVTLTIALLAVKVFTAPTINQEVGERKAKNKEAANLVEELHKEAGEGKKDTPETNEPSPGHRPRGALGEIMAEAEAEEEAAEEEAAEEEAAEEKAAEEKNENETENDDKRNMEKDAGEEEEDDTSDGDKEQVEDDAKMQSVIYSIKNKPEVIKALLRAGETNSDKGSTSREENDIKRGNHVGSNHVGEEEEPSFENEEEEGNDVISPRVLSNIIKKDGDDLYLPMIGSKKVDEQFDGLTLPPEENAIQHHKYKGNGDKISSGHFKEPPGLLPASYAFADYPTSSDEVERKAFERPMETDMRANSGPHEMVDRRQLWPPRSDEEEQNEN*

## MS peptide-4 (GGRWamide)

>GGRWamide (MS-peptide_04) Lineus_longissimus_Nemertea_c32264_g1_i1|m.25175 3prime_partial len:155 loc:184-645(+)

MDGRLLFLVIALSACGSLLACKLPREPTQGEYMKYIVCLQKATGFTSDGGRWGRSVENVLDRSQPLRKLLRSKKTECVLKKNPQPQDYVDYVMCLHSNGHTTEGGRWGRSVDEGLTRDGLKSKSKREECVLKSNPQPQDYVNYVMCLQGTGFTT

>GGRWamide (MS-peptide_04) Lineus_longissimus_Nemertea_c36262_g1_i1|m.37384 5prime_partial len:153 loc:2-460(+)

TTDGGRWGRSIDREFTRNGLKSKSKRDQCVLKSNPQPQDYVNYVMCMQGSGFTSDGGRWGRSTDRARHYSIMEKVFRNVPDFGRWFQLRRESAHEQAEIPARQGGRDDDQETVFSRQANGRVQDLANAGVTIIEHPVKREIRLRDITAKKSS*

>GGRWamide (MS-peptide_04) Nemertea_Riseriellus_occultus_TRINITY_DN75564_c0_g1_i1.p1

SIDRQLTRNGLKSKSKRNQCVLKSNPQPQDYVNYVMCMQGTGFTSDGGRWGRSTDRTKHYTVMERILRNI

>GGRWamide (MS-peptide_04) Nemertea_Baseodiscus_unicolor_TRINITY_DN87702_c0_g1_i2.p1

MGRTSQVCFLLVASFCVVSSTCSLPRNPSNADYVNYVICLQSTRYSTDGGRWGRSVDYQHRRETAKRTQESNGPRCQLNTEPTNSDYVAYVLCLQRSQAGYTSDGGRWGRSIDKKYGRSARSILRRIPDFLTRA

>GGRWamide (MS-peptide_04) Nemertea_Cerebratulus_spec_TRINITY_DN3426_c0_g1_i1.p1

MVVLGAIADTMACRLPNKPKQSDYVEYVACLQQQTGFTTDGGRWGRSVGHILNRIRNILPLSTKKDCKLKKTSDLANFANFLKCVDANSFKTNTVGIPGENRRISNQDRFVTDVLRSGRSIDGALTRNGLKEKSK

>GGRWamide (MS-peptide_04) Nemertea_Cerebratulus_spec_TRINITY_DN15568_c0_g1_i1.p1

DGGRWGRSVDRQLTSNGIRGKEKSKRDQCVLKQNPSTQDYIDYVMCQQNSGFTTDGGRWGRSVDRQLTSNGIRAKETSKRDQCVLKQNPSVQDYIDYVMCQQNSGFTTDGGRWGRSVDRQLTSNGIRGKEKSKRDQCVLKQNPSTQDYIDYVM

>GGRWamide (MS-peptide_04) Nemertea_Cerebratulus_spec_TRINITY_DN1495_c0_g1_i1.p1

DYIDYVMCQQNSGFTTDGGRWGRSVDRQLTQNGILRKSKSKRDECVLKQNPLPQDYVDYVRCLQNNGYTTDGGRWGRSTDRARDHTVMERILRNFPDFYRWFQLRRDAPHEKAEIPSRVGGRDDNQDSVFSRQENGRVQDLADTDVTIIEHPVKR

>GGRWamide (MS-peptide_04) Nemertea_Cerebratulus_marginatus_comp857993_c0_seq1.p1

MYGLVVLAVVLCASDGMLACKLPNKPKESDYMAYVTCLQSQTGFTTDGGRWGRSVDNVLRRVRTFRRTRRSIDRFLSRNGIKQNGKRDQCVLKN

>GGRWamide (MS-peptide_04) Nemertea_Cerebratulus_marginatus_comp32388_c0_seq1.p1

DYINYVTCLENSGFTSDGGRWGRSVDRQLTRNGIMRNSKPKRDECTLKRNPSPQDYVDYVLCMQNDGYTSDGGRWGRSTERAKHRSLMENILRQLPDFHRWFQLRRDSPHEQSEIPTRRGGRDDNQDTVISRQENGRVQDLADRGVTIIEHAHKRRAA

>GGRWamide (MS-peptide_04) Nemertea_Lineus_lacteus_comp20711_c0_seq1.p1

MDSRLLFLVIALSACSSLLACKLPSKPTQQEYMKYIVCLQQATGYTADGGRWGRSVDNVLSRQFRKSRSKKAECVLKPNPQPQDYVDYVRCLHSNGHTTDGGRWGRSIDKRLTRNGLGPKSKRNECVLKPNPQPQDYVDYVMCMQSTGFTSDGGRWGRSIDREFTRNGLKSKSKRNECVLKANPQPQDYYNYVLCMQGSGFTSDGGRWGRSTGRTRHYTIMEKVLRNTPDFGRWFQLRRESAHEQAEIPARQGGRDDDQERVFSRQENGRVQDLANAGVTIIEHPVKREIRLRDIIAKKSS*

>GGRWamide (MS-peptide_04) Nemertea_Lineus_ruber_Lvir.rna.tri.44822.1.p1

MDGNLLFLVIALSACGSLIACKLPSKPTQEEYMKYIVCLQQATGYTSNGGRWGRSVHNDLDRAFEKLRSKKAECVLKSNPQSQDYVNYVMCLQGNGFTSDGGRWGRSVDERLTKRGLESKSKRDECVLKSNPQSQDYVDYVICMHSKGHTTDGGRWGRSVDRELTRTRSGLESKSKRAECALKSNPQPQDYVDYVMCMQGTGFTSDGGRWGRSTDHTKHYRVMEKILRNVPNFGRWFQLRRENAHEQAEIPARQGGRDDDQGRVFSRQENGRVQDLANAGVTIIEHPVKRAIRLRDIIANTSP*

>GGRWamide (MS-peptide_04) Nemertea_Notospermus_geniculatus_scaffold3178.g36394.t1.p1

MCLQQQSGFSTHGGRWGRSDENVLDPRKKSRKVRAKKDECVLKSNPTPQDMIDYAMCSQNNGYTADGGRFGRAADSQVMREGLRSKSKRDTCVLKNNPQPQDFISYAMCLQSNGYTSDGGRWGRSVDGKPTRDVLGSKSKKDTCVLKNNPQPEDFLSYAMCLQSNGYTSDGGRWGRSVDGKLARDGLGSKSKKDTCVLKNNPQPEDFGNGFTSDGGRWGRSVDIELIRGILRSKKDTCVLKNNPQPQDYMNYALCLQGNGFTSDGGRWGRSVDRELSRHQEKSRSKRATCVLKELPRLIDVLAFERCRYGIRFRRSRRSTSRARQHGVLREILREIPNSGRWLPPRRASVHEQPDTPTGGGRDDDLERVVTRQEGGPVEDLTDAGLSVVEQPVKHSAGLRDILSKSKSSSTDI*

>GGRWamide (MS-peptide_04) Nemertea_Notospermus_geniculatus_scaffold3956.g38602.t1.p1

MDGKLVVILVVAFSTFGSLSACTLPNKPTQTEYYNYIMCLQQQSGFSTDGGRWGRSDENVLDPRKKSGKARVKKDECVLKNNPQPQDMIDYAMCLQNNGYTADGGRFGRSVDGEVREGLRSKSKRDTCVLKNNPQPEDFVSYAMCLQSNGYTSYGGRWGRSVDAKLTRGGLRSKSKKDTCVLEDNPQPQDFVSYAMCLQNNGYTSDGGRWGRSVDAKLTRGGLRSKSKKDTCVLEDNPQPQDFVSYAMCLQSNGYTSDGGRWGRSADEKPTRVGLRSRAKKDTCVLKDNPQPQDYMDYALCLQGNGFTSDGGRWGRSVDIELIRGILRSKKDTCVLKNNPQPQDYMNYALCLQGNGFTSDGGRWGRSVDRELSRHQEKSRSKRATCVLKELPRLIDVLAFERCRYGIRFRRSRRSTSRARQHGVLREILREIPNSGRWLPPRRASVHEQPDTPTGGGRDGDVERVVTVTRQEAGPVEDLPDAGVTVVEHPVKHSAGLRDILSKSKSSSTDI*

## MS peptide-5 (GxGH peptide)

>GxGH_peptide (MS-peptide_05) Lineus_longissimus_Nemertea_c37626_g1_i1|m.43692 complete len:114 loc:752-1093(-)

MDTCMKVLFCLYAISMLMMATSRVTNALSSDFGGKPQRREATRGNLMPPGPWIYGTGHLNKRLFKGAFDISNKIEDTAGKEHGIQMMDKFMVFLRLQESGALDKCLASFSKRR*

>GxGH_peptide (MS-peptide_05) Nemertea_Baseodiscus_unicolor_TRINITY_DN160599_c0_g1_i1.p1

LRLQRLGLWRSRRTGHRGTITLYSMDPFLNARTSISNEVEDNVGKMQGIKLMQKFLIFLRLQESGALDKCIGLTANKR*

>GxGH_peptide (MS-peptide_05) Nemertea_Lineus_lacteus_comp15202_c0_seq1.p1

METCMKVLCCLYAISMLMMATSRVTLALNSDFGGQPQRREVTRGNLMPPGPWIYGTGHLNKRLFKGAFDISNKVDDIAGKERGIEIIDKFMIFLRLQESGVLDKCLATLSNRR*

>GxGH_peptide (MS-peptide_05) Nemertea_Lineus_ruber_Lvir.rna.tri.63426.1.p1

MDTCMKVLCCFYAISMLMMGTNRVTHALNSDFGDRPQRREATRGNLMPPGPWIYGTGHLKKRLFKGAFDLSNKLEDVAGKEHGIQMMDKFMFFLRLQESGALDKCLATFAKRR*

>GxGH_peptide (MS-peptide_05) Nemertea_Cerebratulus_spec_TRINITY_DN20868_c0_g3_i1.p1

MEPCTKLLCCLYAISMLMMGPSRLALALTSDFDDRLQRREATRGNLMPPGPWIYGTGHLKKRFYKGAFDVSNKIEESAGKARGIQMMDKFLVFLRLQESGALDKCLATLGVSDRR*

>GxGH_peptide (MS-peptide_05) Nemertea_Paranemertes_peregrina_comp16776_c0_seq1.p1

MGSMWQIYLTLLIVCFSAIQTTSGDTSPWSDAGRPLTRSTMRRNGMPPGPWLYGQGHLQKRMDPGTELKARIATILERIQDEEKKCNCARR*

>GxGH_peptide (MS-peptide_05) Nemertea_Notospermus_geniculatus_scaffold375.g12561.t1.p1

MEYEPCTKLLYCLYAISMLMLGTNRVALADSTGSIDDRLHRREATRGNLMPPGPWIYGTGHLKKRFLKGAFDISNKIEDNQGKVKGIEMMDKFLVFLKLQESGALDKCLATFDLADRR*

>GxGH_peptide (MS-peptide_05) Nemertea_Tubulanus_polymorphus_comp28753_c0_seq3.p1

MFSKATICVLVVSIMVSQSFAKQEIDEFPSTGFERRQQSNVMIPGAWLYGMGHMSKRILGSPKISDKAAIGQGVPMMREFLRYLKLKENRVEPLED*

>GxGH_peptide (MS-peptide_05) Nemertea_Tubulanus_polymorphus_comp28753_c0_seq1.p1

MFSKATICVLVVSIMVSQSFAKGRGGDEPSKEQEIDEFPSTGFERRQQSNVMIPGAWLYGMGHMSKRILGSPKISDKAAIGQGVPMMREFLRYLKLKENRVEPLED*

Leucokinin similarity, F[V/L/I]amide similarity

>Brachiopoda_Terebratalia_transversa_Ttra.rna.tri.10716.1.p2

MKFLIVTLLLFAAAISIFADDEVPLDDEFEETELAPLRNKRNVNCKLSCRPAGSSFTITIQIKGCSRTGAMICCYDCTAVFGRKPRLPPFGRRPTFGGRRRFPPFGGRRTGGWRG*

>Brachiopoda_Laqueus_californicus_comp40919_c0_seq2.p1

WPLRPGKLLVITILLFAAAVSTFADDEVSLDDEFNIDDAEVASLRNKRNTNCKLVCRPRASSFTLTIQIKGCINSGSLVCCYDCKAVFGGRRPNPPFGGRRRNPFGSRRGRYGGGFGGGWRG*

Others:

>AllatostatinA_related_Brachiopoda_Novocrania_anomala_Nano.rna.tri2.74653.1.p1

YFGKRGYTDQSSTKAYGKLADKRQSSMRMHGKRGDKRQGSIRDDIGKLSRIGQGSSMAYGKLDDKRQGSMRGYGEERQDSMMAYGKRGDKRQSSMRNVGKRGDERQDSMRAYGKRGGKRQSSMRNVGKRGDERQDSMRAYGKRG

>RFa_MPCs_Brachiopoda_Novocrania_anomala_Nano.rna.tri2.68150.1.p1

MFEVTVTLLLGILTISWNPNAAVAESSDTSCMDRCSTVGSALIKYCVQIFCSSPVVEVIQANTEEEIAPDFLGEKRDSFWRFGRDSEDAPPPIDSDLKETDGSHERIGRDTGFAFWRFGRAGDNSAAKSIHKTRTKRDSGVYPSLYDPSFRRLLEKTNAYEEIPDYSGRFLEVKRPDTFWRFGREPQEFRQTSDHLIGNVKRSRDSFWRFGRSDGTRDSFWRFGKRNMGTSSQHEREAAPSYTKKDTFWRFGRNWPASSTYWYHQPYISKTFPYLQHNINNIKSKSSLLSENPIHGIAIDVPGQKRTDQFWRFGKRNRVDSVQQHLPRMTPHE*

>AllatostatinA_related_Nemertea_Lineus_longissimus_c47834_g1_i1.p2

MLDMDELGKKSETAESGNTDLNSIKRRLRDFLKYRYMGLSKKRARLFKMWRKFGAIKPSPQLRTRVDPYRYMFGIGK*

>AllatostatinA_related_Nemertea_Cerebratulus_spec_TRINITY_DN32954_c1_g2_i1_m.131299 internal

PDTFAGRKRGPDTFAGRKQGPDTFAGRKQGPDTFAGRKQGSCMPDTFAGRKQGPDTFAGRKQGPDTF

>AllatostatinA_related_Nemertea_Cerebratulus_spec_TRINITY_DN32954_c1_g1_i1_m.131293 internal

KQGPDTFAGRKRGPDTFAGRKRGPDTFAGRKRGPDTLAGRKQGPDTFAGRKRGPDTFAGRKQGPDTFAG

>MPCs_Nemertea_Cerebratulus_spec_TRINITY_DN21013_c0_g1_i2.p2

MLAGKARSHAEGDPMLAGKTRSHAEGDPMLAGKTRCHAERNQMLAGKARSHAERNQMLAGKTRCHAERNQMLAGKAQSHAERNQMLAGKARSHAERNQMLAGKA

>RFa_MPCs_Nemertea_Riseriellus_occultus_TRINITY_DN35367_c0_g1_i2.p2

MQLILDKQCSGLPILEQRAQI

MDSYFIVCLLFLGFTLVAADTDENASELTKRRRPFRWGKREASELTKRLPRYGKREASELTKRLPRFGKQEASELTKREE*

>RFa_MPCs_Nemertea_Cerebratulus_spec_TRINITY_DN17678_c0_g1_i1_m.59192 internal

PRYGKRDADLTKKRALPRYGKRDADLAQKRALPRYGKRDADLTKKRALPRYGKRDADLAQKRALPRYG
