# Supplementary material for: Nemertean, Brachiopod, and Phoronid Neuropeptidomics Reveals Ancestral Spiralian Signaling Systems
Source: Mol Biol Evol. 2021 Jul 17;38(11):4847–66. doi: 10.1093/molbev/msab211 (PMC8557429; doi:10.1093/molbev/msab211)

WWS peptide (MS peptide 1)

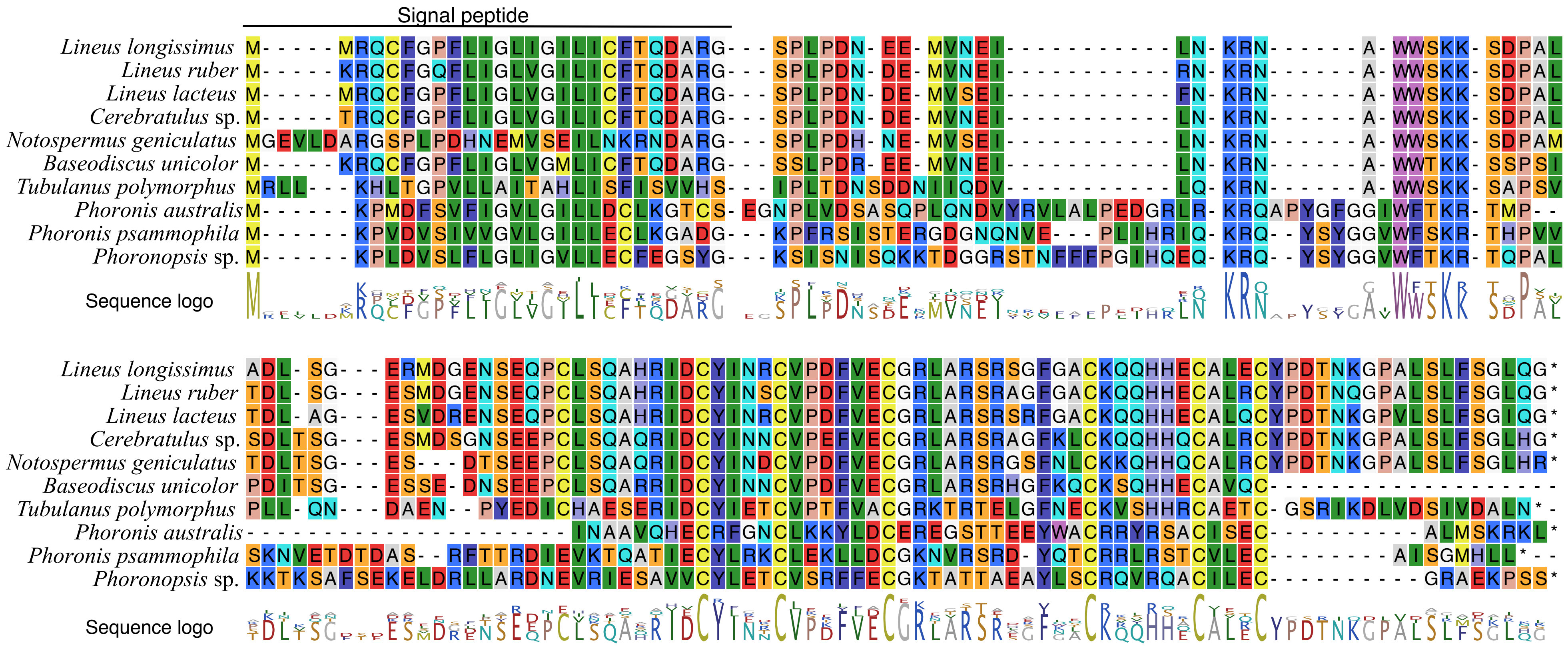

DMF peptide (MS peptide 2)

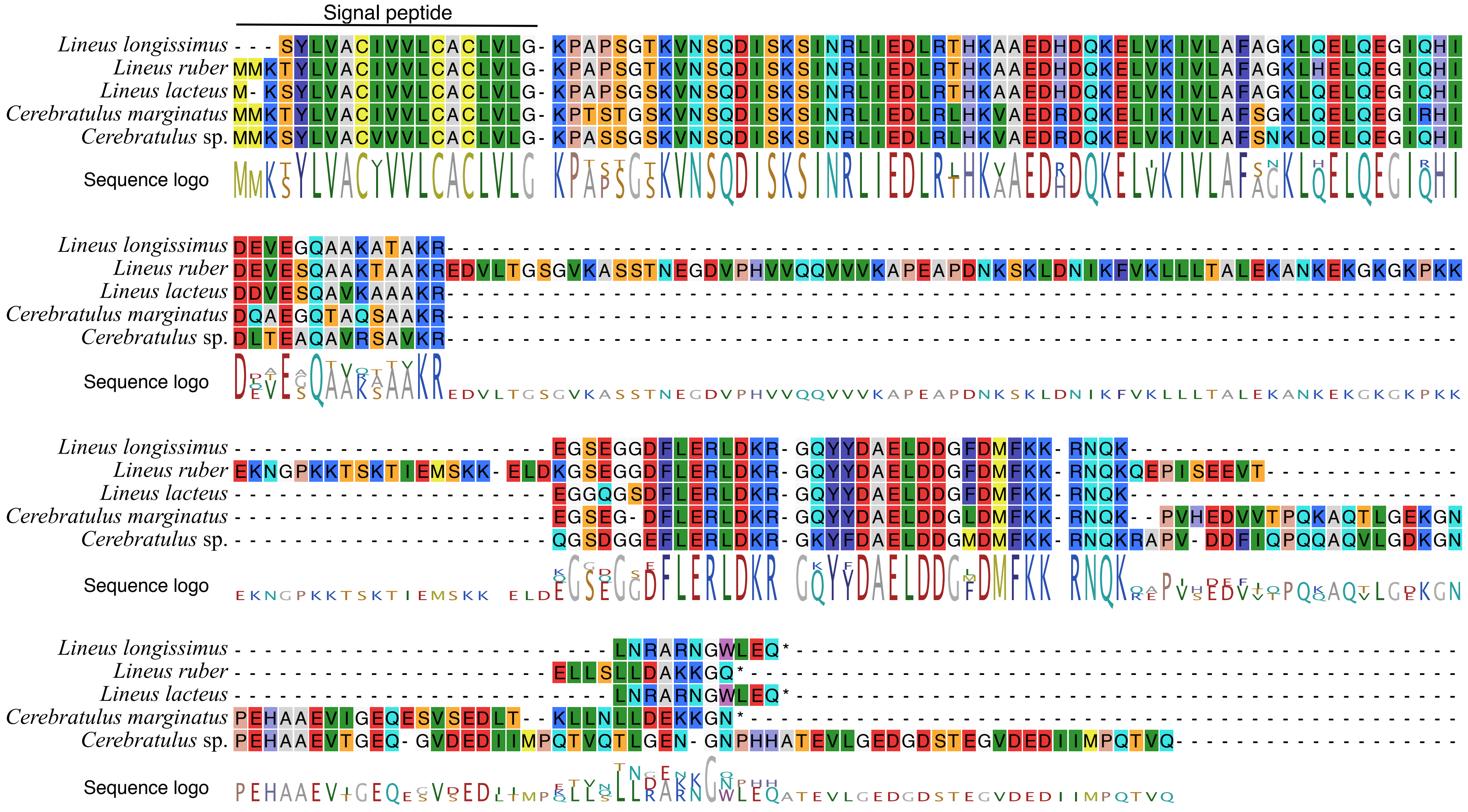

AGEamide (MS peptide 3)

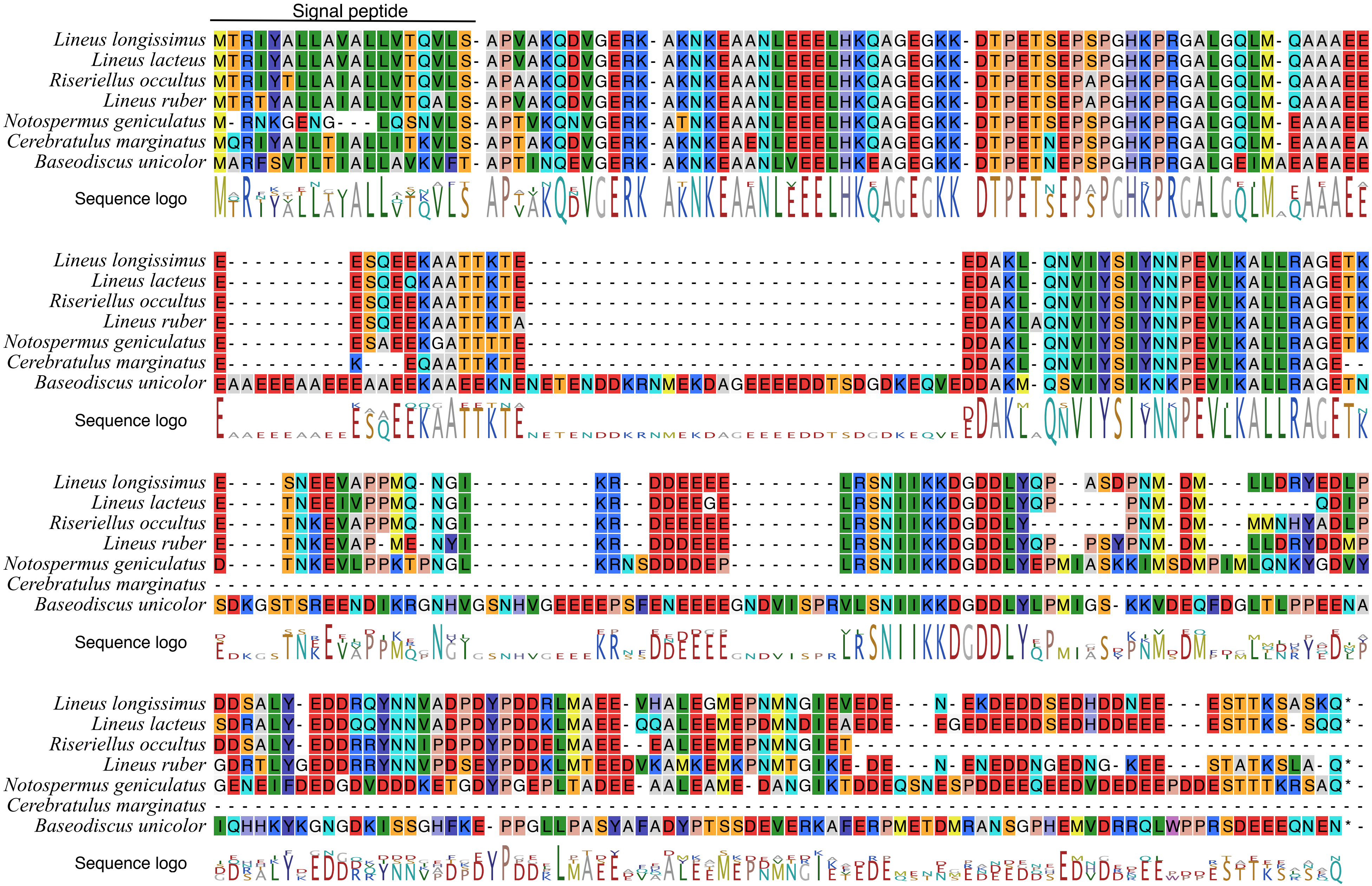

GGRWamide (MS peptide 4)

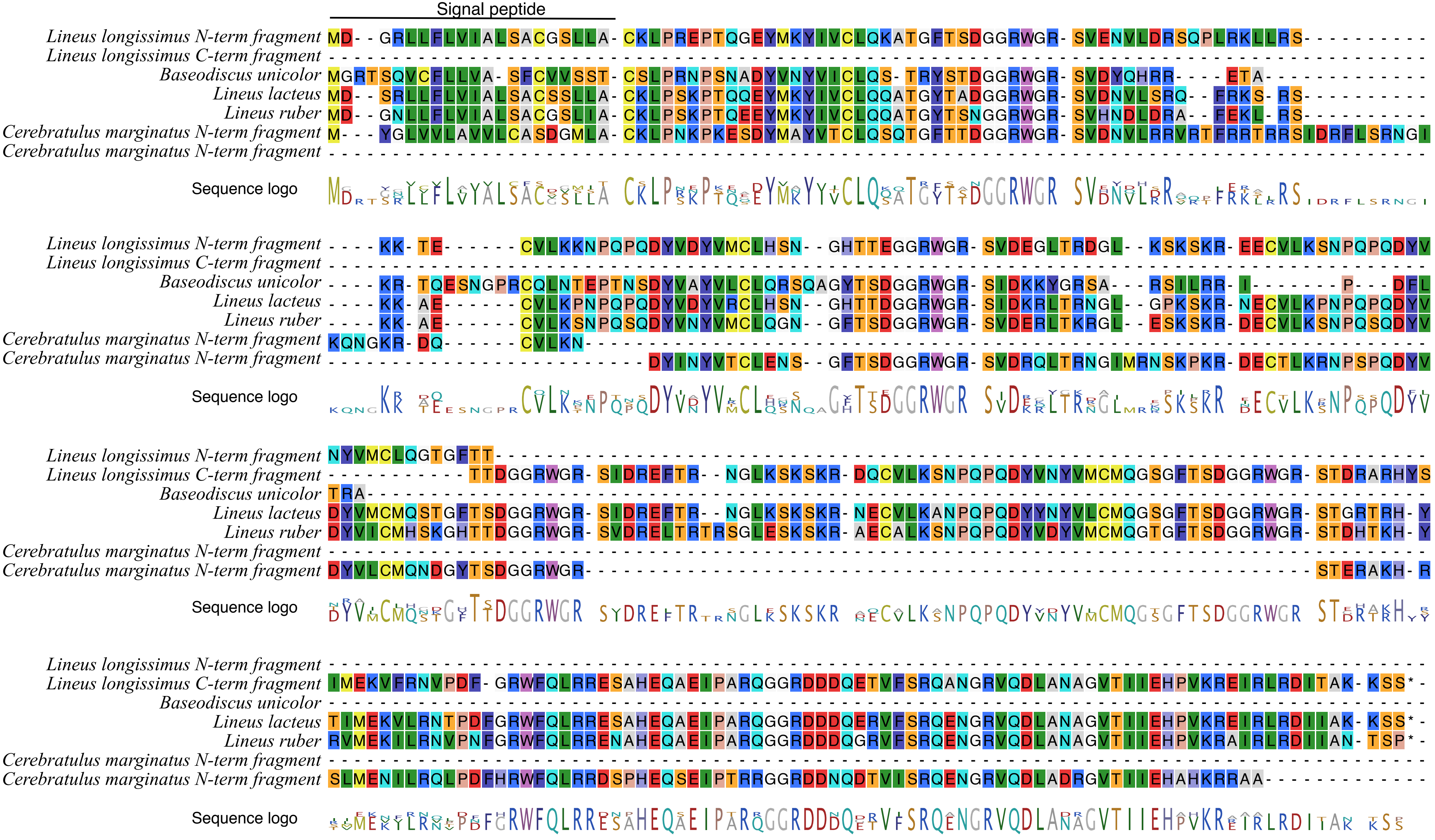

GxGH peptide (MS peptide 5)

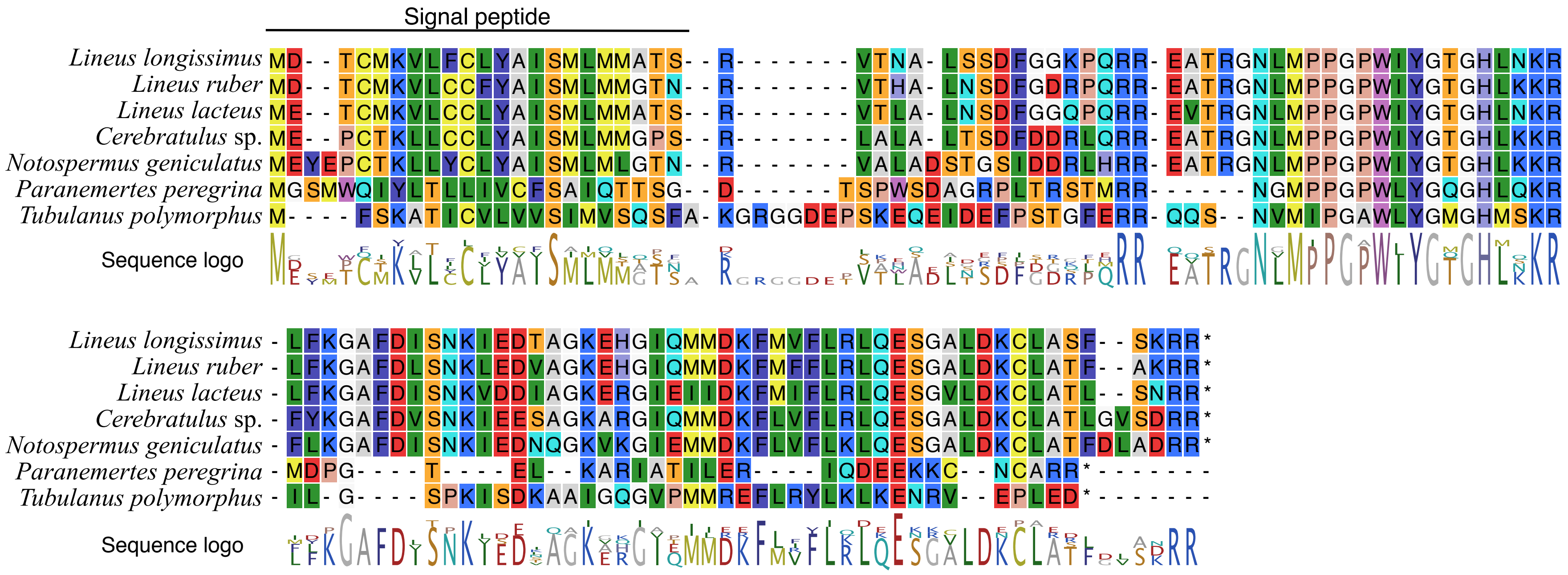

RGWamide/APGWamide

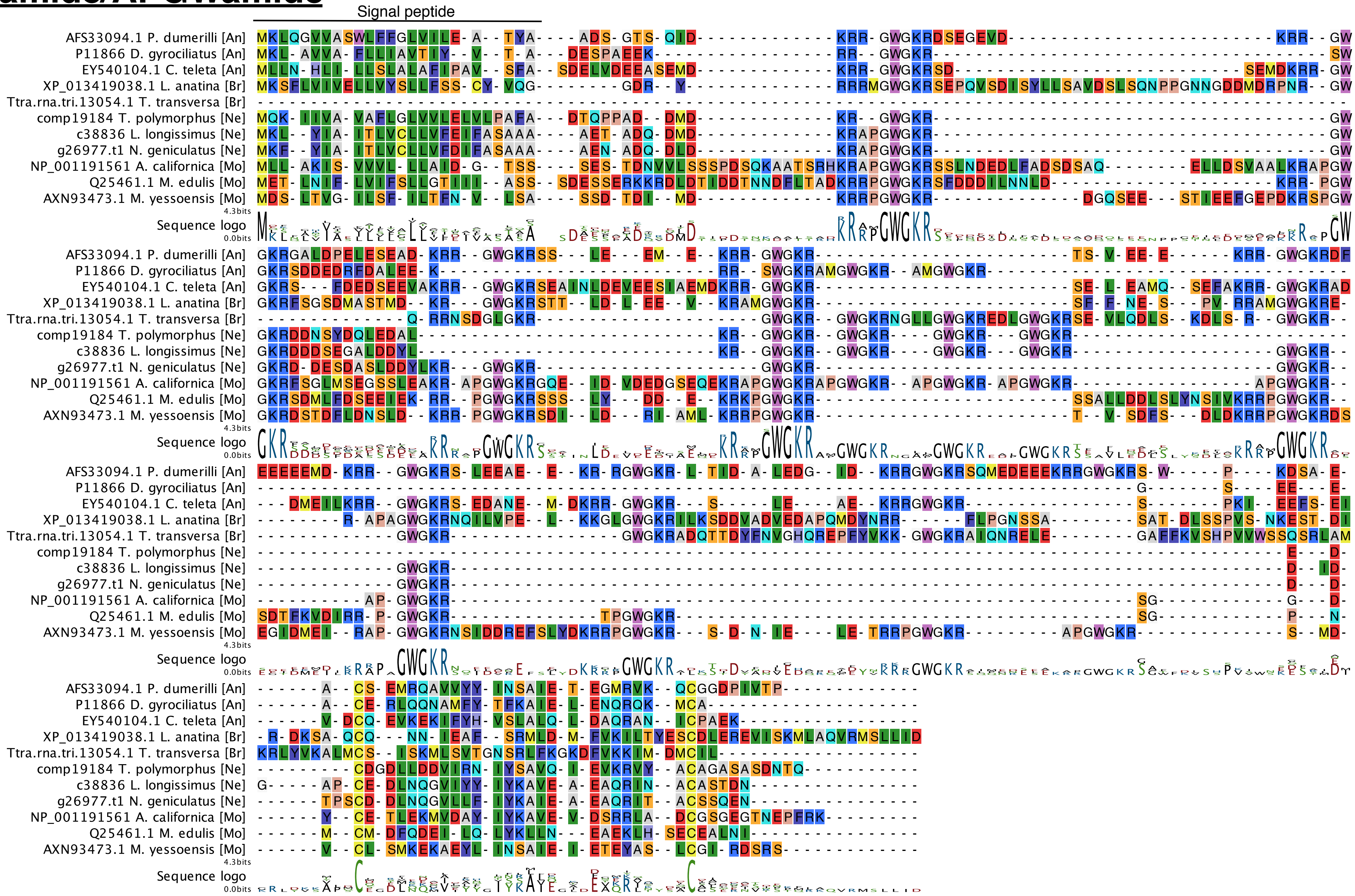

Supplement: msab211_Supplementary_Data [file msab211_supplementary_data.zip › Supplementary_Material_07_Alignments.pdf]
