# Supplementary material for: Nemertean, Brachiopod, and Phoronid Neuropeptidomics Reveals Ancestral Spiralian Signaling Systems
Source: Mol Biol Evol. 2021 Jul 17;38(11):4847–66. doi: 10.1093/molbev/msab211 (PMC8557429; doi:10.1093/molbev/msab211)
Supplement: msab211_Supplementary_Data [file msab211_supplementary_data.zip › Supplementary_Material_19_brachiopod_achatin_correction.docx]

A potential achatin sequence in brachiopods was reported in [De Oliveira AL, Calcino A, Wanninger A. 2019. Extensive conservation of the proneuropeptide and peptide prohormone complement in mollusks. *Scientific Reports*:](http://paperpile.com/b/14jnAQ/y4mf) <http://dx.doi.org/10.1038/s41598-019-40949-0>

This is the potential sequence retrieved from the Supplementary cluster map. Blue indicates the signal peptide, magenta marks basic R/K residues (potential cleavage sites) and yellow marks achatin-peptide like stretches (GxGx). The sequence is published on ncbi as uncharacterized transmembrane protein DDB_G0289901-like (XP_013387045.1), based on an automatic annotation. The sequence lacks canonical cleavage sites, is unusually long for a proneuropeptide sequence (1256 amino acids) and has GxGx stretches similar to some achatin sequences, but not surrounded by potential cleavage sites.

>Brachiopoda lingulaAnatina.g6587.t1

MTAGHARMLLVAIALFSAPWSKHPGGSQARYVDSSSYGSSSSYGDTQDPYSNGDSNGYGDTQDPYGSDDNGDSNGYGDTEDPYGSDGYGDTQDPYSSNGNGDSNGYGDTEDPYGSNGYGDSQDPYSSNDNGDSNGYGDTEDPYGNDGYGDTQDPYSSNGNGDSNGYGDTQDPYGSDGYGDTQDPYGSDGYGDTQDPYGSDGYGDTQDPYSSNGNGDSNGYGDTEDPYGXWLWKHQCCGDGQYDTSLEGCCNEKVYTLSTQDCCNNVYIVTKPKQCLGERGLRYGYPPSSYDTQRQTQQGNNNYYQPPTNGYDSTTPSYGSSSSNGKPKMIGYEDSQMKSTGYDSNVYNGPTTSGYDSMTYGPTTEGYDSMTPSYYQPKIMNGNKPKMTGYGTNGDSMMKMSNGRNSGNGNGPSYPPNSNGNKPMPSGNGMSSSNGQSKTANNNGKPKTLYGYIPKLKGYSTAGNSMMTGYGTNGDSMMKMSNGRNSGNGNGLSYPPNSNGHKSMNSGNGMSSSNGQNKAPNSYKPKMTGYGTNGDSMMKMSNGRNSGNGNGLSYPPNSNGHKSSNSGNGMSSSNGQNKAPNSYKPKMTGYGTNGDSMMKMSNGRNSGNGNGLSYPPNSNGHKSSNSGNGMSSSNGQNKAPNSYKPKMTGYGTNGDSMMKMSNGRNSGNGNGPSYPPNSNGHKSSNSGNGMSSSNGQNKAPNSYKPKMTGYGTNGDSMMKMSNGRNSGNGNGLSYPPNSNGHKSSNSGNGMSSSNGQNKAPNSYKPKMTGYGTNGDSMMKMSNGRNSGNGNGPSYPPNSNGHKSSNSGNGMSSSNGQNKAPNSYKPKMTGYGTNGDSMMKMSNGRNSGNGNGPSYPPNSNGHKSMNSGNGMTSSNGQNKAPNSYKPQMTGYGTNGDSMMKKMLNGLNSGNGNGLSYPPNSNGYQPMNSGNGMSSSNDQNKASNGYPPKMTGYGTNGDSMMKMSNGRNSGNGNGPSYPPNSNGYKPMNSGNGMPSSNGQNKASNSYPPKMTGYSTNGDSMMKMSNGRNSVNGNGPSYPPNSNGYKPMNSGNGMSSSNGQNKAPNSYKPKMTGYGTNGDSMMKMSNGGNSGNGNGPSYPPNSNGYKPMNSGNGMSSSNGQNKAPNSYPPKMTGYGTNGDSMMKKMSNGRNSGNGNGPSYPPTSNGYKPMNSGNGMSSSYGNSGNGNGPSYGPTTPGYDSNTPGYSSGNSGNNSQKKQVGNNNGASTNGITVPSYVMRSNKMNSGGGQQARNQVRGYPYQ

**For a comparison: these are nemertean, annelid, mollusc and hemichordate achatin sequences**

>Nemertea Notospermus geniculatus achatin scaffold1248.g24900.t1.p1

MEFVRFRHRLPVLSKSVAMTTIQTLAALLFLVFCITTCHSVIAEAELCSDDGKCSIKENELVKDKIEDEIVSKLLEVDEKRGFGDKRGFGDKRGFGDKRGFGDKRGFGDKRDKRGFGDKRGFGDKRGFGDKRGFGDKRGFGDKRGFGDKRNGVEHIDFRNPVEEYAYICEILGYPCERSKYATMFAEDALSGDKRGFGDKRGFGDKRGFGDKRGFGDKRGFGDKRGFGNKRGFGDKRGFGDKRGFGDKRGFGDKRGFGDKRGFGDKRGFGDRR*

>Annelida Helobdella robusta achatin jgi|Helro1|177126

MRLSLSKIILLALMLYSVSTSIASAWKAHRTNEDTIDHLLHPSDIRNNSEAPLSLKNKITEKDEDENKNTEENETPDDLNLTNFSDYLRRQIYRGEDFDLDYETVKRGFGDKRGFGDKRGFGDKRGFGDKRGFGDKRGFGDKRGFGDKRGFGDKRGFGDKRGFGDK

>Mollusca Achatina fulica achatin lcl|BAA82260

MASSPHYLLLVVVLVVTLTHITASDFEFLEPDQDVEPFEEDVFLLKRGFADKRGFADKRGFADKRGFADKRGFADKRGFADKRGFADKRGFGDKRDEISPALLRLLQHSYQSQPLPLRGHSAFSRLLARQGLWE

>Deuterostome Saccoglossus kowalevskii achatin lcl|XP_002732147

MASSLLHRIILFLLVSTFLKVRTESSESSPNLHIVGNIQLTELADQGDDALIEIDENEVKRGFGNKREDVVFADDVKRGFGNKRDGFQTILDDEKRGFGNKRAEPEKIYGNTIFGVASLKDLEEDEGRKRGFGNKRDFENSKVSDSDSESVDTIADISELKYGLENKRGFGNKRGSSSMELIDDKRGFGNKRVDTLTELQADDDDKRGFGNKRGFGNKRVDTFEEFQVDDDDKRGFGNKRGFGNKRGFGNKRVDTFEEFQVDDDDKRGFGNKRGFGNKRGFGNKRGFGNKRGFGNKRTETAGYEPDSPLKGFIDKWRKMEEEAVSDRLSEKKNS
