# Supplementary material for: Nemertean, Brachiopod, and Phoronid Neuropeptidomics Reveals Ancestral Spiralian Signaling Systems
Source: Mol Biol Evol. 2021 Jul 17;38(11):4847–66. doi: 10.1093/molbev/msab211 (PMC8557429; doi:10.1093/molbev/msab211)
Supplement: msab211_Supplementary_Data [file msab211_supplementary_data.zip › Supplementary_Material_21_Vasotocin-related_trees.pdf]

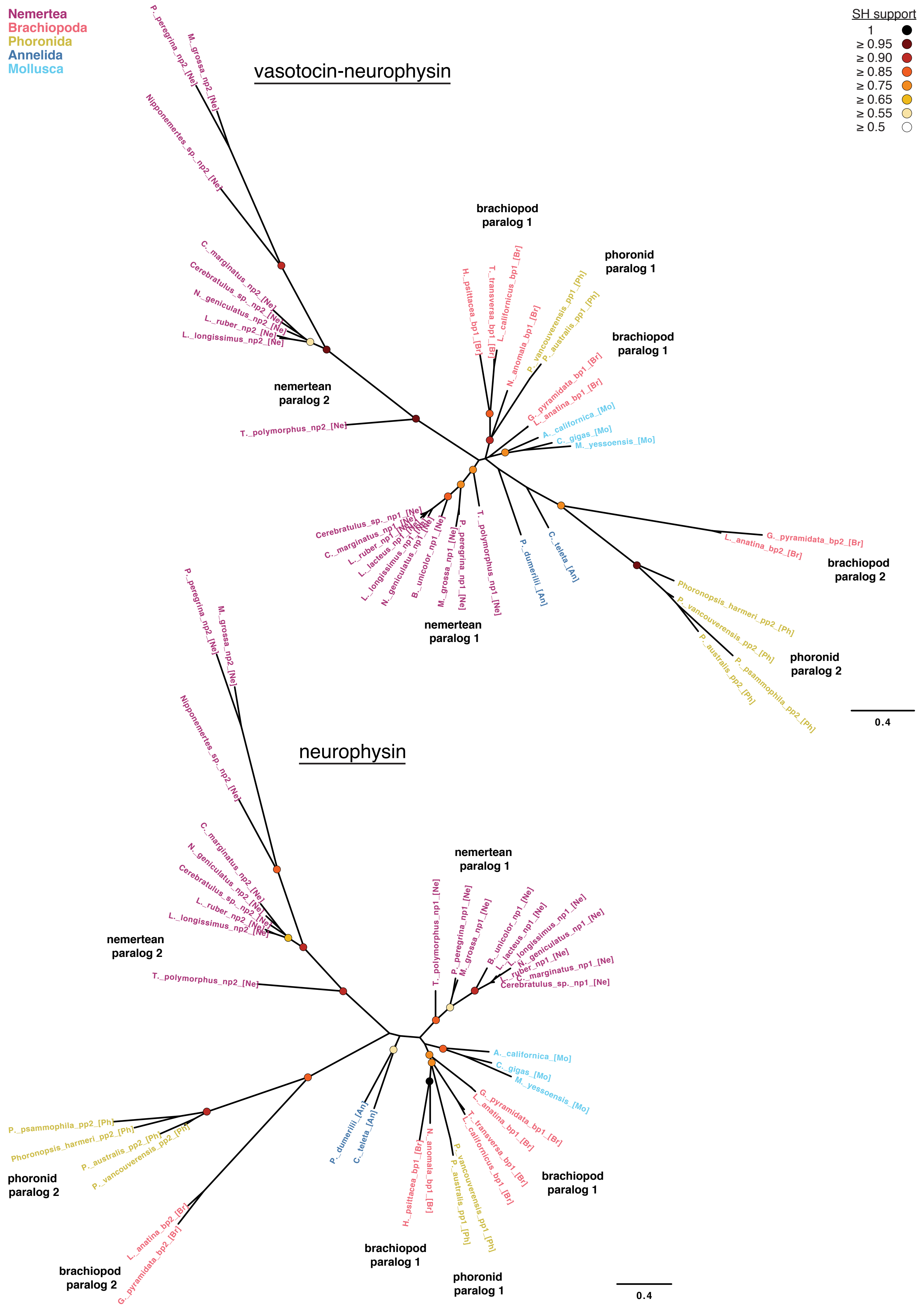
Nemertea
Brachiopoda
Phoronida
Annelida
Mollusca
SH support
1
≥ 0.95
≥ 0.90
≥ 0.85
≥ 0.75
≥ 0.65
≥ 0.55
≥ 0.5
vasotocin-neurophysin
P._peregrina_np2_[Ne]
M._grossa_np2_[Ne]
Nipponemertes_sp._np2_[Ne]
C._marginatus_np2_[Ne]
Cerebratulus_sp._np2_[Ne]
N._geniculatus_np2_[Ne]
L._ruber_np2_[Ne]
L._longissimus_np2_[Ne]
nemertean paralog 2
T._polymorphus_np2_[Ne]
brachiopod paralog 1
H._psittacea_bp1_[Br]
T._transversa_bp1_[Br]
L._californicus_bp1_[Br]
N._anomala_bp1_[Br]
phoronid paralog 1
P._vancouverensis_pp1_[Ph]
P._australis_pp1_[Ph]
brachiopod paralog 1
G._pyramidata_bp1_[Br]
L._anatina_bp1_[Br]
A._californica_[Mo]
C._gigas_[Mo]
M._yessoensis_[Mo]
Cerebratulus_sp._np1_[Ne]
C._marginatus_np1_[Ne]
L._ruber_np1_[Ne]
L._lacteus_np1_[Ne]
L._longissimus_np1_[Ne]
N._geniculatus_np1_[Ne]
B._unicolor_np1_[Ne]
M._grossa_np1_[Ne]
P._peregrina_np1_[Ne]
T._polymorphus_np1_[Ne]
nemertean paralog 1
P._dumerilii_[An]
C._teleta_[An]
G._pyramidata_bp2_[Br]
L._anatina_bp2_[Br]
brachiopod paralog 2
Phoronopsis_harmeri_pp2_[Ph]
P._vancouverensis_pp2_[Ph]
P._australis_pp2_[Ph]
P._psammophila_pp2_[Ph]
phoronid paralog 2
0.4
neurophysin
P._peregrina_np2_[Ne]
M._grossa_np2_[Ne]
Nipponemertes_sp._np2_[Ne]
C._marginatus_np2_[Ne]
N._geniculatus_np2_[Ne]
Cerebratulus_sp._np2_[Ne]
L._ruber_np2_[Ne]
L._longissimus_np2_[Ne]
nemertean paralog 2
T._polymorphus_np2_[Ne]
nemertean paralog 1
T._polymorphus_np1_[Ne]
P._peregrina_np1_[Ne]
M._grossa_np1_[Ne]
B._unicolor_np1_[Ne]
L._lacteus_np1_[Ne]
L._longissimus_np1_[Ne]
N._geniculatus_np1_[Ne]
L._ruber_np1_[Ne]
C._marginatus_np1_[Ne]
Cerebratulus_sp._np1_[Ne]
A._californica_[Mo]
C._gigas_[Mo]
M._yessoensis_[Mo]
G._pyramidata_bp1_[Br]
L._anatina_bp1_[Br]
T._transversa_bp1_[Br]
L._californicus_bp1_[Br]
brachiopod paralog 1
P._vancouverensis_pp1_[Ph]
P._australis_pp1_[Ph]
N._anomala_bp1_[Br]
H._psittacea_bp1_[Br]
brachiopod paralog 1
phoronid paralog 1
P._dumerilii_[An]
C._teleta_[An]
P._psammophila_pp2_[Ph]
Phoronopsis_harmeri_pp2_[Ph]
P._australis_pp2_[Ph]
P._vancouverensis_pp2_[Ph]
phoronid paralog 2
L._anatina_bp2_[Br]
G._pyramidata_bp2_[Br]
brachiopod paralog 2
0.4
