# Supplementary material for: Nemertean, Brachiopod, and Phoronid Neuropeptidomics Reveals Ancestral Spiralian Signaling Systems
Source: Mol Biol Evol. 2021 Jul 17;38(11):4847–66. doi: 10.1093/molbev/msab211 (PMC8557429; doi:10.1093/molbev/msab211)
Supplement: msab211_Supplementary_Data [file Supplementary_Material_08_Annotated_GPCR_trees.pdf › Supplementary_Material_08_Annotated_GPCR_trees.pdf]

a)

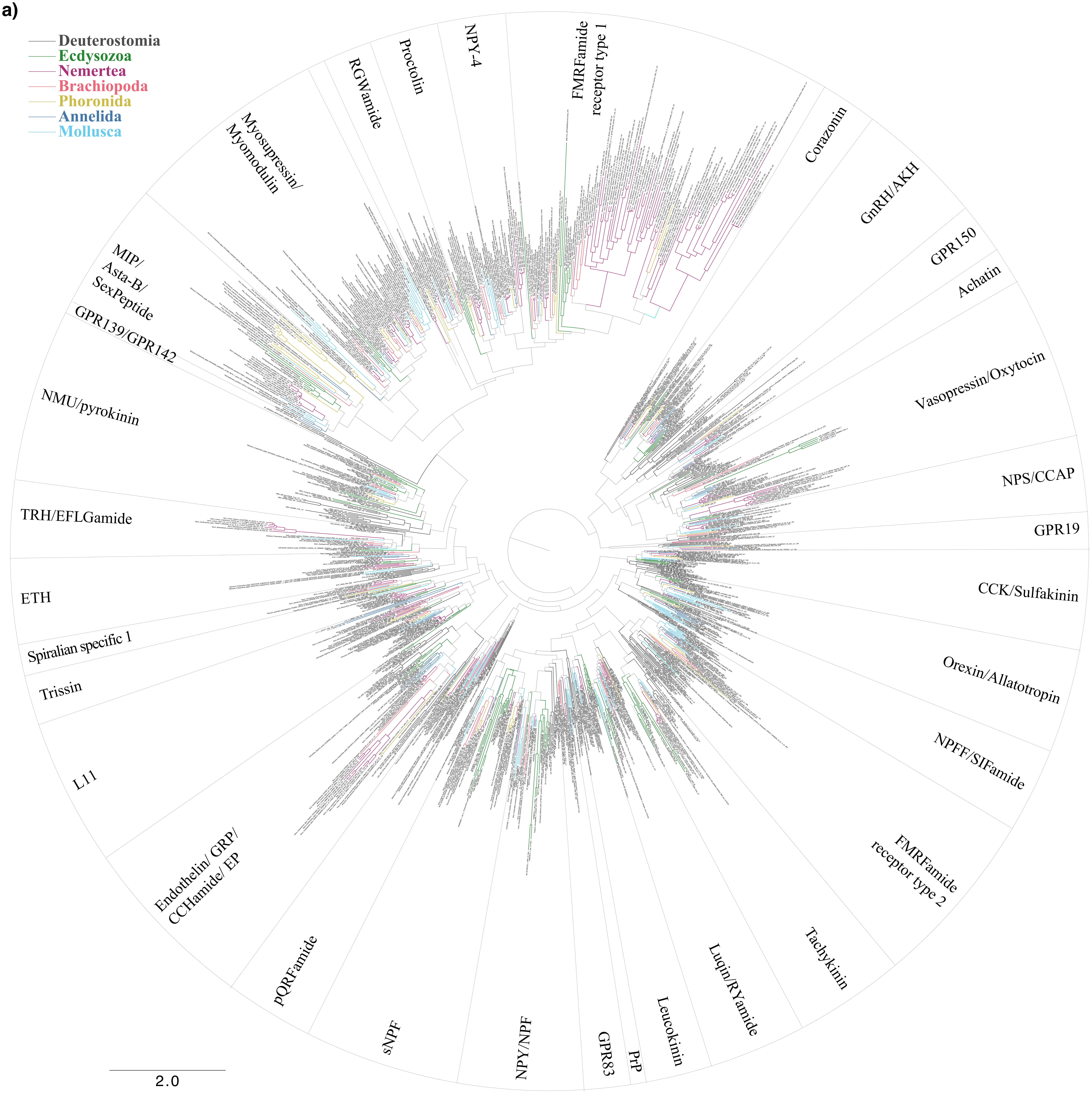
Deuterostomia
Ecdysozoa
Nemertea
Brachiopoda
Phoronida
Annelida
Mollusca
Myosupressin/ Myomodulin
RGWamide
Proctolin
NPY-4
FMRFamide receptor type 1
Corazonin
GnRH/AKH
GPR150
Achatin
Vasopressin/Oxytocin
NPS/CCAP
GPR19
CCK/Sulfakinin
Orexin/Allatotropin
NPFF/SIFamide
FMRFamide receptor type 2
Tachykinin
Luqin/RYamide
Leucokinin
PrP
GPR83
NPY/NPF
sNPF
pQRFamide
Endothelin/ GRP/ CCHamide/ EP
L11
Trissin
Spiralian specific 1
ETH
TRH/EFLGamide
NMU/pyrokinin
GPR139/GPR142
MIP/ Asta-B/ SexPeptide
2.0

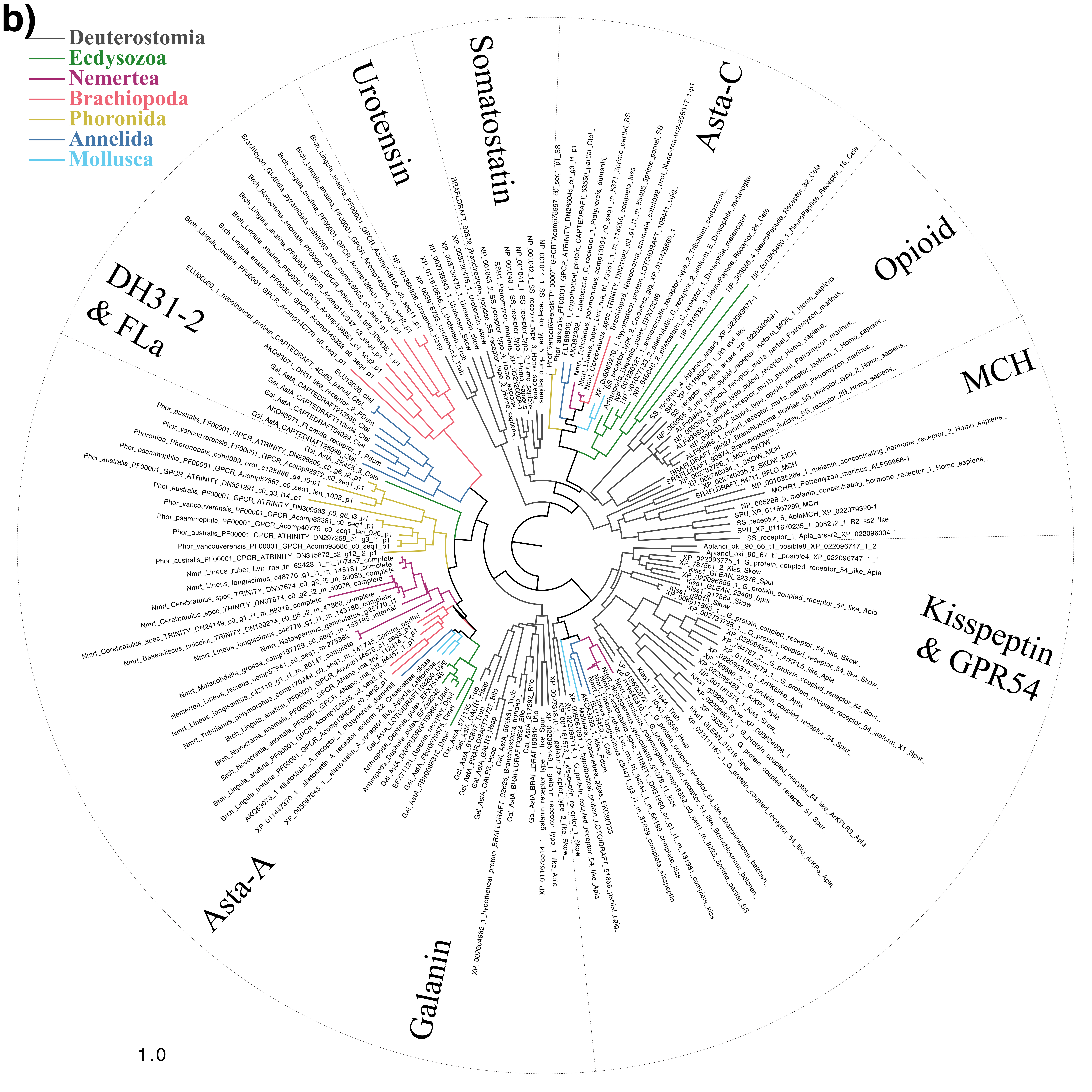
b)
Deuterostomia
Ecdysozoa
Nemertea
Brachiopoda
Phoronida
Annelida
Mollusca
Urotensin
Somatostatin
Asta-C
Opioid
MCH
Kisspeptin & GPR54
Galanin
Asta-A
DH31-2 & FLa
1.0

c)

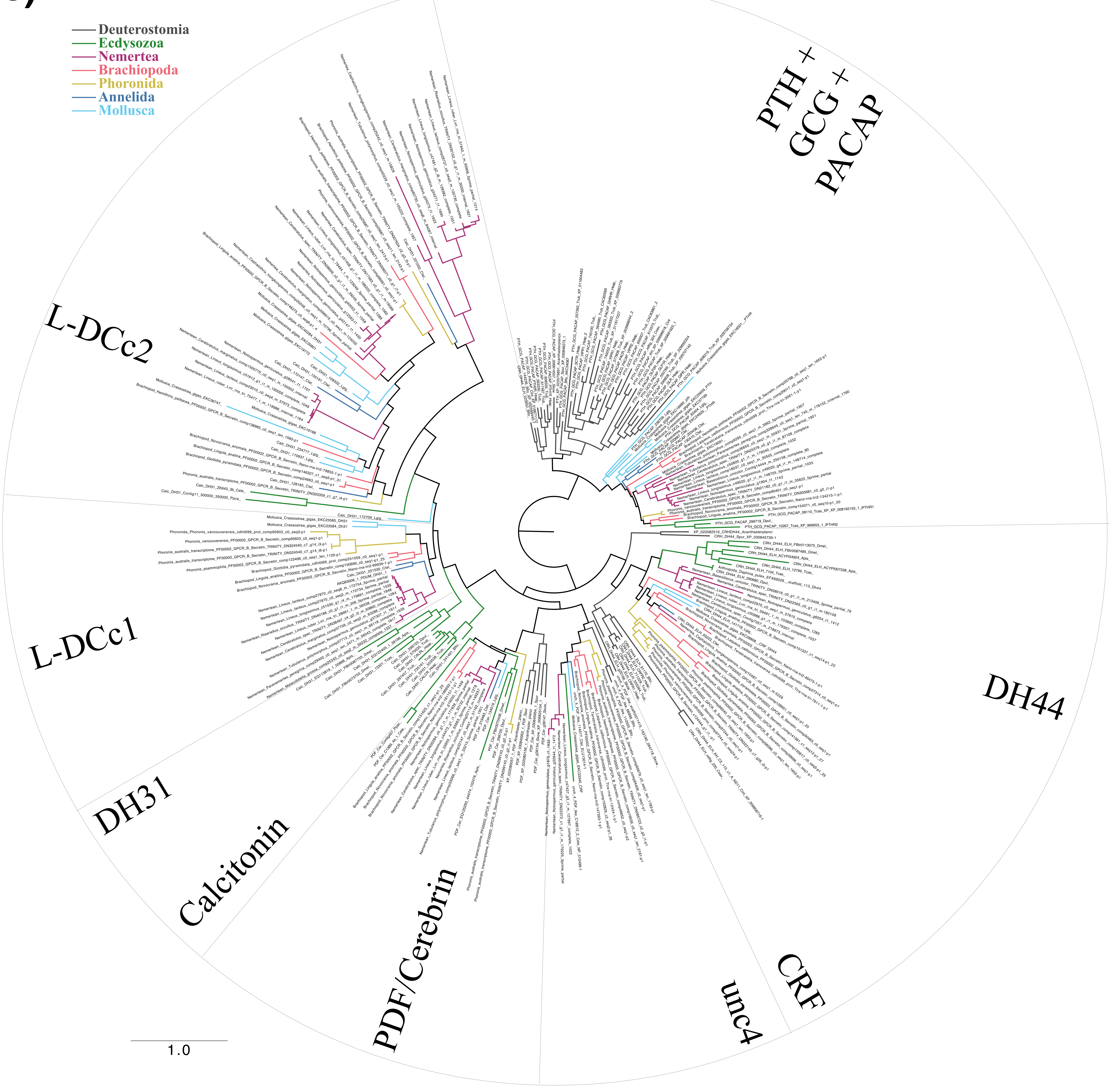
Deuterostomia
Ecdysozoa
Nemertea
Brachiopoda
Phoronida
Annelida
Mollusca
PTH + GCG + PACAP
DH44
CRF
unc4
PDF/Cerebrin
Calcitonin
DH31
L-DCc1
L-DCc2
1.0

Supplementary Figures: Maximum-likelihood analysis of rhodopsin and secretin-type neuropeptide GPCRs. a) Rhodopsin *beta* GPCRs. **b)** Rhodopsin *gamma* GPCRs. **c)** Secretin GPCRs. Terminal branches are colour-coded according to taxon as shown on the upper left. Scale bars on the lower left of each tree indicate the inferred amino acid substitutions per site. Dashed lines demarcate orthologous receptor types. AKH, adipokinetic hormone; Asta, allatostatin; CCAP, crustacean cardioacceleratory peptide; CCK, cholecystokinin; CRF, corticotropin releasing factor; DH, diuretic hormone; ETH, ecdysis triggering hormone; EP, excitatory-peptide; GCG, glucagon, GHS, growth hormone secretagogue; GnRH, gonadotropin releasing hormone; GRP, gastrin releasing peptide; NMU, neuromedin-U; NPS, neuropeptide-S; NPY/NPF, neuropeptide Y, neuropeptide F; MCH, melanin concentrating hormone; MIP, myoinhibitory peptide; PACAP, pituitary adenylate cyclase-activating polypeptide; PBAN, pheromone biosynthesis activating neuropeptide; PDF, pigment dispersing factor; pQRFPa, pyroglutamylated RFamide peptide; PTH, parathyroid hormone; PRP, prolactin releasing peptide; SH, Shimodaira–Hasegawa approximate-likelihood ratio test; TRH, thyrotropin releasing hormone; VIP, vasoactive intestinal peptide.
